# Development of Immobilized Carreira (Phosphoramidite, Olefin) Ligands and Application in Iridium-Catalyzed Asymmetric Allylic Amination

**DOI:** 10.1021/acs.joc.2c02589

**Published:** 2023-01-26

**Authors:** Leijie Zhou, Nicola Zanda, Moreshwar Chaudhari, Mariane Felicio Da Silva, Miquel A. Pericàs

**Affiliations:** † Institute of Chemical Research of Catalonia (ICIQ), The Barcelona Institute of Science and Technology (BIST), E-43007 Tarragona, Spain; ‡ Departament de Química Física i Inorgànica, Universitat Rovira i Virgili, 43007 Tarragona, Spain

## Abstract

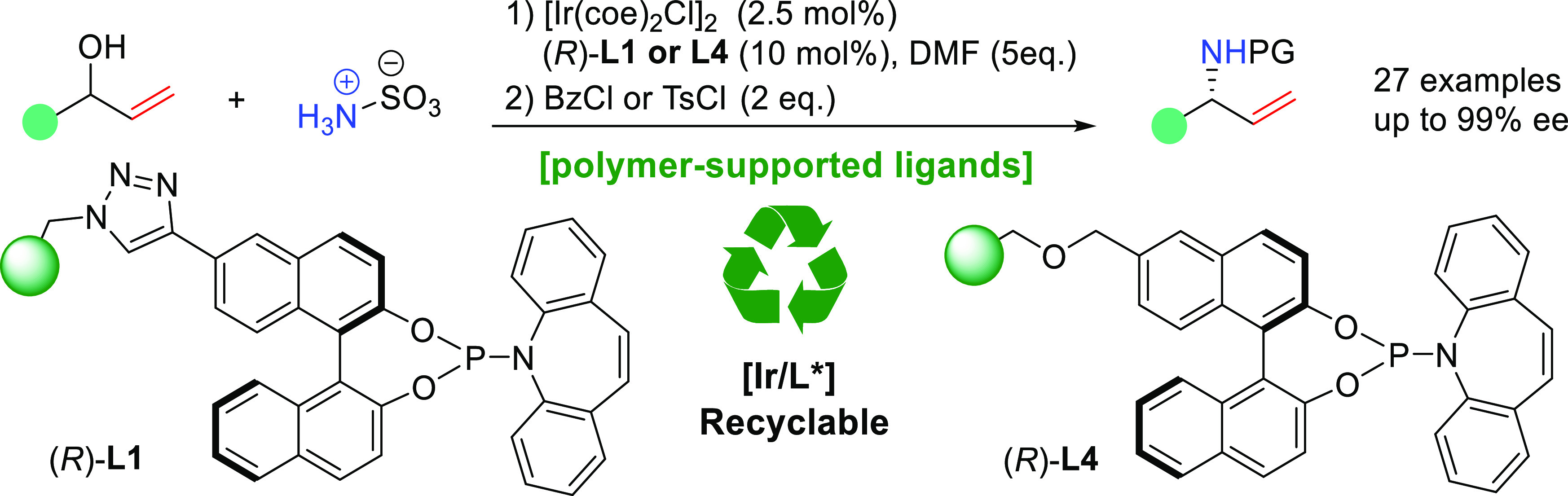

A family of polystyrene-supported (phosphoramidite, olefin) ligands **L1**–**L4**, based on the original design by Defieber and Carreira, has been developed and applied in iridium-catalyzed asymmetric allylic amination of unmasked allylic alcohols (27 examples, up to 99% ee). Among them, functional resins **L1** and **L4** exhibit important advantages such as easy preparation, ligand recyclability, and easy handling for sequential use. As a distinctive advantage, the catalytic use of the iridium complexes of **L1** and **L4** allows the straightforward reuse of a high percentage of the expensive iridium metal involved in the complexes, which is not achievable under homogeneous conditions with the corresponding monomeric complexes.

## Introduction

Transition-metal-catalyzed enantioselective allylic substitution reactions are one of the most powerful tools in synthetic chemistry, with applications in industrial chemical processes. Catalysts derived from Pd, Ir, Mo, Ru, Rh, Ni, and Cu are well explored in the asymmetric allylation process.^1^ Among them, Pd-catalyzed versions (Tsuji–Trost reactions) are the most widely studied and have an inherent tendency of forming chiral, linear substitution products.^2^ In contrast, Ir-catalyzed allylic substitutions usually favor the formation of the branched product with excellent regio- and enantioseletivities.^3^

**Scheme 1:**
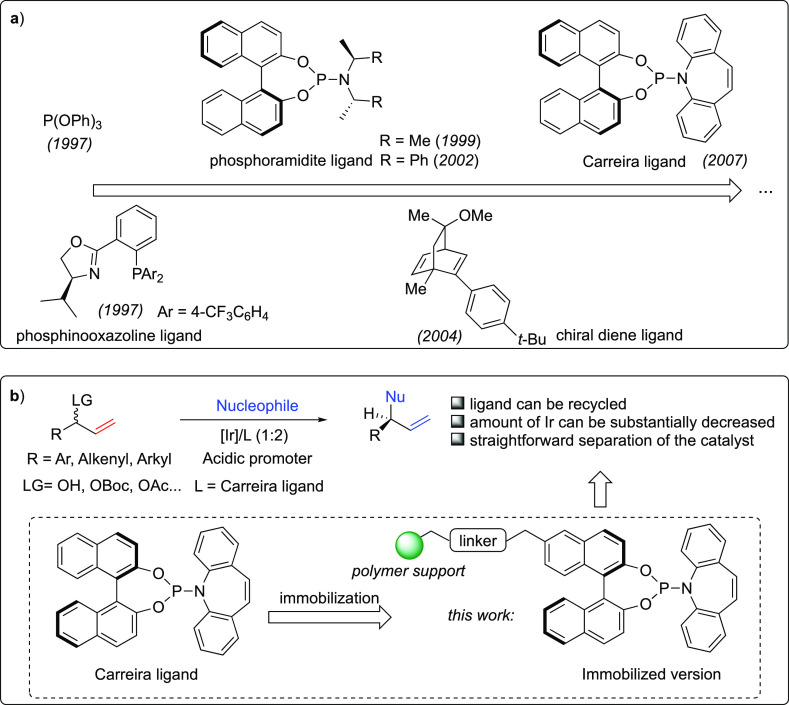
(a) Discovery of the Privileged Carreira Ligand during the Development of Ligands for the Iridium-Catalyzed Allylic Substitution and (b) Comparison with the Immobilized Version in the Asymmetric Allylic Amination Reaction

As a common fact, the development of efficient transition-metal catalysis largely relies on the development of the corresponding ligands. In this respect, the field of Ir-catalyzed asymmetric allylic substitution has benefited from the discovery and use of novel, efficient ligands. In 1997, Takeuchi and Kashio first revealed the potential of iridium catalysis in allylic substitution reactions. They achieved high selectivity on branched products by using P(OPh)_3_ as an achiral ligand to form the catalytic iridium complex with [Ir(cod)Cl]_2_ (Scheme 1a).^4^ The first enantioselective Ir-catalyzed substitution was achieved by the Helmchen group in the same year. Through the introduction of a chiral phosphinooxazoline ligand in combination with [Ir(cod)Cl]_2_, a chiral iridium catalyst was generated in situ. This chiral complex showed excellent catalytic performance, furnishing branched allylic alkylation products with high regio- and enantioselectivities (Scheme 1a).^5^ A few years later, Helmchen and Hartwig groups pioneered the application of the highly efficient Feringa phosphoramidite ligands^6^ to the Ir-catalyzed asymmetric allylic alkylation and allylic amination (Scheme 1a).^7^ Following these reports, many elegant methods were established, and various types of phosphoramidite ligands were further developed with increasingly improved behaviors, thus enabling the fast growth of the area of Ir-catalyzed asymmetric allylation reactions. However, the substrates’ scope was often restricted to linear allylic esters. Naturally abundant and readily available branched allylic alcohols, as well as their easily accessible derivatives, could not be well processed, largely limiting the potential application of this transformation. This limitation was not properly addressed until the discovery by Carreira in 2007 of a novel, privileged type of phosphoramidite ligand incorporating an ancillary olefin function: the (phosphoramidite, olefin) ligand.

In 2004, the Carreira group discovered that a chiral diene ligand enabled the highly selective kinetic resolution of racemic branched allylic carbonates using phenol nucleophiles (Scheme 1a),^8^ which inspired them to explore the combined potential of alkene ligands with the phosphoramidite motif. As a remarkable breakthrough, in 2007 they succeeded in developing the hybrid (phosphoramidite, olefin) ligand. This ligand was employed successfully in the allylic amination of unprotected branched allylic alcohol with the use of sulfamic acids as ammonia surrogates (Scheme 1a).^9^ A few years later, a further development allowed to achieve the direct highly enantioselective version of the reaction.^9c^ This resulted in a useful tool to prepare enantioenriched allylic amines from the racemic, unmasked allylic alcohols. The complex arising from this hybrid ligand and Ir was definitively more robust; thus, a wide variety of reaction conditions could be employed in reactions involving its use. In particular, the tolerance of acid conditions additionally allowed the use of unprotected allylic alcohols, which were directly activated in the reaction. Subsequently, the application of the often-called Carreira ligand and its derivatives was soon widely adopted by the synthetic community in the areas of Rh-catalysis, Ir-catalysis, dual-catalysis, asymmetric photo-catalysis, and also applied in the total synthesis of natural products.^10^

Despite the great progress made over the years, unsolved problems are still existing in the area. For instance, a general interest is to develop protocols that employ a lower loading of the Ir-ligand catalytic complex. Complementary to this is the development of strategies that can allow a facile recovery of the catalyst, both the metal and the ligand.^3^ The implementation of these strategies would result in a rather ideal process from the practical, economical, and sustainability points of view, which would favor an eventual use at the industrial level. Considering the great applicability and the widely spread use of the Carreira ligand, together with the fact that the scarce iridium reserves are under serious danger of depletion due to the increasing use of the metal, we identified the scarcity of iridium as an important sustainability issue of this powerful transformation that could be addressed through an efficient recycling plus reuse strategy.

Over the last few years, our research has been focused on problems of this class and has resulted in the development of a variety of immobilized ligands and organocatalysts,^11^ often suitable for asymmetric continuous flow processes with improved sustainability characteristics.^12^ The immobilization of the catalytic complex into a swellable, microporous polymeric matrix affords the advantages of a heterogeneous catalyst in terms of recyclability (easy separation and reuse) while keeping the properties of homogeneous catalysis, such as minimized mass transfer problems. We envisioned that immobilizing the (phosphinooxazoline, olefin) ≡ (P, olefin) ligand onto a polystyrene (PS)-type support would ensure the re-usability of the Ir-(P, olefin) catalyst while keeping essentially intact its activity and stereoselectivity. Furthermore, several strategies are available for the immobilization of BINOL and SPINOL moieties, which confers a high degree of versatility to the catalyst design.^13^ Herein, we report the preparation of PS-supported species **L1–L4** and their use as robust, recyclable, and highly efficient ligands for the iridium-catalyzed asymmetric allylic amination of unmasked allylic alcohols with sulfamic acid (Scheme 1b).

## Results and Discussion

**Scheme 2:**
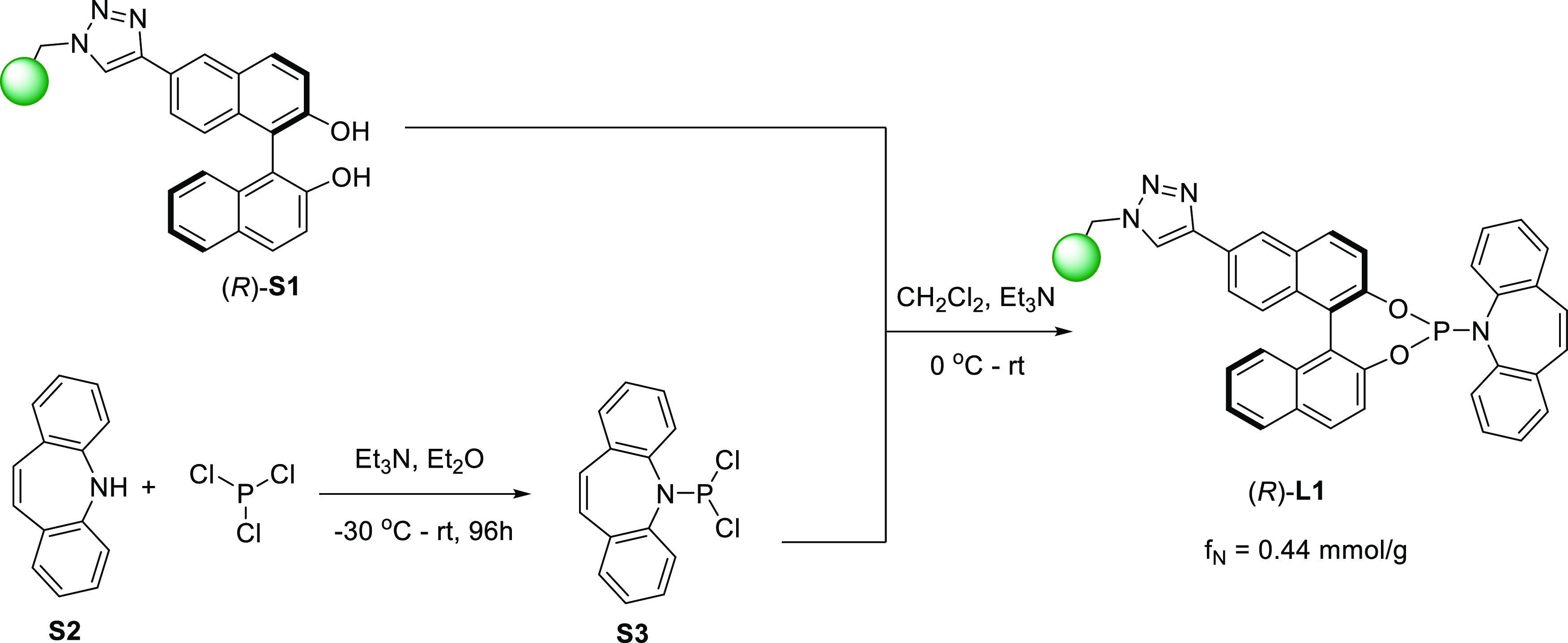
Preparation of the PS-Supported Ligand (*R*)-**L1**

As shown in the original publications, the in situ generated Ir-(P, olefin) complex is relatively more robust and exhibits higher tolerance to air and moisture in comparison with other iridium/phosphoramidite complexes,^9^ and we considered that immobilization onto PS, that places the complex in a hydrophobic environment, would further increase these chemical stability characteristics. Our synthetic design for the preparation of **L1** is based on the previously reported, PS-immobilized BINOL (*R*)-**S1**^13b^ (see the Supporting Information for full details), which has been shown to retain high catalytic activity and recyclability in Ti-mediated allylation reactions. As shown in Scheme 2, our route to **L1** was designed in order to minimize the steps involving reactions on the heterogeneous support and simply involved the convenient coupling of (*R*)-**S1** with readily available 5-(dichlorophosphanyl)-5*H*-dibenzo[*b*,*f*]azepine **S3** under mild reaction conditions.^14^ In this manner, the functional resin (*R*)-**L1** was obtained with a functionalization value of *f*_N_ = 0.44 mmol/g, appropriate for catalytic use.

Having **L1** in hand, we decided to test it in the iridium-catalyzed enantioselective allylic amination reaction. Following the reported homogeneous protocol,^9^ we selected racemic allylic alcohol (±)-**1a** and sulfamic acid **2** as the model substrates to optimize the reaction conditions. We used BzCl to conveniently isolate the Bz-protected amine product (Table 1). To prevent the physical deterioration of resin **L1**, the reaction mixtures were stirred with a shaker instead of a magnetic stirrer. To our delight, in the presence of [Ir(coe)_2_Cl]_2_ as the iridium source and with DMF as the solvent, we could obtain the chiral allylic amine product **3a** in 53% yield and 88% ee (entry 1). A subsequent solvent screening showed that the use of DCE led to small improvements in both yield and enantioselectivity. This solvent, however, was not further considered due to solubility issues with sulfamic acid **2** (entry 2). Using the industrially preferred 2-MeTHF, better contact between reactants and catalyst resulted in a higher yield of product **3a**, along with slightly higher ee (63% yield and 91% ee; entry 3). We next explored the effect of the ratio of Ir/ligand by decreasing or increasing the amount of functional resin (entries 4–5), but no improvements were recorded. To compare the influence of different axially chiral diols and immobilization strategies, we synthetized ligand (*R*)-**L2** from the corresponding 6,6′-bis(styryl) monomer through a copolymerization strategy (see the Supporting Information for full details). Lower yield and lower stereoselectivity were recorded when (*R*)-**L2** was used (entry 6), which can be ascribed to a lower adaptability of the chiral polymeric backbone to the diastereomeric transition states of the reaction. A convenient modification of previously reported (*R*)-SPINOL@polystyrene^13d^ allowed us to prepare (*R*)-**L3** as a SPINOL-derived Carreira ligand. Similar to its homogeneous counterpart, the Ir-**L3** catalyst only exhibited poor catalytic activity and enantioselectivity in the allylic amination reaction (entry 7). As a final parameter, we also tested with ligand (*R*)-**L1** the effect of decreased or increased amounts of catalytic complex (entries 8–9) with no positive results.

**Table 1:**
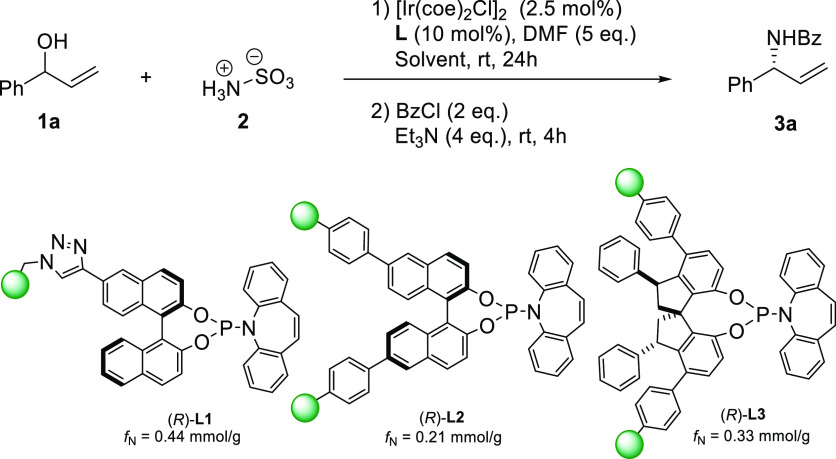
Optimization of Reaction Conditions^a^

entry	ligand	solvent	yield [%]^b^	ee [%]^c^
1	(*R*)-**L1** (10 mol %)	DMF	53	88
2	(*R*)-**L1** (10 mol %)	DCE	56	90
**3**	**(*R*)-L1(10 mol %)**	**2-MeTHF**	**63**	**91**
4	(*R*)-**L1** (5 mol %)	2-MeTHF	56	88
5	(*R*)-**L1** (15 mol %)	2-MeTHF	62	89
6	(*R*)-**L2** (10 mol %)	2-MeTHF	49	71
7	(*R*)-**L3** (10 mol %)	2-MeTHF	12	18
8^d^	(*R*)-**L1** (2 mol %)	2-MeTHF	38	92
9^e^	(*R*)-**L1** (20 mol %)	2-MeTHF	62	89

a Reaction conditions: **1a** (0.25 mmol), **2** (0.30 mmol), [Ir(coe)_2_Cl]_2_ (2.5 mol %), DMF (5 equiv), rt, 1.2 mL of solvent.

b All yields are isolated yields after two steps.

c ee measured by chiral HPLC.

d 0.5 mol % [Ir(coe)_2_Cl]_2_ was used.

e 5 mol % [Ir(coe)_2_Cl]_2_ was used.

We next investigated the generality of the heterogeneous Ir-catalyzed asymmetric allylic amination process with immobilized ligand (*R*)-**L1**. As shown in Table 2, the protocol was robust, and although the reactions were generally performed at a 0.25 mmol scale, the process could also be performed on a 2.5 mmol scale. In this experiment, using allyl alcohol **1a** as a starting material, product **3a** was isolated with a 55% yield and 91% ee. In addition, the free amine could also be protected in situ with TsCl, successfully affording the corresponding Ts-protected allylic amine **4a** in a 39% yield (two steps). A wide variety of 1-aryl substituted allyl alcohols **1a–s** bearing either electron-donating or electron-withdrawing substituents on the aryl group, as well as some 1-hetaryl (**1t**), 1-alkenyl (**1u**), and 1-alkyl or cycloalkyl substituted allyl alcohols (**1v–1y**), were examined. As a general trend, the 1-aryl substituted allyl amines (**3a–s**) were obtained in good yields and with high to very high enantioselectivities. Among them, some substrates bearing electron-deficient aryl substituents afforded the best enantioselectivities (**3h–3i**, **3m–3o**, up to 94% ee). In contrast, some substrates bearing electron-rich aromatic substituents afford the corresponding products with lower enantioselectivity. An example of this was compound **3g**, which was isolated with 80% ee. Fortunately, this issue could be later addressed with the use of the immobilized (P, olefin) ligand **L4** (see below). To our delight, an indole-derived allylic alcohol **1t** was reactive in this heterogeneous process. It delivered the amine product **3t** in 53% yield and 79% ee. A cinnamaldehyde-derived, doubly allylic alcohol **1u** was also examined in the reaction, affording the corresponding protected amine **3u** in 42% yield and 75% ee. It has to be noted that only the branched product was formed, with complete preservation of the chemo- and regioselectivity of the homogeneous Ir-(P, olefin) catalyst. Aliphatic allylic alcohols, that were reported to be rather inactive in previously developed allylic substitution processes, reacted sluggishly, affording the products with good enantioselectivity, albeit in low yields. At a difference with the homogeneous process, decreasing the ligand loading from 10 to 5 mol % did not improve the reactivity, probably due to the low reactivity of aliphatic alcohol being further magnified in the heterogeneous system.

**Table 2:**
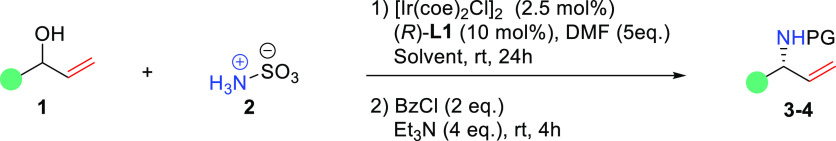

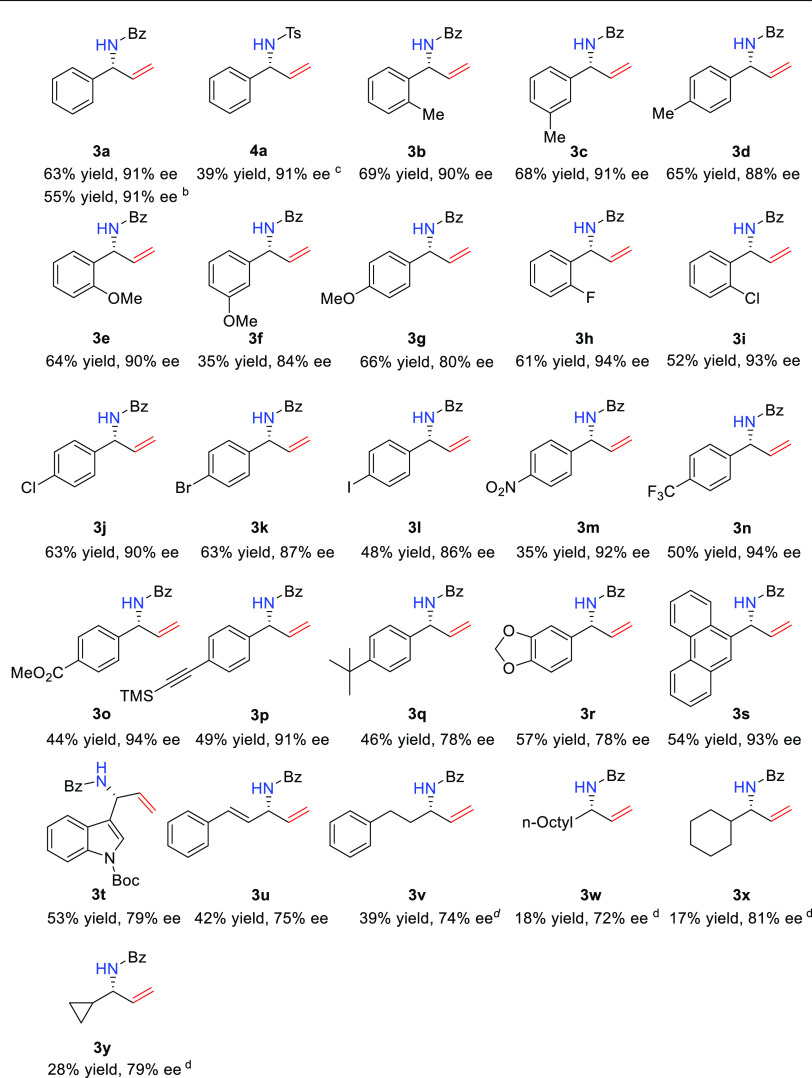
Scope of the Ir-(P.olefin)-Catalyzed Asymmetric Allylic Amination Mediated by PS-Immobilized Ligand **L1**^a^

a Reaction conditions: **1** (0.25 mmol), **2** (0.30 mmol), [Ir(coe)_2_Cl]_2_ (2.5 mol %), (*R*)-**L1** (10 mol %), DMF (5 equiv), rt, 2-MeTHF (1.2 mL). Absolute configurations were assigned by comparison with literature data.

b Reaction was performed at a 2.5 mmol scale.

c TsCl was used instead of BzCl.

d 5 mol % (*R*)-**L1** was used.

**Scheme 3:**
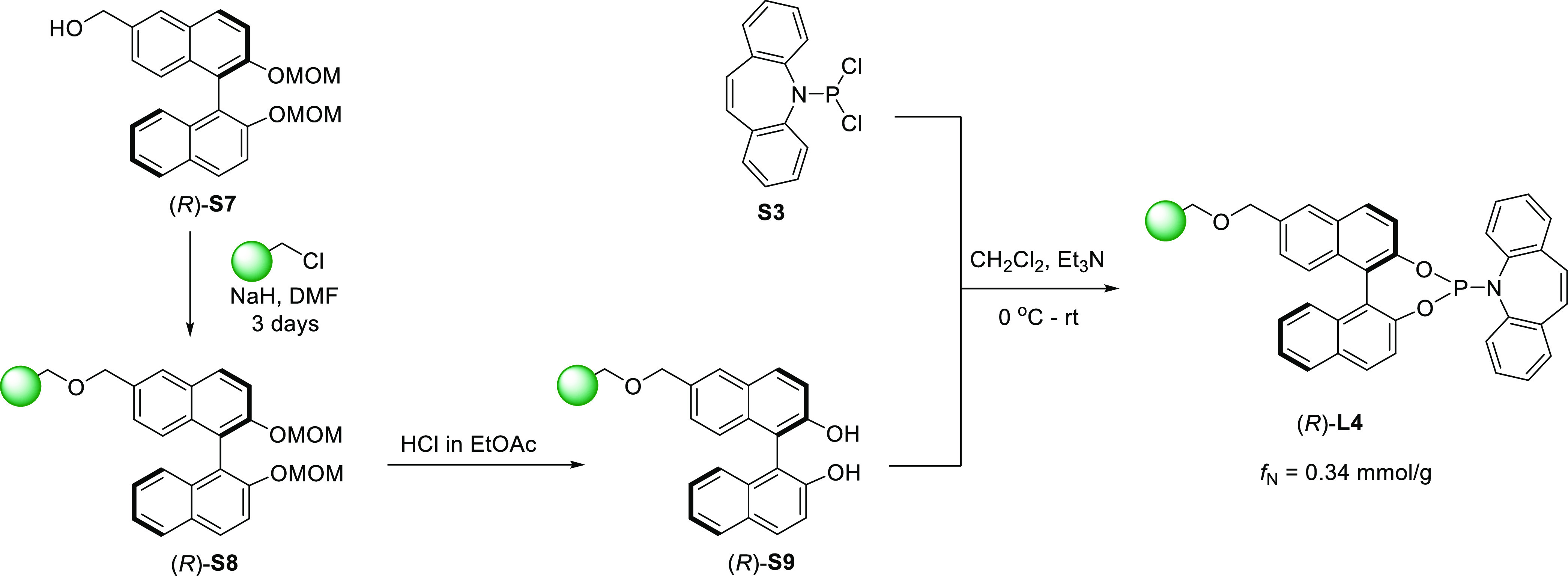
Preparation of the PS-Supported Ligand (*R*)-**L4**

While investigating the catalytic properties of the PS-supported (*R*)-**L1**, we questioned whether the triazole motif embedded in the structure as the linker would also coordinate the metal and whether this could exert a negative influence on the catalytic performance of the iridium complex by triggering a non-enantioselective reaction pathway. To evaluate this possibility, we resorted to use an alternative immobilization strategy for BINOL involving nucleophilic substitution of the Merrifield resin by a 6-hydroxymethyl substituted BINOL derivative. As shown in Scheme 3, we designed and synthesized (*R*)-**L4** according to his principle. The bis-MOM protected derivative (*R*)-**S7** (see the Supporting Information for full details)^13c^ was conveniently immobilized onto a Merrifield resin, and the (P, olefin) moiety was integrated in the usual manner to afford (*R*)-**L4** with a functionalization value of *f*_N_ = 0.34 mmol/g, appropriate for catalytic use.

A representative set of substrates, including those providing lower enantioselectivities with (*R*)-**L1,** was selected and subjected to the optimized reaction conditions for the allylic amination in the presence of (*R*)-**L4** (Table 3). With respect to catalytic activity, similar yields of the corresponding amine products were obtained compared with the results described in Table 2. On the other hand, notable improvements in enantioselectivity were recorded in most of the studied cases. Thus, substrates that were giving the corresponding amination products at relatively low ee with Ir-**L1**, such as **3f** and **3g**, now gave 89% ee with (*R*)-**L4**. Furthermore, even allylic alcohols that, with Ir-**L1**, already had superior performances, affording products with high/very high enantioselectivities, now presented even higher ee (**3a**, **3h–3j**, 92–99% ee). Using (*R*)-**L4**, an heteroaromatic allylic alcohol containing a thiophene motif (**1z**) could be converted to **3z** with a 68% yield and 87% ee.

**Table 3:**
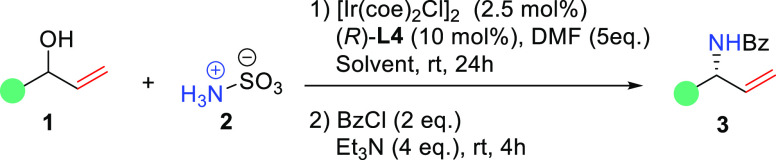

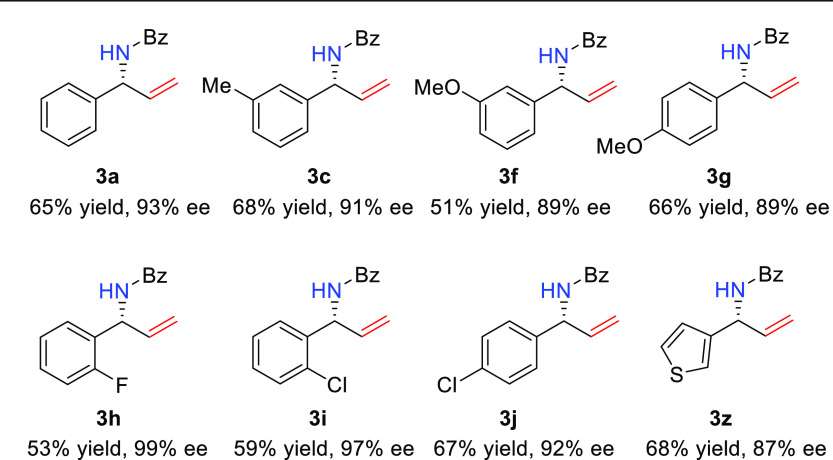
Scope of the Asymmetric Amination Reaction with an Immobilized Ligand (*R*)-**L4**^a^

a Reaction conditions: **1** (0.25 mmol), **2** (0.30 mmol), [Ir(coe)_2_Cl]_2_ (2.5 mol %), (*R*)-**L4** (10 mol %), DMF (5 equiv), rt, 2-MeTHF (1.2 mL).

Another important step in the evaluation of the sustainability characteristics associated with the use of **L1** and **L4** was to assess their recyclability. In this regard, we wanted to prove that the robust Ir–resin complex could allow not only the recycling of the ligand but also most of the expensive iridium metal. To assess this, we first performed a leaching test and analyzed the residue via iridium elemental analysis. The analysis of the crude indicated that, after the amination and washing steps, only 22% of iridium leached from the catalyst. This meant that, without adding additional iridium in following cycles, the recovered solid Ir–resin complex still kept 78% of its initial Ir loading and, probably, the corresponding catalytic potential. Encouraged by this finding, we employed a sample of **L1** and **L4** in several cycles. We used allylic alcohol (**1a**) with sulfamic acid (**2**) under the standard conditions (Table 4). At the end of each amination step, the reaction mixture was filtered and the solid Ir–resin complex washed with DCM (2 × 2 mL) under argon flow, then it was dried under vacuum and used in the next cycle, which started by simply using the recovered ligand and adding 0.5 mol % [Ir(coe)_2_Cl]_2_, according to the indications from the leaching test, instead of using freshly made ligand and 2.5 mol % [Ir(coe)_2_Cl]_2_. To our delight, ligands **L1** and **L4** showed high activity and excellent stereoselectivity in 10 consecutive cycles covering 10 days of uninterrupted operation, thus suggesting high robustness. Although catalytic activity slowly decreases during the operation period, enantioselectivity kept steadily around 90 to 93% ee. A combined weight of 0.27 and 0.29 g of pure **3a** were isolated after the 10 cycle operation, which suggested an accumulated TON of 65 and 70, respectively.

**Table 4:**
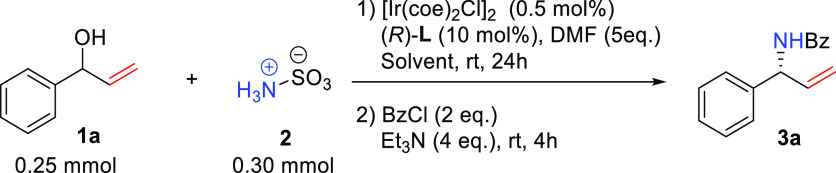
Recycling Experiments of the PS-Supported Ligands **L1** and **L4**^a^

cycle-**L1**	yield [%]	ee [%]	cycle-**L4**	yield [%]	ee [%]
1	63	91	1	65	93
2	59	90	2	61	91
3	52	90	3	56	91
4	48	90	4	51	91
5^b^	51	92	5	53	90
6^b^	46	92	6^b^	49	93
7^b^	45	91	7^b^	44	92
8^b^	38	92	8^b^	40	92
9^b^	32	92	9^b^	35	92
10^b^	25	92	10^b^	28	92

a Only in the first cycle, [Ir(coe)_2_Cl]_2_ (2.5 mol %) was used. All yields were isolated yields after amine protection.

b In this run, 1 equiv of **2** was added instead of 1.2 equiv.

**Scheme 4:**
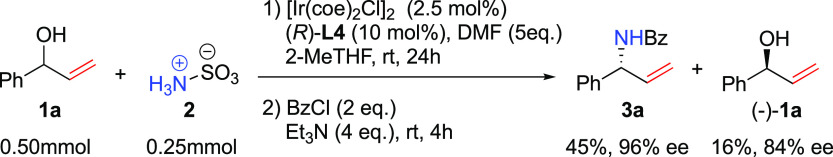
Kinetic Resolution Experiment of Allylic Alcohol **1a**

While testing the scope of the amination reaction with **L1** and **L4**, we noticed in some HPLC analyses the presence of very small amounts of the alcohol substrate in the enantiopure form, which suggested the operation of the dynamic kinetic resolution (DKR) mechanism.^15^ To confirm this, a kinetic resolution experiment of allylic alcohol **1a** was carried out using 0.5 equiv of **2** (Scheme 4). In this manner, a 16% yield of the alcohol **1a** was isolated with 84% enantiomeric purity, along with a 45% yield of **3a** (96% ee), confirming the existence of DKR in the process.

In summary, a family of PS-supported phosphoramidite, olefin ligands **L1**–**L4** has been developed. The optimal functional resins **L1** and **L4** can be easily prepared from cheap, commercially available starting materials. Key to the success of the synthetic strategy is the final coupling step with chlorophosphoramidite **S3**, allowing the rapid construction of a series of immobilized Carreira-type ligands from various readily available, immobilized axially chiral BINOL or SPINOL frameworks. The catalytic resin complexes generated by coordination of [Ir(coe)_2_Cl]_2_ with **L1** or **L4** exhibit excellent performance in catalytic asymmetric allylic amination of unmasked allylic alcohols. Important advantages of the heterogenized ligand systems over the referable homogeneous ones include robustness, resulting in high recyclability, convenient handling for sequential use in the preparation of libraries, and full preservation of the chemo- and enantioselectivity characteristics. Noteworthy, the whole catalytic system (iridium + immobilized P, olefin ligand) can be recycled, and repeated use of a single catalyst sample is possible through simple replenishment of leached iridium metal (ca. 20% per use).

Although additional work is still needed, this paves the way for performing Ir-(P, olefin) catalyzed reactions at 0.5 mol % of Ir loading or below. Moreover, the modularity of the synthetic approach for the preparation of the immobilized (P, olefin) ligands offers promise for the development of even more robust Ir-(P, olefin) complexes suitable for operation in continuous flow.

## Supporting Information Available

Synthetic procedures and complete spectroscopic (NMR and HPLC) characterizations of the catalytic structures and their precursors, synthetic procedures of immobilization of catalysts onto PS stationary phase, and complete characterization of all the products (NMR and HPLC) (PDF)

## Notes

Data Availability Statement: The data underlying this study are available in the published article and its online Supporting Information.
