# Supplementary material for: Development of Immobilized Carreira (Phosphoramidite, Olefin) Ligands and Application in Iridium-Catalyzed Asymmetric Allylic Amination
Source: J Org Chem. 2023 Jan 26;88(4):2166–73. doi: 10.1021/acs.joc.2c02589 (PMC9942233; doi:10.1021/acs.joc.2c02589)

*Supporting Information*

# Development of Immobilized Carreira (Phosphoramidite, Olefin) Ligands and Application in Iridium-Catalyzed Asymmetric Allylic Amination

Leijie Zhou, Nicola Zanda, Moreshwar Chaudhari, Mariane Felicio Da Silva and Miquel A. Pericàs*

miquelangel.pericas@urv.cat

Institute of Chemical Research of Catalonia (ICIQ), The Barcelona Institute of Science and Technology, Av. Països Catalans, 16, 43007 Tarragona, Spain
Departament de Química Física i Inorgànica, Universitat Rovira i Virgili, 43007 Tarragona, Spain

Table of Contents

1. General information S2

2. Preparation of the immobilized ligand (*R*)-**L1** S3

3. Preparation of the immobilized ligand (*R*)-**L2** S4

4. Preparation of the immobilized ligand (*R*)-**L3** S5

5. Preparation of the immobilized ligand (*R*)-**L4** S6

6. Synthesis and characterization of allylic alcohols **1** S8

7. Preparation of products **3**-**4** S16

8. Characterization data of products **3**-**4** S17

9. Direct reuse of the whole catalytic system S31

10. General procedure for the recycle experiment S31

11. References S32

12. NMR spectra S35

13. HPLC chromatograms S88

# 1. General information

Unless otherwise stated, all reactions were conducted under air. All commercial reagents were used as received. Flash chromatography was carried out using 60 mesh silica gel and dry-packed columns. The continuous flow experiments were carried out using a syringe pump (Legato 200 from KDSCIENTIFIC). The packed-bed reactor consisted in an Omnifit glass chromatography column (10 mm bore size and up to maximal 70 mm of adjustable bed height). Thin layer chromatography was carried out using Merck TLC Silicagel 60 F254 aluminum sheets. Components were visualized by UV light (λ = 254 nm) and stained with phosphomolybdic dip. NMR spectra were recorded at 298 K on a Fourier 300 MHz Bruker, a Bruker Avance 400 Ultrashield or a Bruker Avance 500 Ultrashield apparatus. $^1$H NMR spectroscopy chemical shifts are quoted in ppm relative to tetramethylsilane (TMS). $CDCl_3$ was used as internal standard for $^{13}$C NMR spectra. Chemical shifts are given in δ and coupling constants in Hz. IR spectra were recorded on a Bruker Tensor 27 FT-IR spectrometer and are reported in wavenumbers ($cm^{-1}$). Elemental analyses were performed by MEDAC Ltd. (Surrey, UK) on a LECO CHNS 932 micro-analyzer. High performance liquid chromatography (HPLC) was performed on Agilent Technologies chromatographs (1100 and 1200 Series), using Chiralpak IC, AD-H, OJ-H columns and guard columns. High-resolution mass spectra (HRMS) were obtained from the ICIQ High Resolution Mass Spectrometry Unit on MicroTOF Focus and Maxis Impact (Bruker Daltonics) with electrospray ionization. Specific optical rotation measurements were carried out on a Jasco P-1030 polarimeter.

# Experimental Section

## 2. Preparation of the immobilized ligand (*R*)-L1

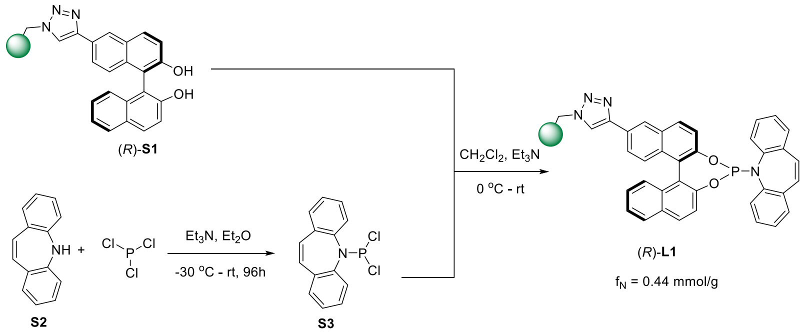


**Polystyrene Supported BINOL S1**

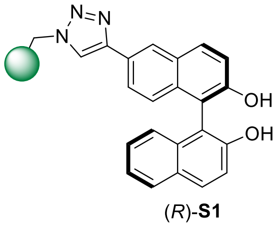


PS supported BINOL **S1** was synthesized following a same procedure previously described by our group in a same scale, by using of a commercial available Merrifiled resin (1% DVB, 100 - 200 mesh, Novabiochem, f = 0.60 mmol/g).[1] Around 6.62 g of the PS supported BINOL (**S1**) was obtained as white solid. The functionalization was calculated on the basis of nitrogen elemental analysis.

EA found (%): C 88.02, H 7.11, N 2.28, O 1.69;

$f_{(N)}$= 0.54 mmol/g resin

**5-(dichlorophosphaneyl)-5*H*-dibenzo[*b*,*f*]azepine S3**

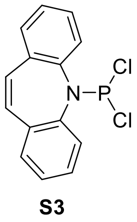


**S3** was synthesized following a modification of a literature procedure.[2] Under argon atmosphere, iminostilbene (5.79 g, 30 mmol) was charged into an oven dried 500 mL round-bottom flask. Dry $Et_2O$ (200 mL) was then added, and the solution was cooled to -30 °C, followed by $Et_3N$ (12.12 g, 4eq, 120 mmol), and cold $PCl_3$ (8.16 g, 2eq, 60 mmol). The resulting bright-orange mixture was then allowed to warm to room temperature and stirred for 96h, during which time the mixture gradually became pale yellow and large amounts of a white solid precipitated. Evaporation of the volatiles under vacuum gave a pale-yellow powder that was extracted with toluene (3 × 20 mL, filtered under argon atmosphere). The combined clear yellow extracts were then kept at fridge overnight to complete crystallization. The precipitate was separated by filtration and dried in vacuum to give **S3** (6.2 g, 70%) as a pale yellow solid. The characterization of the title compound was consistent with the data available in the literature.

**$^{1}H$ NMR** (400 MHz, $CDCl_3$): δ 7.64 – 7.59 (m, 2H), 7.50 – 7.42 (m, 2H), 7.38 – 7.34 (m, 4H), 6.89 (s, 2H).

**$^{13}C$ {1H} NMR** (101 MHz, $CDCl_3$): δ 141.6, 141.5, 135.7, 135.6, 130.9, 129.5, 129.4, 127.9, 127.8, 127.3, 127.2.

**Polystyrene Supported ligand (*R*)-L1**

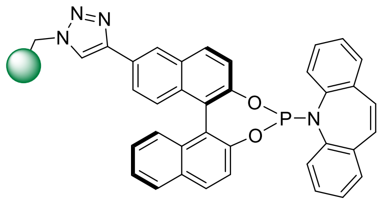

(*R*)-**L1**

(*R*)-**L1** was synthesized following a modification of a literature procedure.[2] Under argon atmosphere, PS supported BINOL **S3** (3.00 g, 1.62 mmol) was charged into an oven dried 100 mL round-bottom flask, followed by addition of dry $CH_2Cl_2$ (20 mL) and shake at room temperature for 1 h to swallow the resin completely. In another 50 mL flask, charged with **S5** (0.95 g, 2eq, 3.24 mmol), dry $CH_2Cl_2$ (15 mL) was added first, followed by $Et_3N$ (0.66 g, 4eq, 6.48 mmol), which afforded a very pale-yellow clear solution. This stirred solution was then cooled and kept at 0 °C. After 5 mins, it was transferred into the PS supported Binol solution described above carefully. The resulting mixture was allowed to shake at room temperature for 48 h. The solution was then filtered and washed with dry $CH_2CH_2$ (4 × 150 mL) quickly under argon flow atmosphere. After dried under high vacuum overnight, 3.3 g (*R*)-**L1** was obtained as pale yellow solid polymer. The functionalization was calculated on the basis of nitrogen elemental analysis.

EA found (%): C 90.51, H 6.88, N 2.48, P 1.25;

$f_{(N)}$= 0.44 mmol/g resin

## 3. Preparation of the immobilized ligand (*R*)-L2

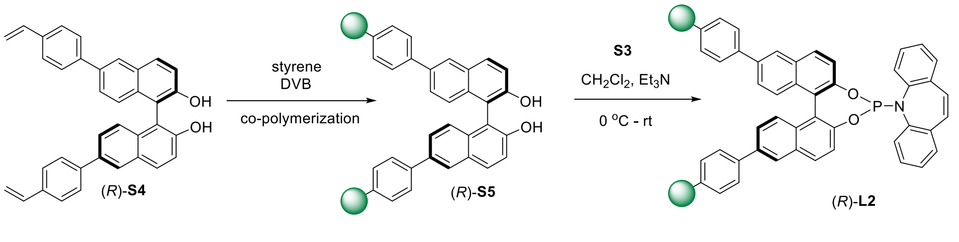

**Polystyrene Supported BINOL (*R*)-S5**

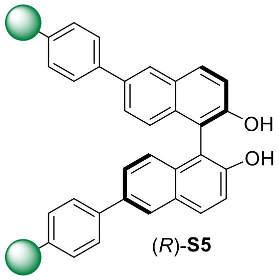

(*R*)-**S5**

**S5** was synthesized following a following a modification of a literature procedure.[3] A 100 mL reactor was charged with a suspension of polyvinyl alcohol (PV-OH) (100 mg, 0.96 µmol, 0.001 equiv.) in 72 mL of degassed MiliQ water. The solution was heated at 100 °C in an oil bath until PV-OH was dissolved. Then, it was cooled to RT and a solution of boric acid (449 mg, 7.26 mmol) in 18 mL of degassed MiliQ water was transferred to the reactor. Later, a degassed solution containing divinylbenzene (DVB), filtered on a short pad of silica immediately before use, (80%, 119 µL, 0.68 mmol, 0.65 equiv.), BINOL derivative **S4** (490 mg, 1.00 mmol, 1eq.), styrene (2.9 mL, 25.5 mmol, 23.66 equiv.) and AIBN (31 mg, 0.19 mmol, 0.18 equiv.) in toluene (2.4 mL) was transferred to the reactor. After that, the system was heated at 90 °C in an oil bath and magnetically stirred at 440 rpm overnight, the aqueous solution was decanted off and the resin was washed with water (50 °C) several times, followed by MeOH and $CH_2Cl_2$. Finally, it was dried overnight in a 40 °C vacuum oven to furnish 2.3 g of light yellow beads for direct use in next step.

**Polystyrene Supported ligand (*R*)-L2**

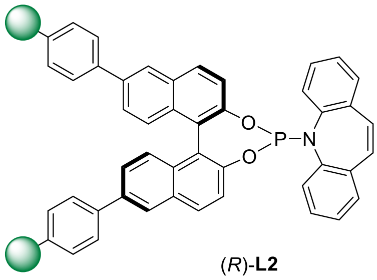

(*R*)-**L2**

(*R*)-**L2** was synthesized following a modification of a literature procedure.[2] Under argon atmosphere, PS supported BINOL **S5** (2.30 g, ca. 1.00 mmol) was charged into an oven dried 100 mL round-bottom flask, followed by addition of dry $CH_2Cl_2$ (20 mL) and shake at room temperature for 1 h to swallow the resin completely. In another 50 mL flask, charged with **S3** (2eq, 2.00 mmol), dry $CH_2Cl_2$ (15 mL) was added first, followed by $Et_3N$ (4eq, 4.00 mmol), which afforded a very pale-yellow clear solution. This stirred solution was then cooled and kept at 0 °C. After 5 mins, it was transferred into the PS supported Binol solution described above carefully. The resulting mixture was allowed to shake at room temperature for 48 h. The solution was then filtered and washed with dry $CH_2CH_2$ (4 × 150 mL) quickly under argon flow atmosphere. After dried under high vacuum overnight, 2.65 g (*R*)-**L2** was obtained as light yellow solid polymer. The functionalization was calculated on the basis of nitrogen elemental analysis.

EA found (%): C 85.18, H 8.13, N 0.30, P 1.05;

$f_{(N)}$= 0.21 mmol/g resin

## 4. Preparation of the immobilized ligand (*R*)-L3

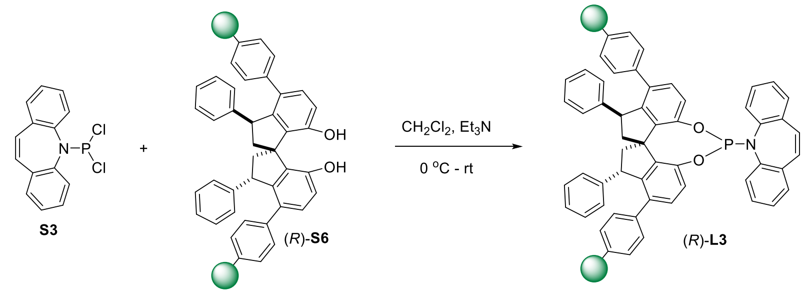


(*R*)-**L3** was synthesized following a modification of a literature procedure.[2] Under argon atmosphere, a previously developed immobilized SPINOL **S6**[3] (0. 27 g, ca. 0.100 mmol) was charged into an oven dried 25 mL round-bottom flask, followed by addition of dry $CH_2Cl_2$ (5 mL) and shake at room temperature for 1 h to swallow the resin completely. In another 10 mL flask, charged with **S3** (2eq, 0.2 mmol), dry $CH_2Cl_2$ (2 mL) was added first, followed by $Et_3N$ (4eq, 0.4 mmol), which afforded a very pale-yellow clear solution. This stirred solution was then cooled and kept at 0 °C. After 5 mins, it was transferred into the PS supported SPINOL solution described above carefully. The resulting mixture was allowed to shake at room temperature for 48 h. The solution was then filtered and washed with dry $CH_2CH_2$ (4 × 20 mL) quickly under argon flow atmosphere. After dried under high vacuum overnight, 0.30 g (*R*)-**L3** was obtained as light yellow solid polymer. The functionalization was calculated on the basis of nitrogen elemental analysis.

EA found (%): C 86.44, H 6.90, N 0.46, P 0.65;

$f_{(N)}$= 0.33 mmol/g resin

## 5. Preparation of the immobilized ligand (*R*)-L4

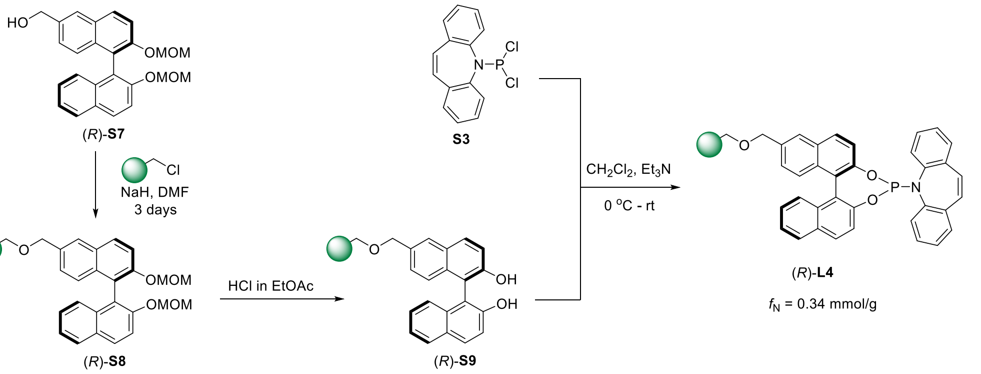

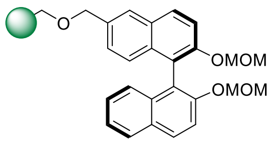


(*R*)-**S8**

**S8** was prepared according to a reported procedure,[4] to a suspension of NaH (0.165 g, 1.15 mmol) in dry DMF (16 mL) was added (2,2'-bis(methoxymethoxy)-[1,1'-binaphthalen]-6-yl)methanol[5] (0.41g, 1 mmol, dissolved in 10 mL of DMF) at rt, under Ar atmosphere. In a second flask, the Merrifield resin (1% DVB, 1.83 g, 1.10 mmol of active chlorine) and $Bu_4NI$ (0.050 g, 0.14 mmol) were suspended in dry DMF (13 mL) in order to swell the resin. Both suspensions were shaken for 1 h and the first one was then added via cannula to the resin. The reaction mixture was shaken at room temperature for 72h. The resulting resin was filtered and washed successively with water (200 mL), water/THF 1:1 (200 mL), THF (200 mL), THF/$CH_2Cl_2$ 1:1 (200 mL) and $CH_2Cl_2$ (200 mL). Then it was allowed to dry overnight under vacuum at 50 °C and 2.20 g of **S8** were isolated.

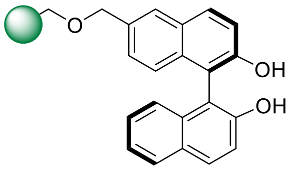


(*R*)-**S9**

A mixture of resin **S8** (2.20 g, ca. 1.00 mmol) and HCl in EtOAc (2 M, 23 mL, 46 mmol) was shaken at rt overnight. After filtration, the resin was washed successively with EtOAc (200 mL), EtOAc/$CH_2Cl_2$ (200 mL) and $CH_2Cl_2$ (200 mL) and dried overnight under vacuum at 50 °C to afford **S9** (2.05 g).

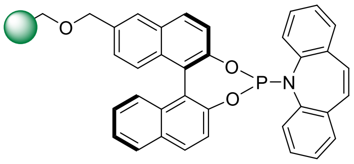


(*R*)-**L4**

(*R*)-**L4** was synthesized following a modification of a literature procedure.[2] Under argon atmosphere, immobilized BINOL **S9** (2.05 g, ca. 1.00 mmol) was charged into an oven dried 100 mL round-bottom flask, followed by addition of dry $CH_2Cl_2$ (20 mL) and shake at room temperature for 1 h to swallow the resin completely. In another 50 mL flask, charged with **S3** (0.59 g, 2eq, 2.00 mmol), dry $CH_2Cl_2$ (10 mL) was added first, followed by $Et_3N$ (0.41 g, 4eq, 4 mmol), which afforded a very pale-yellow clear solution. This stirred solution was then cooled and kept at 0 °C. After 5 mins, it was transferred into the PS supported BINOL suspension described above carefully. The resulting mixture was allowed to shake at room temperature for 48 h. The solution was then filtered and washed with dry $CH_2CH_2$ (4 × 150 mL) quickly under argon flow atmosphere. After dried under high vacuum overnight, 2.50 g (*R*)-**L1** was obtained as pale yellow solid polymer. The functionalization was calculated on the basis of nitrogen elemental analysis.

EA found (%): C 88.18, H 7.04, N 0.48, P 0.99;

$f_{(N)}$= 0.34 mmol/g resin

## **6.** Synthesis and Characterization of Allylic Alcohols **1**

### ***GP1 - General Procedure for the Synthesis of Allylic Alcohols***

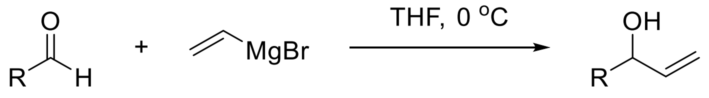


Under argon atmosphere and at 0 °C, to a solution of aldehyde (1 equiv.) in anhydrous THF (0.2 M), a 1 M solution of vinylmagnesium bromide in THF (1.50 equiv.) was added carefully. Then was it was allowed to warm to room temperature and stirred overnight. The reaction was quenched carefully by saturated aq. $NH_4Cl$, and extracted with EtOAc. The combined organic fractions were washed with brine, dried over $MgSO_4$. Purification of the crude by flash column chromatography on silica gel using EtOAc/cyclohexane (1:2 to 1:15) as solvent provided the alcohol products **1a**-**1z**.

**1-phenylprop-2-en-1-ol**

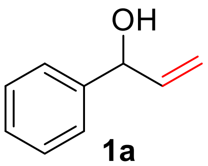


**1a**

**1a** was prepared according to **GP1**, obtained as colorless oil (1.14 g, 85% yield), the characterization of the title compound was consistent with the data available in the literature.[6]

**$^1$H NMR** (500 MHz, $CDCl_3$): δ 7.53 – 7.30 (m, 5H), 6.08 (ddd, J = 16.9, 10.2, 6.1 Hz, 1H), 5.38 (dt, J = 17.1, 1.4 Hz, 1H), 5.31 – 5.11 (m, 2H), 2.48 – 1.91 (m, 1H).

**$^{13}$C {1H} NMR** (126 MHz, $CDCl_3$): δ 142.6, 140.3, 128.6, 127.7, 126.3, 115.1, 75.3.

**1-(*o*-tolyl)prop-2-en-1-ol**

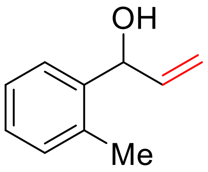


**1b**

**1b** was prepared according to **GP1**, obtained as colorless oil (1.18 g, 80% yield), the characterization of the title compound was consistent with the data available in the literature.[7]

**$^1$H NMR** (400 MHz, $CDCl_3$): δ 7.48 (dd, J = 7.3, 1.9 Hz, 1H), 7.30 – 7.16 (m, 3H), 6.06 (ddd, J = 17.1, 10.4, 5.7 Hz, 1H), 5.42 (dd, J = 5.5, 3.9 Hz, 1H), 5.34 (dt, J = 17.2, 1.5 Hz, 1H), 5.24 (dt, J = 10.4, 1.4 Hz, 1H), 2.39 (s, 3H), 2.23 (dd, J = 4.1, 1.8 Hz, 1H).

**$^{13}$C {1H} NMR** (101 MHz, $CDCl_3$): δ 140.5, 139.4, 135.3, 130.5, 127.6, 126.3, 125.9, 115.1, 72.0, 19.1.

**1-(*m*-tolyl)prop-2-en-1-ol**

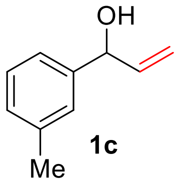

1c

**1c** was prepared according to **GP1**, obtained as colorless oil (1.27 g, 86% yield), the characterization of the title compound was consistent with the data available in the literature.[8]

**$^{1}$H NMR** (400 MHz, $CDCl_3$): δ 7.43 – 7.03 (m, 4H), 6.08 (ddd, J = 17.1, 10.3, 6.0 Hz, 1H), 5.39 (dt, J = 17.1, 1.5 Hz, 1H), 5.30 – 5.15 (m, 2H), 2.39 (d, J = 0.8 Hz, 3H), 2.03 (d, J = 3.2 Hz, 1H).

**$^{13}$C {1H} NMR** (101 MHz, $CDCl_3$): δ 142.5, 140.3, 138.2, 128.5(x2), 127.0, 123.4, 114.9, 75.3, 21.4.

**1-(*p*-tolyl)prop-2-en-1-ol**

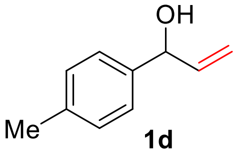

1d

**1d** was prepared according to **GP1**, obtained as colorless oil (1.08 g, 73% yield),the characterization of the title compound was consistent with the data available in the literature.[9]

**$^{1}$H NMR** (400 MHz, $CDCl_3$): δ 7.37 – 7.24 (m, 2H), 7.20 (dt, J = 7.8, 0.7 Hz, 2H), 6.08 (ddd, J = 17.1, 10.3, 5.9 Hz, 1H), 5.37 (dt, J = 17.1, 1.5 Hz, 1H), 5.28 – 5.02 (m, 2H), 2.39 (s, 3H), 2.10 (s, 1H).

**$^{13}$C {1H} NMR** (101 MHz, $CDCl_3$): δ 140.4, 139.7, 137.4, 129.2, 126.3, 114.8, 75.1, 21.1.

**1-(2-methoxyphenyl)prop-2-en-1-ol**

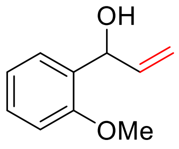

1e

**1e** was prepared according to **GP1**, obtained as light-yellow oil (1.18 g, 72% yield), the characterization of the title compound was consistent with the data available in the literature.[10]

**$^{1}$H NMR** (400 MHz, $CDCl_3$): δ 7.47 – 7.21 (m, 2H), 7.13 – 6.79 (m, 2H), 6.16 (ddd, J = 17.2, 10.4, 5.5 Hz, 1H), 5.45 (d, J = 5.9 Hz, 1H), 5.35 (dt, J = 17.2, 1.5 Hz, 1H), 5.20 (dt, J = 10.4, 1.5 Hz, 1H), 3.88 (d, J = 1.9 Hz, 3H), 2.98 (s, 1H).

**$^{13}$C {1H} NMR** (101 MHz, $CDCl_3$): 156.7, 139.5, 130.86, 130.83, 128.7, 127.4, 120.9, 114.4, 110.7, 71.4, 55.4.

**1-(3-methoxyphenyl)prop-2-en-1-ol**

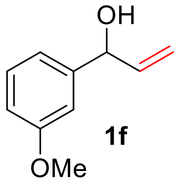

1f

**1f** was prepared according to **GP1**, obtained as light-yellow oil (1.14 g, 70% yield), the characterization of the title compound was consistent with the data available in the literature.[8]

**$^1$H NMR** (400 MHz, $CDCl_3$): δ 7.41 – 7.24 (m, 1H), 7.14 – 6.93 (m, 2H), 6.85 (ddd, J = 8.3, 2.5, 1.2 Hz, 1H), 6.06 (ddd, J = 17.2, 10.3, 6.0 Hz, 1H), 5.37 (dt, J = 17.1, 1.5 Hz, 1H), 5.29 – 5.10 (m, 2H), 3.83 (s, 3H).

**$^{13}$C {1H} NMR** (101 MHz, $CDCl_3$): 159.8, 144.3, 140.1, 129.5, 118.6, 115.1, 113.3, 111.7, 75.2, 55.2.

**1-(4-methoxyphenyl)prop-2-en-1-ol**

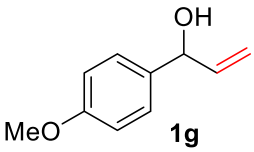


**1g**

**1g** was prepared according to **GP1**, obtained as light-yellow oil (1.06 g, 65% yield), the characterization of the title compound was consistent with the data available in the literature.[11]

**$^1$H NMR** (400 MHz, $CDCl_3$): δ 7.58 – 7.25 (m, 2H), 7.05 – 6.80 (m, 2H), 6.06 (ddd, J = 17.2, 10.3, 5.8 Hz, 1H), 5.35 (dt, J = 17.1, 1.5 Hz, 1H), 5.30 – 5.07 (m, 2H), 3.82 (s, 3H), 2.23 (s, 1H).

**$^{13}$C {1H} NMR** (101 MHz, $CDCl_3$): 159.2, 140.4, 134.9, 127.7, 114.7, 113.9, 74.8, 55.3.

**1-(2-fluorophenyl)prop-2-en-1-ol**

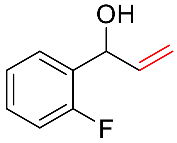


**1h**

**1h** was prepared according to **GP1**, obtained as light-yellow oil (1.19 g, 75% yield), the characterization of the title compound was consistent with the data available in the literature.[12]

**$^1$H NMR** (400 MHz, $CDCl_3$): δ 7.47 (td, J = 7.5, 1.9 Hz, 1H), 7.40 – 6.96 (m, 3H), 6.09 (dddd, J = 16.9, 10.4, 5.6, 0.8 Hz, 1H), 5.54 (td, J = 5.3, 4.8, 2.9 Hz, 1H), 5.45 – 5.28 (m, 1H), 5.23 (dt, J = 10.4, 1.4 Hz, 1H), 2.30 (d, J = 4.4 Hz, 1H).

**$^{13}$C {1H} NMR** (101 MHz, $CDCl_3$): δ 160.0 (d, J = 246.3 Hz), 138.9, 129.7 (d, J = 13.2 Hz), 129.2 (d, J = 8.3 Hz), 127.6 (d, J = 4.2 Hz), 124.3 (d, J = 3.5 Hz), 115.4 (d, J = 21.7 Hz), 115.3, 69.2 (d, J = 3.2 Hz).

**$^{19}$F NMR** (376 MHz, $CDCl_3$): δ -119.3 (s, 1F).

**1-(2-chlorophenyl)prop-2-en-1-ol**

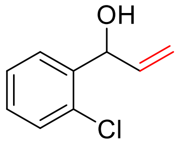


**1i**

**1i** was prepared according to **GP1**, obtained as light-yellow oil (1.19 g, 71% yield), the characterization of the title compound was consistent with the data available in the literature.[11]

**$^1$H NMR** (400 MHz, $CDCl_3$): δ 7.56 (dd, J = 7.7, 1.8 Hz, 1H), 7.44 – 7.16 (m, 3H), 6.06 (dddd, J = 16.5, 10.4, 5.5, 0.6 Hz, 1H), 5.66 (tt, J = 3.4, 1.7 Hz, 1H), 5.41 (dt, J = 17.2, 1.4 Hz, 1H), 5.25 (dt, J = 10.4, 1.4 Hz, 1H), 2.25 (d, J = 3.9 Hz, 1H)..

**$^{13}$C {1H} NMR** (101 MHz, $CDCl_3$): 139.9, 138.3, 132.4, 129.5, 128.8, 127.6, 127.2, 115.6, 71.4.

**1-(4-chlorophenyl)prop-2-en-1-ol**

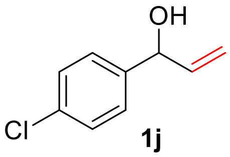


**1j**

**1j** was prepared according to **GP1**, obtained as light-yellow oil (1.33 g, 79% yield), the characterization of the title compound was consistent with the data available in the literature.[11]

**$^1$H NMR** (400 MHz, $CDCl_3$): δ 7.51 – 7.20 (m, 4H), 5.99 (ddd, J = 17.1, 10.3, 6.1 Hz, 1H), 5.33 (dt, J = 17.1, 1.4 Hz, 1H), 5.28 – 5.07 (m, 2H), 2.60 (d, J = 3.2 Hz, 1H).

**$^{13}$C {1H} NMR** (101 MHz, $CDCl_3$): 141.0, 139.8, 133.4, 128.6, 127.7, 115.6, 74.6.

**1-(4-bromophenyl)prop-2-en-1-ol**

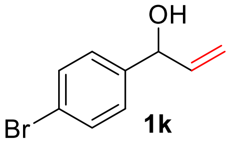


**1k**

**1k** was prepared according to **GP1**, obtained as light-yellow oil (1.38 g, 65% yield), the characterization of the title compound was consistent with the data available in the literature.[13]

**$^1$H NMR** (400 MHz, $CDCl_3$): δ 7.63 – 7.38 (m, 2H), 7.32 – 7.13 (m, 2H), 6.00 (ddd, J = 17.1, 10.3, 6.1 Hz, 1H), 5.40 – 5.03 (m, 3H), 2.41 (d, J = 2.8 Hz, 1H).

**$^{13}$C {1H} NMR** (101 MHz, $CDCl_3$): 141.5, 139.8, 131.6, 128.0, 121.5, 115.7, 74.7.

**1-(4-iodophenyl)prop-2-en-1-ol**

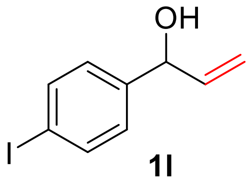


**1l**

**1l** was prepared according to **GP1**, obtained as white solid (1.64 g, 63% yield), the title compound is known (CAS:1418147-91-7), although its characterization data have not been reported.

**$^1$H NMR** (400 MHz, $CDCl_3$): δ 7.83 – 7.57 (m, 2H), 7.21 – 7.04 (m, 2H), 6.01 (ddd, J = 17.1, 10.3, 6.1 Hz, 1H), 5.36 (dt, J = 17.1, 1.3 Hz, 1H), 5.30 – 5.01 (m, 2H), 2.07 (d, J = 3.1 Hz, 1H).

**$^{13}$C {1H} NMR** (101 MHz, $CDCl_3$): 142.2, 139.8, 137.6, 128.3, 115.7, 93.2, 74.8.

**1-(4-nitrophenyl)prop-2-en-1-ol**

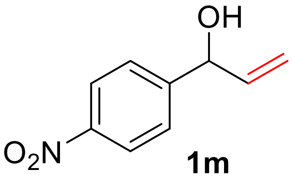


**1m**

**1m** was prepared according to **GP1**, obtained as light-yellow oil (1.09 g, 61% yield), the characterization of the title compound was consistent with the data available in the literature.[14]

**$^1$H NMR** (400 MHz, $CDCl_3$): δ 8.36 – 8.13 (m, 2H), 7.73 – 7.43 (m, 2H), 6.01 (ddd, J = 16.9, 10.2, 6.5 Hz, 1H), 5.49 – 5.21 (m, 3H), 2.19 (s, 1H).

**$^{13}$C {1H} NMR** (101 MHz, $CDCl_3$): 149.5, 147.3, 139.1, 126.9, 123.7, 116.8, 74.5.

**1-(4-(trifluoromethyl)phenyl)prop-2-en-1-ol**

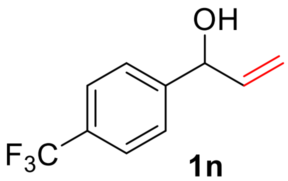


**1n**

**1n** was prepared according to **GP1**, obtained as light-yellow oil (1.19 g, 59% yield), the characterization of the title compound was consistent with the data available in the literature.[7]

**$^1$H NMR** (400 MHz, $CDCl_3$): δ 7.68 – 7.55 (m, 2H), 7.55 – 7.45 (m, 2H), 6.02 (ddd, J = 16.8, 10.1, 6.3 Hz, 1H), 5.45 – 5.32 (m, 1H), 5.32 – 5.17 (m, 2H), 2.42 (d, J = 3.5 Hz, 1H).

**$^{13}$C {1H} NMR** (101 MHz, $CDCl_3$): 146.3 (d, J = 1.8 Hz), 139.6, 129.8 (q, J = 32.3 Hz), 126.5, 125.4 (q, J = 3.7 Hz), 124.1 (q, J = 272.7 Hz), 116.1, 74.8.

**$^{19}$F NMR** (376 MHz, $CDCl_3$) δ -62.6 (s, 3F).

**methyl 4-(1-hydroxyallyl)benzoate**

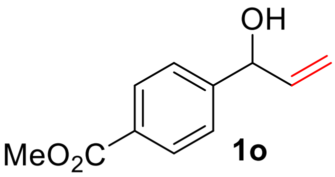


**1o**

**1o** was prepared according to **GP1**, obtained as light-yellow oil (1.01 g, 53% yield), the characterization of the title compound was consistent with the data available in the literature.[15]

**$^1$H NMR** (400 MHz, $CDCl_3$): δ 8.08 – 7.94 (m, 2H), 7.57 – 7.39 (m, 2H), 6.16 – 5.91 (m, 1H), 5.37 (dt, J = 17.1, 1.3 Hz, 1H), 5.33 – 5.13 (m, 2H), 3.92 (d, J = 0.6 Hz, 3H), 2.40 (d, J = 3.4 Hz, 1H).

**$^{13}$C {1H} NMR** (101 MHz, $CDCl_3$): 166.9, 147.6, 139.7, 129.8, 129.3, 126.1, 115.9, 74.9, 52.1.

**1-(4-((trimethylsilyl)ethynyl)phenyl)prop-2-en-1-ol**

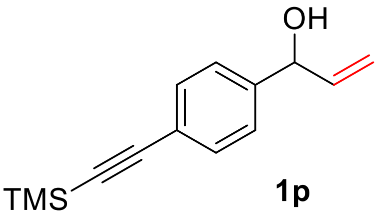


**1p**

**1p** was prepared according to **GP1**, obtained as light-yellow oil (1.38 g, 60% yield), the characterization of the title compound was consistent with the data available in the literature.[16]

**$^1$H NMR** (400 MHz, $CDCl_3$): δ 7.47 (d, J = 8.4 Hz, 2H), 7.41 – 7.20 (m, 3H), 6.01 (ddd, J = 17.2, 10.3, 6.1 Hz, 1H), 5.34 (dt, J = 17.1, 1.4 Hz, 1H), 5.26 – 5.10 (m, 2H), 2.15 (d, J = 4.1 Hz, 1H), 0.28 (s, 9H).

**$^{13}$C {1H} NMR** (101 MHz, $CDCl_3$): 142.8, 139.8, 132.1, 126.1, 122.4, 115.6, 104.9, 94.2, 75.0, 0.0.

**1-(4-(*tert*-butyl)phenyl)prop-2-en-1-ol**

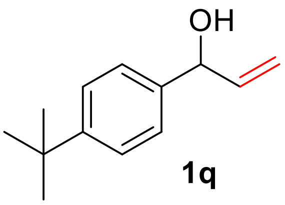

1q

**1q** was prepared according to **GP1**, obtained as colorless oil (1.24 g, 65% yield), the characterization of the title compound was consistent with the data available in the literature.[17]

**$^1$H NMR** (400 MHz, $CDCl_3$): δ 7.53 – 7.40 (m, 2H), 7.40 – 7.30 (m, 2H), 6.10 (ddd, J = 17.2, 10.3, 6.0 Hz, 1H), 5.39 (dt, J = 17.1, 1.5 Hz, 1H), 5.29 – 5.10 (m, 2H), 2.43 – 2.22 (m, 1H), 1.38 (s, 9H).

**$^{13}$C {1H} NMR** (101 MHz, $CDCl_3$): 150.7, 140.3, 139.7, 126.1, 125.5, 114.8, 75.1, 34.5, 31.4.

**1-(benzo[*d*][1,3]dioxol-5-yl)prop-2-en-1-ol**

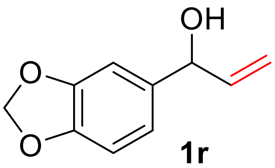

1r

**1r** was prepared according to **GP1**, obtained as light-yellow oil (1.14 g, 64% yield), the characterization of the title compound was consistent with the data available in the literature.[18]

**$^1$H NMR** (400 MHz, $CDCl_3$): δ 6.92 – 6.71 (m, 3H), 6.01 (ddd, J = 17.1, 10.3, 5.9 Hz, 1H), 5.94 (s, 2H), 5.33 (dt, J = 17.1, 1.4 Hz, 1H), 5.19 (dt, J = 10.3, 1.4 Hz, 1H), 5.10 (d, J = 5.8 Hz, 1H), 2.36 (s, 1H).

**$^{13}$C {1H} NMR** (101 MHz, $CDCl_3$): 147.8, 147.0, 140.2, 136.7, 119.8, 114.9, 108.1, 107.0, 101.0, 75.0.

**1-(phenanthren-9-yl)prop-2-en-1-ol**

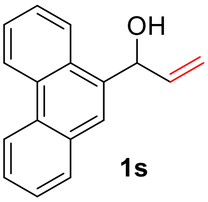

1s

**1s** was prepared according to **GP1**, obtained as white solid (2.01 g, 86% yield), the title compound was previously unknown.

**MP** = 98-100 °C

**$^1$H NMR** (500 MHz, $CDCl_3$): δ 8.94 – 8.59 (m, 2H), 8.24 (dd, J = 8.1, 1.5 Hz, 1H), 7.98 – 7.81 (m, 2H), 7.79 – 7.53 (m, 4H), 6.33 (ddd, J = 17.3, 10.4, 5.3 Hz, 1H), 5.93 (t, J = 4.4 Hz, 1H), 5.52 (dt, J = 17.3, 1.5 Hz, 1H), 5.35 (dt, J = 10.4, 1.4 Hz, 1H), 2.32 (d, J = 3.8 Hz, 1H).

**$^{13}$C {1H} NMR** (126 MHz, $CDCl_3$): δ 139.4, 136.3, 131.4, 130.9, 130.3, 129.7, 128.9, 126.9, 126.8, 126.6, 126.4, 124.9, 124.6, 123.2, 122.5, 116.2, 72.4.

**IR** (ATR): ν 3307, 1449, 1060, 927, 726, 423 $cm^{-1}$.

**HRMS** (ESI) m/z: $[M+H]^+$ Calcd for $C_{17}H_{15}O^+$ 235.1117; Found 235.1110.

***tert*-butyl 3-(1-hydroxyallyl)-1*H*-indole-1-carboxylate**

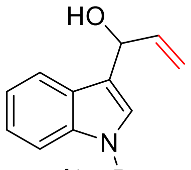

**1t** Boc

**1t** was prepared according to **GP1**, obtained as light-yellow oil (1.50 g, 55% yield), the characterization of the title compound was consistent with the data available in the literature.[19]

**$^{1}$H NMR** (400 MHz, $CDCl_3$): δ 8.17 (d, J = 8.3 Hz, 1H), 7.70 (ddd, J = 7.8, 1.3, 0.7 Hz, 1H), 7.58 (s, 1H), 7.35 (ddd, J = 8.4, 7.2, 1.3 Hz, 1H), 7.31 – 7.18 (m, 1H), 6.23 (ddd, J = 17.4, 10.3, 5.6 Hz, 1H), 5.66 – 5.43 (m, 2H), 5.36 – 5.26 (m, 1H), 2.03 (d, J = 4.2 Hz, 1H), 1.69 (s, 9H).

**$^{13}$C {1H} NMR** (101 MHz, $CDCl_3$): 149.7, 138.9, 135.9, 128.5, 124.6, 123.0, 122.6, 122.2, 119.9, 115.9, 115.3, 83.8, 68.9, 28.2.

**(*E*)-1-phenylpenta-1,4-dien-3-ol**

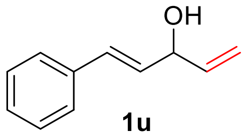

**1u**

**1u** was prepared according to **GP1**, obtained as light-yellow oil (0.83 g, 52% yield), the characterization of the title compound was consistent with the data available in the literature.[20]

**$^{1}$H NMR** (400 MHz, $CDCl_3$): δ 7.44 – 7.40 (m, 2H), 7.37 – 7.32 (m, 2H), 7.30 – 7.26 (m, 1H), 6.65 (dd, J = 16.0, 1.3 Hz, 1H), 6.27 (dd, J = 15.9, 6.4 Hz, 1H), 6.02 (ddd, J = 17.2, 10.4, 5.9 Hz, 1H), 5.38 (dt, J = 17.2, 1.4 Hz, 1H), 5.23 (dt, J = 10.4, 1.3 Hz, 1H), 4.84 (ddt, J = 6.1, 4.7, 1.4 Hz, 1H), 2.10 (s, 1H).

**$^{13}$C {1H} NMR** (101 MHz, $CDCl_3$): 139.3, 136.6, 130.8, 130.4, 128.6, 127.8, 126.5, 115.4, 73.8.

**5-phenylpent-1-en-3-ol**

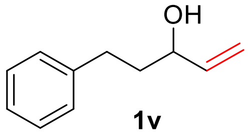

**1v**

**1v** was prepared according to **GP1**, obtained as light-yellow oil (0.66 g, 41% yield), the characterization of the title compound was consistent with the data available in the literature.[21]

**$^{1}$H NMR** (400 MHz, $CDCl_3$): δ 7.47 – 7.14 (m, 5H), 5.94 (ddd, J = 17.2, 10.4, 6.2 Hz, 1H), 5.28 (dt, J = 17.2, 1.4 Hz, 1H), 5.18 (dt, J = 10.4, 1.3 Hz, 1H), 4.16 (q, J = 6.4 Hz, 1H), 2.76 (qdd, J = 13.9, 8.7, 7.0 Hz, 2H), 2.14 – 1.82 (m, 2H), 1.78 (s, 1H).

**$^{13}$C {1H} NMR** (101 MHz, $CDCl_3$): 141.9, 141.0, 128.49, 128.43, 125.8, 114.9, 72.4, 38.5, 31.6.

**undec-1-en-3-ol**

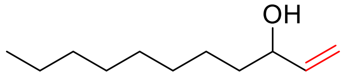

**1w**

**1w** was prepared according to **GP1**, obtained as light-yellow oil (0.60 g, 35% yield),the characterization of the title compound was consistent with the data available in the literature.[22]

**$^{1}$H NMR** (400 MHz, $CDCl_3$): δ 5.88 (ddd, J = 16.9, 10.4, 6.2 Hz, 1H), 5.23 (dt, J = 17.2, 1.5 Hz, 1H), 5.11 (dt, J = 10.4, 1.4 Hz, 1H), 4.10 (dd, J = 6.6, 3.7 Hz, 1H), 1.69 (t, J = 2.7 Hz, 1H), 1.53 (tt, J = 8.4, 3.8 Hz, 2H), 1.33 – 1.25 (m, 12H), 0.91 – 0.88 (m, 3H).

**$^{13}$C {1H} NMR** (101 MHz, $CDCl_3$): 141.3, 114.4, 73.2, 37.0, 31.8, 29.57, 29.55, 29.2, 25.3, 22.6, 14.0.

**1-cyclohexylprop-2-en-1-ol**

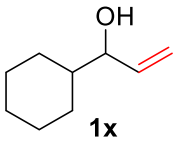


**1x**

**1x** was prepared according to **GP1**, obtained as colorless oil (0.70 g, 50% yield), the characterization of the title compound was consistent with the data available in the literature.[23]

**$^{1}$H NMR** (500 MHz, $CDCl_3$):δ 5.92 – 5.72 (m, 1H), 5.24 – 5.02 (m, 2H), 3.82 (q, J = 5.7 Hz, 1H), 2.08 – 1.91 (m, 1H), 1.88 – 1.80 (m, 1H), 1.79 – 1.61 (m, 4H), 1.38 (dddq, J = 12.3, 9.4, 6.2, 3.1 Hz, 1H), 1.31 – 1.04 (m, 4H), 0.98 (dddd, J = 19.4, 12.4, 9.6, 3.5 Hz, 2H).

**$^{13}$C {1H} NMR** (126 MHz, $CDCl_3$): δ 139.8, 115.3, 77.6, 43.4, 28.7, 28.3, 26.5, 26.1, 26.0.

**1-cyclopropylprop-2-en-1-ol**

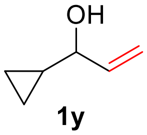


**1y**

**1y** was prepared according to **GP1**, obtained as colorless oil (0.45 g, 46% yield), the characterization of the title compound was consistent with the data available in the literature.[24]

**$^{1}$H NMR** (500 MHz, $CDCl_3$): δ 6.08 – 5.87 (m, 1H), 5.28 (dt, J = 17.2, 1.5 Hz, 1H), 5.13 (dt, J = 10.5, 1.4 Hz, 1H), 3.56 – 3.42 (m, 1H), 1.74 (s, 1H), 1.10 – 0.90 (m, 1H), 0.71 – 0.51 (m, 2H), 0.50 – 0.21 (m, 2H).

**$^{13}$C {1H} NMR** (126 MHz, $CDCl_3$): δ 139.6, 114.6, 17.3, 3.0, 1.9.

**1-(thiophen-3-yl)prop-2-en-1-ol**

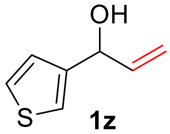


**1z**

**1z** was prepared according to **GP1**, obtained as light-yellow oil (0.99 g, 71% yield), the characterization of the title compound was consistent with the data available in the literature.[25]

**$^{1}$H NMR** (400 MHz, $CDCl_3$): δ 7.32 (dd, J = 5.0, 3.0 Hz, 1H), 7.22 (dt, J = 3.0, 1.1 Hz, 1H), 7.08 (dd, J = 5.0, 1.3 Hz, 1H), 6.09 (ddd, J = 17.1, 10.3, 6.1 Hz, 1H), 5.36 (dt, J = 17.1, 1.4 Hz, 1H), 5.32 – 5.13 (m, 2H), 2.62 – 2.40 (m, 1H).

**$^{13}$C {1H} NMR** (101 MHz, $CDCl_3$): δ 144.1, 139.7, 126.3, 126.2, 121.4, 115.3, 71.5.

## 7. Preparation of allylic amine products 3-4

### *GP2 - General Procedure for the heterogeneous Enantioselective iridium-catalyzed allylic amination reactions.*

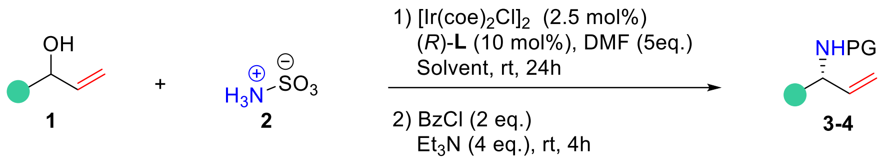


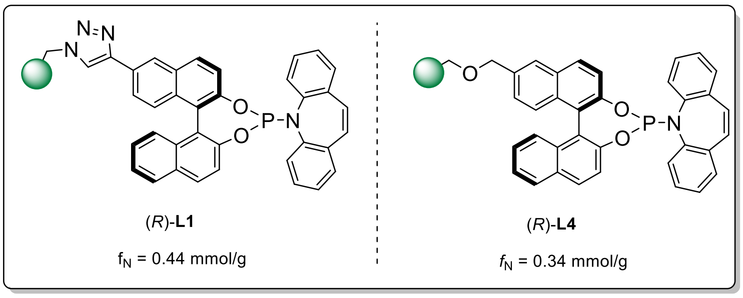


A freshly dried screw-capped vial, fitted with a rubber septum, was charged with $[Ir(COE)_2Cl]_2$ (5.6 mg, 6.25 μmol, 0.025 eq.), Ligand (*R*)-**L1** (57 mg, 0.025mmol, 0.1 eq.) or (*R*)-**L4** (74 mg, 0.025mmol, 0.1 eq.), and sulfamic acid (30 mg, 0.30 mmol, 1.2 eq.). After 3 vacuum-argon cycles of the reaction system, dimethylformamide (5 eq., 1.25 mmol, 0.1 mL) was added followed by 2-MeTHF (0.6 mL). The resulting mixture was placed in a shaker, and shaked for 15mins during which the solution and resin turned dark red. The substrate (0.25 mmol, 1.0 eq.) was added as a solution in 2-MeTHF (0.6 mL) to the reaction, which then turned yellow. The reaction was allowed to shake at room temperature for 24h. Under argon flow, the reaction was filtered and washed with DCM quickly, the resulting solid was dried under vacuum and kept under argon for further reuse. The combined organic portion was concentrated under reduced pressure and dissolved in 1 mL DCM, to which was added triethylamine (0.15 mL, 1.0 mmol 4eq.) and benzoyl chloride (55 μL, 0.5 mmol 2eq.), the resulting mixture was stirred at room temperature for 4h. The crude residue was then purified by column chromatography on silica gel using cyclohexane/EtOAc (12:1) as the eluent to give the desired ester product. In few cases, the purified products contain very small amount of starting material even after very carefully purification, but this don't have an influence on the products characterization.

### *GP3 - General Procedure for the heterogeneous Enantioselective iridium-catalyzed allylic amination reactions using* (*R*)-**L4**.

For compounds described in Table 3, following the same procedure of GP2, ligand (*R*)-**L4** (74 mg, 0.025mmol, 0.1 eq.) was used instead of Ligand (*R*)-**L1** (57 mg, 0.025mmol, 0.1 eq.)

## 8. Characterization Data for products 3-4

**(*R*)-*N*-(1-phenylallyl)benzamide**

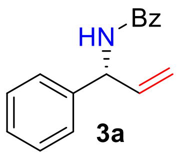

**3a**

**3a** was prepared according to GP3 (table 3), using **1a** (34 mg, 0.25 mmol), sulfamic acid **2** (30 mg, 0.30 mmol) and (*R*)-**L4** (74 mg, 0.025mmol) as the ligand. The crude mixture was purified by flash column chromatography (gradient from cyclohexane to 12% EtOAc in cyclohexane) to afford product **3a** (Table 3, 38.5 mg, 65% yield and 93% ee) as a white solid. A scale up experiment carried out with **1a** (340 mg, 2.5 mmol), sulfamic acid **2** (300 mg, 3.0 mmol) and (*R*)-**L1** (570 mg, 0.25 mmol) as the ligand afforded product **3a** in 55% yield and 91% ee (326 mg). The characterization of the title compound was consistent with the data available in the literature.[26] The absolute configuration was assigned by comparing with the literature value.

**$^1$H NMR** (500 MHz, $CDCl_3$): δ 7.94 – 7.73 (m, 2H), 7.60 – 7.29 (m, 8H), 6.53 (d, J = 8.0 Hz, 1H), 6.14 (ddd, J = 17.3, 10.1, 5.3 Hz, 1H), 5.88 (ddt, J = 8.4, 5.3, 1.7 Hz, 1H), 5.49 – 5.20 (m, 2H).).

**$^{13}$C {1H} NMR** (126 MHz, $CDCl_3$): δ 166.4, 140.5, 137.2, 134.3, 131.6, 128.8, 128.6, 127.8, 127.3, 126.9, 116.1, 55.5.

**$[\alpha]_D$**: +52.2 (*c* 1.15, $CH_2Cl_2$).

**HPLC** (Daicel Chiralpak AD-H column, hexane/*i*-PrOH (85:15), flow rate 1.0 mL/min, λ = 210 nm): $t_{major}$ = 8.059 min; $t_{minor}$ = 12.005 min. 93% ee.

**(*R*)-*N*-(1-(*o*-tolyl)allyl)benzamide**

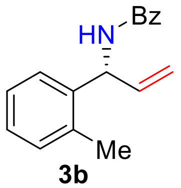

**3b**

**3b** was prepared according to GP2, using **1b** (37 mg, 0.25 mmol), sulfamic acid **2** (30 mg, 0.30 mmol) and (*R*)-**L1** (57 mg, 0.025mmol) as the ligand. The crude mixture was purified by flash column chromatography (gradient from cyclohexane to 12% EtOAc in cyclohexane) to afford product **3b** (43.3 mg, 69% yield and 90% ee) as a white solid. The characterization of the title compound was consistent with the data available in the literature.[27]

**$^1$H NMR** (500 MHz, $CDCl_3$): δ 7.90 – 7.74 (m, 2H), 7.62 – 7.30 (m, 4H), 7.27 – 7.18 (m, 3H), 6.46 (d, J = 8.1 Hz, 1H), 6.14 (ddd, J = 17.1, 10.3, 4.6 Hz, 1H), 6.05 (ddt, J = 8.2, 4.4, 1.9 Hz, 1H), 5.52 – 5.10 (m, 2H), 2.45 (s, 3H).

**$^{13}$C {1H} NMR** (126 MHz, $CDCl_3$): δ 166.4, 138.4, 137.0, 136.7, 134.2, 131.6, 130.9, 128.6, 127.8, 127.0, 126.7, 126.3, 115.7, 52.2, 19.3.

**$[\alpha]_D$**: +34.2 (*c* 0.72, $CH_2Cl_2$).

**HPLC** (Daicel Chiralpak AD-H column, hexane/*i*-PrOH (85:15), flow rate 1.0 mL/min, λ = 240 nm): $t_{major}$ = 6.434 min; $t_{minor}$ = 8.005 min. 90% ee.

**(*R*)-*N*-(1-(*m*-tolyl)allyl)benzamide**

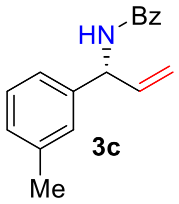


3c

**3c** was prepared according to GP2, using **1c** (37 mg, 0.25 mmol), sulfamic acid **2** (30 mg, 0.30 mmol) and (*R*)-**L1** (57 mg, 0.025mmol) as the ligand. The crude mixture was purified by flash column chromatography (gradient from cyclohexane to 12% EtOAc in cyclohexane) to afford product **3c** (42.8 mg, 68% yield and 91% ee) as a white solid. The title compound was previously unknown. **MP** = 66-68 °C

**$^1$H NMR** (400 MHz, $CDCl_3$): δ 7.93 – 7.68 (m, 2H), 7.63 – 7.39 (m, 3H), 7.33 – 7.07 (m, 4H), 6.49 (d, J = 8.2 Hz, 1H), 6.13 (ddd, J = 17.0, 10.4, 5.3 Hz, 1H), 5.84 (ddt, J = 8.5, 5.2, 1.8 Hz, 1H), 5.52 – 5.26 (m, 2H), 2.38 (d, J = 0.8 Hz, 3H).

**$^{13}$C {1H} NMR** (101 MHz, $CDCl_3$): δ 166.4, 140.5, 138.5, 137.3, 134.4, 131.5, 128.7, 128.6, 128.5, 128.1, 127.0, 124.3, 115.9, 55.5, 21.4.

**$[\alpha]_D$**: +40.7 (*c* 1.14, $CH_2Cl_2$).

**IR** (ATR): ν 3312, 1632, 1522,1324, 916, 691 $cm^{-1}$.

**HRMS** (ESI) m/z: $[M+Na]^+$ Calcd for $C_{17}H_{17}NONa^+$ 274.1202; Found 274.1196.

**HPLC** (Daicel Chiralpak AD-H column, hexane/*i*-PrOH (85:15), flow rate 1.0 mL/min, λ = 240 nm): $t_{major}$ = 6.730 min; $t_{minor}$ = 8.510 min. 91% ee.

**(*R*)-*N*-(1-(*p*-tolyl)allyl)benzamide**

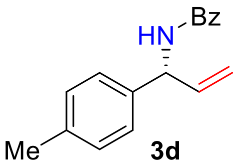


3d

**3d** was prepared according to GP2, using **1d** (37 mg, 0.25 mmol), sulfamic acid **2** (30 mg, 0.30 mmol) and (*R*)-**L1** (57 mg, 0.025mmol) as the ligand. The crude mixture was purified by flash column chromatography (gradient from cyclohexane to 12% EtOAc in cyclohexane) to afford product **3d** (40.8 mg, 65% yield and 88% ee) as a white solid. The characterization of the title compound was consistent with the data available in the literature.[28]

**$^1$H NMR** (400 MHz, $CDCl_3$): δ 8.17 – 7.70 (m, 2H), 7.62 – 7.39 (m, 3H), 7.36 – 7.12 (m, 4H), 6.51 (d, *J* = 8.1 Hz, 1H), 6.28 – 6.05 (m, 1H), 5.84 (ddd, *J* = 7.8, 4.5, 1.8 Hz, 1H), 5.39 – 5.18 (m, 2H), 2.37 (s, 3H).

**$^{13}$C {1H} NMR** (101 MHz, $CDCl_3$): 166.4, 137.6, 137.5, 137.3, 134.4, 131.5, 129.5, 128.5, 127.2, 127.0, 115.8, 55.3, 21.1.

**$[\alpha]_D$**: +53.0 (*c* 1.00, $CH_2Cl_2$).

**HPLC** (Daicel Chiralpak AD-H column, hexane/*i*-PrOH (85:15), flow rate 1.0 mL/min, λ = 240 nm): $t_{major}$ = 8.615 min; $t_{minor}$ = 12.628 min. 88% ee.

**(*R*)-*N*-(1-(2-methoxyphenyl)allyl)benzamide**

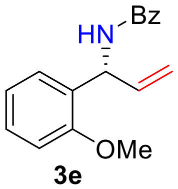

**3e**

**3e** was prepared according to GP2, using **1e** (41 mg, 0.25 mmol), sulfamic acid **2** (30 mg, 0.30 mmol) and (*R*)-**L1** (57 mg, 0.025mmol) as the ligand. The crude mixture was purified by flash column chromatography (gradient from cyclohexane to 12% EtOAc in cyclohexane) to afford product **3e** (42.7 mg, 64% yield and 90% ee) as a white solid. The characterization of the title compound was consistent with the data available in the literature.[27]

**$^1$H NMR** (500 MHz, $CDCl_3$): δ 7.86 – 7.72 (m, 2H), 7.62 – 7.40 (m, 3H), 7.32 (t, *J* = 7.5 Hz, 2H), 7.08 – 6.89 (m, 2H), 6.14 (ddd, *J* = 17.1, 10.2, 5.2 Hz, 1H), 6.03 – 5.88 (m, 1H), 5.30 – 5.05 (m, 2H), 3.92 (s, 3H).

**$^{13}$C {1H} NMR** (126 MHz, $CDCl_3$): δ 166.1, 157.2, 137.6, 134.8, 131.3, 129.3, 129.0, 128.5, 128.2, 126.9, 121.1, 114.9, 111.4, 55.6, 53.9.

**$[\alpha]_D$**: +48.6 (*c* 0.79, $CH_2Cl_2$).

**HPLC** (Daicel Chiralpak AD-H column, hexane/*i*-PrOH (85:15), flow rate 1.0 mL/min, λ = 240 nm): $t_{major}$ = 8.826 min; $t_{minor}$ = 16.479 min. 90% ee.

**(*R*)-*N*-(1-(3-methoxyphenyl)allyl)benzamide**

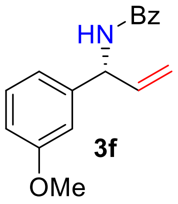

**3f**

**3f** was prepared according to GP3 (table 3), using **1f** (41 mg, 0.25 mmol), sulfamic acid **2** (30 mg, 0.30 mmol) and (*R*)-**L4** (74 mg, 0.025mmol) as the ligand. The crude mixture was purified by flash column chromatography (gradient from cyclohexane to 12% EtOAc in cyclohexane) to afford product **3f** (34.0 mg, 51% yield and 89% ee) as a white solid. The characterization of the title compound was consistent with the data available in the literature.[27]

**$^1$H NMR** (500 MHz, $CDCl_3$): δ 7.96 – 7.68 (m, 2H), 7.61 – 7.50 (m, 1H), 7.50 – 7.41 (m, 2H), 7.35 – 7.29 (m, 1H), 6.99 (ddt, *J* = 7.5, 1.7, 0.8 Hz, 1H), 6.94 (d, *J* = 2.0 Hz, 1H), 6.87 (ddd, *J* = 8.2, 2.6, 1.0 Hz, 1H), 6.39 (d, *J* = 8.1 Hz, 1H), 6.21 – 6.01 (m, 1H), 5.89 – 5.74 (m, 1H), 5.46 – 5.16 (m, 2H), 3.83 (d, *J* = 0.9 Hz, 3H).

**$^{13}$C {1H} NMR** (126 MHz, $CDCl_3$): δ 166.4, 159.9, 142.1, 137.0, 134.3, 131.6, 129.9, 128.6, 126.9, 119.5, 116.1, 113.2, 113.0, 55.4, 55.2.

**$[\alpha]_D$**: +14.7 (*c* 0.63, $CH_2Cl_2$).

**HPLC** (Daicel Chiralpak AD-H column, hexane/*i*-PrOH (85:15), flow rate 1.0 mL/min, λ = 240 nm): $t_{major}$ = 9.652 min; $t_{minor}$ = 12.524 min. 89% ee.

**(*R*)-*N*-(1-(4-methoxyphenyl)allyl)benzamide**

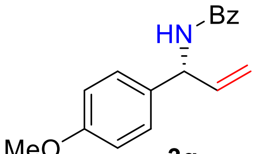


**3g**

**3g** was prepared according to GP3 (table 3), using **1g** (41 mg, 0.25 mmol), sulfamic acid **2** (30 mg, 0.30 mmol) and (*R*)-**L4** (74 mg, 0.025mmol) as the ligand. The crude mixture was purified by flash column chromatography (gradient from cyclohexane to 12% EtOAc in cyclohexane) to afford product **3g** (44.1 mg, 66% yield and 89% ee) as a white solid. The characterization of the title compound was consistent with the data available in the literature.[29]

**$^1$H NMR** (500 MHz, $CDCl_3$): δ 7.90 – 7.70 (m, 2H), 7.59 – 7.47 (m, 1H), 7.49 – 7.38 (m, 2H), 7.38 – 7.22 (m, 2H), 7.01 – 6.81 (m, 2H), 6.39 (d, *J* = 8.1 Hz, 1H), 6.13 (ddd, *J* = 17.4, 10.1, 5.1 Hz, 1H), 5.92 – 5.75 (m, 1H), 5.35 – 5.14 (m, 2H), 3.83 (s, 3H).

**$^{13}$C {1H} NMR** (126 MHz, $CDCl_3$): δ 166.4, 159.2, 137.3, 134.4, 132.6, 131.5, 128.6, 128.5, 126.9, 115.7, 114.2, 55.3, 54.9.

**$[\alpha]_D$**: +57.4 (*c* 0.95, $CH_2Cl_2$).

**HPLC** (Daicel Chiralpak AD-H column, hexane/*i*-PrOH (85:15), flow rate 1.0 mL/min, λ = 240 nm): $t_{major}$ = 11.193 min; $t_{minor}$ = 16.845 min. 89% ee.

**(*R*)-*N*-(1-(2-fluorophenyl)allyl)benzamide**

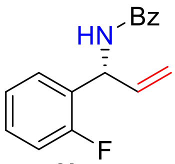


**3h**

**3h** was prepared according to GP3 (table 3), using **1h** (38 mg, 0.25 mmol), sulfamic acid **2** (30 mg, 0.30 mmol) and (*R*)-**L4** (74 mg, 0.025mmol) as the ligand. The crude mixture was purified by flash column chromatography (gradient from cyclohexane to 12% EtOAc in cyclohexane) to afford product **3h** (33.8 mg, 53% yield and 99% ee) as a white solid. The title compound was previously unknown. **MP** = 108-110 °C

**$^1$H NMR** (500 MHz, $CDCl_3$): δ 7.95 – 7.73 (m, 2H), 7.58 – 7.25 (m, 5H), 7.26 – 6.99 (m, 2H), 6.74 (d, *J* = 8.6 Hz, 1H), 6.32 – 6.08 (m, 1H), 6.03 (dt, *J* = 8.5, 3.5 Hz, 1H), 5.37 – 5.09 (m, 2H).

**$^{13}$C {1H} NMR** (126 MHz, $CDCl_3$): δ 166.4, 160.9(d, J = 246.4 Hz), 136.5, 134.2, 131.6, 129.5 (d, J = 8.4 Hz), 129.4 (d, J = 4.6 Hz), 128.6, 127.4 (d, J = 13.3 Hz). 127.0, 124.52 (d, J = 3.3 Hz), 116.2, 116.04 (d, J = 21.6 Hz), 52.1.

**$^{19}$F NMR** (471 MHz, $CDCl_3$) δ -117.12 (s, 1F).

**$[\alpha]_D$**: +7.2 (*c* 0.75, $CH_2Cl_2$).

**IR** (ATR): ν 3301, 1633, 1523, 1227, 923, 690 $cm^{-1}$.

**HRMS** (ESI) m/z: $[M+Na]^+$ Calcd for $C_{16}H_{14}FNONa^+$ 278.0952; Found 278.0948.

**HPLC** (Daicel Chiralpak AD-H column, hexane/*i*-PrOH (85:15), flow rate 1.0 mL/min, λ = 240 nm): $t_{major}$ = 8.658 min; $t_{minor}$ = 10.522 min. 99% ee.

**(*R*)-*N*-(1-(2-chlorophenyl)allyl)benzamide**

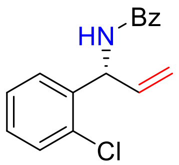

**3i**

**3i** was prepared according to GP3 (table 3), using **1i** (42 mg, 0.25 mmol), sulfamic acid **2** (30 mg, 0.30 mmol) and (*R*)-**L4** (74 mg, 0.025mmol) as the ligand. The crude mixture was purified by flash column chromatography (gradient from cyclohexane to 12% EtOAc in cyclohexane) to afford product **3i** (40.1 mg, 59% yield and 97% ee) as a white solid. The title compound was previously unknown. **MP** = 126-127 °C

**$^1$H NMR** (500 MHz, $CDCl_3$): δ 8.06 – 7.70 (m, 2H), 7.65 – 7.36 (m, 5H), 7.36 – 7.15 (m, 2H), 6.71 (d, *J* = 7.6 Hz, 1H), 6.26 – 6.00 (m, 2H), 5.43 – 5.18 (m, 2H).

**$^{13}$C {1H} NMR** (126 MHz, $CDCl_3$): δ 166.3, 137.7, 136.0, 134.2, 133.6, 131.6, 130.3, 129.2, 129.1, 128.6, 127.1, 127.0, 116.6, 53.8.

**$[\alpha]_D$**: –13.1 (*c* 0.71, $CH_2Cl_2$).

**IR** (ATR): ν 3305, 1634, 1524, 1351, 915, 693 $cm^{-1}$.

**HRMS** (ESI) m/z: $[M+H]^+$ Calcd for $C_{16}H_{15}ClNO^+$: 272.0837; Found 272.0843.

**HPLC** (Daicel Chiralpak AD-H column, hexane/*i*-PrOH (85:15), flow rate 1.0 mL/min, λ = 240 nm): $t_{major}$ = 7.646 min; $t_{minor}$ = 9.950 min. 97% ee.

**(*R*)-*N*-(1-(4-chlorophenyl)allyl)benzamide**

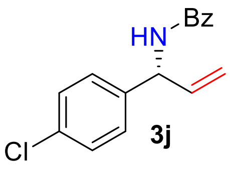

**3j**

**3j** was prepared according to GP3 (table 3), using **1j** (42 mg, 0.25 mmol), sulfamic acid **2** (30 mg, 0.30 mmol) and (*R*)-**L4** (74 mg, 0.025mmol) as the ligand. The crude mixture was purified by flash column chromatography (gradient from cyclohexane to 12% EtOAc in cyclohexane) to afford product **3j** (45.4 mg, 67% yield and 92% ee) as a white solid. The title compound was previously unknown. **MP** = 111-113 °C

**$^1$H NMR** (500 MHz, $CDCl_3$): δ 7.93 – 7.73 (m, 2H), 7.62 – 7.49 (m, 1H), 7.45 (ddt, *J* = 8.2, 6.6, 1.2 Hz, 2H), 7.42 – 7.22 (m, 4H), 6.48 (d, *J* = 8.1 Hz, 1H), 6.24 – 5.94 (m, 1H), 5.83 (ddt, *J* = 7.4, 5.4, 1.7 Hz, 1H), 5.44 – 5.20 (m, 2H).

**$^{13}$C {1H} NMR** (126 MHz, $CDCl_3$): δ 166.5, 139.0, 136.7, 134.1, 133.5, 131.7, 128.9, 128.6 (x2), 127.0, 116.8, 54.9.

**[α]D**: +25.9 (*c* 1.00, $CH_2Cl_2$).

**IR** (ATR): ν 3304, 1634, 1523, 1090, 920, 692 $cm^{-1}$.

**HRMS** (ESI) m/z: $[M+Na]^+$ Calcd for $C_{16}H_{14}ClNNaO^+$294.0656; Found 294.0652.

**HPLC** (Daicel Chiralpak AD-H column, hexane/*i*-PrOH (85:15), flow rate 1.0 mL/min, λ = 240 nm): $t_{major}$ = 8.423 min; $t_{minor}$ = 11.809 min. 92% ee.

**(*R*)-*N*-(1-(4-bromophenyl)allyl)benzamide**

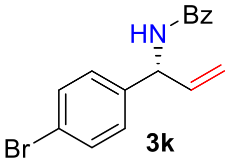

3k

**3k** was prepared according to GP2, using **1k** (53 mg, 0.25 mmol), sulfamic acid **2** (30 mg, 0.30 mmol) and (*R*)-**L1** (57 mg, 0.025mmol) as the ligand. The crude mixture was purified by flash column chromatography (gradient from cyclohexane to 12% EtOAc in cyclohexane) to afford product **3k** (49.6 mg, 63% yield and 87% ee) as a white solid. The title compound was previously unknown. **MP** = 153-155 °C

**$^1$H NMR** (500 MHz, $CDCl_3$): δ 7.95 – 7.71 (m, 2H), 7.65 – 7.42 (m, 5H), 7.27 (dd, *J* = 8.5, 6.5 Hz, 2H), 6.47 (d, *J* = 8.0 Hz, 1H), 6.30 – 6.01 (m, 1H), 5.82 (dt, *J* = 7.8, 3.6 Hz, 1H), 5.55 – 5.16 (m, 2H).

**$^{13}$C {1H} NMR** (126 MHz, $CDCl_3$): δ 166.5, 139.6, 136.7, 134.1, 131.9, 131.7, 129.0, 128.6, 127.0, 121.7, 116.9, 55.0.

**[α]D**: +19.5 (*c* 1.10, $CH_2Cl_2$).

**IR** (ATR): ν 3283, 1633, 1528, 1335, 930, 694 $cm^{-1}$.

**HRMS** (ESI) m/z: $[M+Na]^+$ Calcd for $C_{16}H_{14}BrNNaO^+$338.0151; Found 338.0151.

**HPLC** (Daicel Chiralpak AD-H column, hexane/*i*-PrOH (85:15), flow rate 1.0 mL/min, λ = 240 nm): $t_{major}$ = 8.873 min; $t_{minor}$ = 13.366min. 87% ee.

**(*R*)-*N*-(1-(4-iodophenyl)allyl)benzamide**

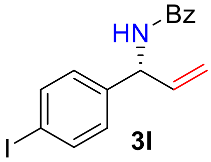

3l

**3l** was prepared according to GP2, using **1l** (65 mg, 0.25 mmol), sulfamic acid **2** (30 mg, 0.30 mmol) and (*R*)-**L1** (57 mg, 0.025mmol) as the ligand. The crude mixture was purified by flash column chromatography (gradient from cyclohexane to 12% EtOAc in cyclohexane) to afford product **3l** (43.6 mg, 48% yield and 86% ee) as a white solid. The title compound was previously unknown. **MP** = 157-159 °C

**$^1$H NMR** (500 MHz, $CDCl_3$): δ 7.88 – 7.75 (m, 2H), 7.70 (dd, *J* = 8.6, 2.3 Hz, 2H), 7.61 – 7.39 (m, 3H), 7.14 (dd, *J* = 8.0, 5.7 Hz, 2H), 6.50 (d, *J* = 8.1 Hz, 1H), 6.08 (ddd, *J* = 17.1, 10.3, 5.5 Hz, 1H), 5.79 (ddt, *J* = 7.5, 5.6, 1.7 Hz, 1H), 5.42 – 5.20 (m, 2H).

**$^{13}$C {1H} NMR** (126 MHz, $CDCl_3$): δ 166.5, 140.3, 137.8, 137.5, 136.6, 134.1, 131.7, 129.2, 128.6, 128.3, 127.0, 116.9, 55.1.

**[α]$_D$**: +23.0 (*c* 1.00, $CH_2Cl_2$).

**IR** (ATR): ν 3282, 1633, 1528, 925, 695 cm$^{-1}$.

**HRMS** (ESI) m/z: [M+Na]$^+$ Calcd for $C_{16}H_{14}INNaO^+$ 386.0012; Found 386.0005.

**HPLC** (Daicel Chiralpak AD-H column, hexane/*i*-PrOH (85:15), flow rate 1.0 mL/min, λ = 240 nm): $t_{major}$ = 9.530 min; $t_{minor}$ = 13.526 min. 86% ee.

**(*R*)-*N*-(1-(4-nitrophenyl)allyl)benzamide**

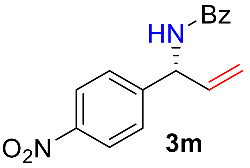


**3m** was prepared according to GP2, using **1m** (45 mg, 0.25 mmol), sulfamic acid **2** (30 mg, 0.30 mmol) and (*R*)-**L1** (57 mg, 0.025mmol) as the ligand. The crude mixture was purified by flash column chromatography (gradient from cyclohexane to 12% EtOAc in cyclohexane) to afford product **3m** (24.7 mg, 35% yield and 92% ee) as a white solid. The characterization of the title compound was consistent with the data available in the literature.[28]

**$^1$H NMR** (500 MHz, $CDCl_3$): δ 8.44 – 8.11 (m, 2H), 8.03 – 7.73 (m, 2H), 7.71 – 7.37 (m, 5H), 6.59 (d, *J* = 7.7 Hz, 1H), 6.11 (ddd, *J* = 17.1, 10.3, 5.9 Hz, 1H), 5.92 (td, *J* = 6.8, 5.9, 1.7 Hz, 1H), 5.47 – 5.28 (m, 2H).

**$^{13}$C {1H} NMR** (126 MHz, $CDCl_3$): δ 166.7, 147.9, 135.9, 133.7, 132.0, 128.7, 128.0, 127.0, 126.9, 124.0, 118.3, 55.3.

**[α]$_D$**: –10.6 (*c* 0.63, $CH_2Cl_2$).

**HPLC** (Daicel Chiralpak AD-H column, hexane/*i*-PrOH (85:15), flow rate 1.0 mL/min, λ = 240 nm): $t_{major}$ = 12.772 min; $t_{minor}$ = 28.878 min. 92% ee.

**(*R*)-*N*-(1-(4-(trifluoromethyl)phenyl)allyl)benzamide**

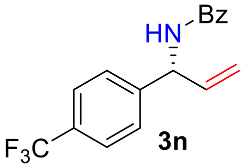


**3n** was prepared according to GP2, using **1n** (51 mg, 0.25 mmol), sulfamic acid **2** (30 mg, 0.30 mmol) and (*R*)-**L1** (57 mg, 0.025mmol) as the ligand. The crude mixture was purified by flash column chromatography (gradient from cyclohexane to 12% EtOAc in cyclohexane) to afford product **3n** (43.8 mg, 50% yield and 94% ee) as a white solid. The characterization of the title compound was consistent with the data available in the literature.[28]

**$^{1}$H NMR** (500 MHz, $CDCl_3$): δ 7.99 – 7.76 (m, 2H), 7.70 – 7.41 (m, 7H), 6.60 (d, *J* = 8.0 Hz, 1H), 6.11 (ddd, *J* = 17.1, 10.3, 5.7 Hz, 1H), 5.94 – 5.82 (m, 1H), 5.52 – 5.25 (m, 2H).

**$^{13}$C {1H} NMR** (126 MHz, $CDCl_3$): 166.6, 144.5, 136.4, 133.9, 131.8, 130.0 (q, J = 31.5 Hz), 128.7, 127.5, 127.0, 125.7 (q, J = 3.8 Hz), 124.0 (d, J = 272.0 Hz), 117.4, 55.3.

**$^{19}$F NMR** (471 MHz, $CDCl_3$) δ -62.5 (s, 3F).

**[α]$_D$**: +6.9 (*c* 1.04, $CH_2Cl_2$).

**HPLC** (Daicel Chiralpak AD-H column, hexane/*i*-PrOH (85:15), flow rate 1.0 mL/min, λ = 240 nm): $t_{major}$ = 6.322 min; $t_{minor}$ = 8.403 min. 94% ee.

**Methyl (*R*)-4-(1-benzamidoallyl)benzoate**

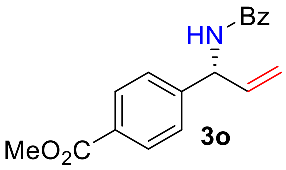


**3o**

**3o** was prepared according to GP2, using **1o** (48 mg, 0.25 mmol), sulfamic acid **2** (30 mg, 0.30 mmol) and (*R*)-**L1** (57 mg, 0.025mmol) as the ligand. The crude mixture was purified by flash column chromatography (gradient from cyclohexane to 12% EtOAc in cyclohexane) to afford product **3o** (32.5 mg, 44% yield and 94% ee) as a white solid. The characterization of the title compound was consistent with the data available in the literature.[28]

**$^{1}$H NMR** (500 MHz, $CDCl_3$): δ 8.18 – 8.00 (m, 2H), 7.91 – 7.75 (m, 2H), 7.64 – 7.35 (m, 5H), 6.50 (d, *J* = 8.1 Hz, 1H), 6.12 (ddd, *J* = 17.1, 10.3, 5.6 Hz, 1H), 5.92 (dd, *J* = 8.0, 5.9 Hz, 1H), 5.57 – 5.25 (m, 2H).

**$^{13}$C {1H} NMR** (126 MHz, $CDCl_3$): δ 166.7, 166.5, 145.6, 136.6, 134.0, 131.8, 130.1, 129.5, 128.6, 127.2, 127.0, 117.2, 55.3, 52.1.

**[α]$_D$**: +19.8 (*c* 0.85, $CH_2Cl_2$).

**HPLC** (Daicel Chiralpak AD-H column, hexane/*i*-PrOH (85:15), flow rate 1.0 mL/min, λ = 240 nm): $t_{major}$ = 13.046 min; $t_{minor}$ = 20.395 min. 94% ee.

**(*R*)-*N*-(1-(4-((trimethylsilyl)ethynyl)phenyl)allyl)benzamide**

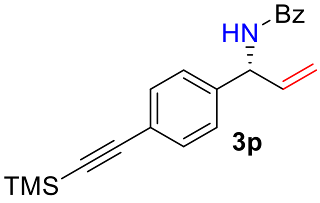


**3p**

**3p** was prepared according to GP2, using **1p** (58 mg, 0.25 mmol), sulfamic acid **2** (30 mg, 0.30 mmol) and (*R*)-**L1** (57 mg, 0.025mmol) as the ligand. The crude mixture was purified by flash column chromatography (gradient from cyclohexane to 12% EtOAc in cyclohexane) to afford product **3p** (40.8 mg, 49% yield and 91% ee) as a white solid. The title compound was previously unknown. **MP** = 68-70 °C

$^1$**H NMR** (500 MHz, $CDCl_3$): δ 7.85 – 7.69 (m, 2H), 7.57 – 7.50 (m, 1H), 7.50 – 7.40 (m, 4H), 7.36 – 7.29 (m, 2H), 6.43 (d, *J* = 8.2 Hz, 1H), 6.19 – 6.03 (m, 1H), 5.86 (t, *J* = 6.9 Hz, 1H), 5.38 – 5.22 (m, 2H), 0.27 (s, 9H).

$^{13}$**C {1H} NMR** (101 MHz, $CDCl_3$): δ 166.5, 140.8, 136.7, 134.2, 132.3, 131.7, 128.6, 127.1, 126.9, 122.6, 116.7, 104.6, 94.6, 55.2, 0.0.

**[α]$_D$**: +35.6 (*c* 0.66, $CH_2Cl_2$).

**IR** (ATR): ν 3289, 2959, 2157, 1634, 837, 693 cm$^{-1}$.

**HRMS** (ESI) m/z: $[M+H]^+$ Calcd for $C_{21}H_{24}NOSi^+$ 334.1622; Found 334.1615.

**HPLC** (Daicel Chiralpak AD-H column, hexane/*i*-PrOH (95:5), flow rate 1.0 mL/min, λ = 240 nm): $t_{major}$ = 15.472 min; $t_{minor}$ = 17.960 min. 91% ee.

**(*R*)-*N*-(1-(4-(*tert*-butyl)phenyl)allyl)benzamide**

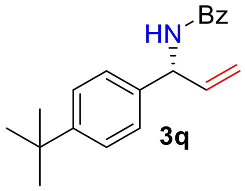

**3q**

**3q** was prepared according to GP2, using **1q** (48 mg, 0.25 mmol), sulfamic acid **2** (30 mg, 0.30 mmol) and (*R*)-**L1** (57 mg, 0.025mmol) as the ligand. The crude mixture was purified by flash column chromatography (gradient from cyclohexane to 12% EtOAc in cyclohexane) to afford product **3q** (33.7 mg, 46% yield and 78% ee) as a white solid. The title compound was previously unknown. **MP** = 136-138 °C

$^1$**H NMR** (500 MHz, $CDCl_3$): δ 7.85 – 7.79 (m, 2H), 7.55 – 7.49 (m, 1H), 7.48 – 7.39 (m, 4H), 7.34 (s, 1H), 6.41 (d, *J* = 8.2 Hz, 1H), 6.14 (ddd, *J* = 17.1, 10.3, 5.3 Hz, 1H), 5.86 (dt, *J* = 8.0, 3.6 Hz, 1H), 5.38 – 5.32 (m, 1H), 5.35 – 5.29 (m, 1H), 1.34 (s, 9H).

$^{13}$**C {1H} NMR** (126 MHz, $CDCl_3$): δ 166.4, 150.8, 137.4, 137.2, 134.4, 131.5, 128.6, 127.1, 127.0, 125.7, 115.8, 55.2, 34.5, 31.3.

**[α]$_D$**: +31.3 (*c* 1.00, $CH_2Cl_2$).

**IR** (ATR): ν 3320, 2959, 1631, 1311, 690, 587 cm$^{-1}$.

**HRMS** (ESI) m/z: $[M+Na]^+$ Calcd for $C_{20}H_{23}NNaO^+$ 316.1672; Found 316.1672.

**HPLC** (Daicel Chiralpak AD-H column, hexane/*i*-PrOH (85:15), flow rate 1.0 mL/min, λ = 240 nm): $t_{major}$ = 5.781 min; $t_{minor}$ = 6.819 min. 78% ee.

**(*R*)-*N*-(1-(benzo[*d*][1,3]dioxol-5-yl)allyl)benzamide**

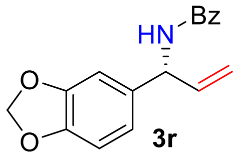

**3r**

**3r** was prepared according to GP2, using **1r** (45 mg, 0.25 mmol), sulfamic acid **2** (30 mg, 0.30 mmol) and (*R*)-**L1** (57 mg, 0.025mmol) as the ligand. The crude mixture was purified by flash column chromatography (gradient from cyclohexane to 12% EtOAc in

cyclohexane) to afford product **3r** (40.0 mg, 57% yield and 78% ee) as a white solid. The characterization of the title compound was consistent with the data available in the literature.[28]

**$^1$H NMR** (500 MHz, $CDCl_3$): δ 7.95 – 7.76 (m, 2H), 7.59 – 7.35 (m, 3H), 6.98 – 6.72 (m, 3H), 6.41 (d, *J* = 8.1 Hz, 1H), 6.09 (ddd, *J* = 17.3, 10.2, 5.2 Hz, 1H), 5.97 (s, 2H), 5.87 – 5.67 (m, 1H), 5.44 – 5.21 (m, 2H).

**$^{13}$C {1H} NMR** (126 MHz, $CDCl_3$): δ 166.4, 148.0, 147.1, 137.2, 134.5, 134.3, 131.6, 128.6, 126.9, 120.7, 116.0, 108.4, 107.8, 101.1, 55.3.

**[α]$_D$**: +51.7 (*c* 0.82, $CH_2Cl_2$).

**HPLC** (Daicel Chiralpak AD-H column, hexane/*i*-PrOH (85:15), flow rate 1.0 mL/min, λ = 240 nm): $t_{major}$ = 12.449 min; $t_{minor}$ = 18.966 min. 78% ee.

**(*R*)-*N*-(1-(phenanthren-9-yl)allyl)benzamide**

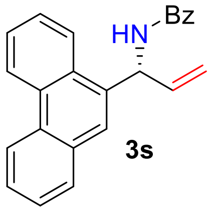

3s

**3s** was prepared according to GP2, using **1s** (59 mg, 0.25 mmol), sulfamic acid **2** (30 mg, 0.30 mmol) and (*R*)-**L1** (57 mg, 0.025mmol) as the ligand. The crude mixture was purified by flash column chromatography (gradient from cyclohexane to 12% EtOAc in cyclohexane) to afford product **3s** (45.5 mg, 54% yield and 93% ee) as a white solid. The title compound was previously unknown. **MP** = 213-215 °C

**$^1$H NMR** (500 MHz, $CDCl_3$): δ 8.82 – 8.76 (m, 1H), 8.75 – 8.69 (m, 1H), 8.26 (dd, *J* = 8.1, 1.5 Hz, 1H), 7.90 (dd, *J* = 7.8, 1.4 Hz, 1H), 7.85 – 7.76 (m, 3H), 7.75 – 7.61 (m, 4H), 7.56 – 7.33 (m, 3H), 6.68 (dq, *J* = 6.1, 2.0 Hz, 1H), 6.50 – 6.29 (m, 2H), 5.63 – 5.42 (m, 2H), 5.32 (s, 1H).

**$^{13}$C {1H} NMR** (126 MHz, $CDCl_3$): δ 166.5, 136.6, 134.3, 134.1, 131.7, 131.1, 131.0, 130.4, 130.1, 128.7, 128.6, 127.3, 127.2, 127.0, 126.9, 126.8, 126.5, 124.3, 123.2, 122.5, 116.1, 51.7.

**[α]$_D$**: +175.8 (*c* 0.62, $CH_2Cl_2$).

**IR** (ATR): ν 3282, 1627, 1519, 1280, 920, 695, 428 $cm^{-1}$.

**HRMS** (ESI) m/z: $[M+Na]^+$ Calcd for $C_{24}H_{19}NNaO^+$ 360.1359; Found 360.1357.

**HPLC** (Daicel Chiralpak AD-H column, hexane/*i*-PrOH (85:15), flow rate 1.0 mL/min, λ = 240 nm): $t_{major}$ = 9.637 min; $t_{minor}$ = 22.598 min. 93% ee.

***tert*-Butyl (*R*)-3-(1-benzamidoallyl)-1*H*-indole-1-carboxylatee**

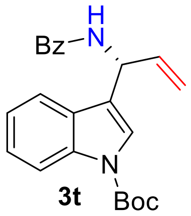

3t

**3t** was prepared according to GP2, using **1t** (68 mg, 0.25 mmol), sulfamic acid **2** (30 mg, 0.30 mmol) and (*R*)-**L1** (57 mg, 0.025mmol) as the ligand. The crude mixture was purified by flash column chromatography (gradient from cyclohexane to 40% EtOAc in cyclohexane) to afford product **3t** (49.8 mg, 53% yield and 79% ee) as a white solid. The title compound was previously unknown. **MP** = 131-132 °C

**$^{1}$H NMR** (500 MHz, $CDCl_3$): δ 8.16 (d, *J* = 8.3 Hz, 1H), 7.90 – 7.72 (m, 2H), 7.66 (dt, *J* = 7.8, 1.0 Hz, 1H), 7.62 – 7.19 (m, 6H), 6.39 (d, *J* = 8.3 Hz, 1H), 6.28 (ddd, *J* = 17.1, 10.3, 4.9 Hz, 1H), 6.21 – 6.07 (m, 1H), 5.52 – 5.30 (m, 2H), 1.70 (s, 9H).

**$^{13}$C {1H} NMR** (126 MHz, $CDCl_3$): δ 166.6, 149.6, 135.8, 134.2, 131.6, 128.7, 128.6, 126.9, 124.8, 123.9, 122.9, 120.2, 119.6, 116.2, 115.4, 84.0, 47.9, 28.2.

**$[\alpha]_D$**: +85.9 (*c* 0.51, $CH_2Cl_2$).

**IR** (ATR): ν 2920, 1735, 1633, 1367, 1151, 694 $cm^{-1}$.

**HRMS** (ESI) m/z: $[M+Na]^+$ Calcd for $C_{23}H_{24}N_2NaO_3^+$ 399.1679; Found 399.1679.

**HPLC** (Daicel Chiralpak AD-H column, hexane/*i*-PrOH (85:15), flow rate 1.0 mL/min, λ = 240 nm): $t_{major}$ = 5.744 min; $t_{minor}$ = 7.269 min. 79% ee.

**(*R,E*)-*N*-(1-phenylpenta-1,4-dien-3-yl)benzamide**

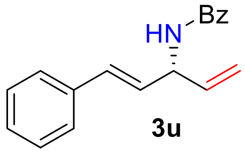

3u

**3u** was prepared according to GP2, using **1u** (40 mg, 0.25 mmol), sulfamic acid **2** (30 mg, 0.30 mmol) and (*R*)-**L1** (57 mg, 0.025mmol) as the ligand. The crude mixture was purified by flash column chromatography (gradient from cyclohexane to 12% EtOAc in cyclohexane) to afford product **3u** (27.6 mg, 42% yield and 75% ee) as a white solid. The title compound was previously unknown. **MP** = 135-136 °C

**$^{1}$H NMR** (500 MHz, $CDCl_3$): δ 8.02 – 7.75 (m, 2H), 7.59 – 7.51 (m, 1H), 7.51 – 7.44 (m, 2H), 7.42 – 7.38 (m, 2H), 7.34 (dd, *J* = 8.5, 6.8 Hz, 2H), 7.31 – 7.25 (m, 1H), 6.65 (dd, *J* = 16.0, 1.5 Hz, 1H), 6.30 (dd, *J* = 16.0, 6.1 Hz, 2H), 6.05 (ddd, *J* = 17.3, 10.4, 5.3 Hz, 1H), 5.49 (ddd, *J* = 6.4, 2.9, 1.4 Hz, 1H), 5.44 – 5.26 (m, 2H).

**$^{13}$C {1H} NMR** (126 MHz, $CDCl_3$): δ 166.5, 136.7, 136.4, 134.4, 131.8, 131.6, 128.7, 128.6, 127.8 (x2), 127.0 (x2), 126.5, 116.3, 53.0.

**$[\alpha]_D$**: +32.3 (*c* 0.56, $CH_2Cl_2$).

**IR** (ATR): ν 3316, 2960, 1632, 1312, 920, 691 $cm^{-1}$.

**HRMS** (ESI) m/z: $[M+Na]^+$ Calcd for $C_{18}H_{17}NNaO^+$ 286.1202; Found 286.1198.

**HPLC** (Daicel Chiralpak AD-H column, hexane/*i*-PrOH (85:15), flow rate 1.0 mL/min, λ = 254 nm): $t_{major}$ = 8.052 min; $t_{minor}$ = 9.828 min. 75% ee.

**(S)-*N*-(5-phenylpent-1-en-3-yl)benzamide**

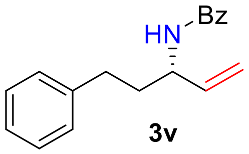


**3v**

**3v** was prepared according to GP2, using **1v** (41 mg, 0.25 mmol), sulfamic acid **2** (30 mg, 0.30 mmol) and (*R*)-**L1** (29 mg, 0.0125mmol) as the ligand. The crude mixture was purified by flash column chromatography (gradient from cyclohexane to 12% EtOAc in cyclohexane) to afford product **3v** (25.8 mg, 39% yield and 74% ee) as a white solid. The characterization of the title compound was consistent with the data available in the literature.[27]

**$^1$H NMR** (500 MHz, $CDCl_3$): δ 7.84 – 7.67 (m, 2H), 7.56 – 7.48 (m, 1H), 7.49 – 7.41 (m, 2H), 7.34 – 7.27 (m, 2H), 7.27 – 7.19 (m, 3H), 6.05 (d, *J* = 8.6 Hz, 1H), 5.93 (ddd, *J* = 17.2, 10.4, 5.5 Hz, 1H), 5.46 – 5.13 (m, 2H), 4.79 (ddt, *J* = 8.9, 7.5, 5.8 Hz, 1H), 2.78 (t, *J* = 7.9 Hz, 2H), 2.03 (dddd, *J* = 14.5, 13.4, 6.8, 4.4 Hz, 2H).

**$^{13}$C {1H} NMR** (126 MHz, $CDCl_3$): δ 166.7, 141.5, 138.0, 134.5, 131.4, 128.6, 128.5, 128.4, 126.8, 126.0, 115.4, 51.6, 36.3, 32.2.

**$[\alpha]_D$**: +17.6 (*c* 0.58, $CH_2Cl_2$).

**HPLC** (Daicel Chiralpak AD-H column, hexane/*i*-PrOH (85:15), flow rate 1.0 mL/min, λ = 240 nm): $t_{major}$ = 12.285 min; $t_{minor}$ = 13.184 min. 74% ee.

**(*S*)-*N*-(undec-1-en-3-yl)benzamidee**

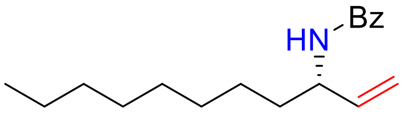


**3w**

**3w** was prepared according to GP2, using **1w** (43 mg, 0.25 mmol), sulfamic acid **2** (30 mg, 0.30 mmol) and (*R*)-**L1** (29 mg, 0.0125mmol) as the ligand. The crude mixture was purified by flash column chromatography (gradient from cyclohexane to 12% EtOAc in cyclohexane) to afford product **3w** (12.3 mg, 18% yield and 72% ee) as a white solid. The title compound was previously unknown. **MP** = 74-76 °C

**$^1$H NMR** (500 MHz, $CDCl_3$): δ 7.87 – 7.71 (m, 2H), 7.57 – 7.39 (m, 3H), 6.02 (d, *J* = 8.6 Hz, 1H), 5.88 (ddd, *J* = 17.3, 10.4, 5.6 Hz, 1H), 5.29 – 5.06 (m, 2H), 4.73 – 4.61 (m, 1H), 1.69 – 1.60 (m, 2H), 1.42 – 1.26 (m, 12H), 0.89 (t, *J* = 6.8 Hz, 3H).

**$^{13}$C {1H} NMR** (126 MHz, $CDCl_3$): δ 166.7, 138.5, 134.8, 131.4, 128.5, 126.8, 114.9, 51.7, 35.0, 31.8, 29.4 (x2) 29.2, 25.8, 22.6, 14.1.

**$[\alpha]_D$**: +22.0 (*c* 0.30, $CH_2Cl_2$).

**IR** (ATR): ν 3310, 2918, 1633, 1524, 923, 694 $cm^{-1}$.

**HRMS** (ESI) m/z: [M-H]- Calcd for $C_{18}H_{26}NO^-$ 272.2020; Found 272.2018.

**HPLC** (Daicel Chiralpak OD-H column, hexane/*i*-PrOH (90:10), flow rate 1.0 mL/min, λ = 240 nm): $t_{minor}$ = 7.052 min; $t_{major}$ = 8.523 min. 72% ee.

**(*R*)-*N*-(1-cyclohexylallyl)benzamide**

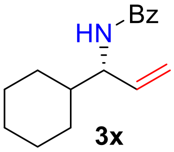


**3x** was prepared according to GP2, using **1x** (35 mg, 0.25 mmol), sulfamic acid **2** (30 mg, 0.30 mmol) and (*R*)-**L1** (29 mg, 0.0125mmol) as the ligand. The crude mixture was purified by flash column chromatography (gradient from cyclohexane to 12% EtOAc in cyclohexane) to afford product **3x** (10.4 mg, 17% yield and 81% ee) as a white solid. The characterization of the title compound was consistent with the data available in the literature.[27]

**¹H NMR** (500 MHz, $CDCl_3$): δ 8.00 – 7.70 (m, 2H), 7.63 – 7.41 (m, 3H), 6.09 (d, *J* = 9.1 Hz, 1H), 5.87 (ddd, *J* = 16.8, 10.4, 6.1 Hz, 1H), 5.34 – 5.09 (m, 2H), 4.74 – 4.46 (m, 1H), 1.81 – 1.68 (m, 4H), 1.59 (ddt, *J* = 8.6, 5.8, 2.7 Hz, 1H), 1.33 – 1.02 (m, 6H).

**$^{13}$C {1H} NMR** (126 MHz, $CDCl_3$): δ 166.8, 136.9, 134.9, 131.4, 128.6, 126.8, 115.7, 56.4, 42.2, 29.4, 28.8, 26.3, 26.2, 26.1.

**$[\alpha]_D$**: +51.8 (*c* 0.64, $CH_2Cl_2$).

**HPLC** (Daicel Chiralpak OD-H column, hexane/*i*-PrOH (90:10), flow rate 1.0 mL/min, λ = 240 nm): $t_{major}$ = 11.802 min; $t_{minor}$ = 14.456 min. 81% ee.

**(*R*)-*N*-(1-cyclopropylallyl)benzamide**

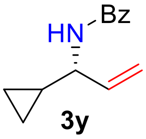


**3y** was prepared according to GP2, using **1y** (25 mg, 0.25 mmol), sulfamic acid **2** (30 mg, 0.30 mmol) and (*R*)-**L1** (29 mg, 0.0125mmol) as the ligand. The crude mixture was purified by flash column chromatography (gradient from cyclohexane to 8% EtOAc in hexane) to afford product **3y** (14.1 mg, 28% yield and 79% ee) as a white solid. The characterization of the title compound was consistent with the data available in the literature.[28]

**¹H NMR** (500 MHz, $CDCl_3$): δ 7.95 – 7.73 (m, 2H), 7.60 – 7.38 (m, 3H), 6.39 – 6.13 (m, 1H), 5.95 (ddd, *J* = 17.3, 10.4, 5.0 Hz, 1H), 5.32 (dt, *J* = 17.2, 1.5 Hz, 1H), 5.19 (dt, *J* = 10.5, 1.4 Hz, 1H), 4.16 (tdt, *J* = 8.4, 5.0, 1.7 Hz, 1H), 1.08 – 0.97 (m, 1H), 0.68 – 0.45 (m, 3H), 0.40 (ddt, *J* = 9.3, 5.7, 4.6 Hz, 1H).

**$^{13}$C {1H} NMR** (126 MHz, $CDCl_3$): δ 166.7, 137.2, 134.7, 131.4, 128.5, 126.9, 115.0, 55.5, 15.4, 3.1, 3.0.

**$[\alpha]_D$**: +26.9 (*c* 0.63, $CH_2Cl_2$).

**HPLC** (Daicel Chiralpak IC column, hexane/*i*-PrOH (95:5), flow rate 1.0 mL/min, λ = 240 nm): $t_{major}$ = 27.574 min; $t_{minor}$ = 34.816 min. 79% ee.

**(*R*)-*N*-(1-(thiophen-3-yl)allyl)benzamide**

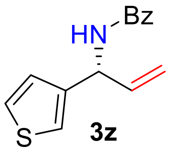


**3z** was prepared according to GP3 (table 3), using **1z** (35 mg, 0.25 mmol), sulfamic acid **2** (30 mg, 0.30 mmol) and (*R*)-**L4** (74 mg, 0.025mmol) as the ligand. The crude mixture was purified by flash column chromatography (gradient from cyclohexane to 8% EtOAc in hexane) to afford product **3z** (41.3 mg, 68% yield and 87% ee) as a white solid. The characterization of the title compound was consistent with the data available in the literature.[27]

**$^1$H NMR** (500 MHz, $CDCl_3$): δ 7.88 – 7.80 (m, 2H), 7.57 – 7.50 (m, 1H), 7.50 – 7.41 (m, 2H), 7.36 (dd, *J* = 5.0, 3.0 Hz, 1H), 7.24 (dt, *J* = 3.1, 1.1 Hz, 1H), 7.11 (dd, *J* = 5.0, 1.3 Hz, 1H), 6.41 (d, *J* = 8.4 Hz, 1H), 6.17 (ddd, *J* = 17.2, 10.3, 5.5 Hz, 1H), 5.97 (ddd, *J* = 9.1, 4.4, 3.0 Hz, 1H), 5.50 – 5.18 (m, 2H).

**$^{13}$C {1H} NMR** (126 MHz, $CDCl_3$):δ 166.4, 141.6, 136.7, 134.3, 131.6, 128.6, 126.9, 126.8, 126.5, 122.2, 116.1, 51.3, 0.0.

**[α]$_D$**: +58.9 (*c* 1.00, $CH_2Cl_2$).

**HPLC** (Daicel Chiralpak AD-H column, hexane/*i*-PrOH (85:15), flow rate 1.0 mL/min, λ = 240 nm): $t_{major}$ = 9.167 min; $t_{minor}$ = 12.977 min. 87% ee.

**(*R*)-4-Methyl-*N*-(1-phenylallyl)benzenesulfonamide**

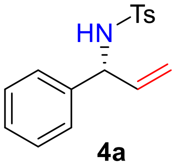


**4a** was prepared according to GP2, using **1a** (34 mg, 0.25 mmol), sulfamic acid **2** (30 mg, 0.30 mmol) and (*R*)-**L1** (57 mg, 0.025mmol) as the ligand, TsCl was used instead of BzCl in step 2. The crude mixture was purified by flash column chromatography (gradient from cyclohexane to 12% EtOAc in cyclohexane) to afford product **4a** (28.1 mg, 39% yield) as a white solid. Known compound, the characterization of the title compound was consistent with the data available in the literature.[30]

**$^1$H NMR** (500 MHz, $CDCl_3$): δ 7.82 – 7.56 (m, 2H), 7.27 – 7.17 (m, 5H), 7.17 – 7.04 (m, 2H), 5.89 (ddd, *J* = 16.9, 10.5, 5.7 Hz, 1H), 5.17 – 5.08 (m, 2H), 5.04 – 4.90 (m, 2H), 2.41 (s, 3H).

**$^{13}$C {1H} NMR** (126 MHz, $CDCl_3$): δ 143.2, 139.4, 137.6, 137.1, 129.4, 128.6, 127.7, 127.2, 127.1, 116.8, 59.8, 21.5.

**[α]$_D$**: +37.2 (*c* 1.10, $CH_2Cl_2$).

## 9. Direct reuse of the whole catalytic system (iridium + immobilized P, olefin ligand).

After a standard run of preparing **3a** according to GP2, the recovered catalytic system Ir- **L4** was subjected to the same reactor, along with addition of sulfamic acid (30 mg, 0.30 mmol, 1.2 eq.) were added sequentially. After 3 vacuum-argon cycles of the reaction system, dimethylformamide (5 eq., 1.25 mmol, 0.1 mL) was added followed by 2-MeTHF (0.6 mL). The resulting mixture was placed in a shaker, and shaked for 15mins during which the color of solution and resin stayed yellow. The substrate **1a** (34 mg, 0.25 mmol, 1.0 eq.) was added as a solution in 2-MeTHF (0.6 mL) to the reaction, and the reaction was allowed to shake at room temperature for 24h. Under argon flow, the reaction was filtered and washed with DCM quickly, the resulting solid was dried under vacuum and kept under argon for reuse in next cycle. The combined organic portion was concentrated under reduced pressure and dissolved in 1 mL DCM, to which was added triethylamine (0.15 mL, 1.0 mmol 4eq.) and benzoyl chloride (55 µL, 0.5 mmol 2eq.), the resulting mixture was stirred at room temperature for 4h. The crude residue was then purified to give the desired ester product in 39% yield and 93% ee.

## 10. General procedure for recycling experiments.

After the first run of preparing **3a** according to GP2, the recovered catalyst of Ir-**L1** or Ir- **L4** was added to a freshly dried screw-capped vial fitted with a rubber septum, $[Ir(COE)_2Cl]_2$ (1.2 mg, 1.05 µmol, 0.005 eq.), and sulfamic acid (30 mg, 0.30 mmol, 1.2 eq.) were added sequentially. After 3 vacuum-argon cycles of the reaction system, dimethylformamide (5 eq., 1.25 mmol, 0.1 mL) was added followed by 2-MeTHF (0.6 mL). The resulting mixture was placed in a shaker, and shaked for 15mins during which the color of solution and resin stayed yellow. The substrate **1a** (34 mg, 0.25 mmol, 1.0 eq.) was added as a solution in 2-MeTHF (0.6 mL) to the reaction, which then turned yellow. The reaction was allowed to shake at room temperature for 24h. Under argon flow, the reaction was filtered and washed with DCM quickly, the resulting solid was dried under vacuum and kept under argon for reuse in next cycle. The combined organic portion was concentrated under reduced pressure and dissolved in 1 mL DCM, to which was added triethylamine (0.15 mL, 1.0 mmol 4eq.) and benzoyl chloride (55 µL, 0.5 mmol 2eq.), the resulting mixture was stirred at room temperature for 4h. The crude residue was then purified by column chromatography on silica gel using cyclohexane/EtOAc (12:1) as the eluent to give the desired ester product. Results are shown in the manuscript.

## 11. References

[1] Yadav, J.; Stanton, G. R.; Fan, X.; Robinson, J. R.; Schelter, E. J.; Walsh, P. J.; Pericàs, M. A. Asymmetric Allylation of Ketones and Subsequent Tandem Reactions Catalyzed by a Novel Polymer-Supported Titanium–BINOLate Complex. *Chem. Eur. J.* **2014**, *20*, 7122-7127.

[2] Herrera, A.; Linden, A.; Heinemann, F. W.; Brachvogel, R. C.; von Delius, M.; Dorta, R. Optimized Syntheses of Optically Pure P-Alkene Ligands: Crystal Structures of a Pair of P-Stereogenic Diastereomers. *Synthesis* **2016**, *48*, 1117-1121.

[3] Lai, J.; Fianchini, M.; Pericàs, M. A. Development of Immobilized SPINOL-Derived Chiral Phosphoric Acids for Catalytic Continuous Flow Processes. Use in the Catalytic Desymmetrization of 3, 3-Disubstituted Oxetanes. *ACS Catal.* **2020**, *10*, 14971-14983.

[4] Osorio-Planes, L.; Rodríguez-Escrich, C.; Pericàs, M. A. Enantioselective Continuous-Flow Production of 3-Indolylmethanamines Mediated by an Immobilized Phosphoric Acid Catalyst. *Chem. Eur. J.* **2014**, *20*, 2367-2372.

[5] D. Jayaprakash, H. Sasai, Synthesis and catalytic applications of soluble polymer-supported BINOL. *Tetrahedron: Asymmetry* **2001**, *12*, 2589.

[6] Bischofberger, N.; Waldmann, H.; Saito, T.; Simon, E.; Lees, W.; Bednarski, M.; Whitesides, G. *J. Org. Chem*., **1988**, *53*, 3457-3465.

[7] Marion N.; Gealageas R.; Nolan S. P. [(NHC)$Au^{I}$]-Catalyzed Rearrangement of Allylic Acetates *Org. Lett.,* **2007**, *9*, 2653-2656.

[8] Lin, H.; Liu, Y.; Wu, Z.-L. Highly diastereo- and enantio-selective epoxidation of secondary allylic alcohols catalyzed by styrene monooxygenase *Chem. Commun*., **2011**, *47*, 2610-2612.

[9] Batory, L. A.; McInnis, C. E.; Njardarson, J. T. Copper-Catalyzed Rearrangement of Vinyl Oxiranes *J. Am. Chem. Soc*., **2006**, *128*, 16054.

[10] Morrill, C.; Grubbs, R. H. Highly Selective 1,3-Isomerization of Allylic Alcohols via Rhenium Oxo Catalysis J*. Am. Chem. Soc*., **200**5, *127*, 2842-2843.

[11] Fernandes, R. A.; Kattanguru, P. Tandem benzylic oxidation/dihydroxylation ofa-vinyl-anda-alkenylbenzyl alcohols *Hel. Chim. A.*, **2015**, *98*, 92-107.

[12] Logan, A. W. J.; Parker, J. S.; Hallside, M. S.; Burton, J. W., Manganese(III) Acetate Mediated Oxidative Radical Cyclizations. Toward Vicinal All-Carbon Quaternary Stereocenters. *Org. Lett.* **2012**, *14*, 2940-2943.

[13] Latham, D. E.; Polidano, K.; Williams, J. M. J.; Morrill, L. C., One-Pot Conversion of Allylic Alcohols to α-Methyl Ketones via Iron-Catalyzed Isomerization–Methylation. *Org. Lett.* **2019,** *21*, 7914-7918.

[14] George, S.; Narina, S.V.; Sudalai, A. A short enantioselective synthesis of (−)-chloramphenicol and (+)-thiamphenicol using tethered aminohydroxylation *Tetrahedron*, **2006**, *62*, 10202-10207.

[15] Kikuchi, K.; Hibi, S.; Yoshimura, H.; Tokuhara, N.; Tai, K.; Hida, T.; Yamauchi, T.; Nagai, M. Syntheses and Structure−Activity Relationships of 5,6,7,8-Tetrahydro-5,5,8,8-tetramethyl-2-quinoxaline Derivatives with Retinoic Acid Receptor α Agonistic Activity *J. Med. Chem.*, **2000**, *43*, 409-419.

[16] Gan, Y.; Hu, H.; Liu, Y. Nickel-Catalyzed Homo- and Cross-Coupling of Allyl Alcohols via Allyl Boronates *Org. Lett.* **2020**, *22*, 4418–4423.

[17] Musacchio A. J.; Nguyen, L. Q.; Beard, G. H.; Knowles, R. R. Catalytic Olefin Hydroamination with Aminium Radical Cations: A Photoredox Method for Direct C–N Bond Formation *J. Am. Chem. Soc.* **2014**, *136*, 12217–12220.

[18] Briot, A.; Baehr, C.; Brouillard, R.; Wagner, A.; Mioskowski, C. Concise Synthesis of Dihydrochalcones via Palladium-Catalyzed Coupling of Aryl Halides and 1-Aryl-2-propen-1-ols *J. Org. Chem*., **2004**, *69*, 1374-1377.

[19] Delaye, P.-O.; Vasse, J.-L.; Szymoniak, J. Asymmetric synthesis of proline-based conformationally constrained tryptophan mimetic *Org. Biomol. Chem.* **2010**, *16*, 3635-3637.

[20] Burgess. K.; Jennings, L. D. Enantioselective Esterifications of Unsaturated Alcohols Mediated by a Lipase Prepared from Pseudomonas sp. *J. Am. Chem. Soc.*, **1991**, *113*, 6129-6139.

[21] Bartels, B.; Garcia-Yebra, C.; Rominger, F.; Helmchen, G. Iridium-Catalysed Allylic Substitution: Stereochemical Aspects and Isolation of $Ir^{III}$ Complexes Related to the Catalytic Cycle *Eur. J. Inorg. Chem*., **2002**, *10*, 2569-2587.

[22] Grünanger, C. U.; Breit, B. Remote Control of Regio- and Diastereoselectivity in the Hydroformylation of Bishomoallylic Alcohols with Catalytic Amounts of a Reversibly Bound Directing Group *Angew. Chem., Int. Ed.*, **2010**, *49*, 967-970.

[23] Lin, H.; Liu, Y.; Wu, Z.-L. Highly diastereo- and enantio-selective epoxidation of secondary allylic alcohols catalyzed by styrene monooxygenase *Chem. Commun.*, **2011**, *47*, 2610-2612

[24] Kirmse, W.; Rode, K. Desaminierungsreaktionen, 46 Zerfall von 1-Alkenylcyclopropandiazonium-Ionen. *Chem. Ber.* **1987**, *120*, 839.

[25] Lin, H.; Liu, Y.; Wu, Z.-L. Trost, B.M.; Fraisse, P.L.; Ball, Z. A Stereospecific Ruthenium-Catalyzed Allylic Alkylation *Angew. Chem., Int. Ed.*, **2002**, *41*, 1059-1061.

[26] Castagnolo, D.; Armaroli, S.; Corelli, F.; Botta, M. Enantioselective synthesis of 1-aryl-2-propenylamines: a new approach to a stereoselective synthesis of the Taxol® side chain *Tetrahedron*, **2004**, *15*, 941-949.

[27] Roggen M.; Carreira E. M. Stereospecific substitution of allylic alcohols to give optically active primary allylic amines: unique reactivity of a (P,alkene)Ir complex modulated by Iodide *J. Am. Chem. Soc.* **2010**, *132*, 11917–11919.

[28] Lafrance M.; Roggen M.; Carreira E. M. Direct, Enantioselective iridium-catalyzed allylic amination of racemic allylic alcohols *Angew. Chem. Int. Ed.* **2012**, *51*, 3470 –3473

[29] Almansa R.; Collados J. F.; Guijarro D.; Yus M. Asymmetric synthesis of α- and β-amino acids by diastereoselective addition of triorganozincates to N-(tert-butanesulfinyl)imines *Tetrahedron* **2010**, *21*, 1421-1431.

[30] Shea, R.G.; Fitzner, J.N.; Frankhauser, J.E.; Spaltenstein, A.; Carpino, P.A.; Peevey. R.M.; Pratt, D.V.; Tenge, B.J.; Hopkins, P.B. Allylic selenides in organic synthesis: new methods for the synthesis of allylic amines *J. Org. Chem*., **1986**, *51*, 5243-5252.

[31] Meng, C. Y.; Liang, X.; Wei, K.; Yang, Y. R. Enantioselective Ir-Catalyzed Allylic Alkylation of Racemic Allylic Alcohols with Malonates. *Org. Lett.* **2019**, *21*, 840-843.

## 12. NMR Spectra

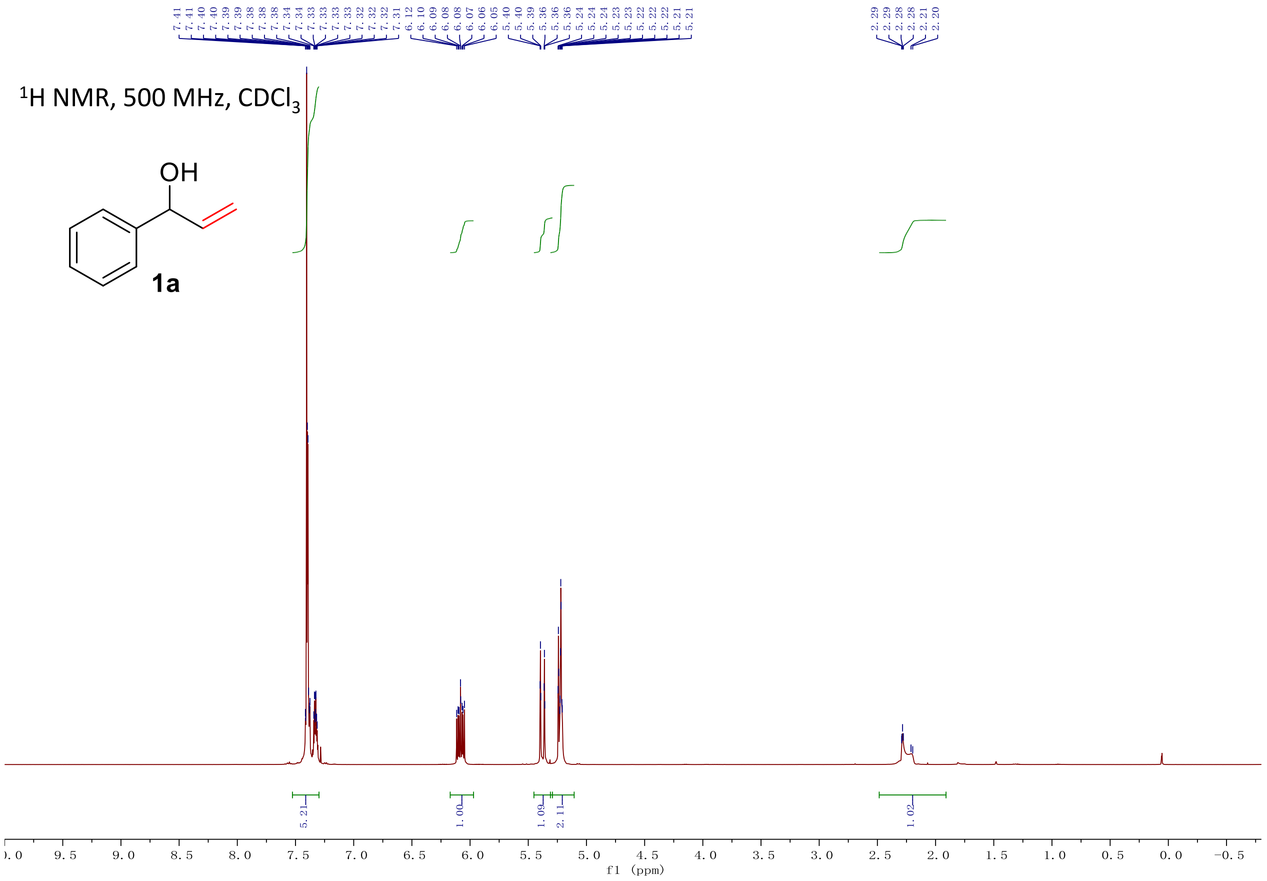


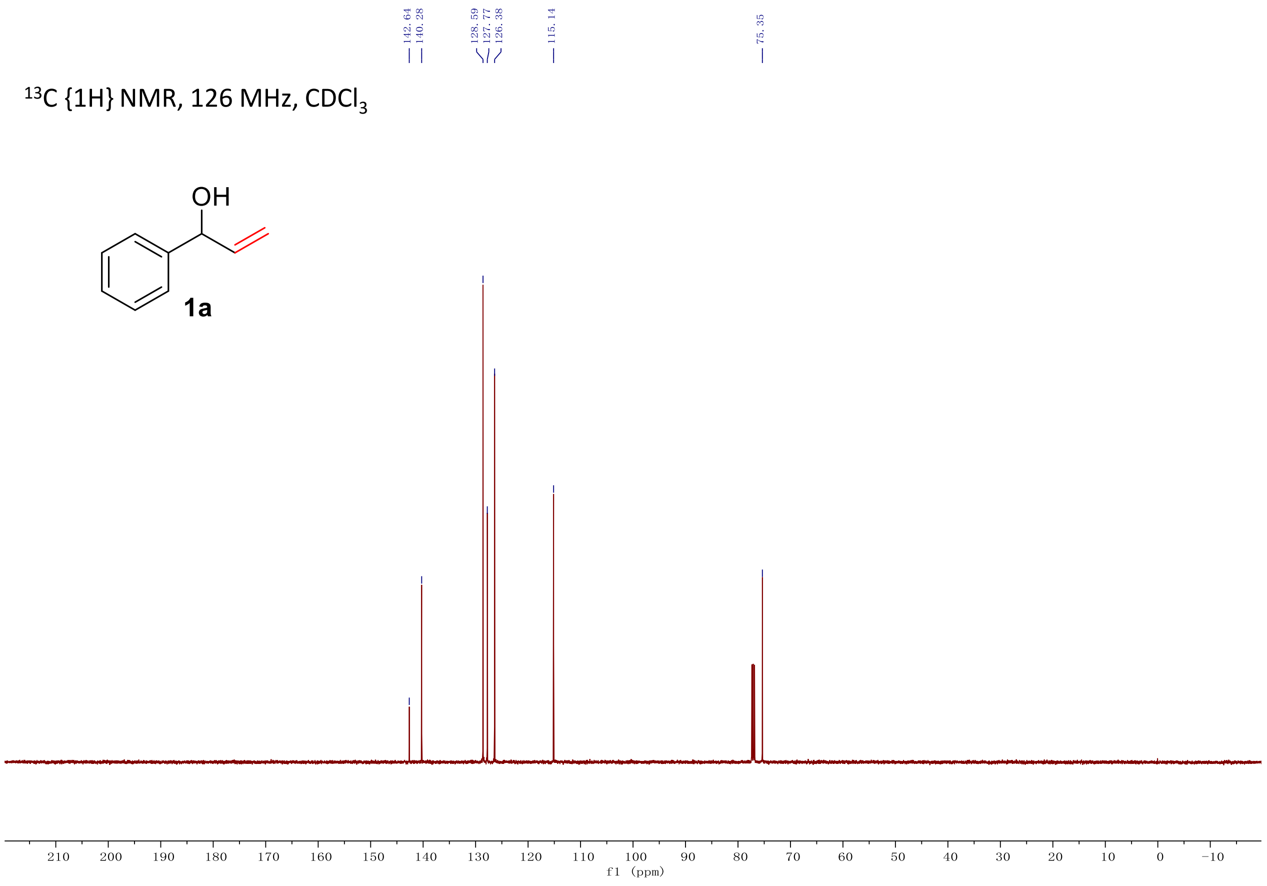

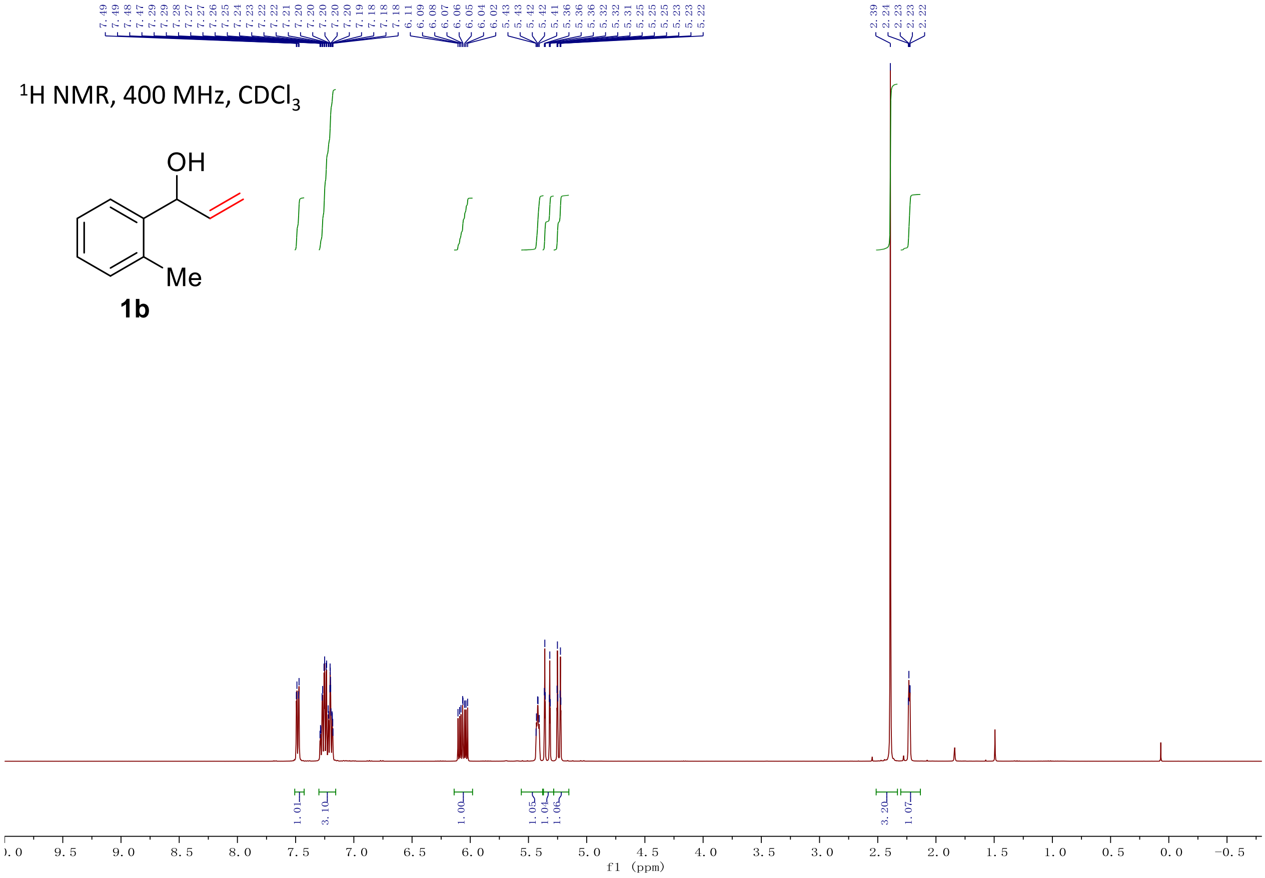
1H NMR, 400 MHz, CDCl3
OH
Me
1b
7.49 7.49 7.48 7.47 7.29 7.28 7.27 7.27 7.26 7.25 7.24 7.23 7.22 7.22 7.21 7.20 7.20 7.20 7.20 7.20 7.19 7.18 7.18 7.18 6.11 6.09 6.08 6.07 6.06 6.05 6.04 6.02 5.43 5.43 5.42 5.42 5.41 5.36 5.36 5.32 5.32 5.31 5.25 5.25 5.23 5.23 5.22
2.39 2.24 2.23 2.23 2.22
1.01 3.10 1.00 1.05 1.04 1.06 3.20 1.07
f1 (ppm)

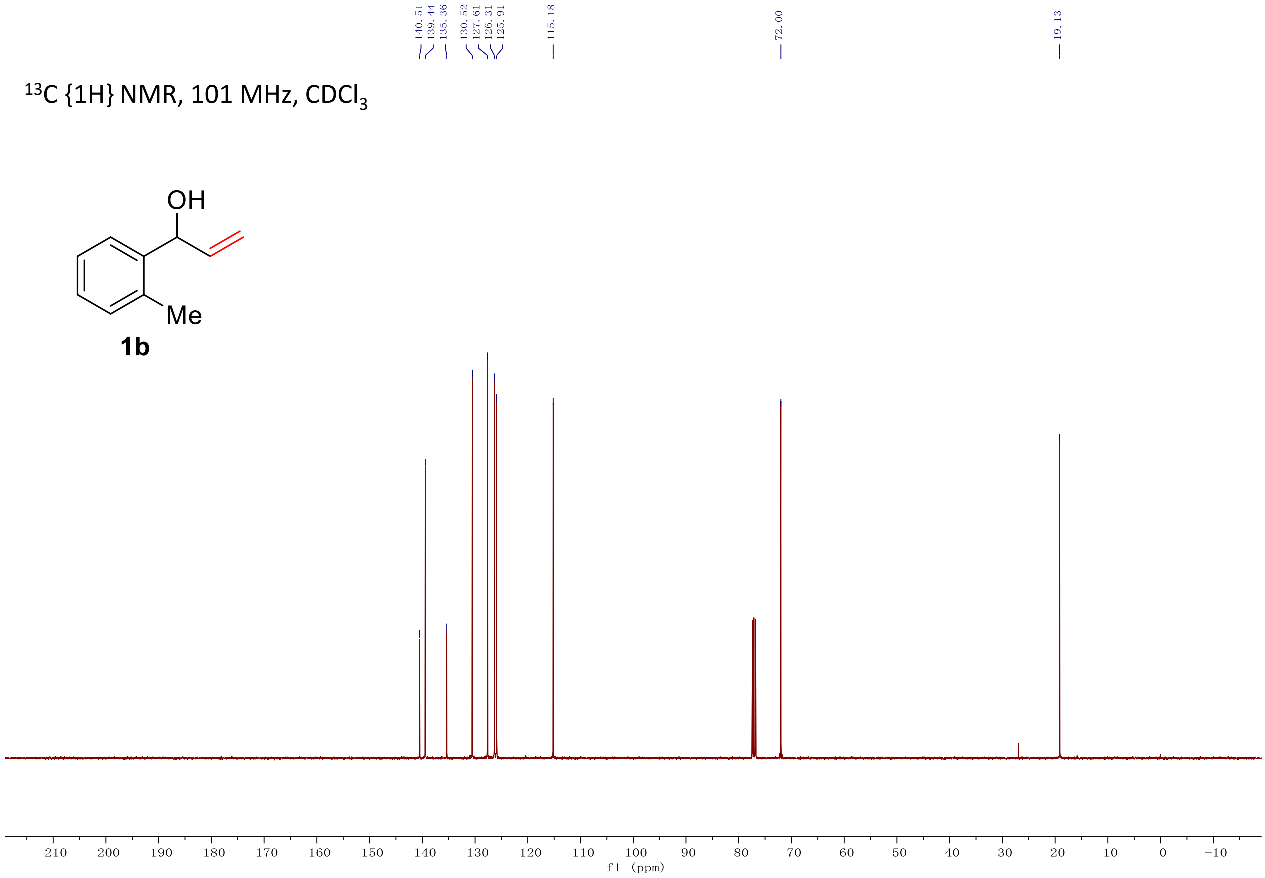
13C {1H} NMR, 101 MHz, CDCl3
OH
Me
1b
140.51 139.44 135.36 130.52 127.61 126.31 125.91 115.18 72.00 19.13
f1 (ppm)

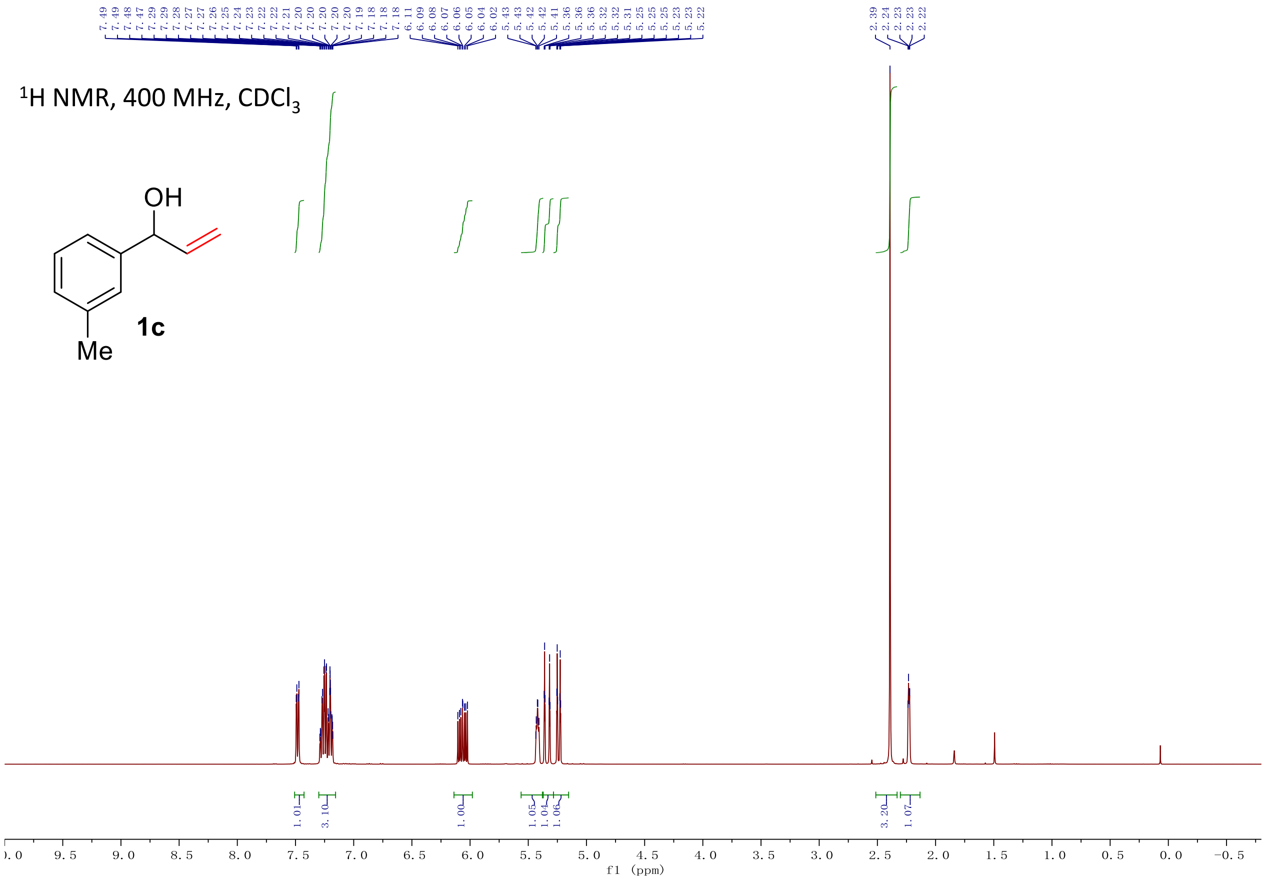
7.49 7.49 7.48 7.47 7.29 7.29 7.28 7.27 7.27 7.26 7.25 7.24 7.23 7.22 7.22 7.21 7.20 7.20 7.20 7.20 7.20 7.19 7.18 7.18 6.11 6.09 6.08 6.07 6.06 6.05 6.04 6.02 5.43 5.43 5.42 5.41 5.36 5.36 5.32 5.32 5.31 5.25 5.25 5.23 5.23 5.22
2.39 2.24 2.23 2.23 2.22
$^{1}$H NMR, 400 MHz, $CDCl_3$
OH
Me
1c
1.01 3.10 1.00 1.05 1.04 1.06 3.20 1.07
f1 (ppm)

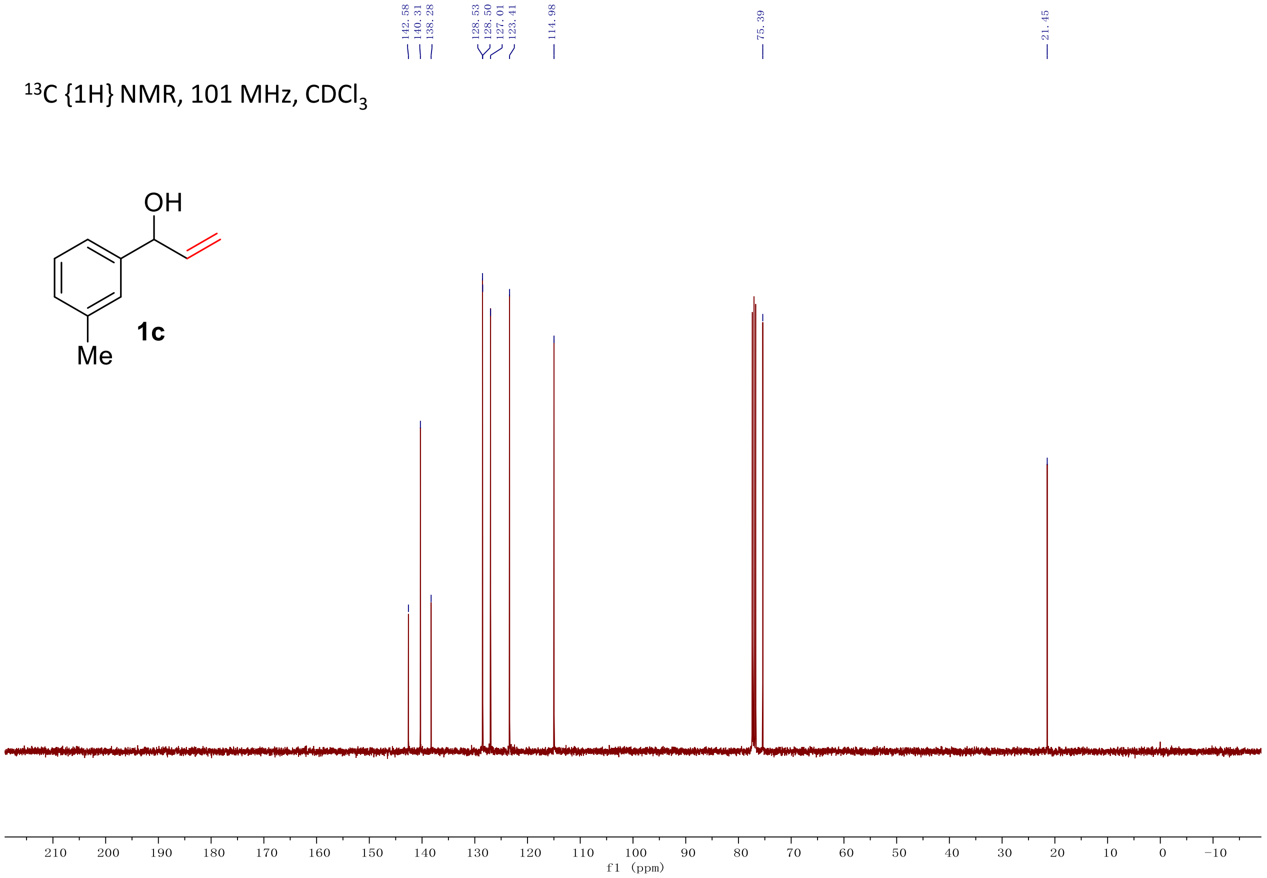
142.58 140.31 138.28 128.53 128.50 127.01 123.41 114.98 75.39 21.45
$^{13}$C {1H} NMR, 101 MHz, $CDCl_3$
OH
Me
1c
f1 (ppm)

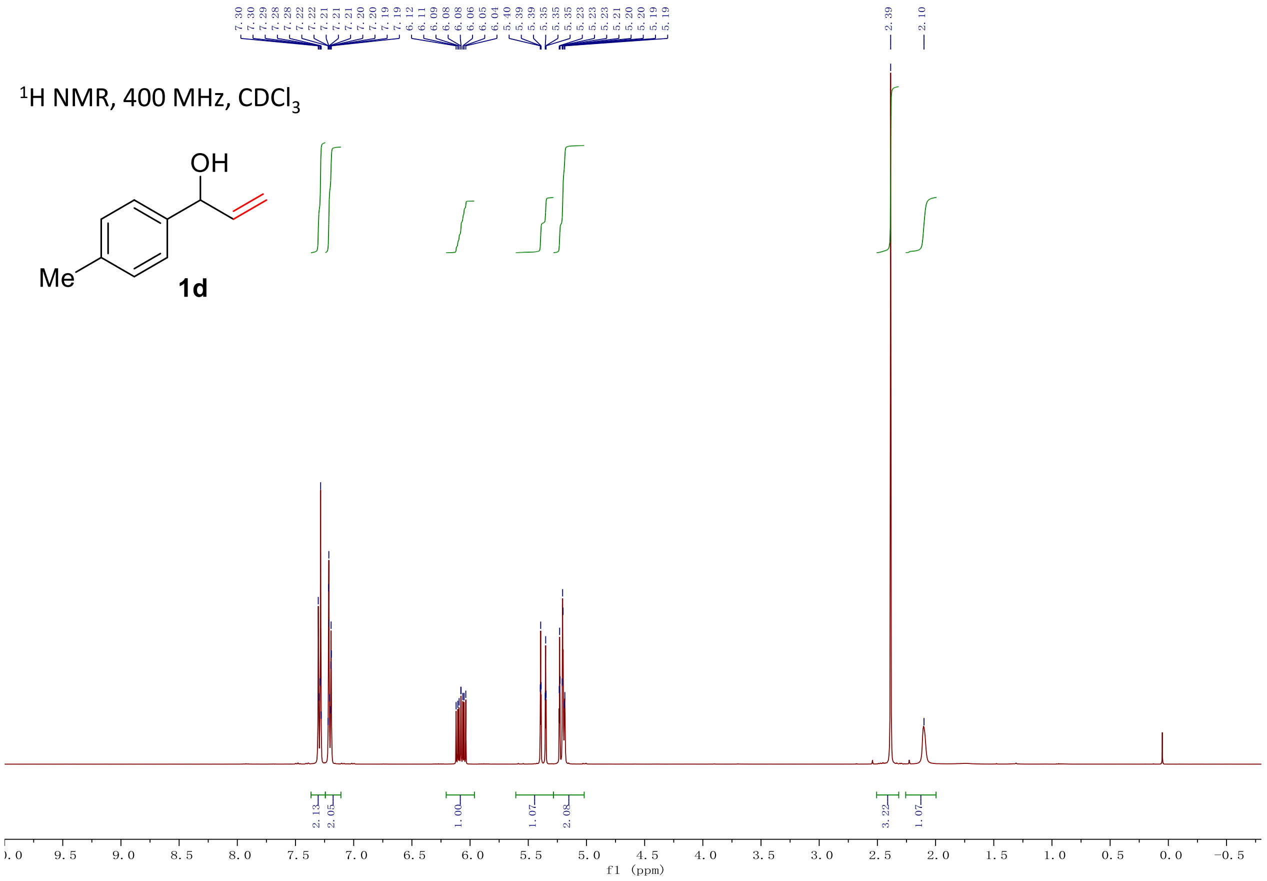
1H NMR, 400 MHz, CDCl3
OH
Me
1d
7.30 7.30 7.29 7.28 7.28 7.22 7.22 7.21 7.21 7.21 7.20 7.20 7.19 7.19 6.12 6.11 6.09 6.08 6.08 6.06 6.05 6.04 5.40 5.39 5.39 5.35 5.35 5.35 5.23 5.23 5.23 5.21 5.20 5.20 5.19 5.19
2.39
2.10
2.13 2.05 1.00 1.07 2.08 3.22 1.07
f1 (ppm)

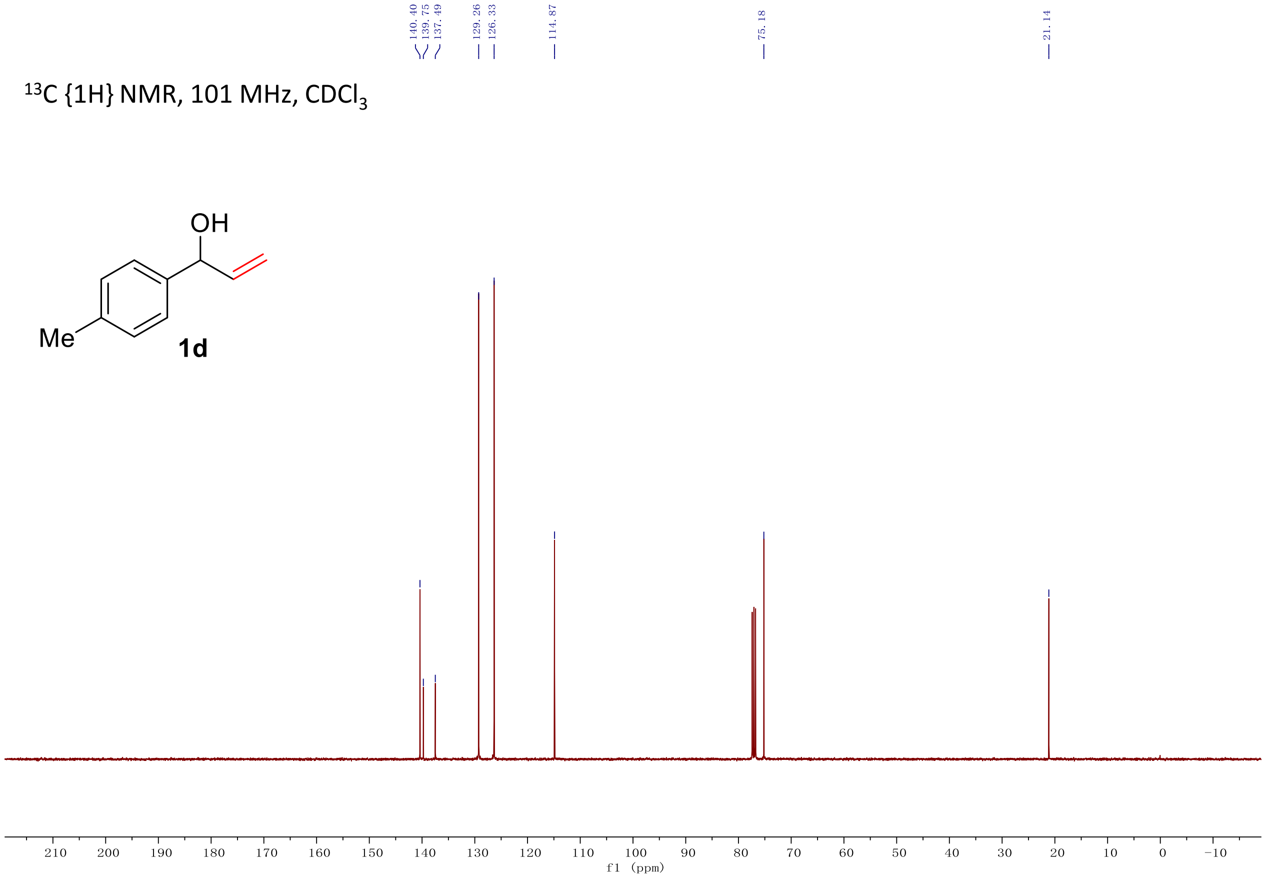
13C {1H} NMR, 101 MHz, CDCl3
OH
Me
1d
140.40 139.75 137.49
129.26 126.33
114.87
75.18
21.14
f1 (ppm)

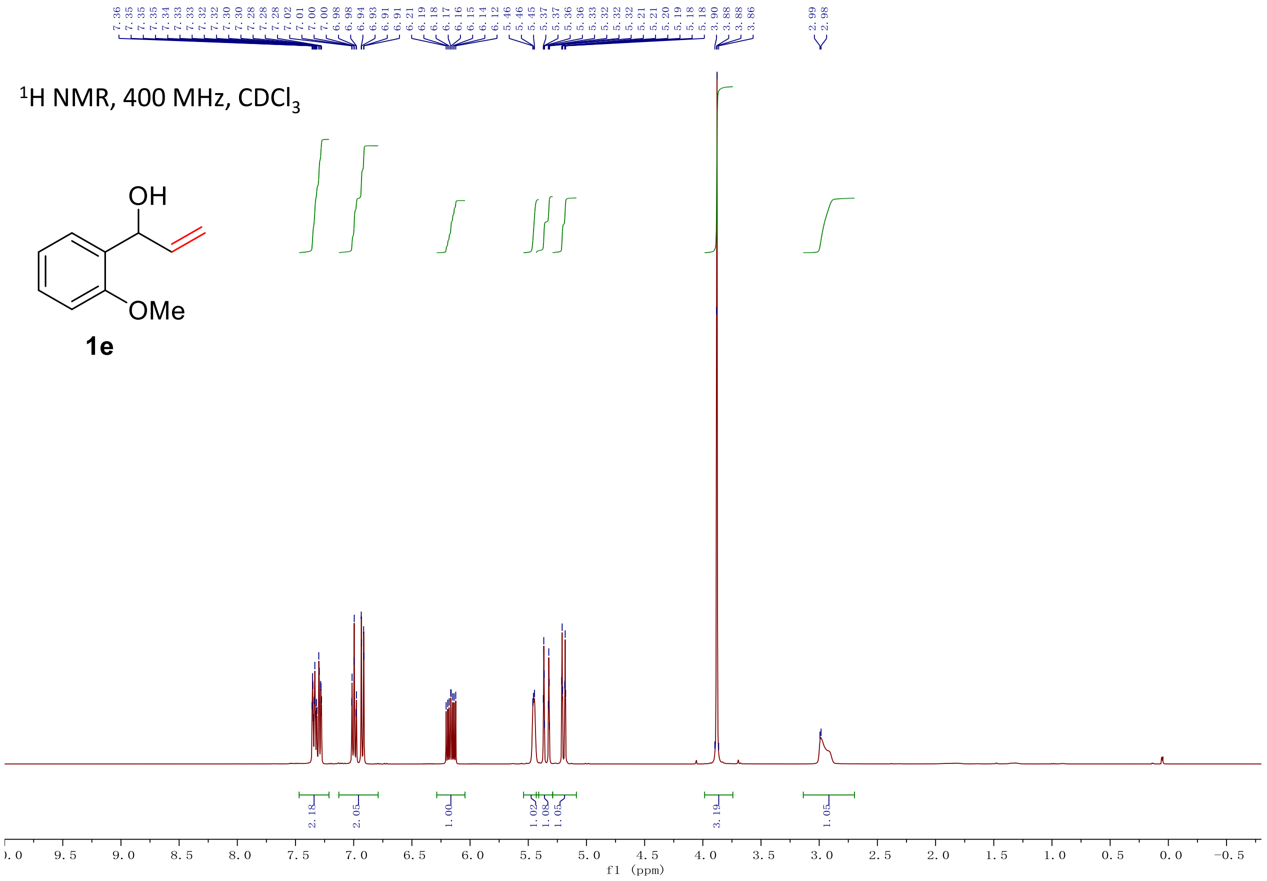
7.36 7.35 7.35 7.34 7.33 7.33 7.32 7.32 7.30 7.30 7.28 7.28 7.28 7.02 7.01 7.00 7.00 6.98 6.94 6.93 6.91 6.91 6.21 6.19 6.18 6.17 6.16 6.15 6.14 6.12 5.46 5.45 5.37 5.37 5.36 5.33 5.32 5.32 5.21 5.21 5.20 5.19 5.18 3.90 3.88 3.86 2.99 2.98
$^1$H NMR, 400 MHz, CDCl$_3$
OH
OMe
1e
2.18 2.05 1.00 1.02 1.08 1.05 3.19 1.05
f1 (ppm)

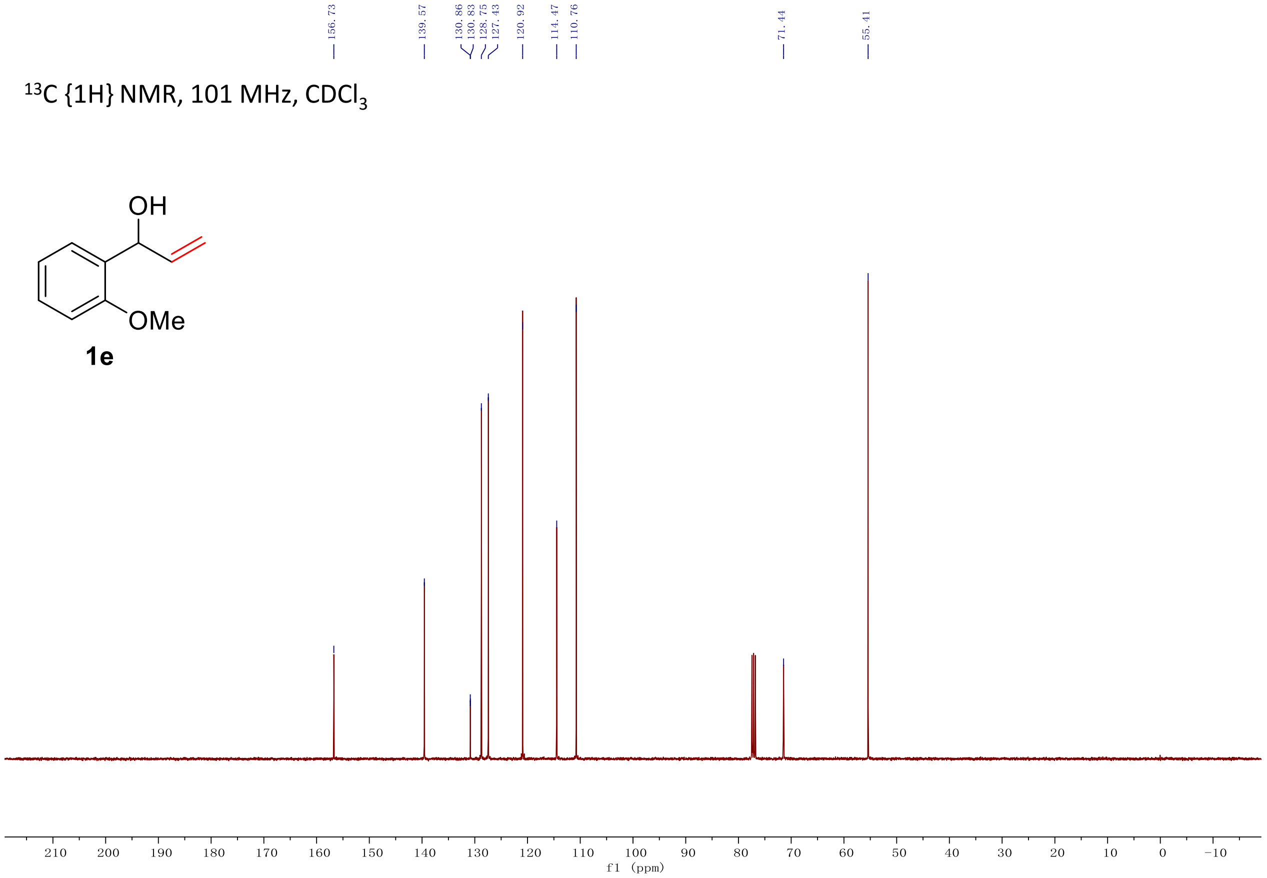
156.73 139.57 130.86 130.83 128.75 127.43 120.92 114.47 110.76 71.44 55.41
$^{13}$C {1H} NMR, 101 MHz, CDCl$_3$
OH
OMe
1e
f1 (ppm)

$^{1}$H NMR, 400 MHz, $CDCl_3$

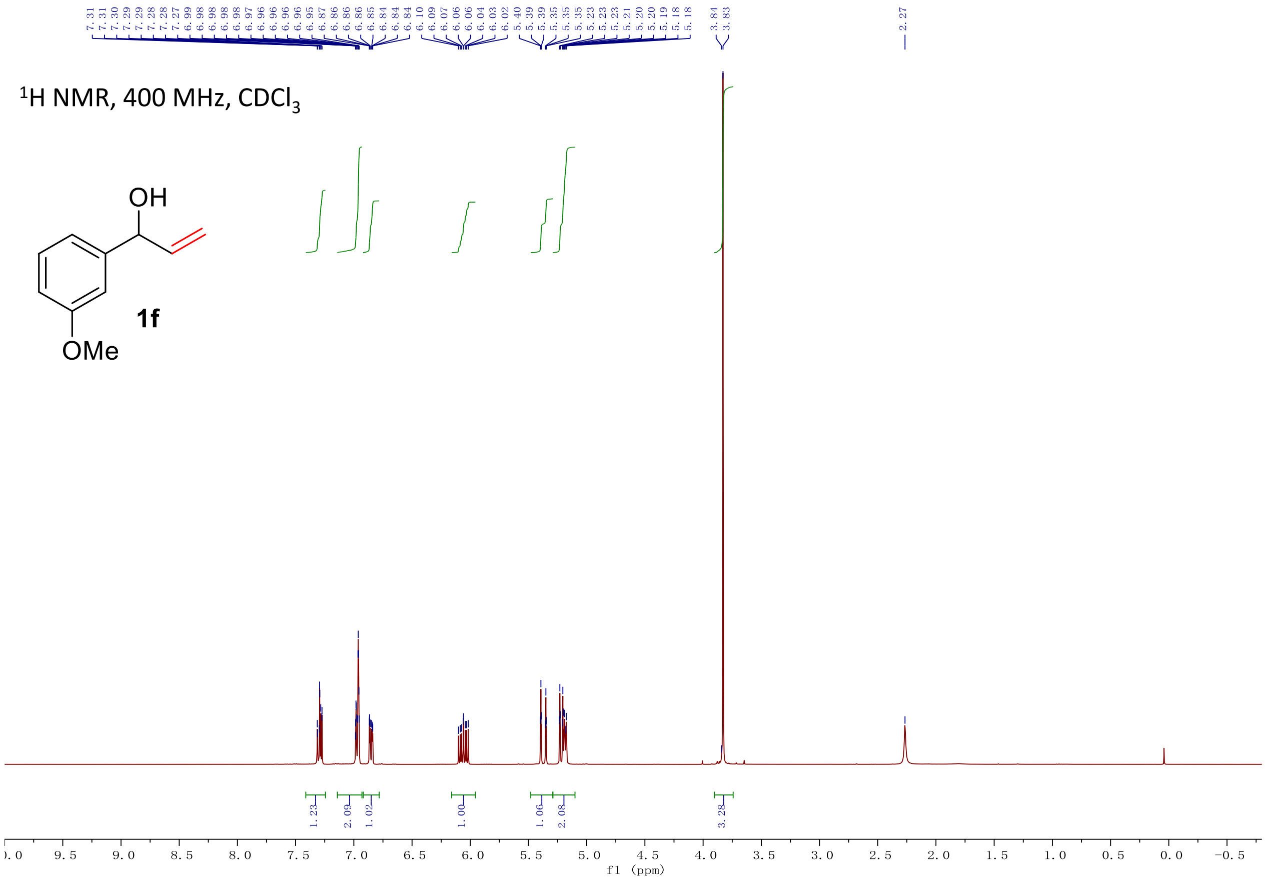


$^{13}$C {1H} NMR, 101 MHz, $CDCl_3$

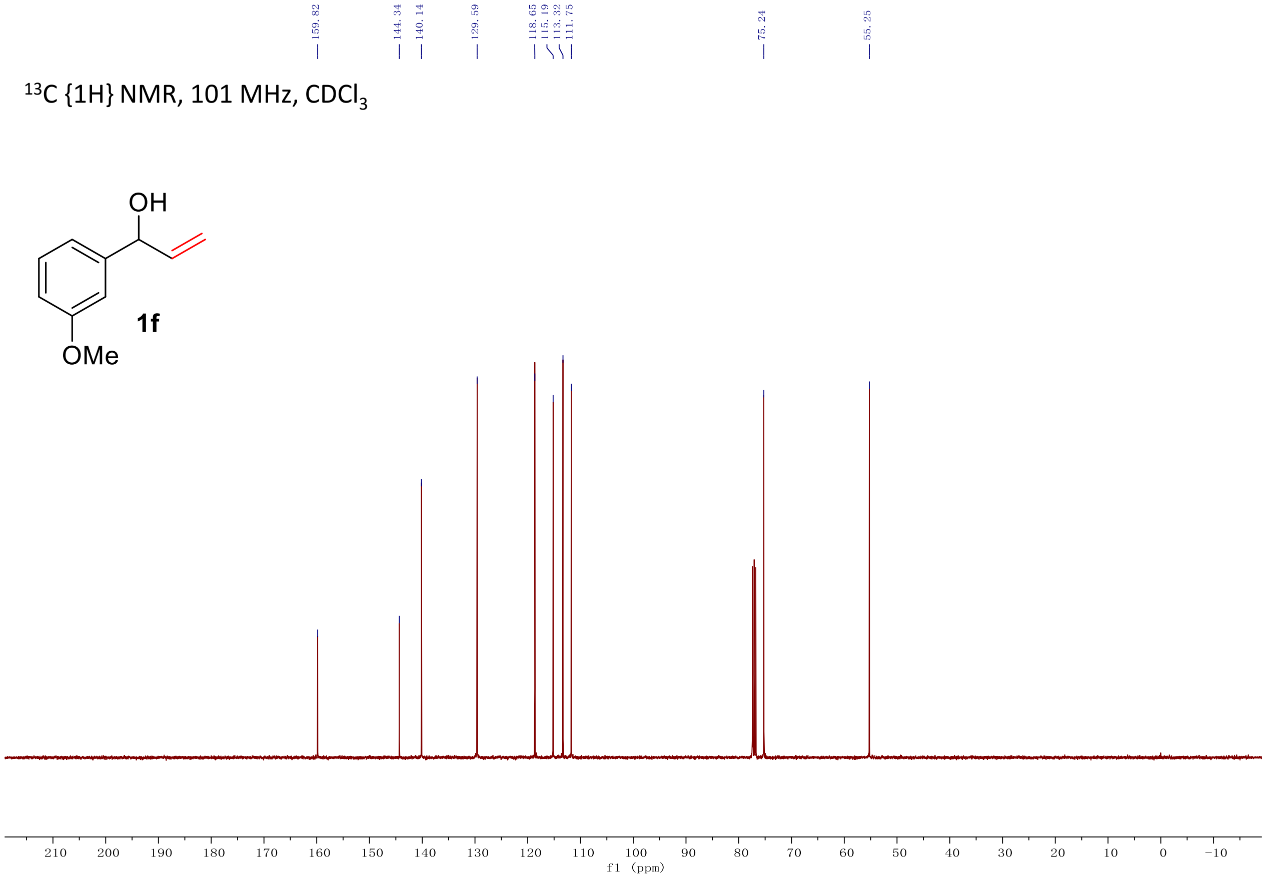

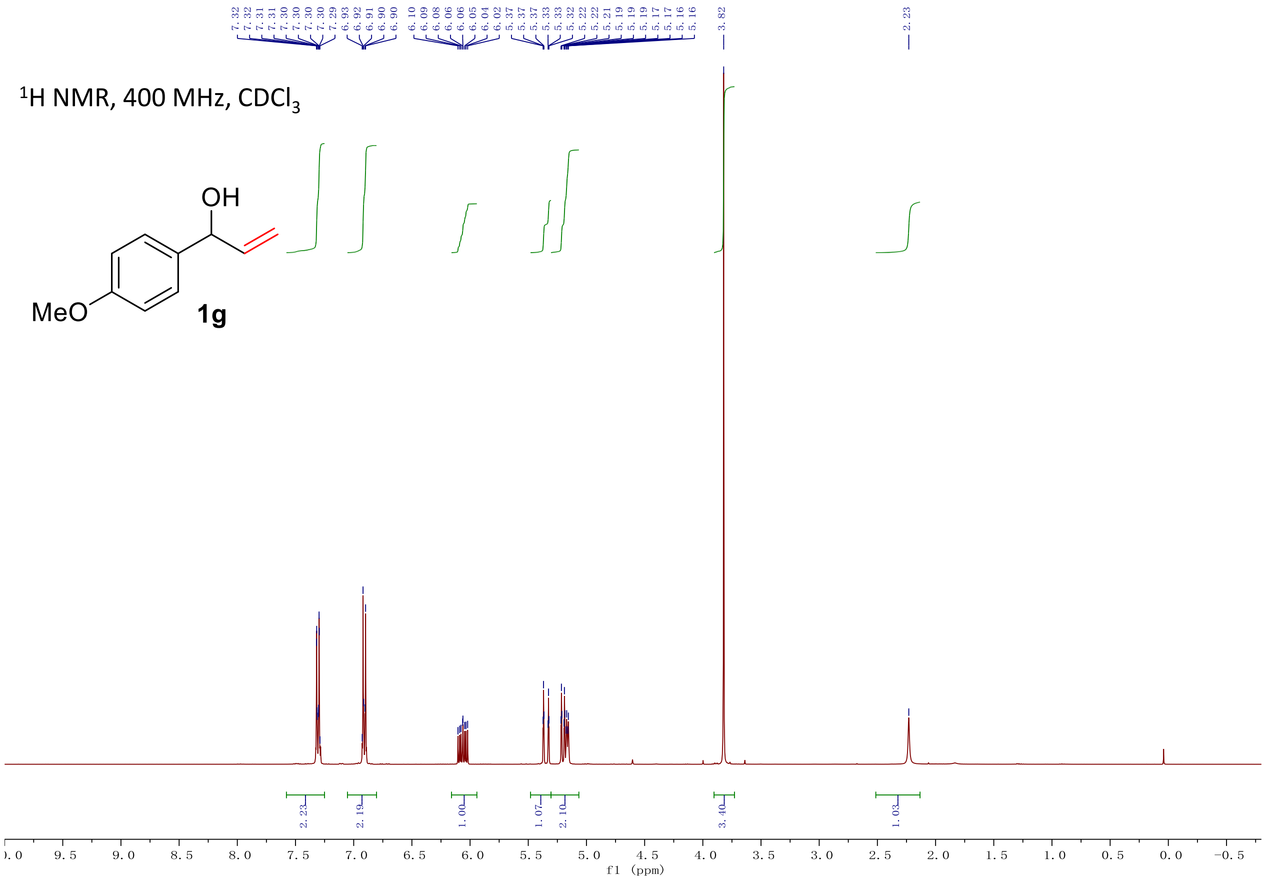

1H NMR, 400 MHz, CDCl3
OH
MeO
1g
f1 (ppm)


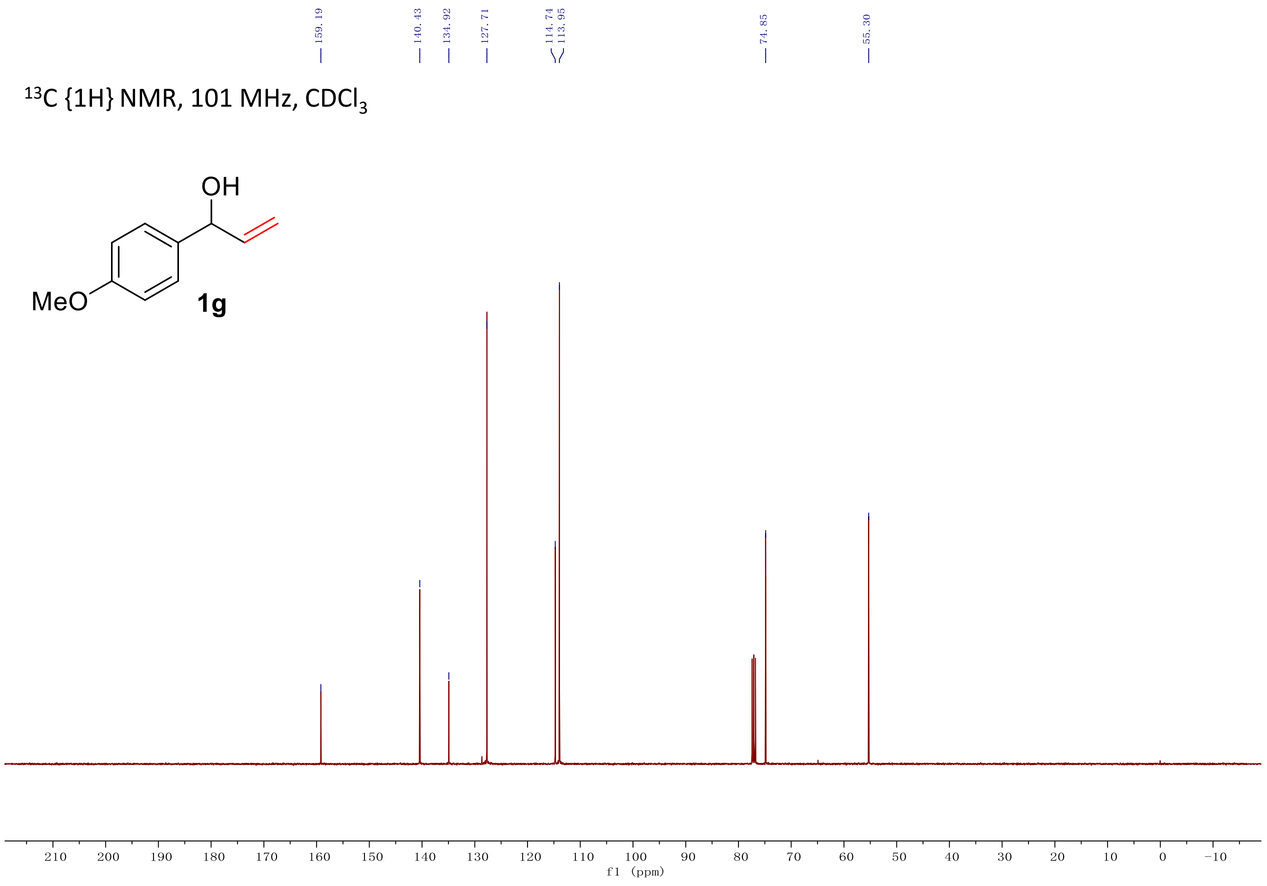

13C {1H} NMR, 101 MHz, CDCl3
159.19
140.43
134.92
127.71
114.74
113.95
74.85
55.30
OH
MeO
1g
f1 (ppm)

$^{1}H$ NMR, 400 MHz, $CDCl_3$

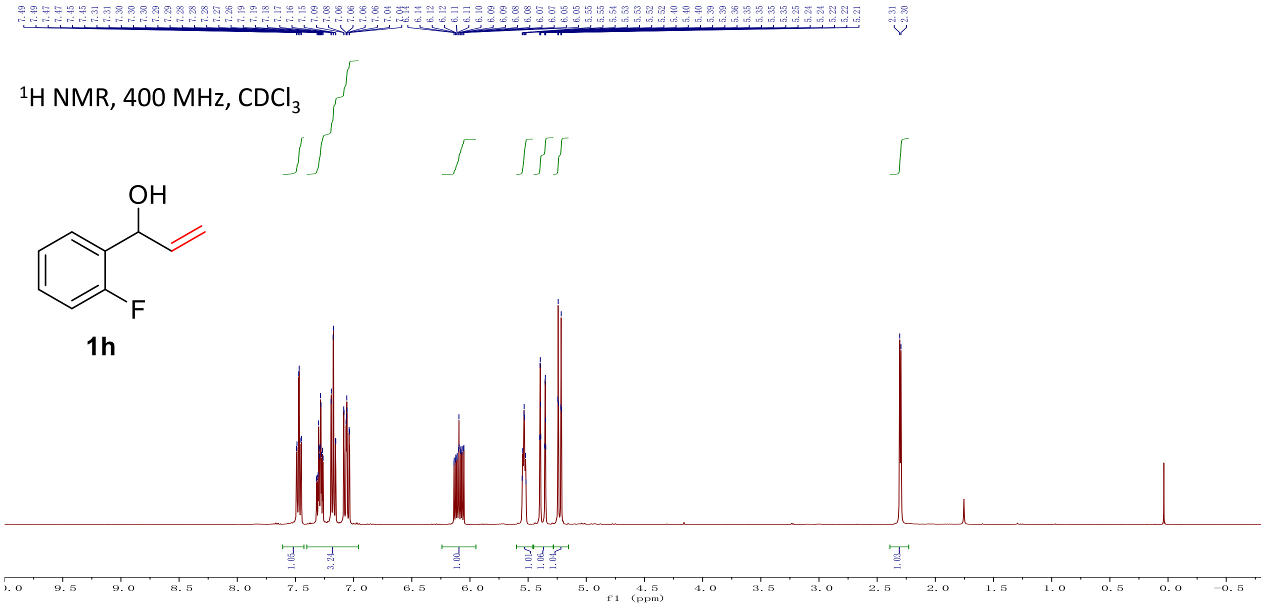


$^{13}C$ {1H} NMR, 101 MHz, $CDCl_3$

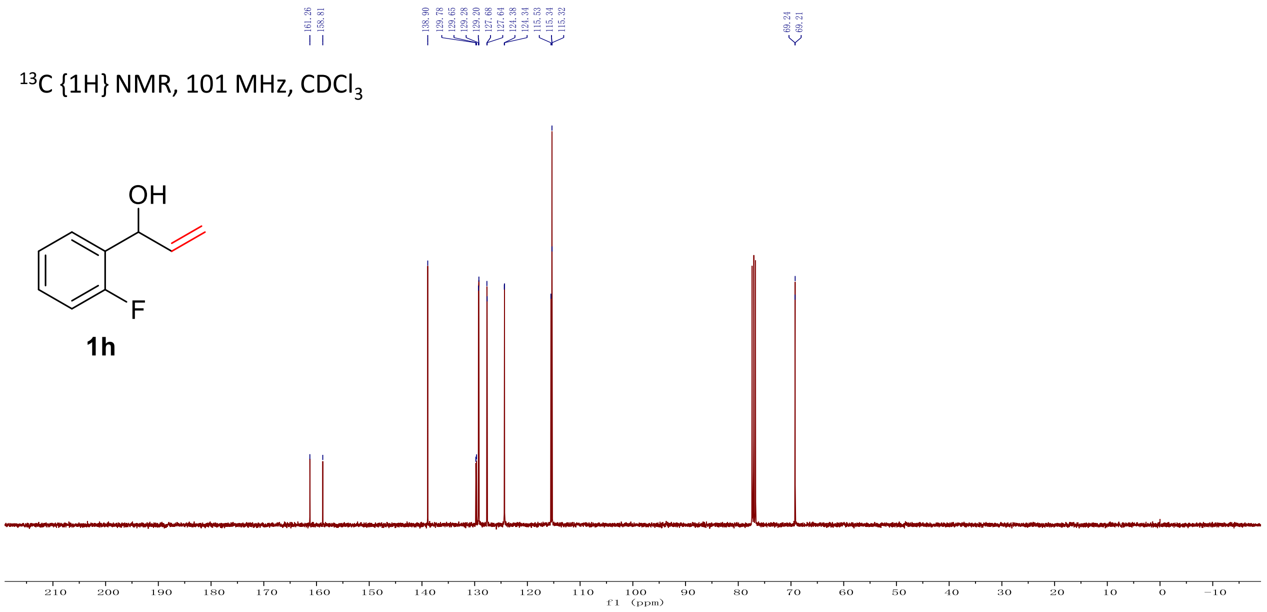


$^{13}C$ {1H} NMR, 101 MHz, $CDCl_3$

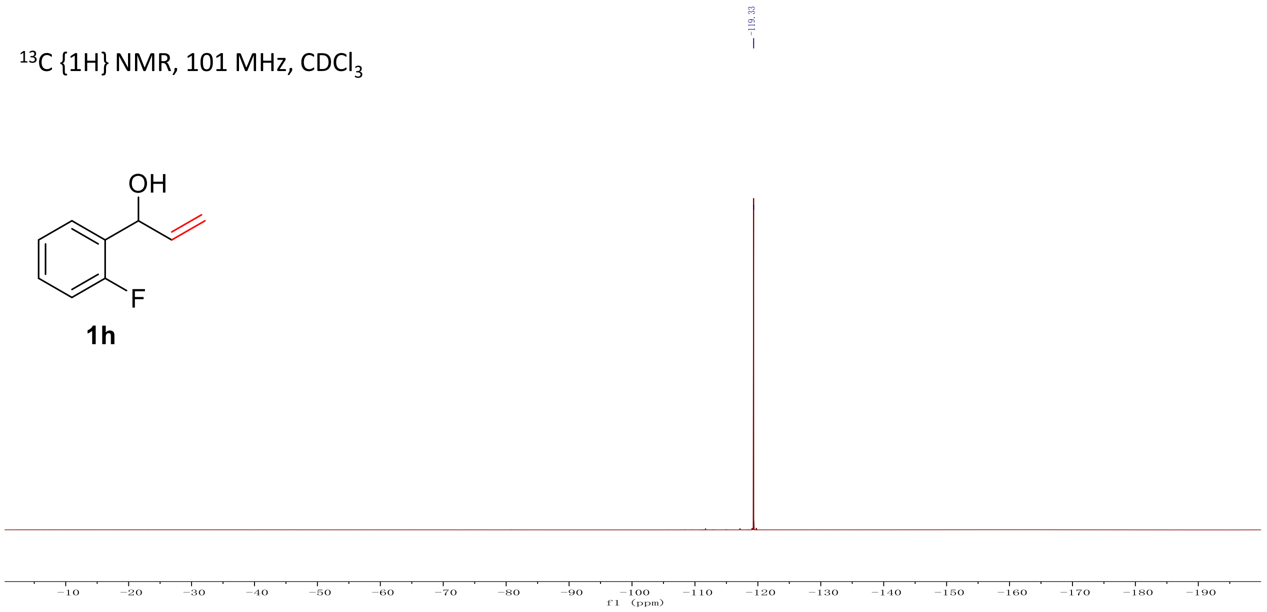

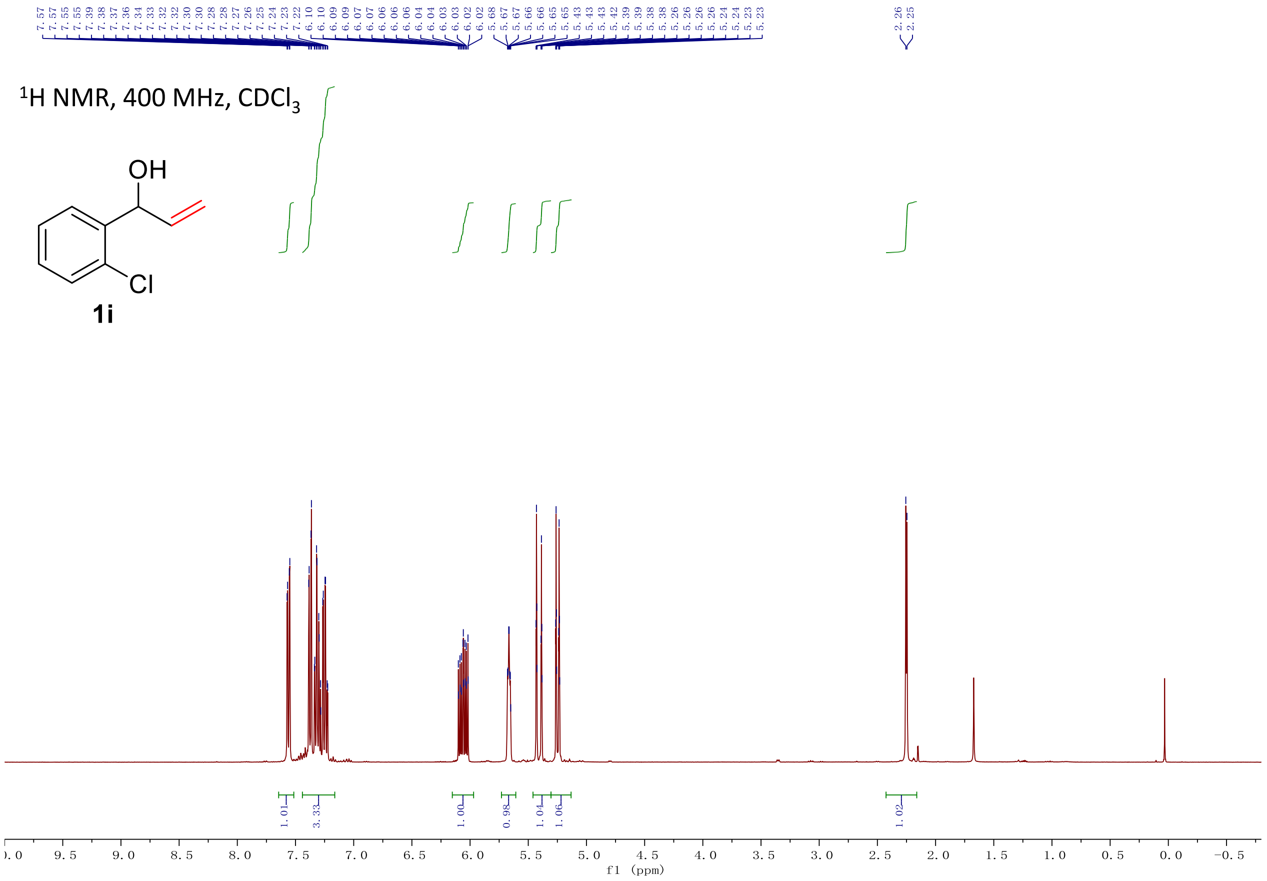
$^1$H NMR, 400 MHz, CDCl$_3$
OH
Cl
1i
1.01
3.33
1.00
0.98
1.04
1.06
1.02
f1 (ppm)

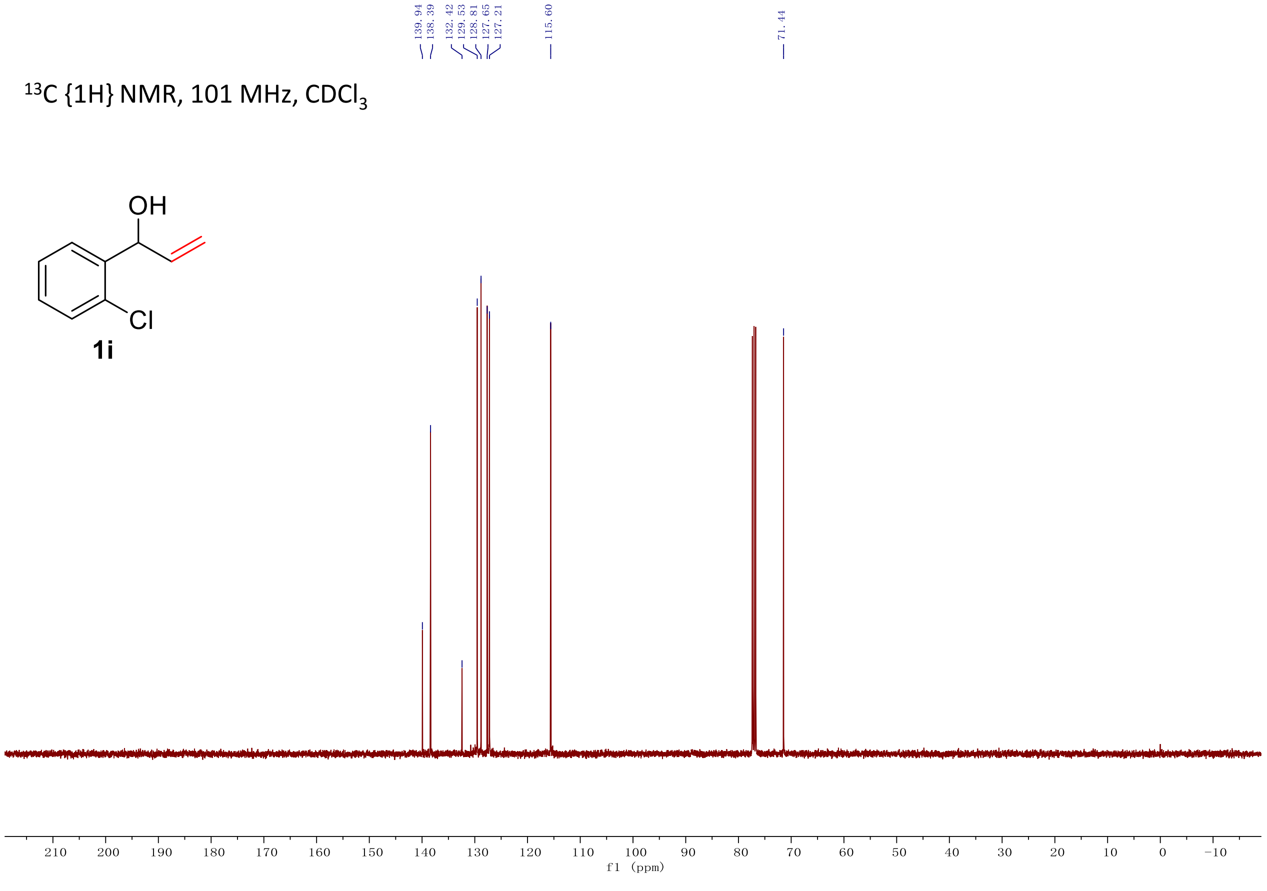
139.94
138.39
132.42
129.53
128.81
127.65
127.21
115.60
71.44
$^{13}$C {1H} NMR, 101 MHz, CDCl$_3$
OH
Cl
1i
f1 (ppm)

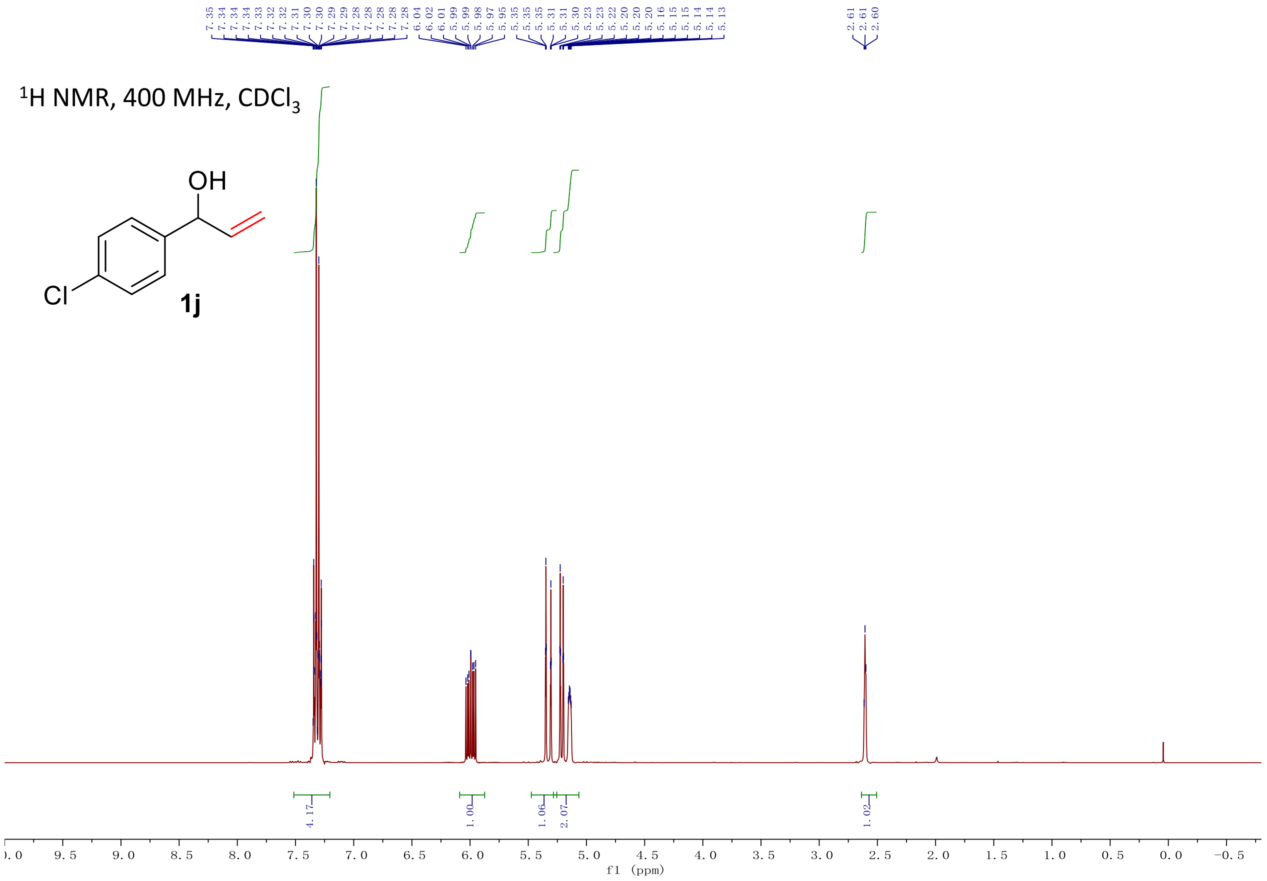

7.35 7.34 7.34 7.33 7.32 7.32 7.31 7.30 7.30 7.29 7.29 7.28 7.28 7.28 7.28 7.28 6.04 6.02 6.01 5.99 5.99 5.98 5.97 5.95 5.35 5.35 5.35 5.31 5.31 5.30 5.23 5.23 5.22 5.20 5.20 5.20 5.16 5.15 5.15 5.14 5.14 5.13
2.61 2.61 2.60
¹H NMR, 400 MHz, CDCl₃
OH
Cl
1j
4.17
1.00
1.06
2.07
1.02
f1 (ppm)


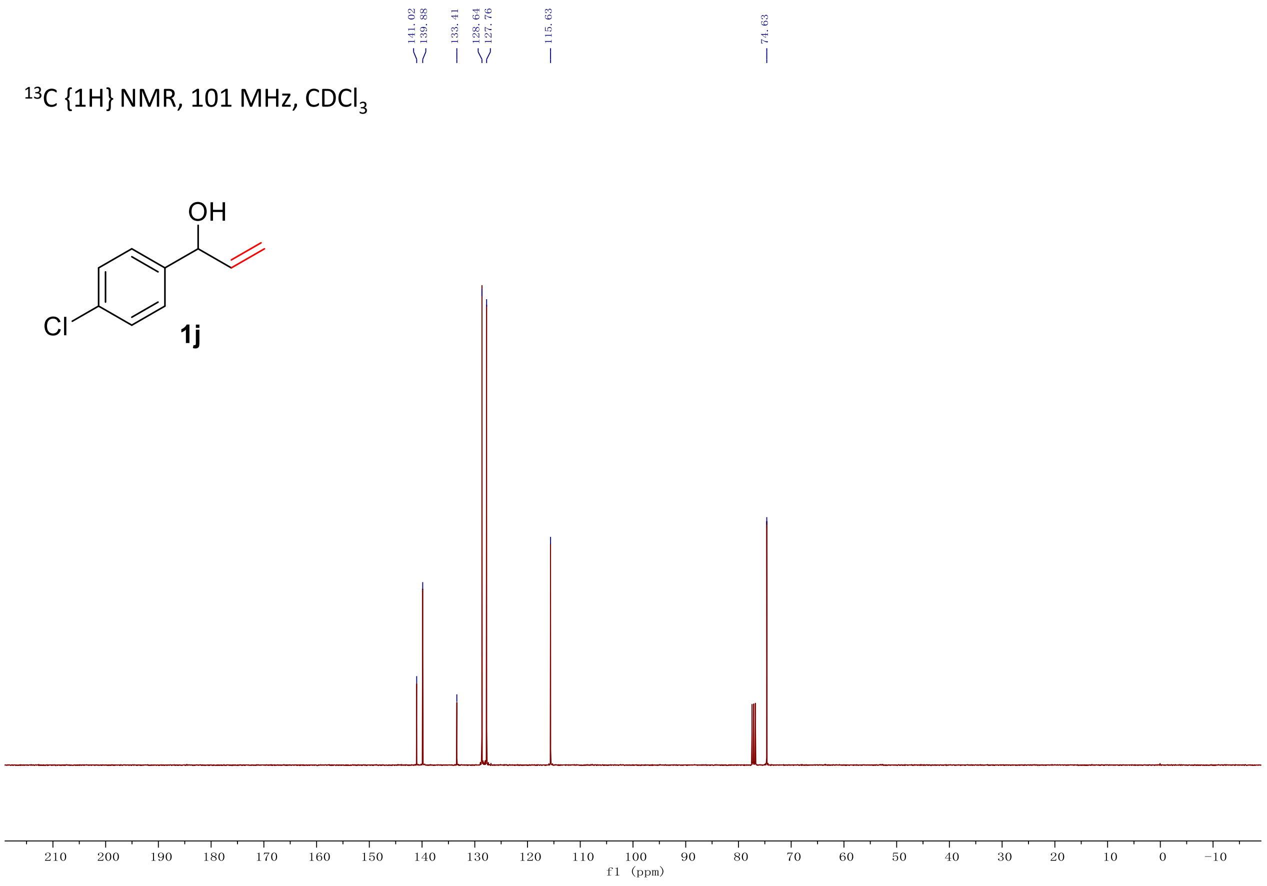

141.02 139.88 133.41 128.64 127.76 115.63 74.63
¹³C {1H} NMR, 101 MHz, CDCl₃
OH
Cl
1j
f1 (ppm)

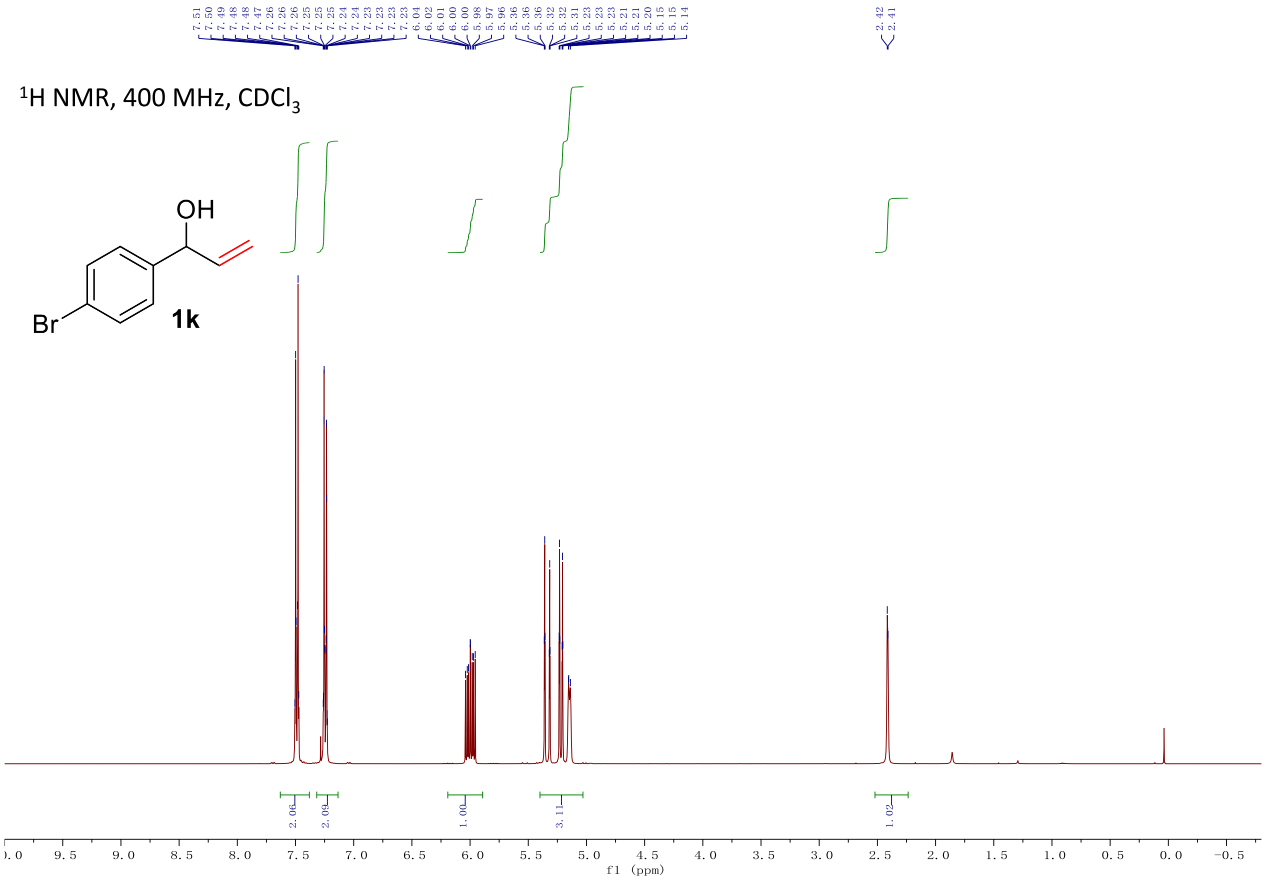
7.51 7.50 7.49 7.48 7.48 7.47 7.26 7.26 7.26 7.25 7.25 7.25 7.24 7.24 7.23 7.23 7.23 7.23 6.04 6.02 6.01 6.00 6.00 5.98 5.97 5.96 5.36 5.36 5.36 5.32 5.32 5.31 5.23 5.23 5.23 5.21 5.21 5.20 5.15 5.15 5.14
2.42 2.41
1H NMR, 400 MHz, CDCl3
OH
Br
1k
2.06
2.09
1.00
3.11
1.02
f1 (ppm)

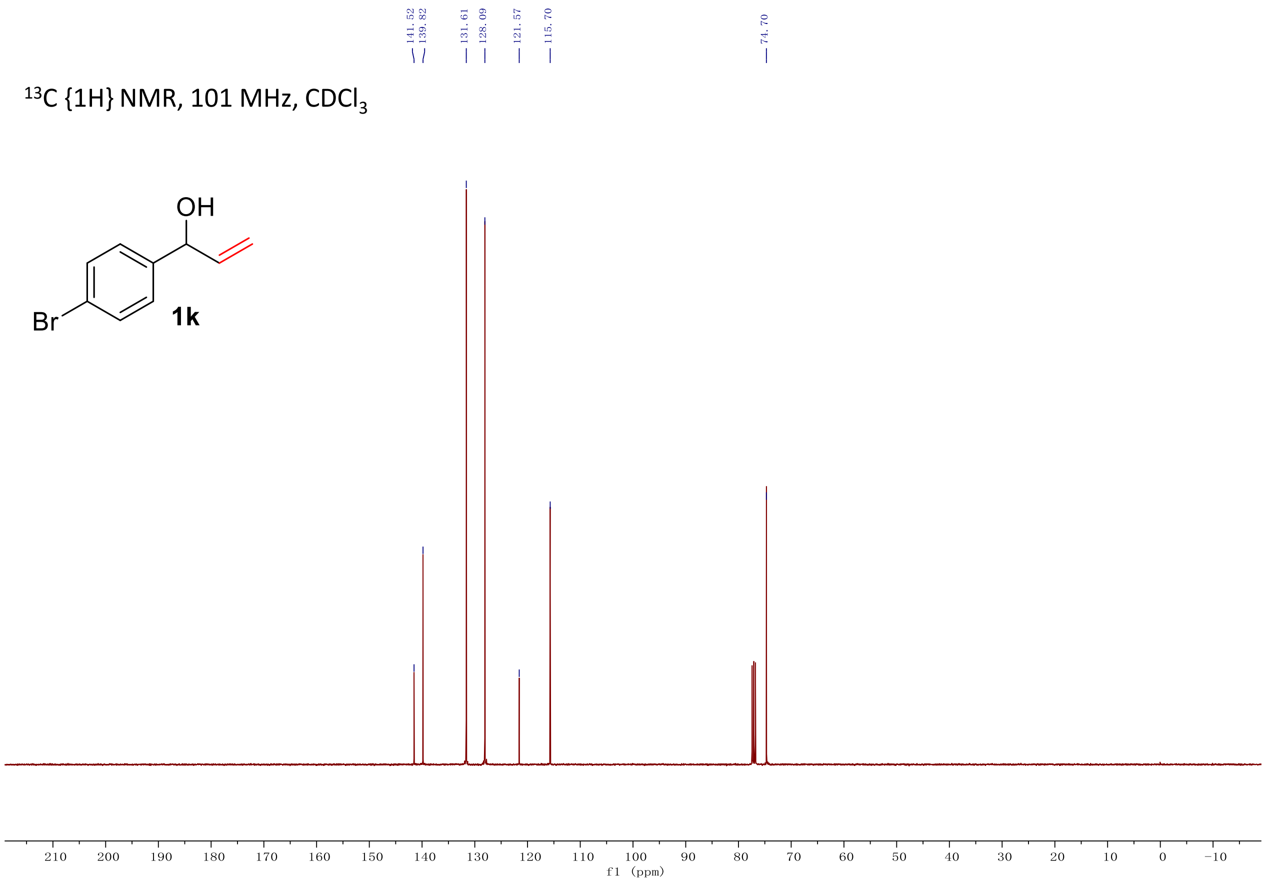
141.52 139.82 131.61 128.09 121.57 115.70
74.70
13C {1H} NMR, 101 MHz, CDCl3
OH
Br
1k
f1 (ppm)

$^{1}H$ NMR, 400 MHz, $CDCl_3$

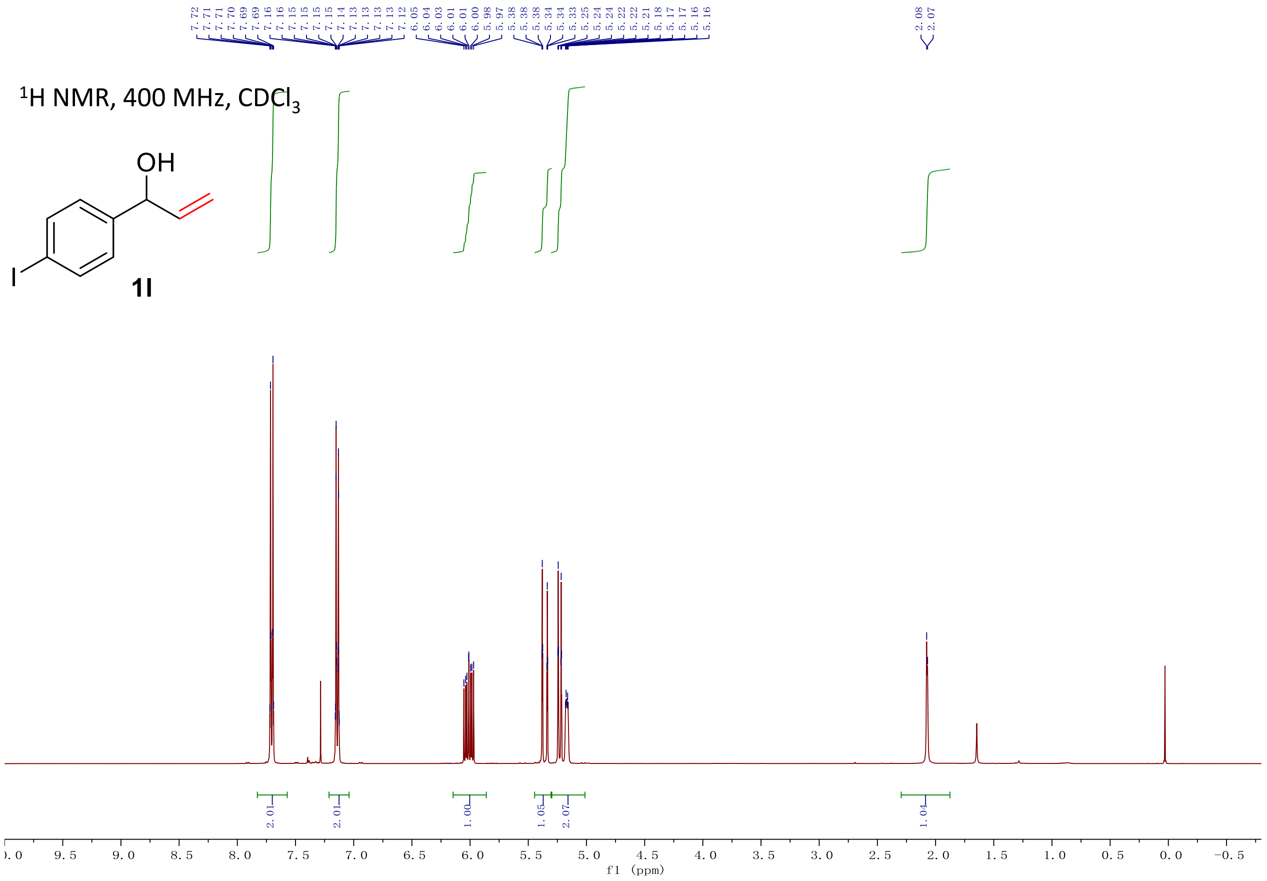

$^{13}C$ {1H} NMR, 101 MHz, $CDCl_3$

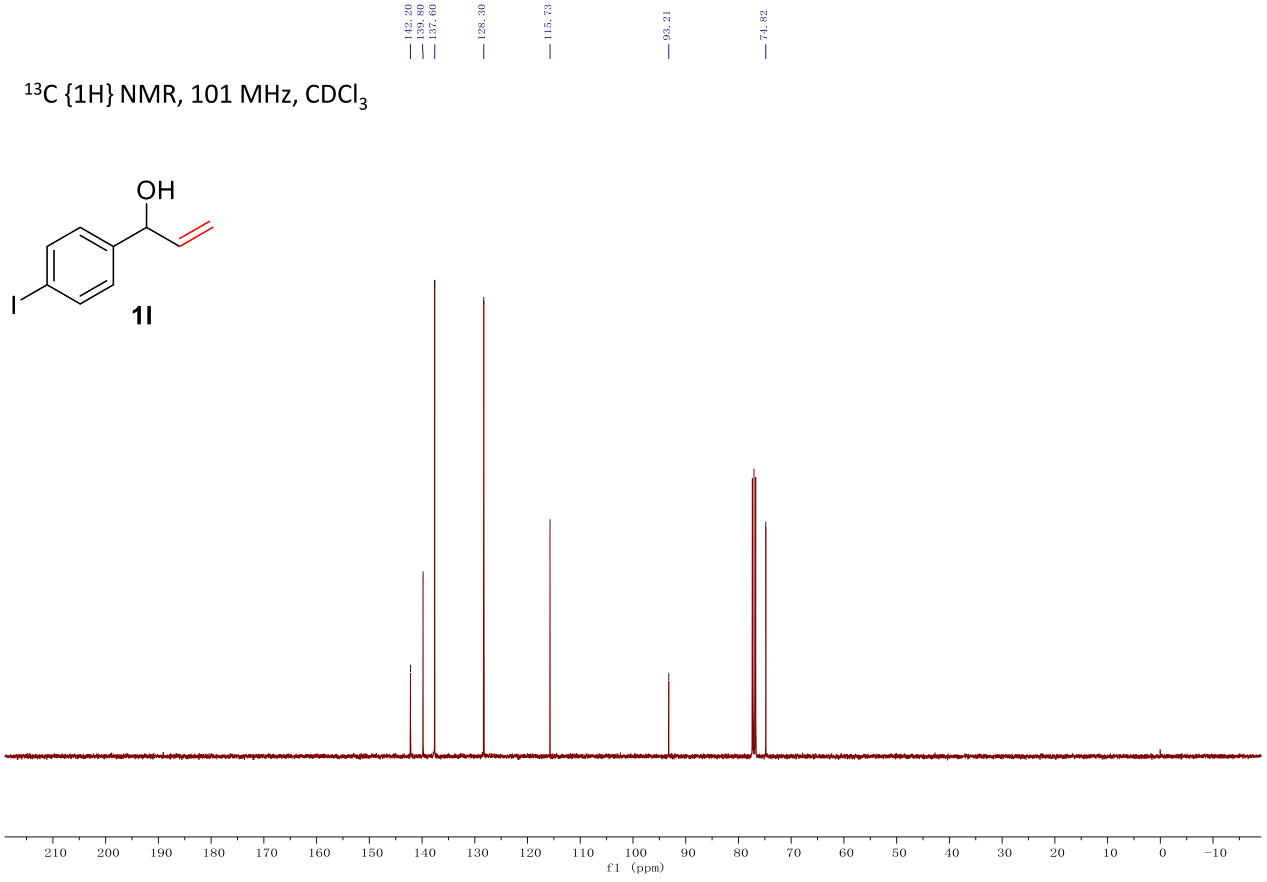

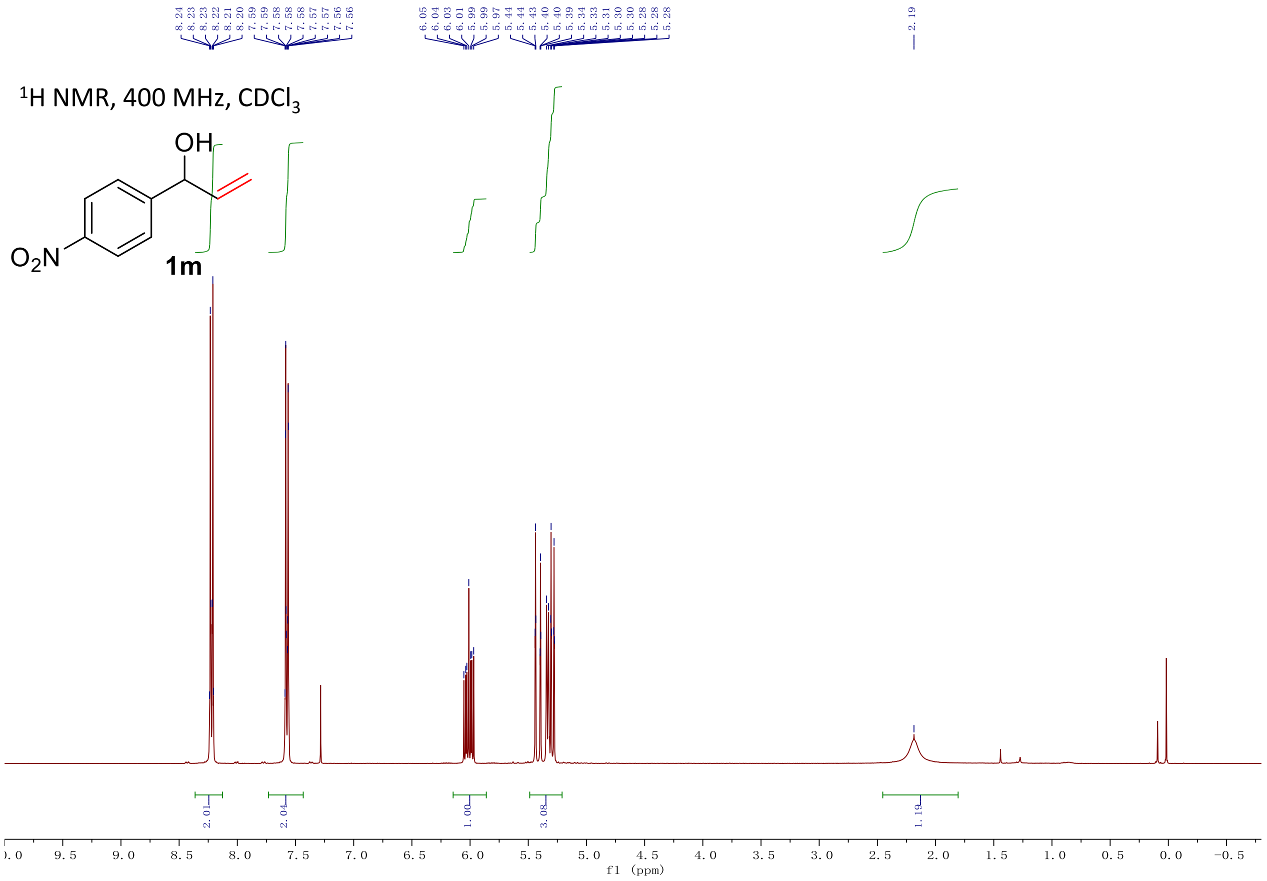

8.24 8.23 8.23 8.22 8.21 8.20 7.59 7.58 7.58 7.58 7.57 7.57 7.56 7.56
6.05 6.04 6.03 6.01 5.99 5.99 5.97 5.44 5.44 5.43 5.40 5.40 5.39 5.34 5.33 5.31 5.30 5.28 5.28 5.28
2.19
1H NMR, 400 MHz, CDCl3
OH
O2N
1m
2.01
2.04
1.00
3.08
1.19
f1 (ppm)


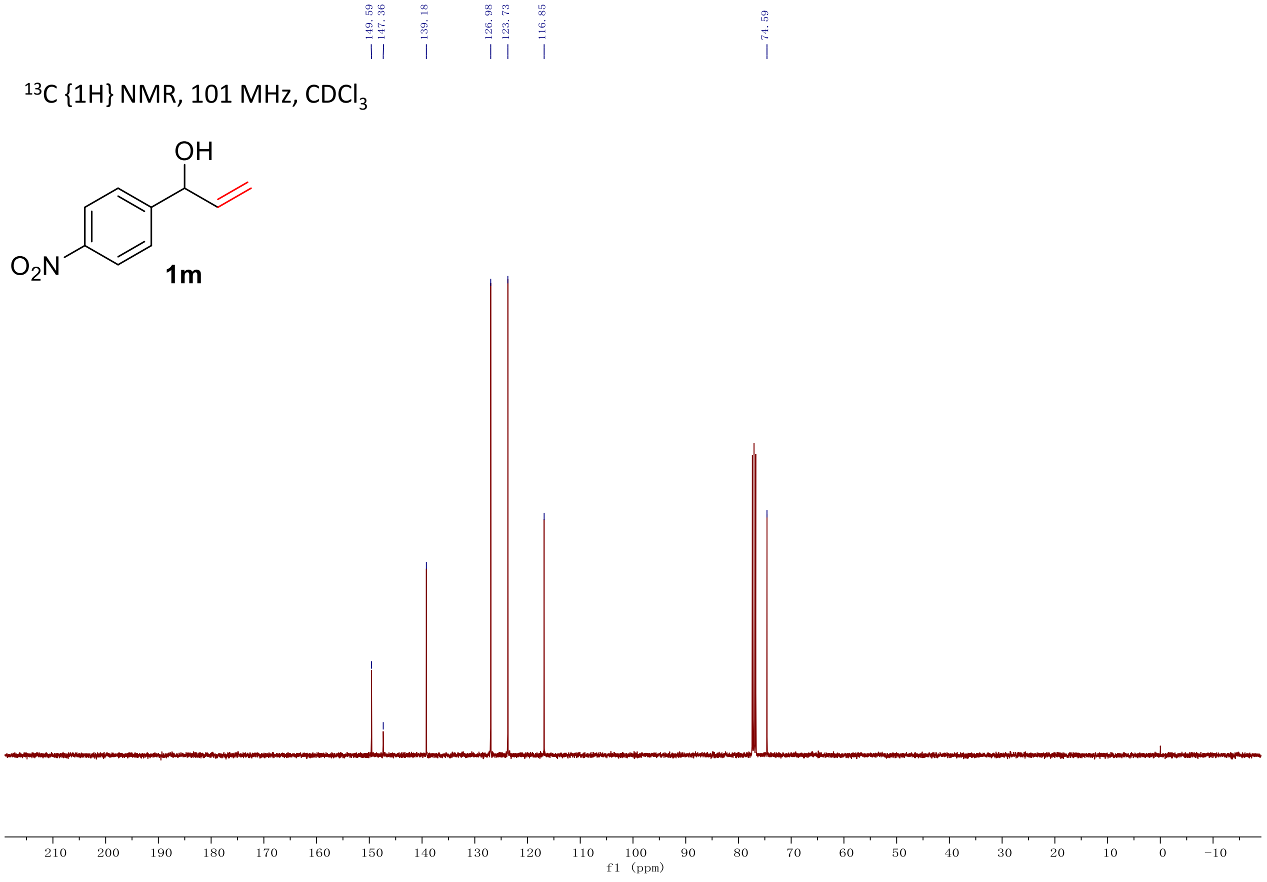

149.59 147.36 139.18 126.98 123.73 116.85 74.59
13C {1H} NMR, 101 MHz, CDCl3
OH
O2N
1m
f1 (ppm)

$^1H$ NMR, 400 MHz, $CDCl_3$

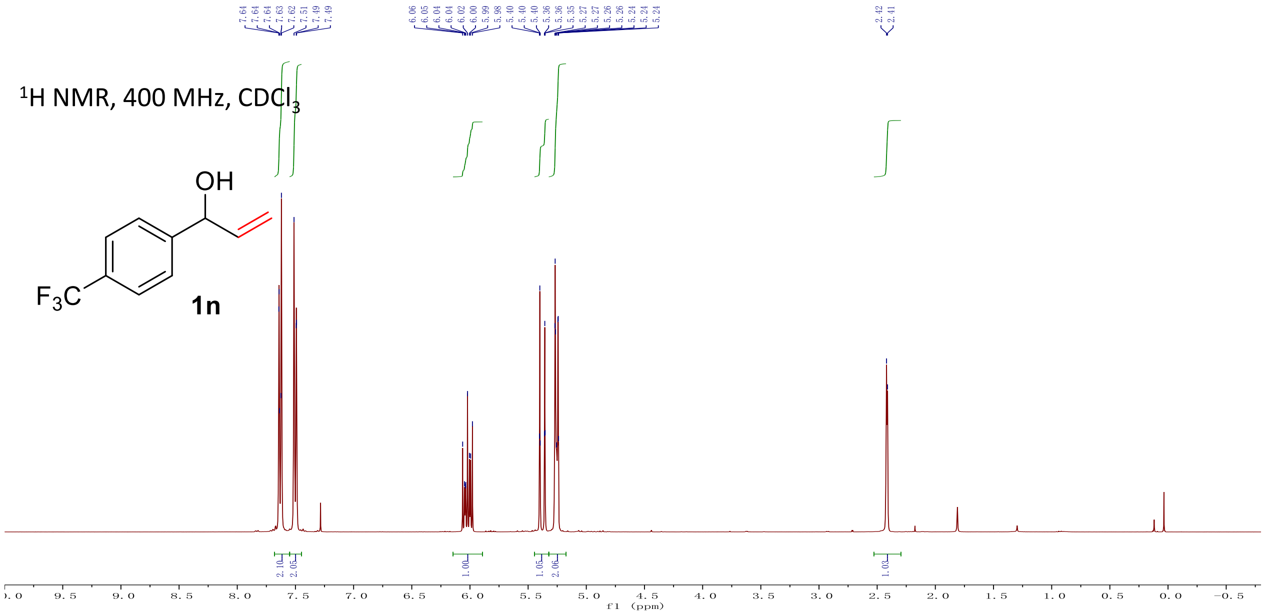


$^{13}C$ {1H} NMR, 101 MHz, $CDCl_3$

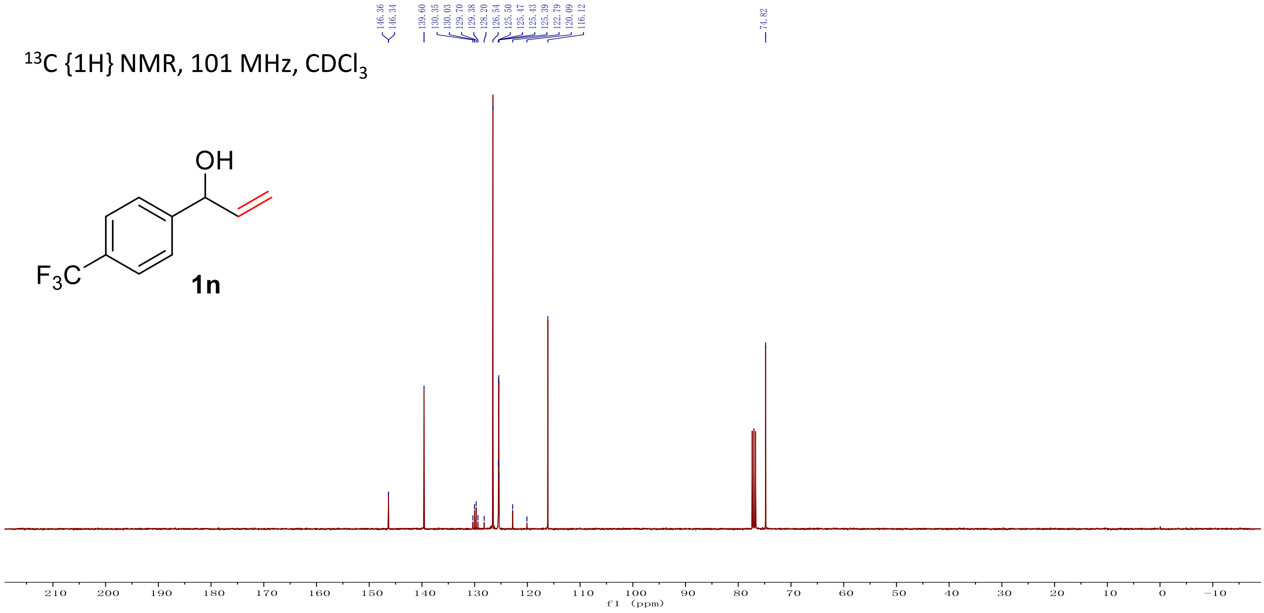


$^{19}F$ NMR, 376 MHz, $CDCl_3$

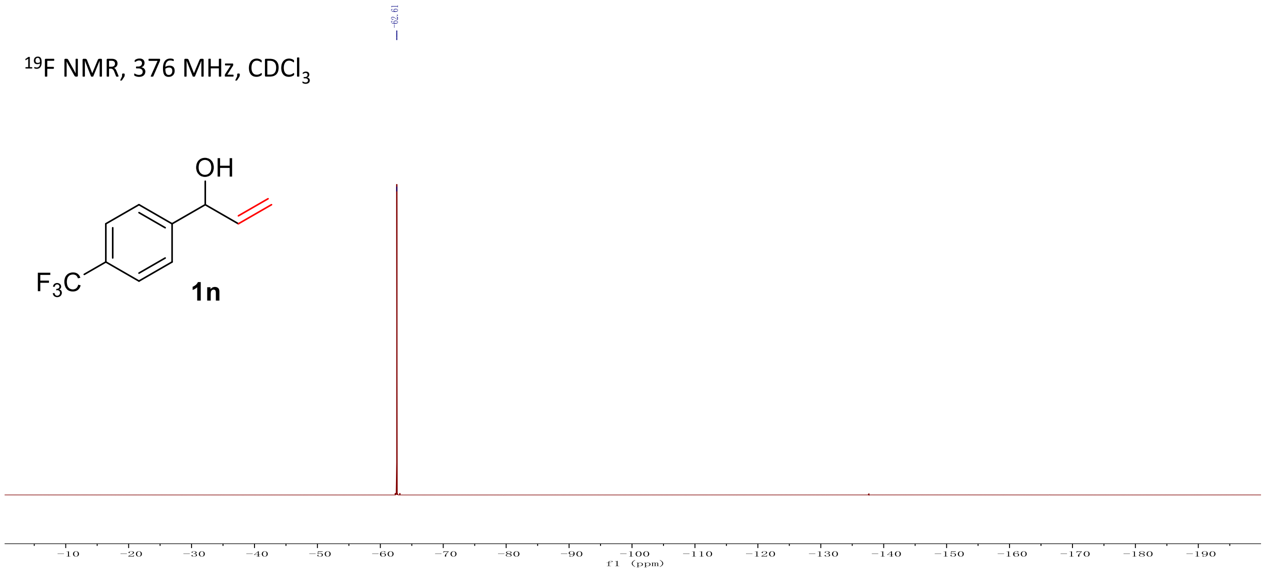

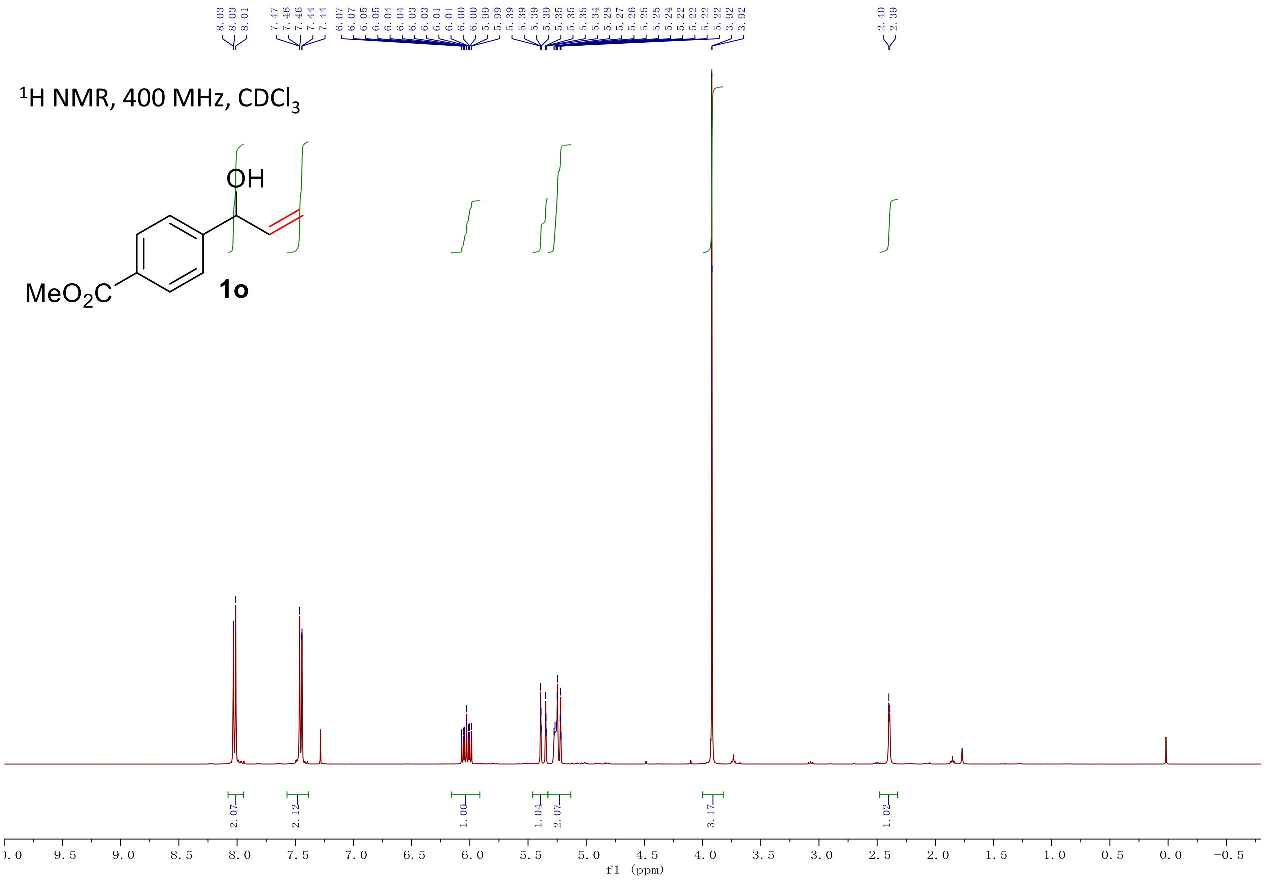
1H NMR, 400 MHz, CDCl3
OH
MeO2C
1o
8.03 8.03 8.01 7.47 7.46 7.46 7.44 7.44 6.07 6.07 6.05 6.05 6.04 6.04 6.03 6.03 6.01 6.01 6.00 6.00 5.99 5.99 5.39 5.39 5.39 5.39 5.35 5.35 5.35 5.34 5.28 5.27 5.26 5.25 5.25 5.24 5.22 5.22 5.22 5.22 3.92 3.92 2.40 2.39
2.07 2.12 1.00 1.04 2.07 3.17 1.02
f1 (ppm)

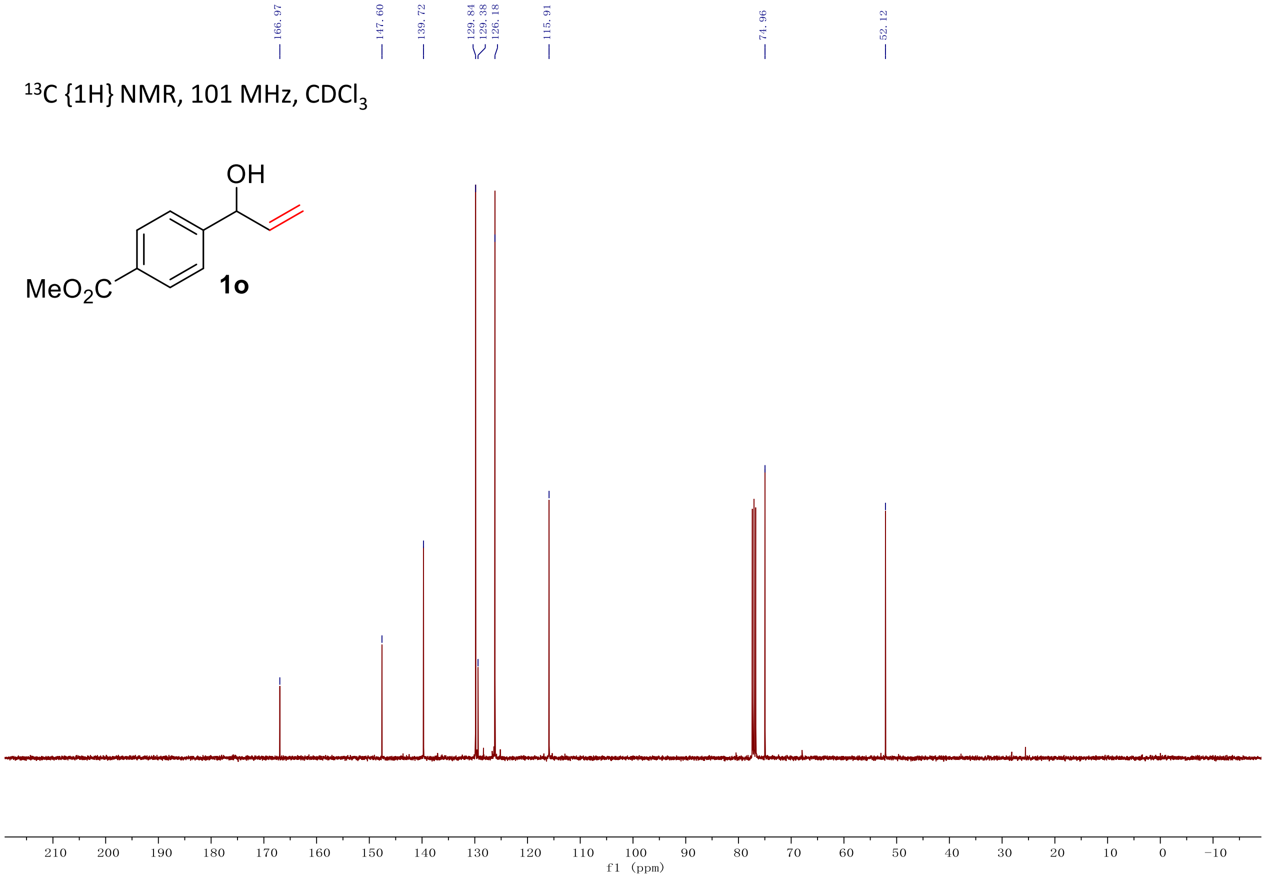
13C {1H} NMR, 101 MHz, CDCl3
OH
MeO2C
1o
166.97 147.60 139.72 129.84 129.38 126.18 115.91 74.96 52.12
f1 (ppm)

$^{1}H$ NMR, 400 MHz, $CDCl_3$

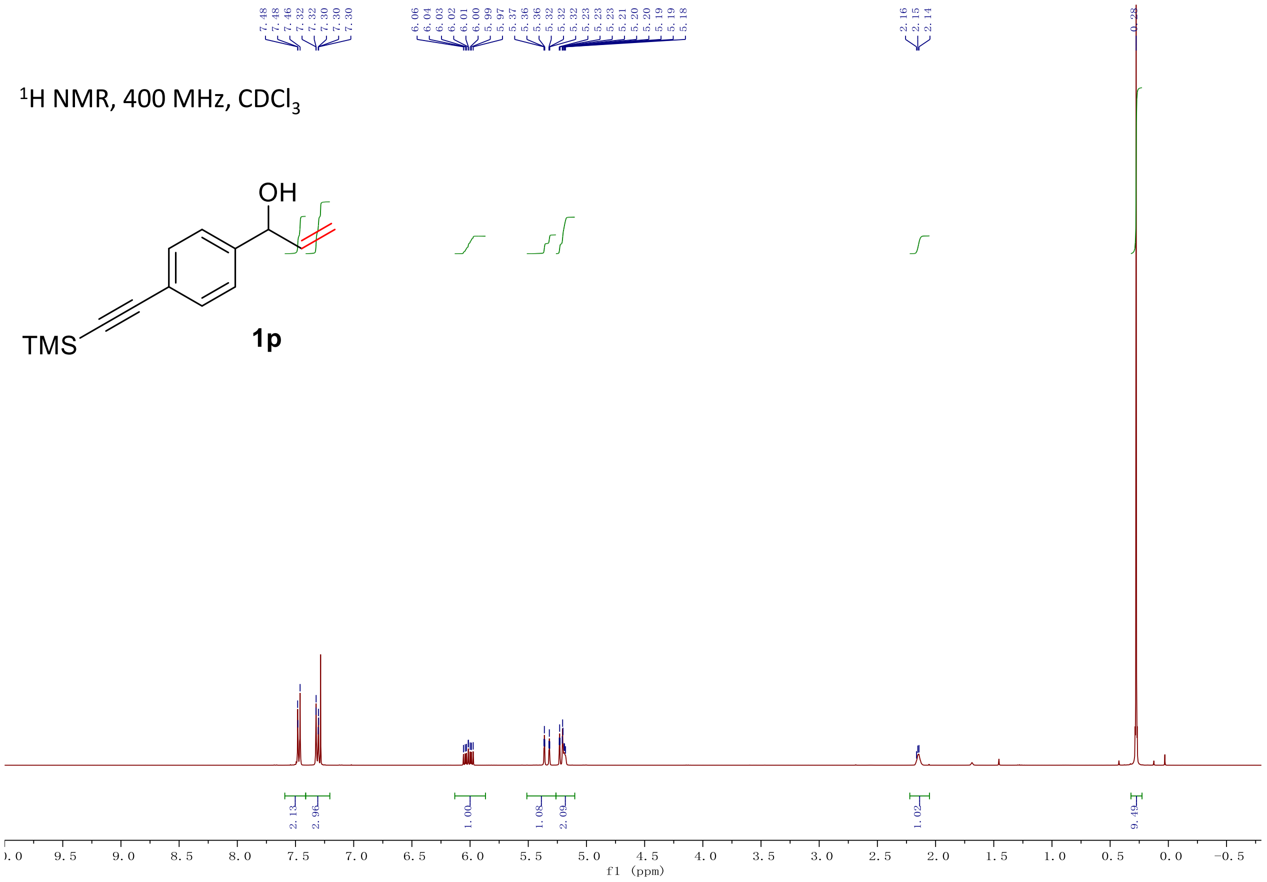

$^{13}C$ {1H} NMR, 101 MHz, $CDCl_3$

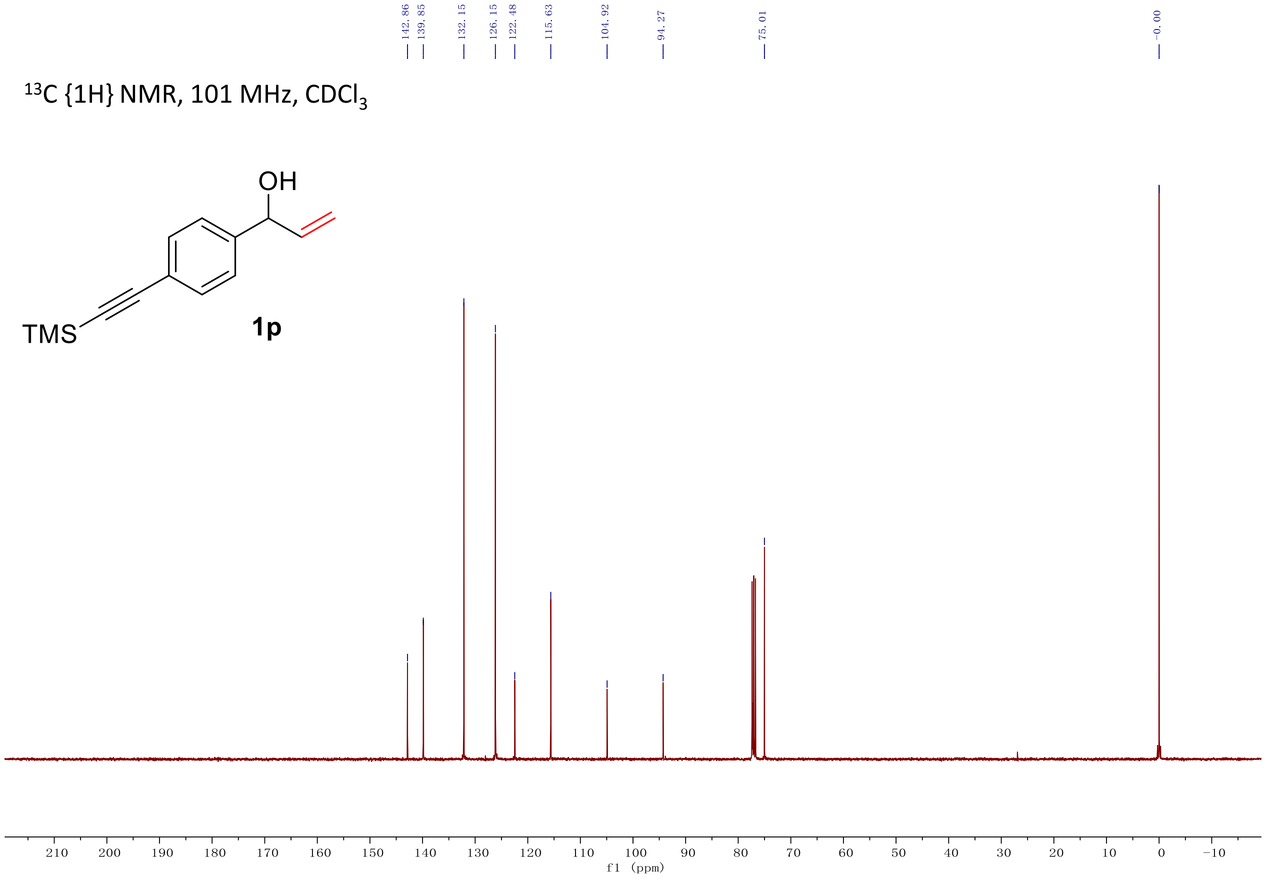

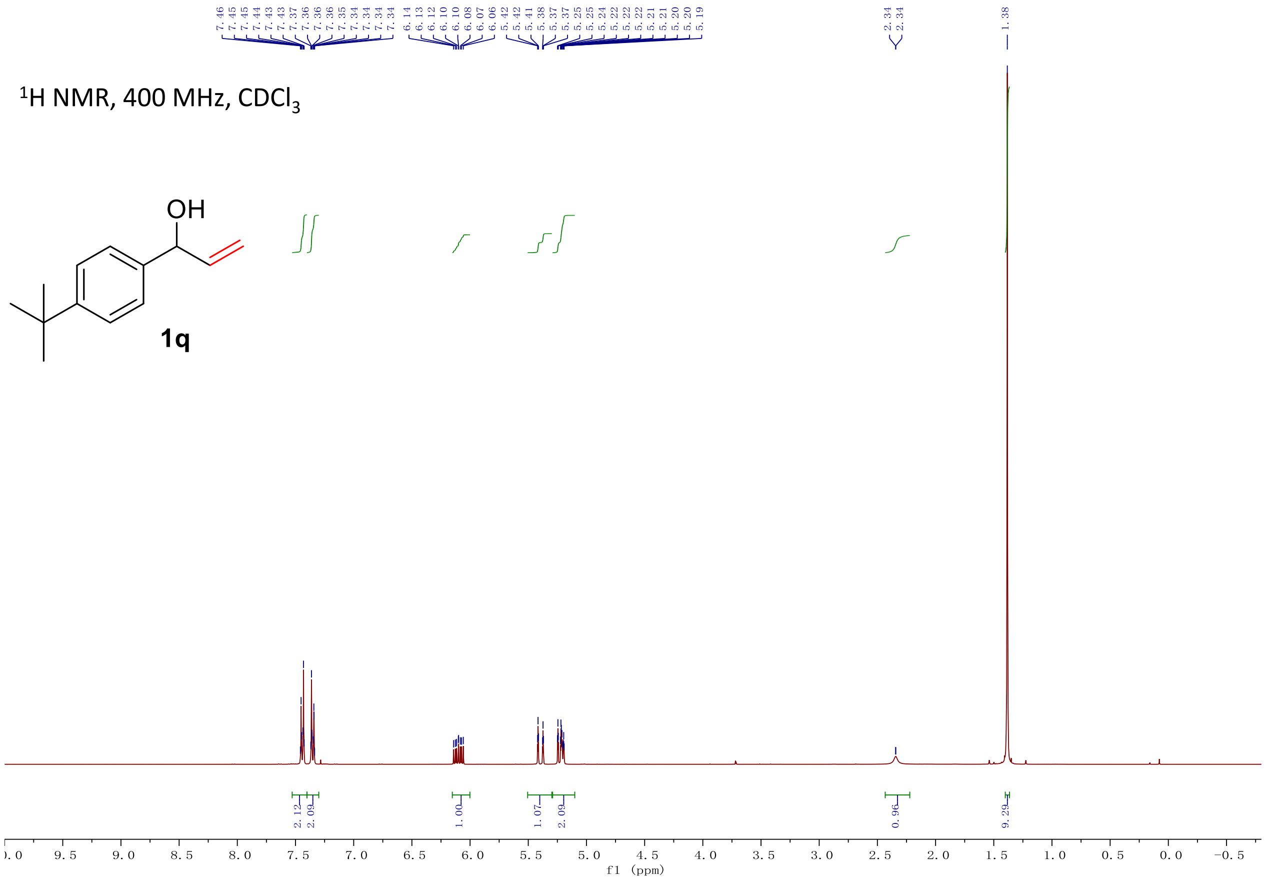
1H NMR, 400 MHz, CDCl3
OH
1q

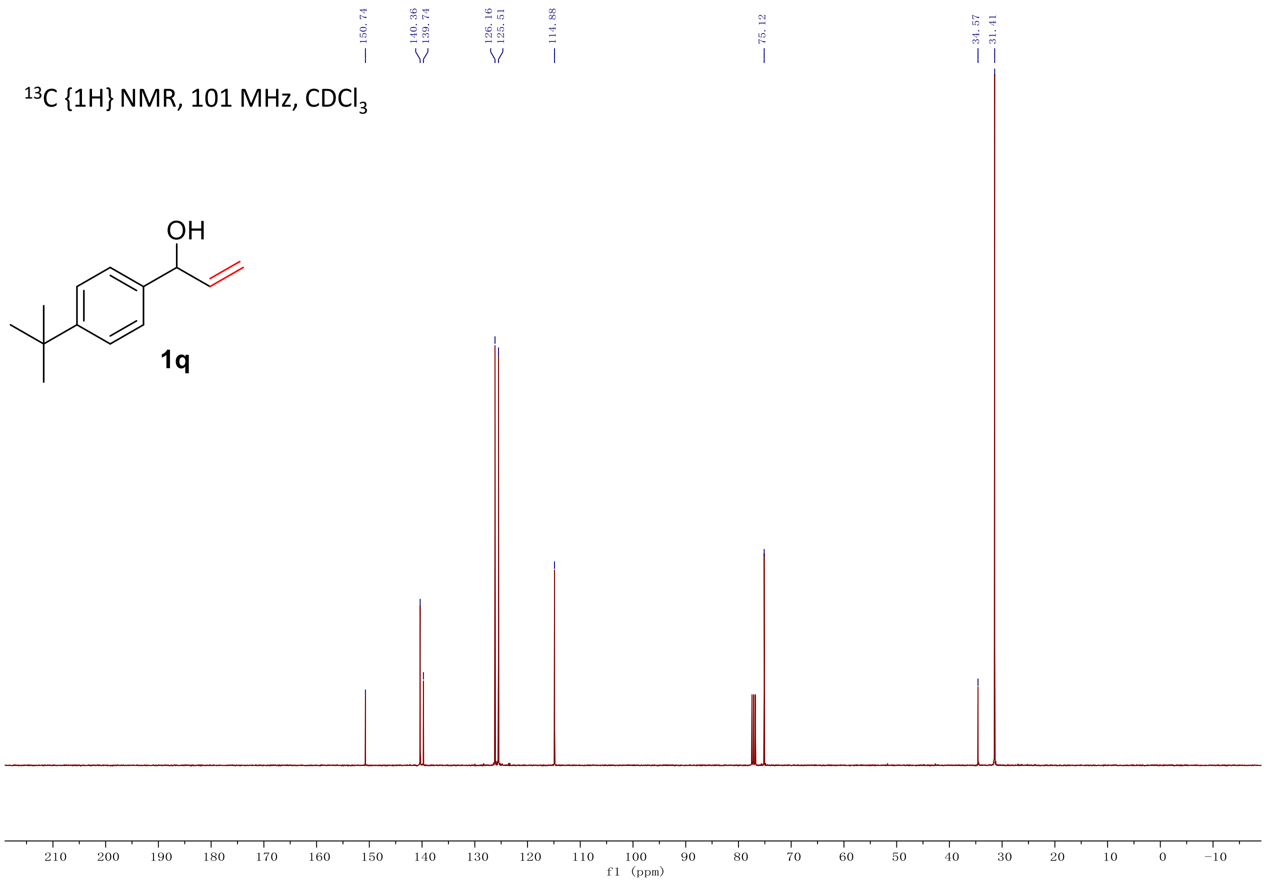
13C {1H} NMR, 101 MHz, CDCl3
OH
1q

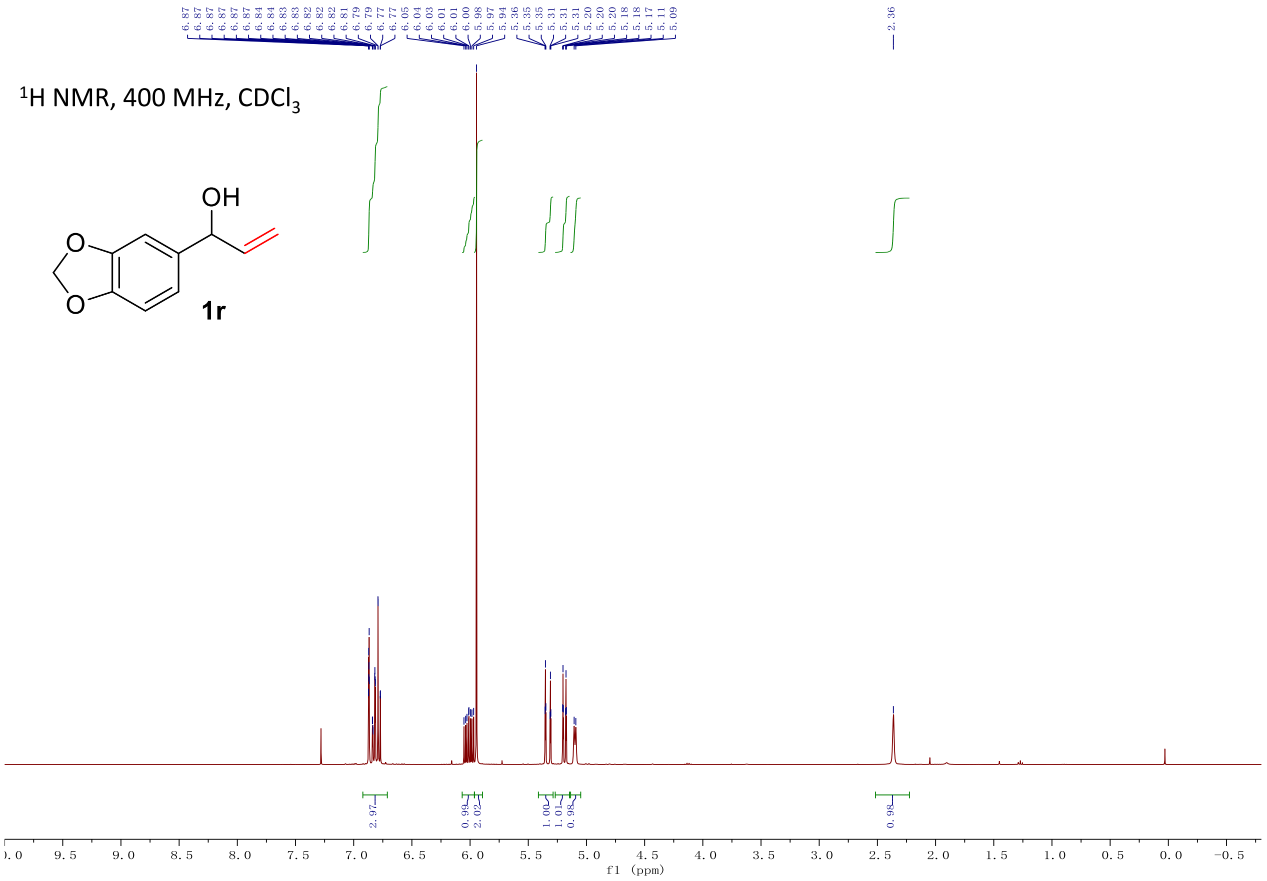

6.87 6.87 6.87 6.87 6.87 6.84 6.84 6.83 6.83 6.82 6.82 6.81 6.79 6.79 6.77 6.77 6.05 6.04 6.03 6.01 6.01 6.00 5.98 5.97 5.94 5.36 5.35 5.35 5.31 5.31 5.31 5.20 5.20 5.20 5.18 5.17 5.11 5.09
2.36
1H NMR, 400 MHz, CDCl3
OH
O
O
1r
2.97
0.99
2.02
1.00
1.01
0.98
0.98
f1 (ppm)


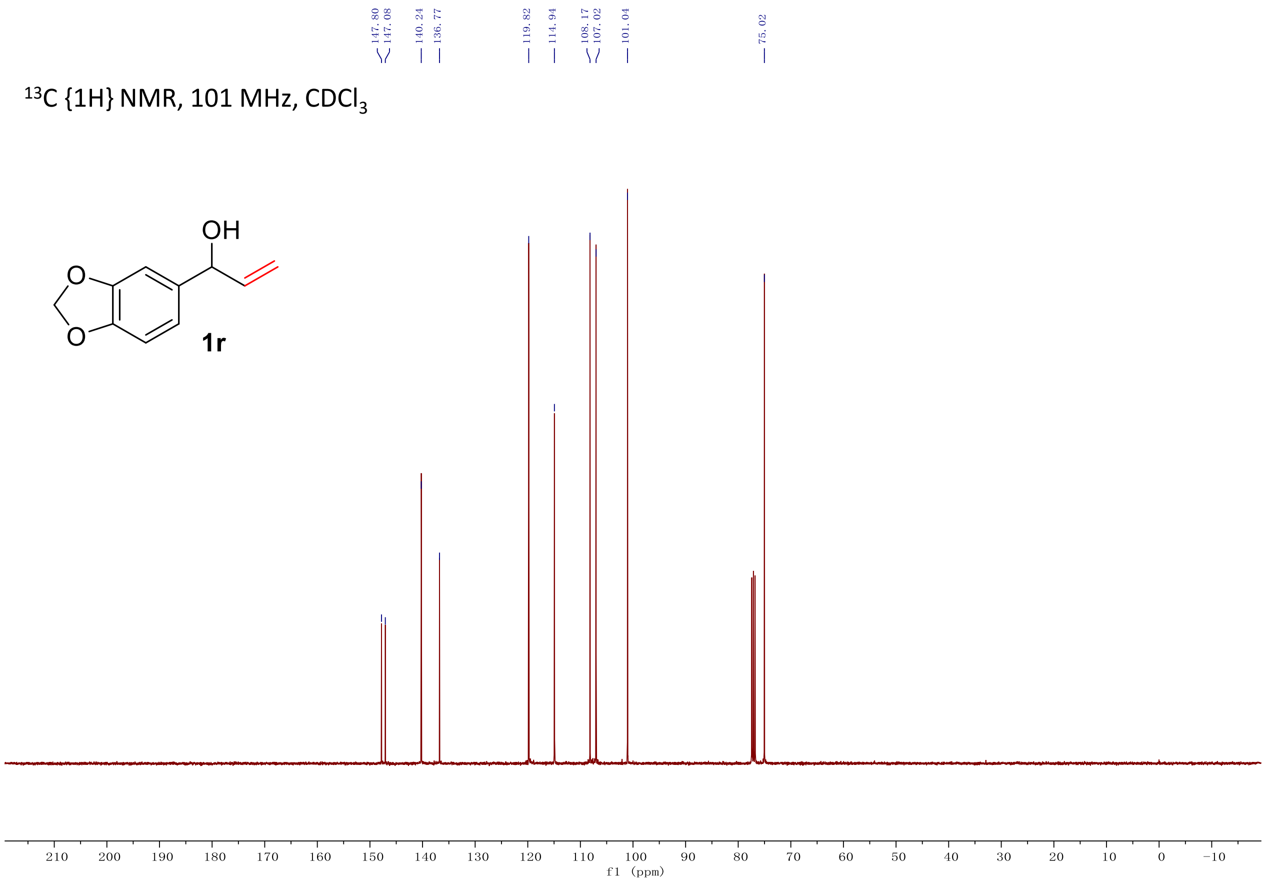

147.80 147.08 140.24 136.77 119.82 114.94 108.17 107.02 101.04 75.02
13C {1H} NMR, 101 MHz, CDCl3
OH
O
O
1r
f1 (ppm)

$^1H$ NMR, 400 MHz, $CDCl_3$

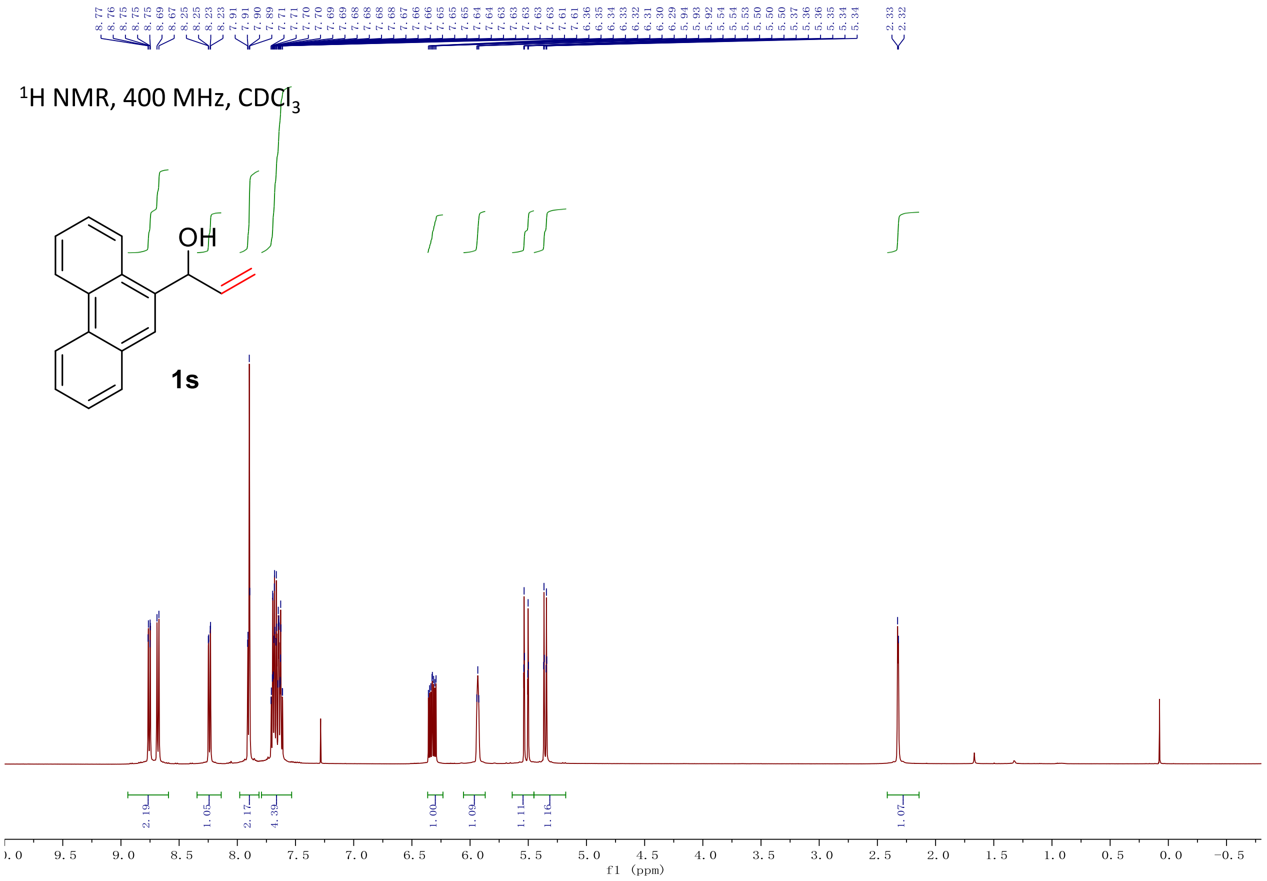

$^{13}C$ {1H} NMR, 101 MHz, $CDCl_3$

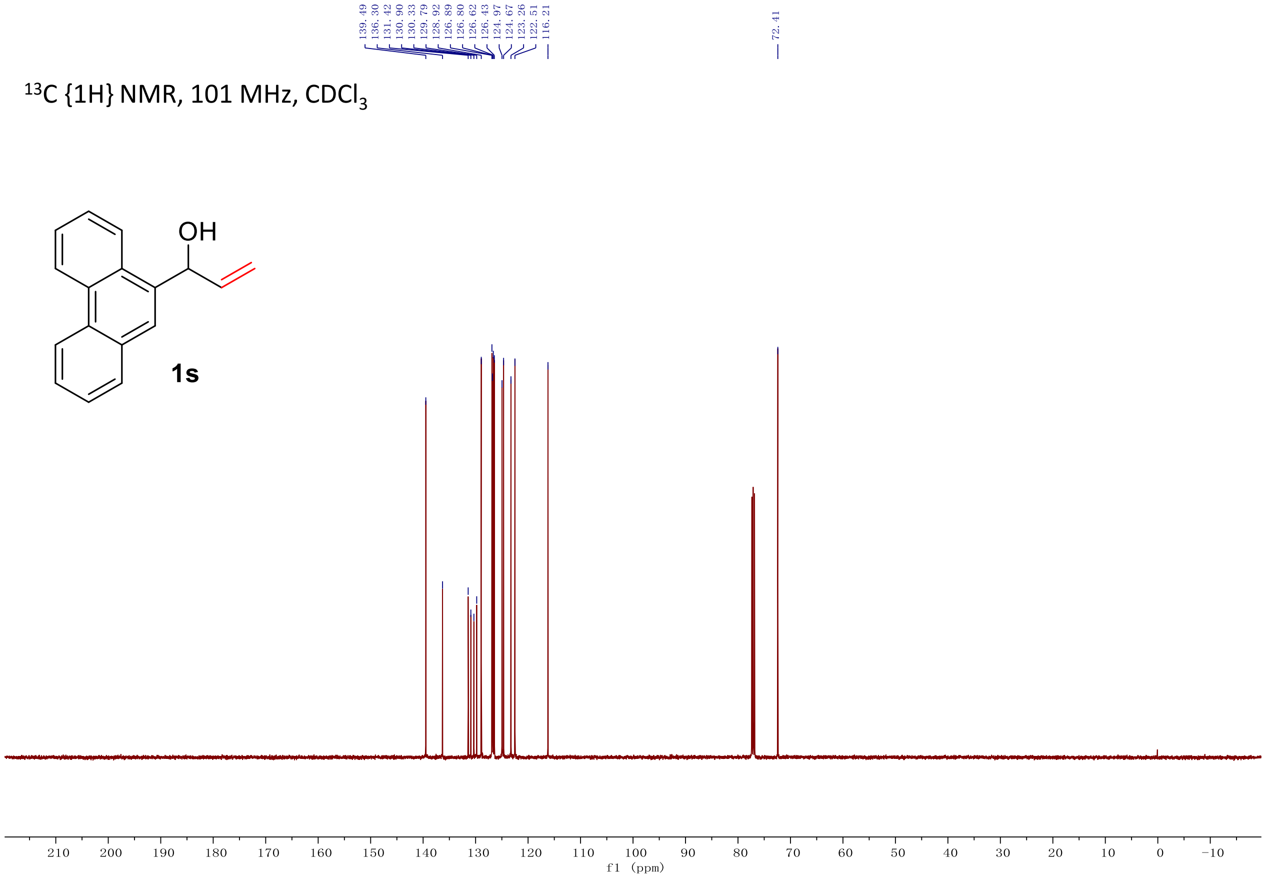

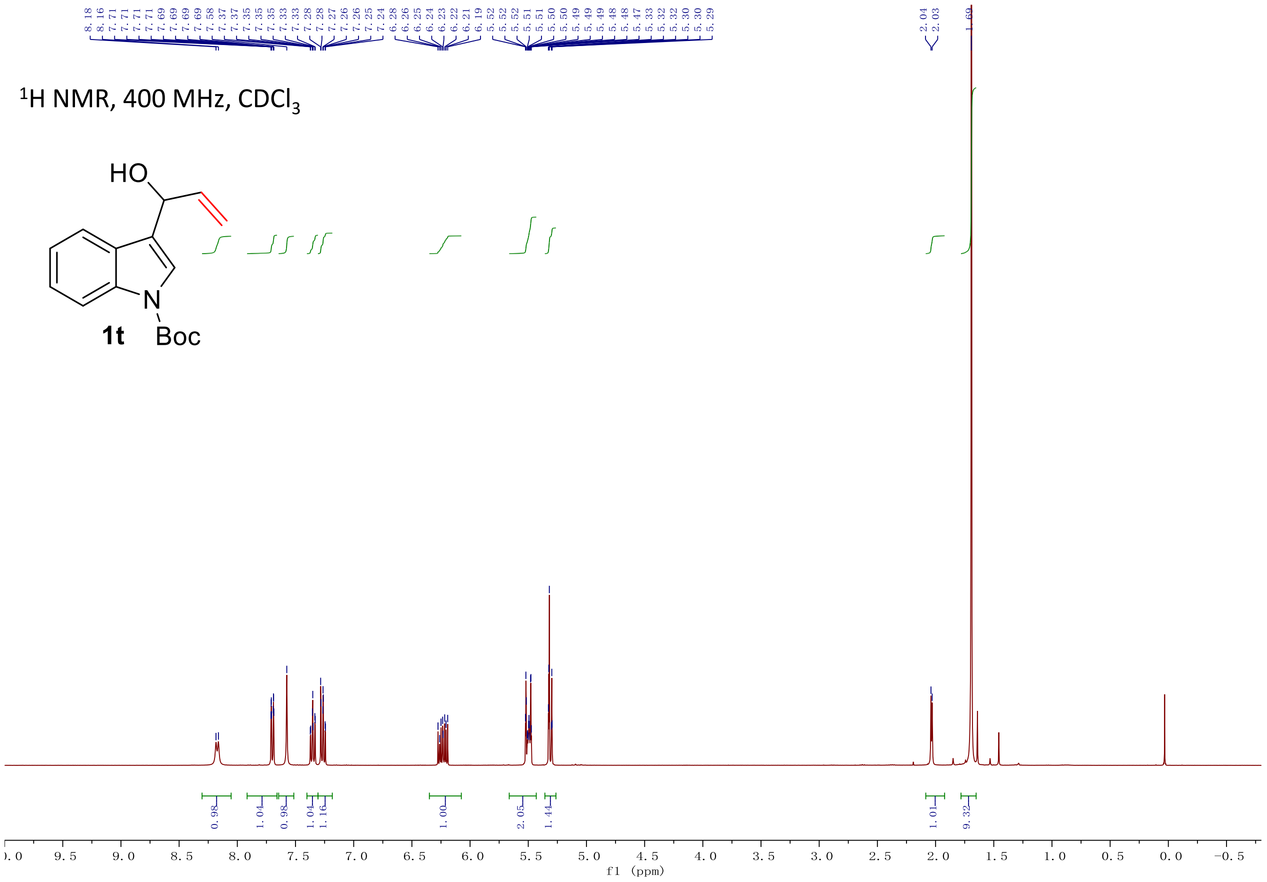

$^{1}$H NMR, 400 MHz, $CDCl_3$
HO
N
1t
Boc
f1 (ppm)


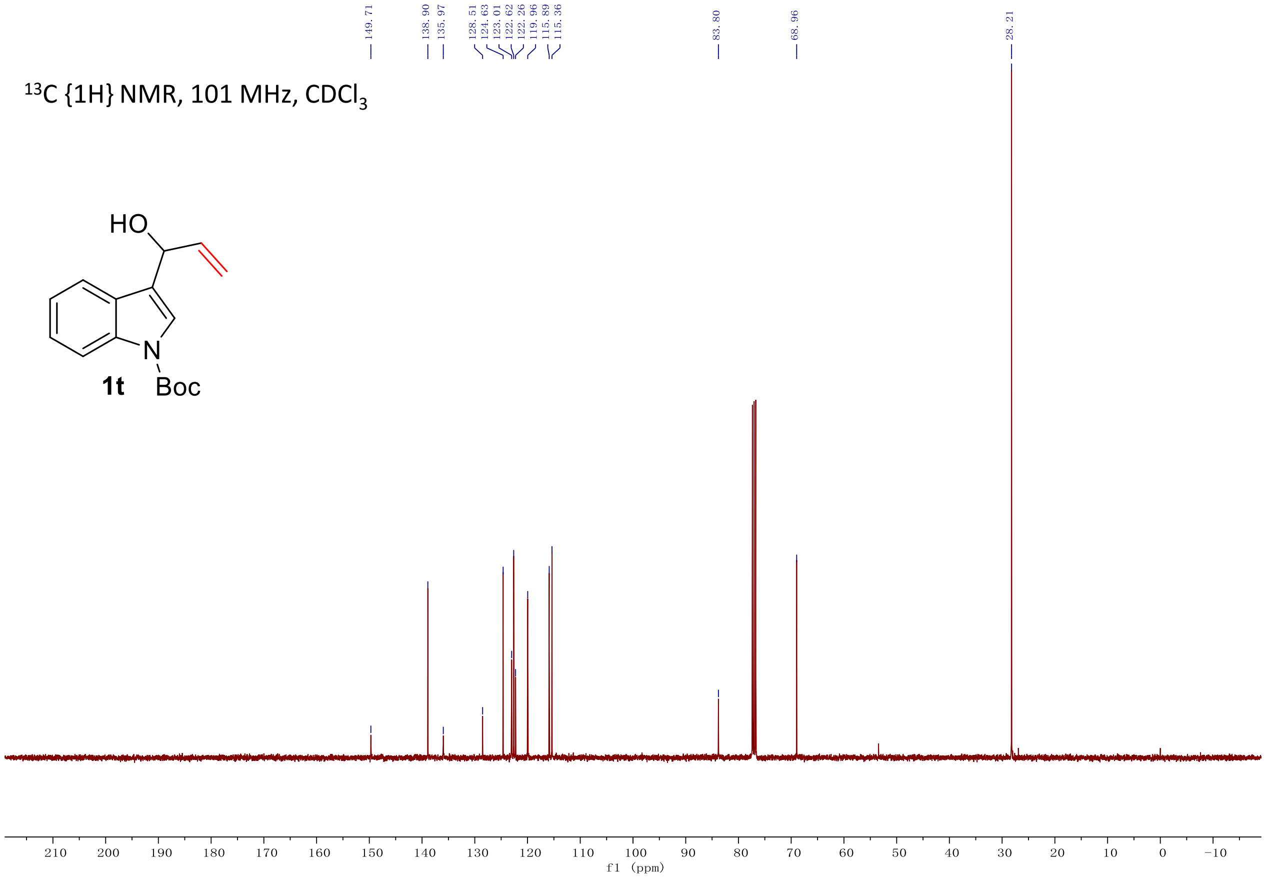

$^{13}$C {1H} NMR, 101 MHz, $CDCl_3$
HO
N
1t
Boc
149.71
138.90
135.97
128.51
124.63
123.01
122.62
122.26
119.96
115.89
115.36
83.80
68.96
28.21
f1 (ppm)

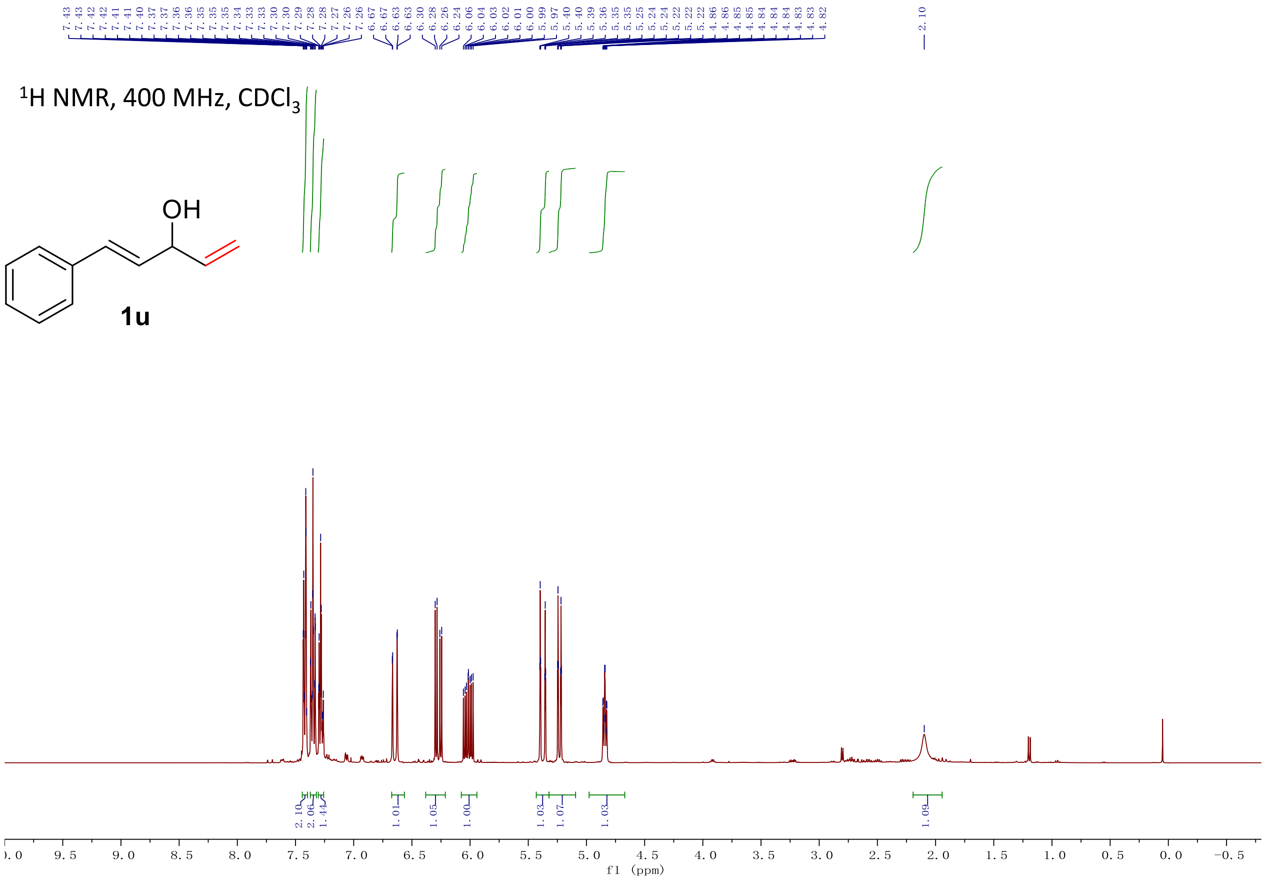

1H NMR, 400 MHz, CDCl3
OH
1u
f1 (ppm)


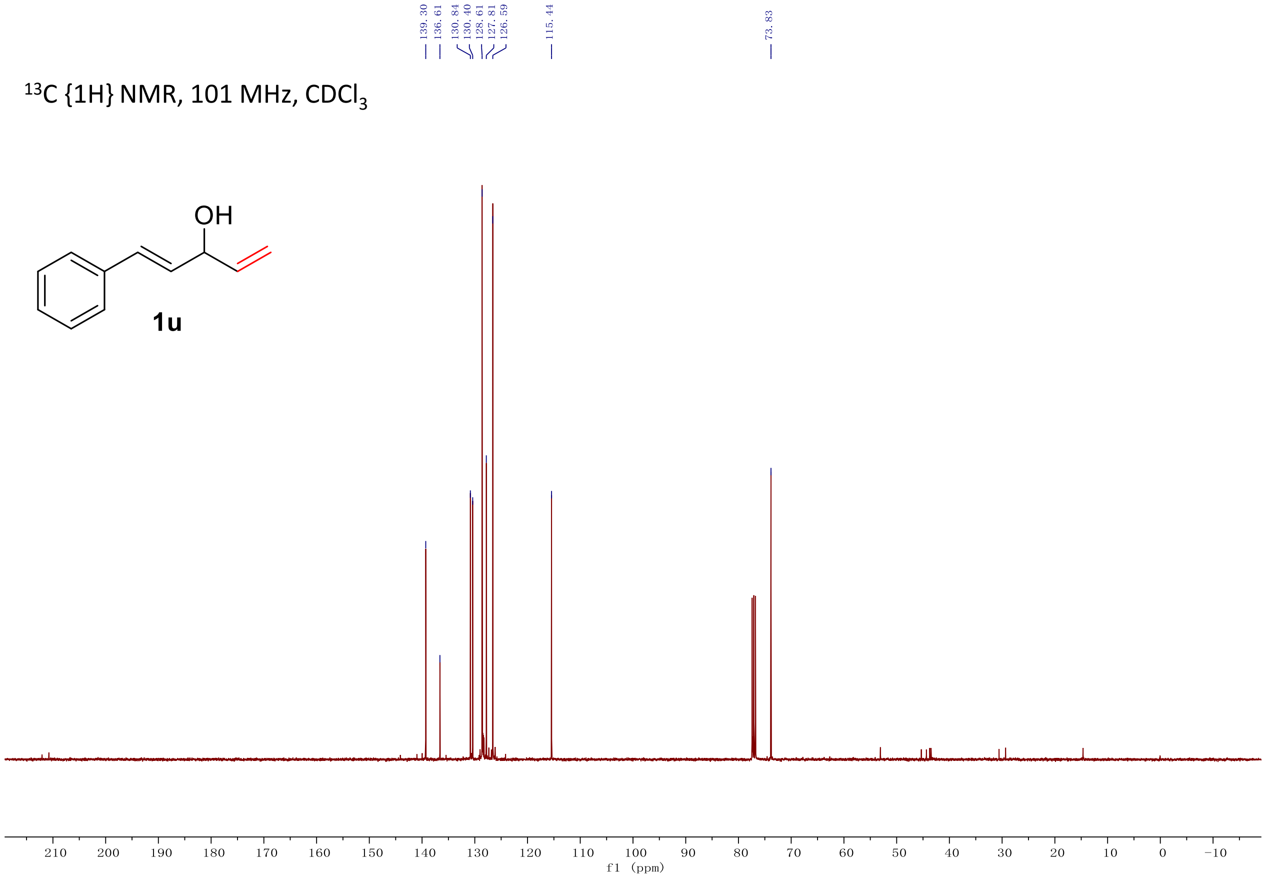

13C {1H} NMR, 101 MHz, CDCl3
OH
1u
f1 (ppm)

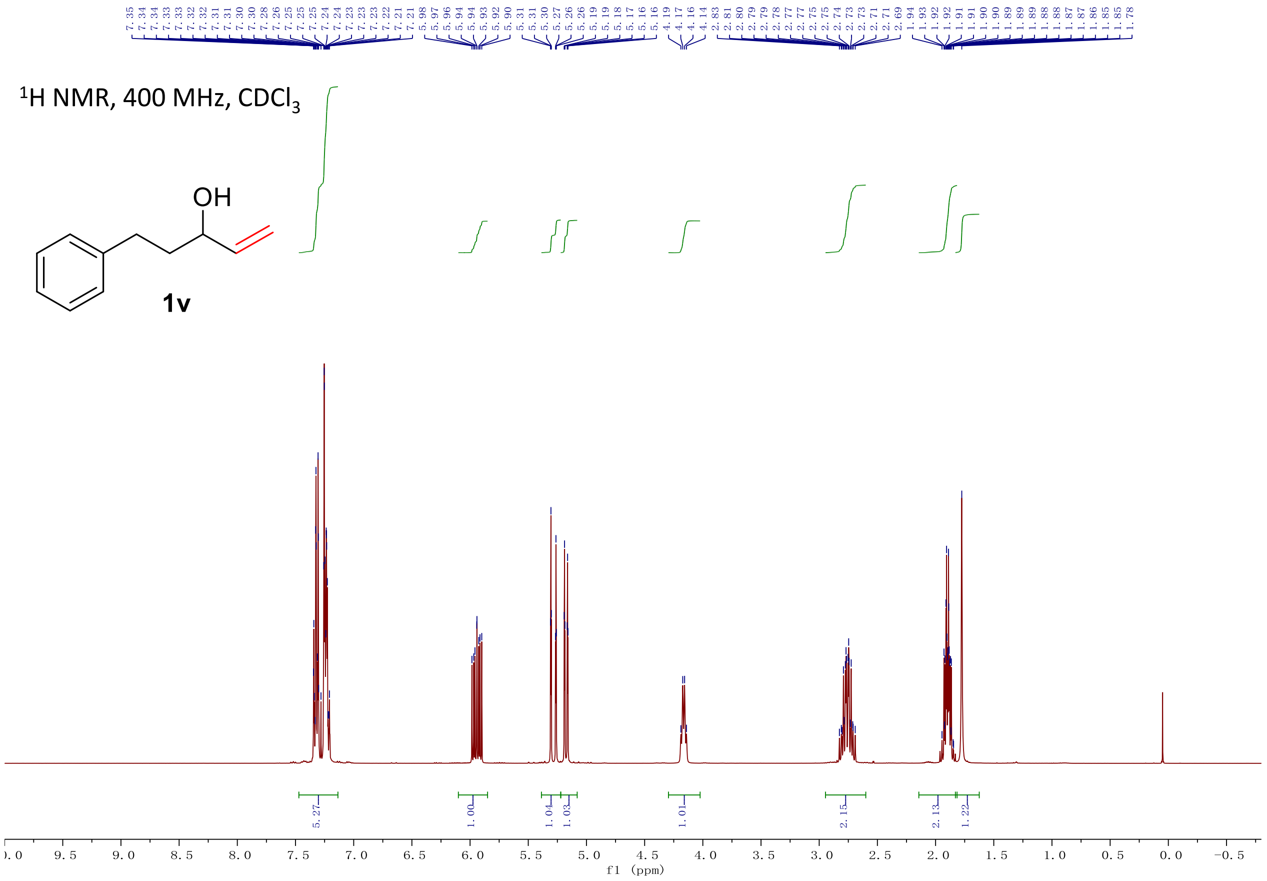
1H NMR, 400 MHz, CDCl3
OH
1v
f1 (ppm)

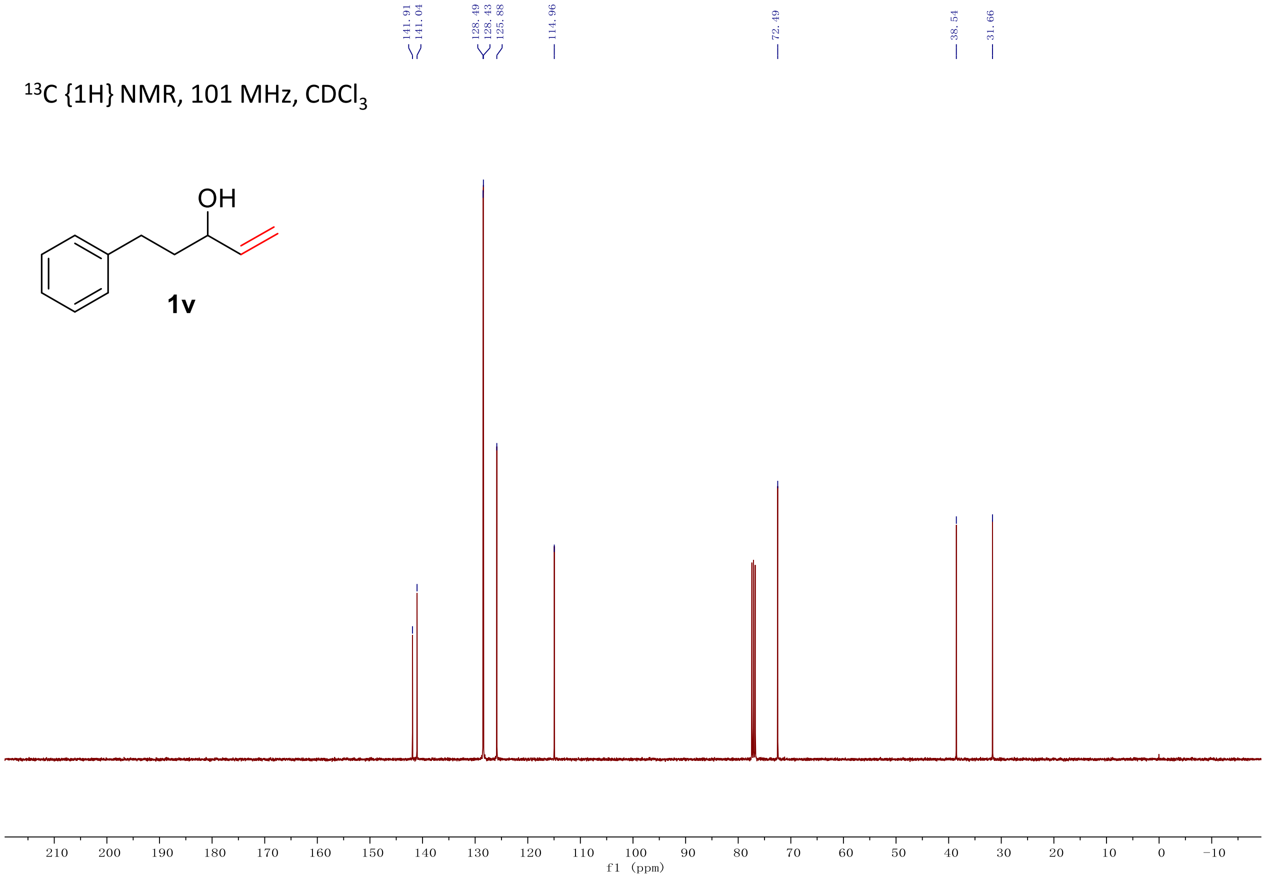
13C {1H} NMR, 101 MHz, CDCl3
OH
1v
141.91
141.04
128.49
128.43
125.88
114.96
72.49
38.54
31.66
f1 (ppm)

$^{1}H$ NMR, 400 MHz, $CDCl_3$

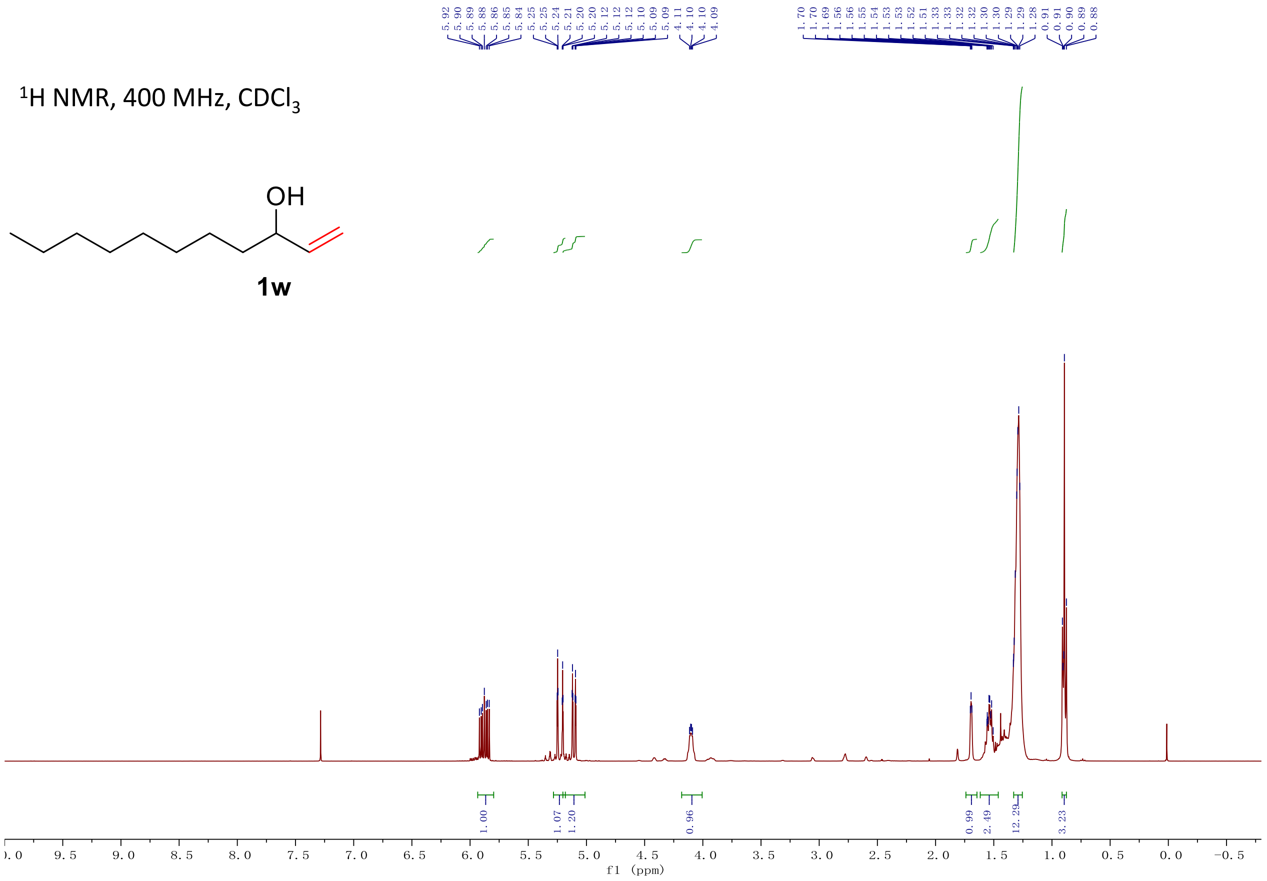


$^{13}C$ {1H} NMR, 101 MHz, $CDCl_3$

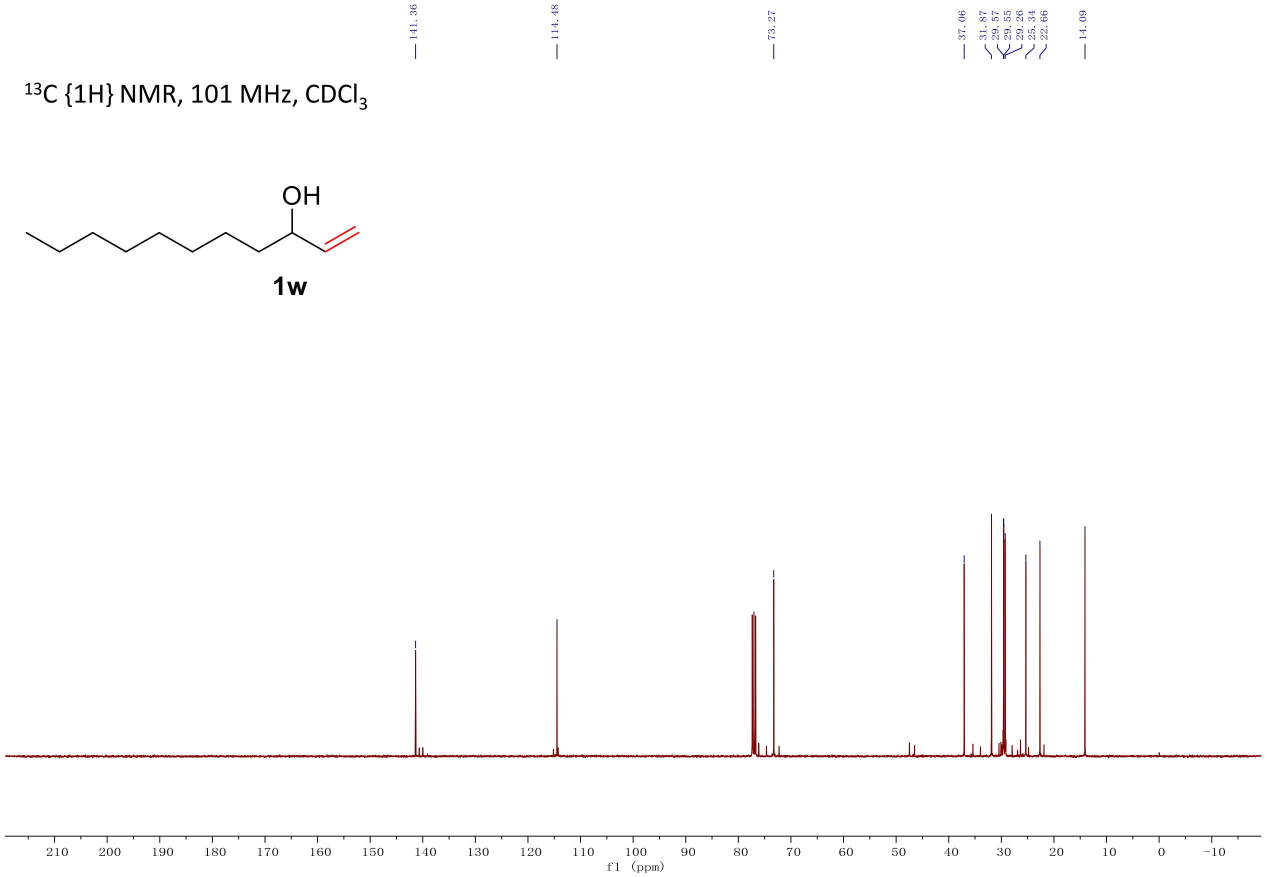

$^{1}H$ NMR, 500 MHz, $CDCl_3$

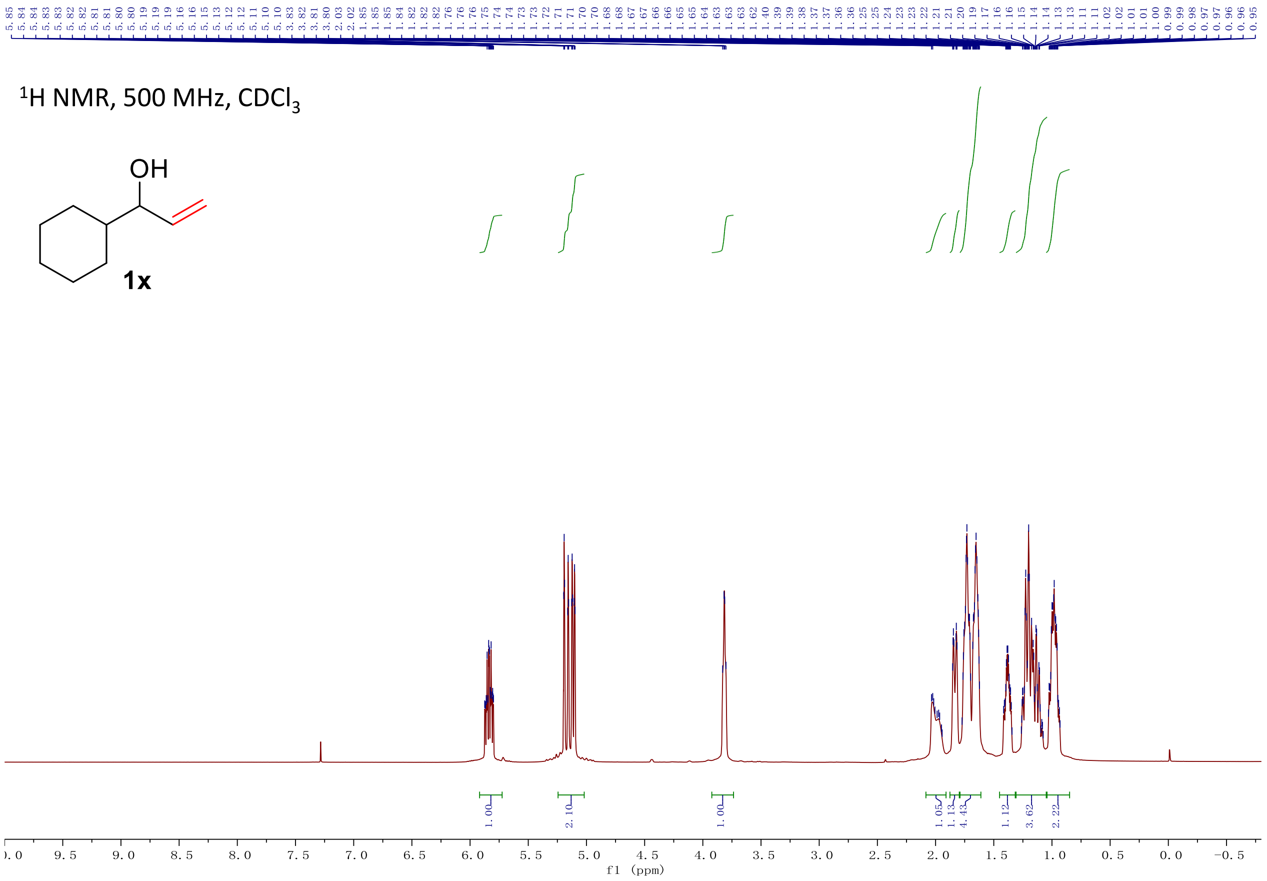


$^{13}C$ {1H} NMR, 126 MHz, $CDCl_3$

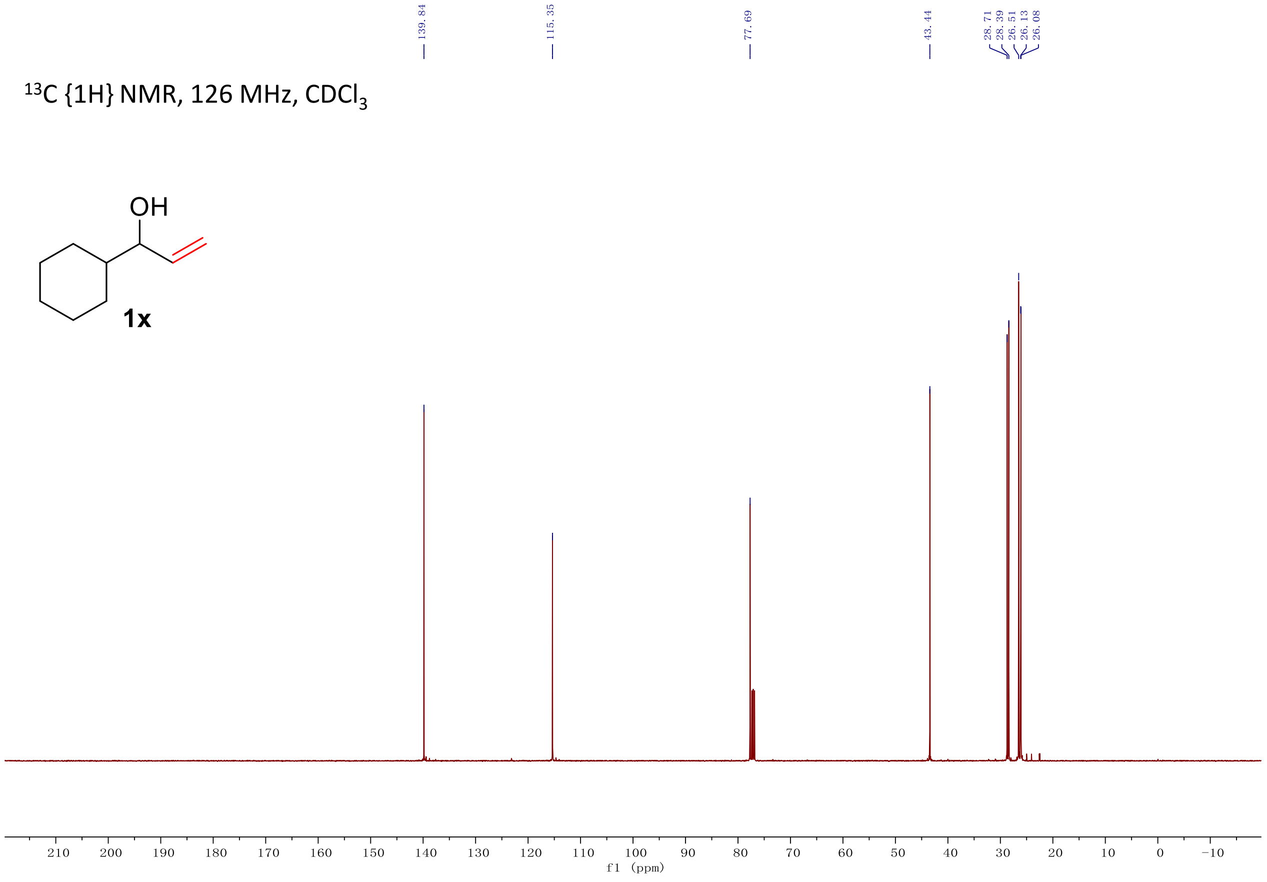

$^1H$ NMR, 500 MHz, $CDCl_3$

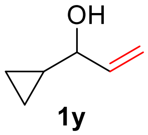

**1y**

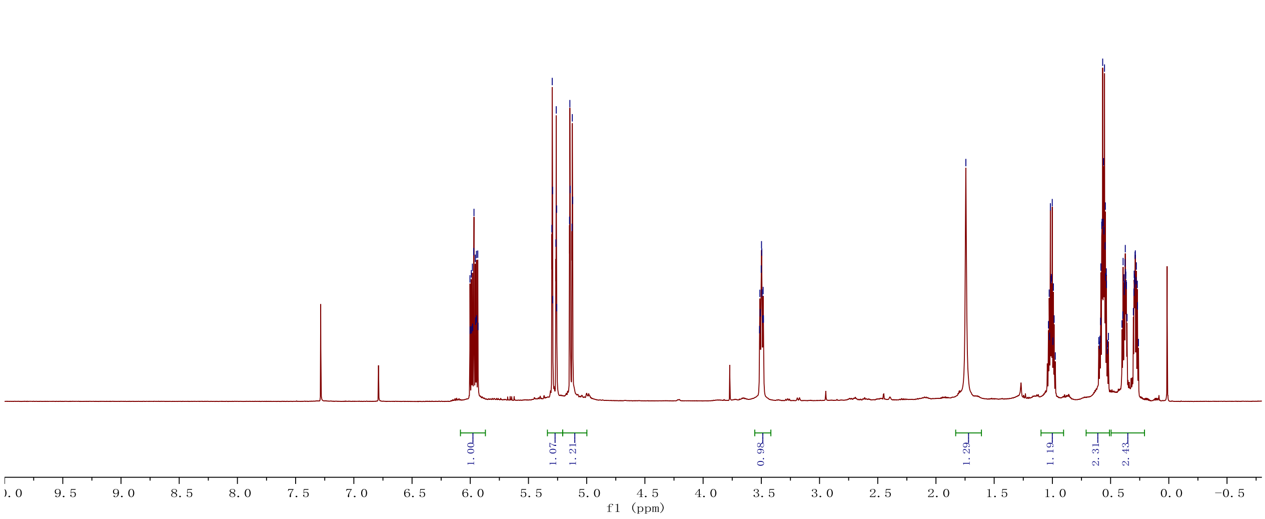


$^{13}C$ {1H} NMR, 126 MHz, $CDCl_3$

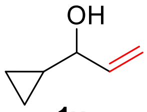

**1y**

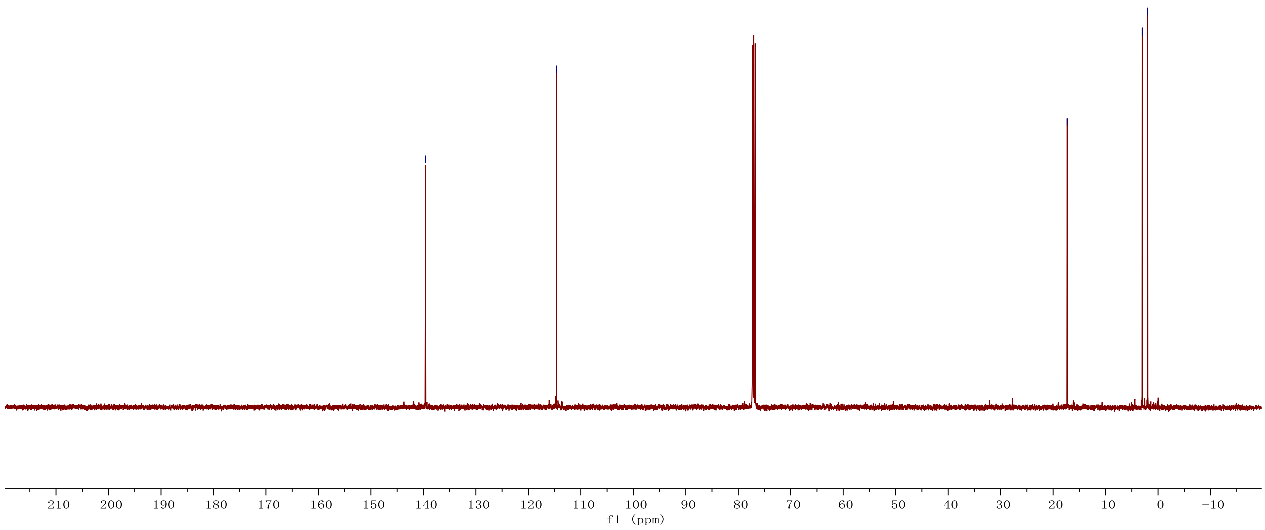

$^{1}H$ NMR, 400 MHz, $CDCl_3$

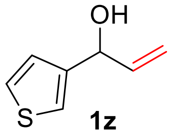

**1z**

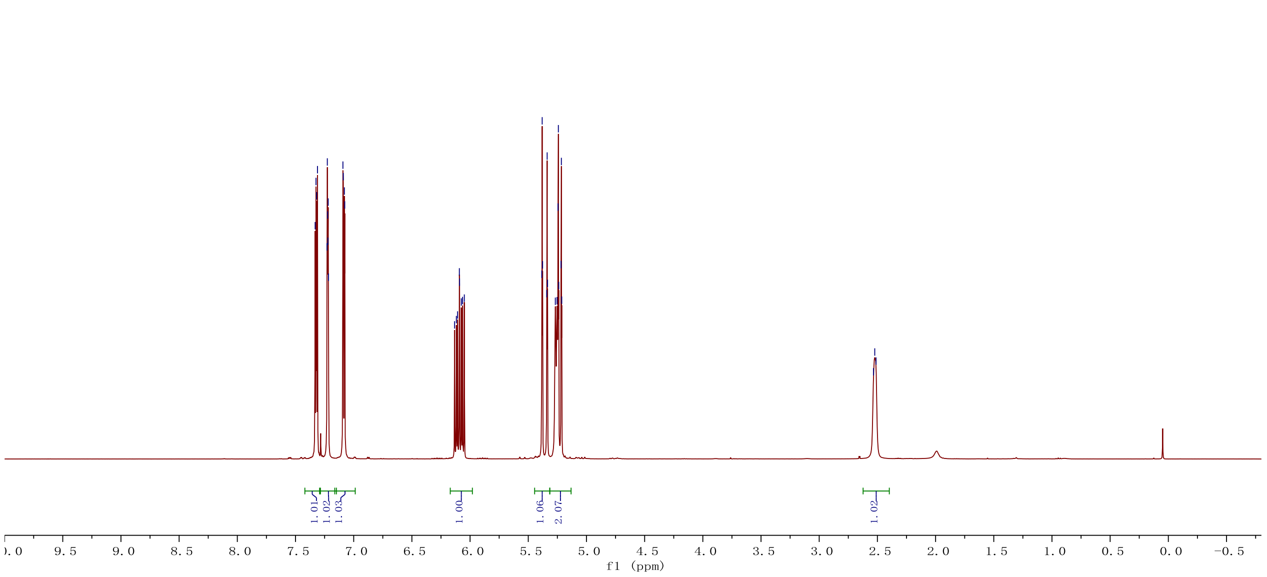


$^{13}C$ {1H} NMR, 101 MHz, $CDCl_3$

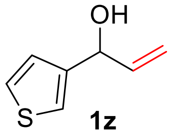

**1z**

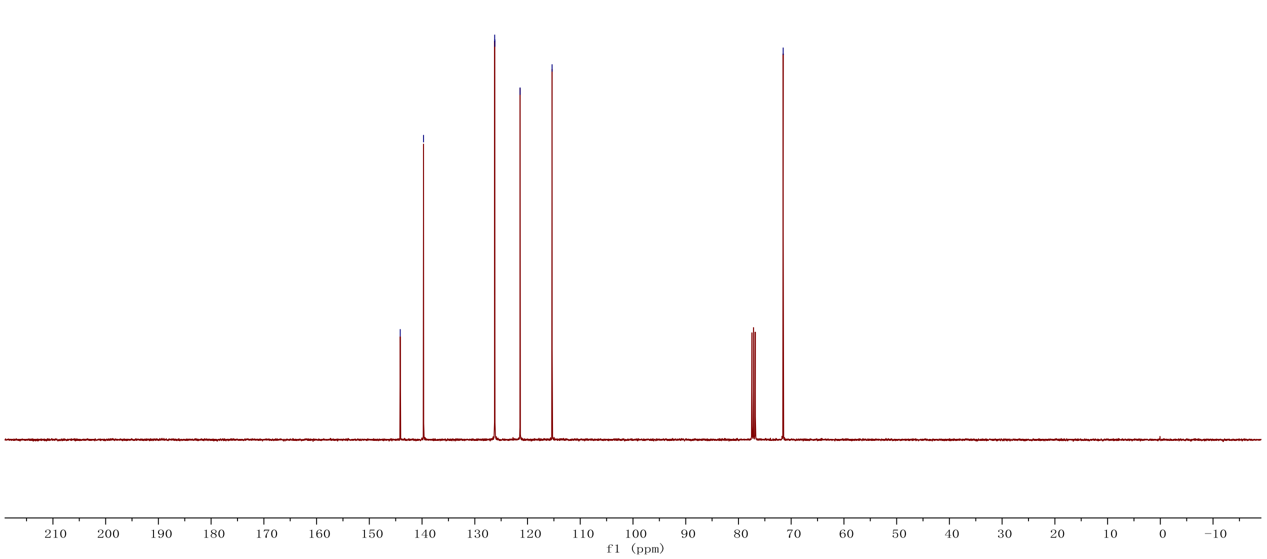

$^{1}H$ NMR, 500 MHz, $CDCl_3$

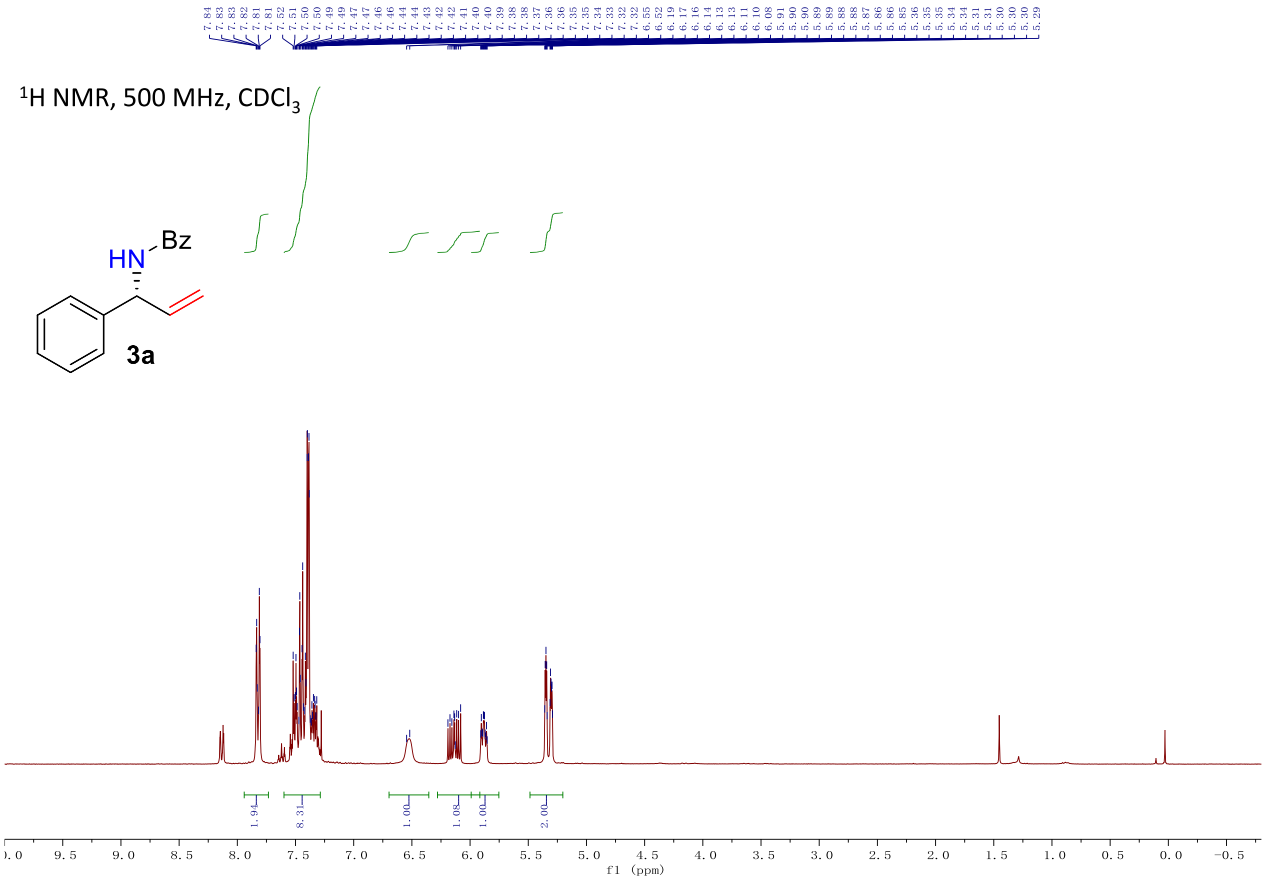


**3a**

$^{13}C$ {1H} NMR, 126 MHz, $CDCl_3$

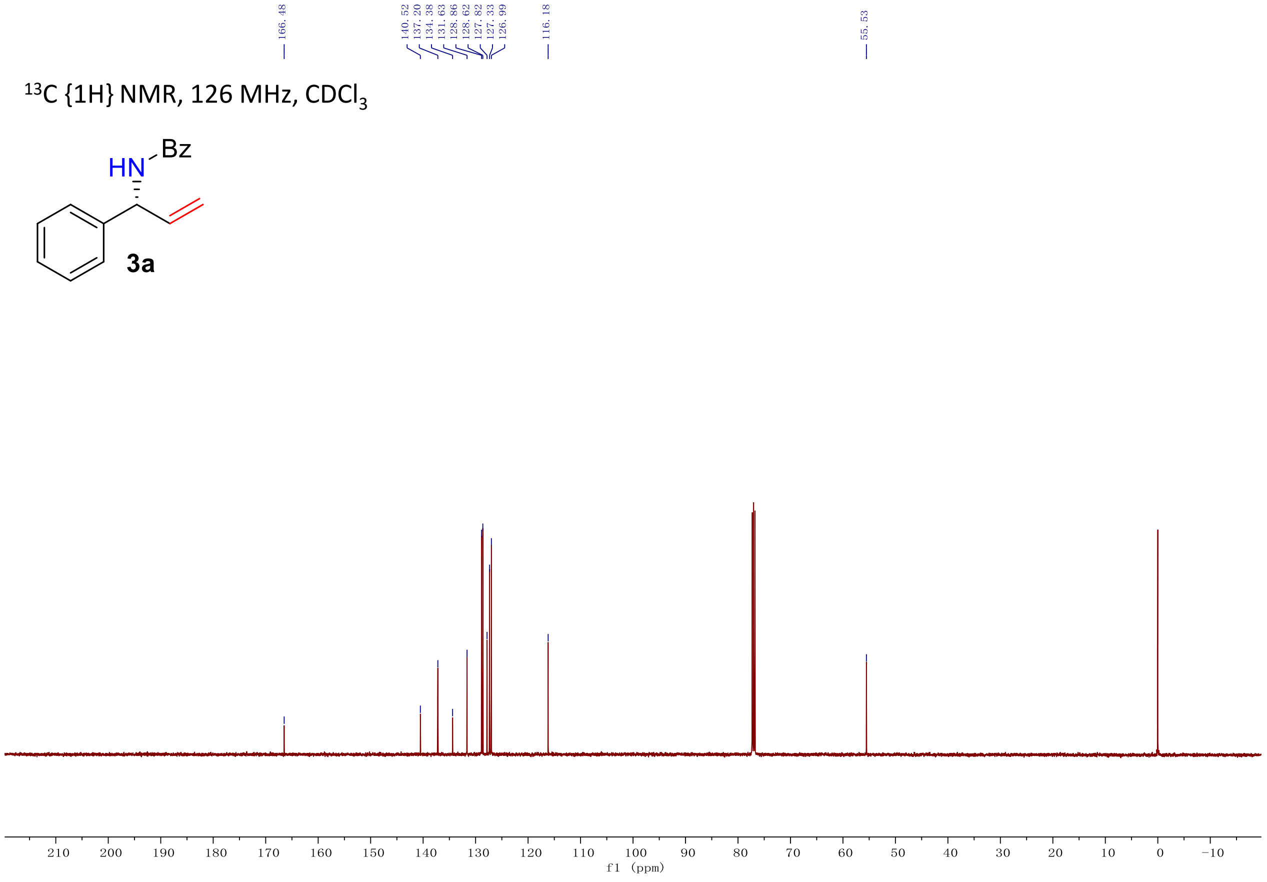


**3a**

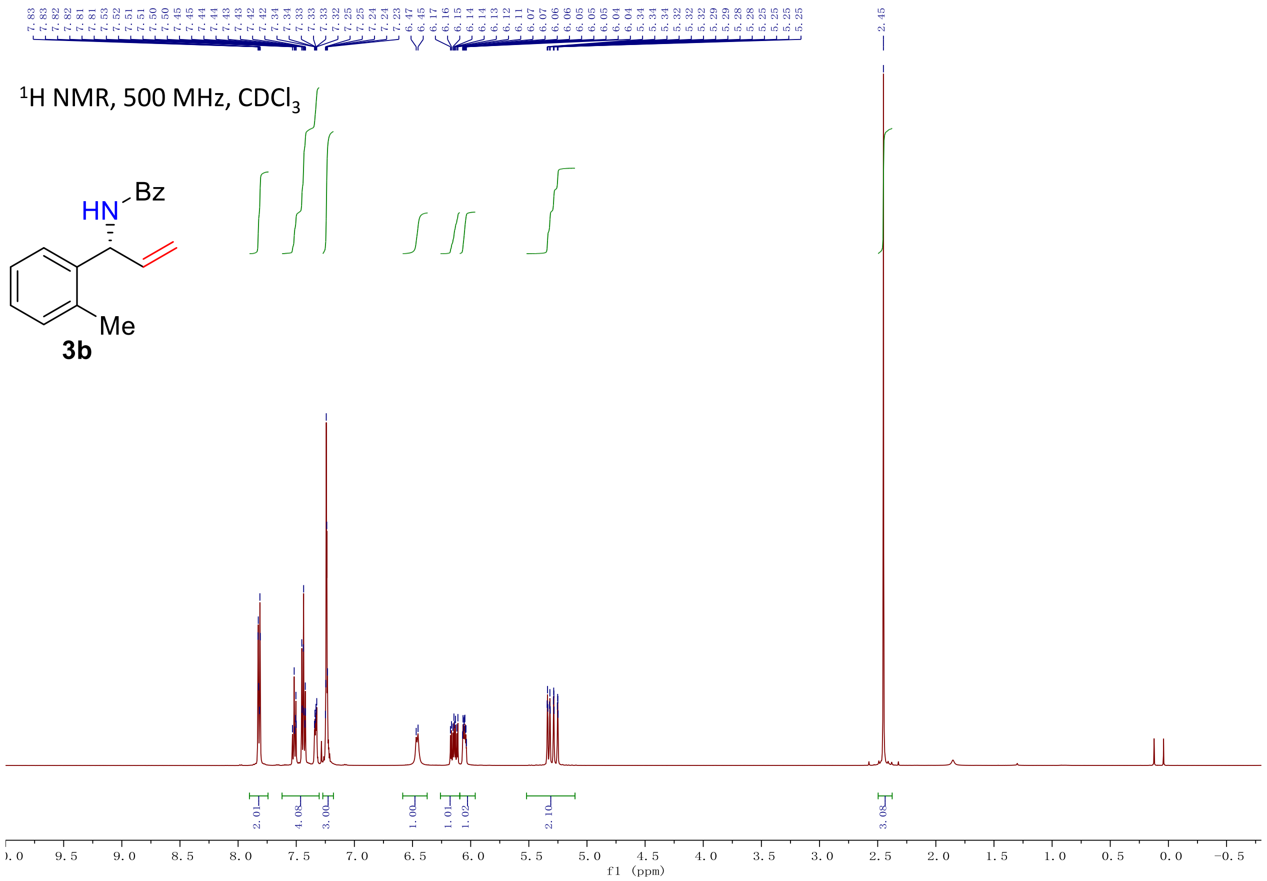

¹H NMR, 500 MHz, CDCl3
HN
Bz
Me
3b
2.45
2.01
4.08
3.00
1.00
1.01
1.02
2.10
3.08
f1 (ppm)


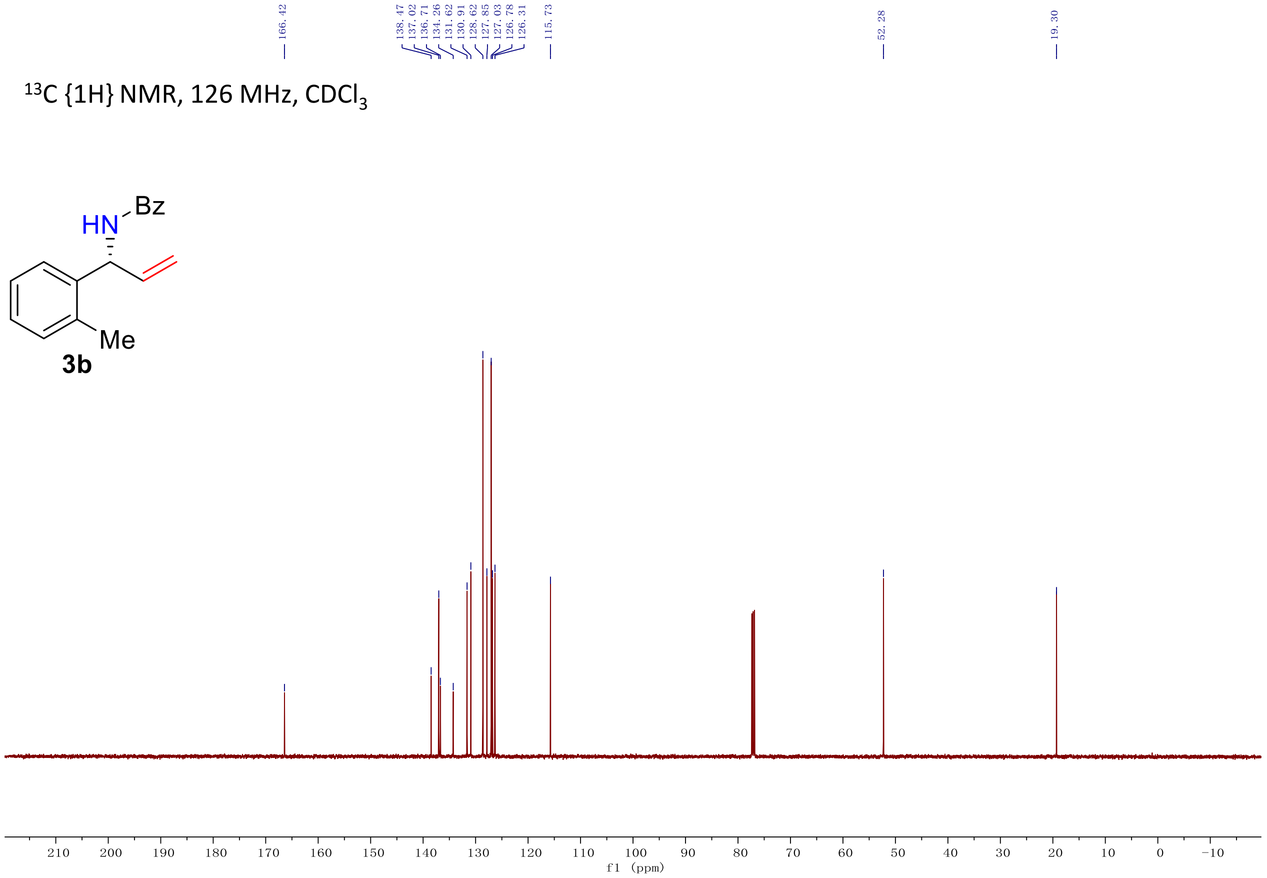

¹³C {1H} NMR, 126 MHz, CDCl3
166.42
138.47
137.02
136.71
134.26
131.62
130.91
128.62
127.85
127.03
126.78
126.31
115.73
52.28
19.30
HN
Bz
Me
3b
f1 (ppm)

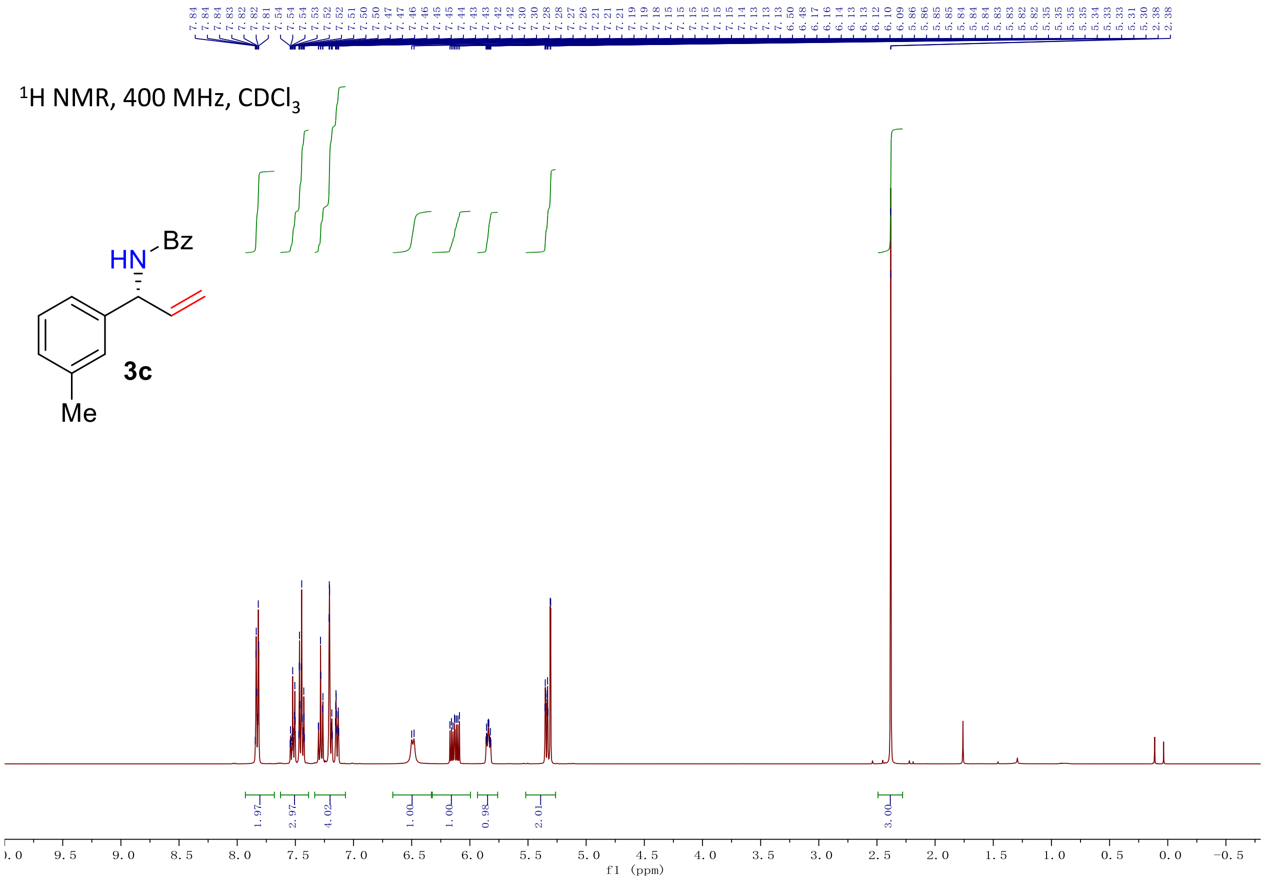

1H NMR, 400 MHz, CDCl3
HN
Bz
3c
Me
f1 (ppm)


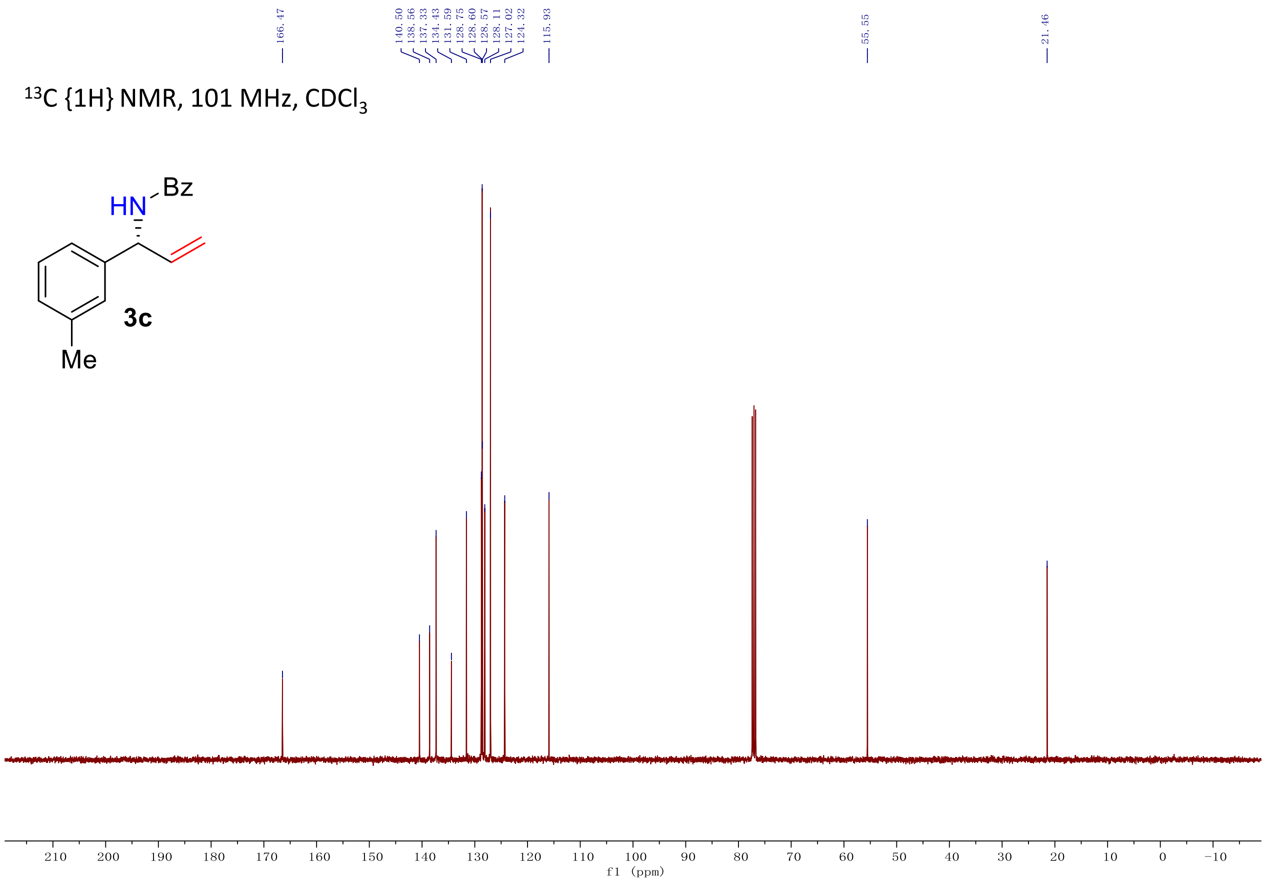

13C {1H} NMR, 101 MHz, CDCl3
HN
Bz
3c
Me
f1 (ppm)

1H NMR, 400 MHz, $CDCl_3$

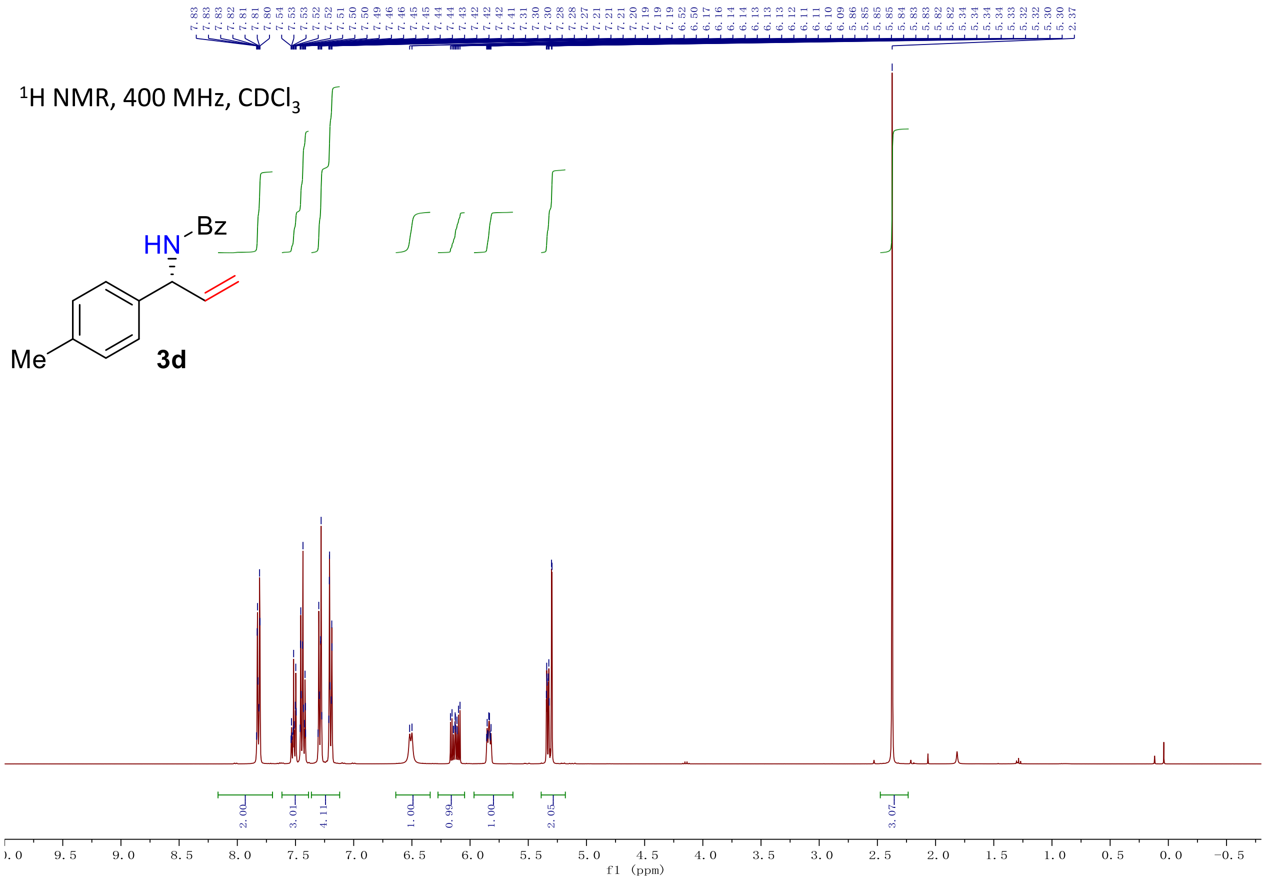

13C {1H} NMR, 101 MHz, $CDCl_3$

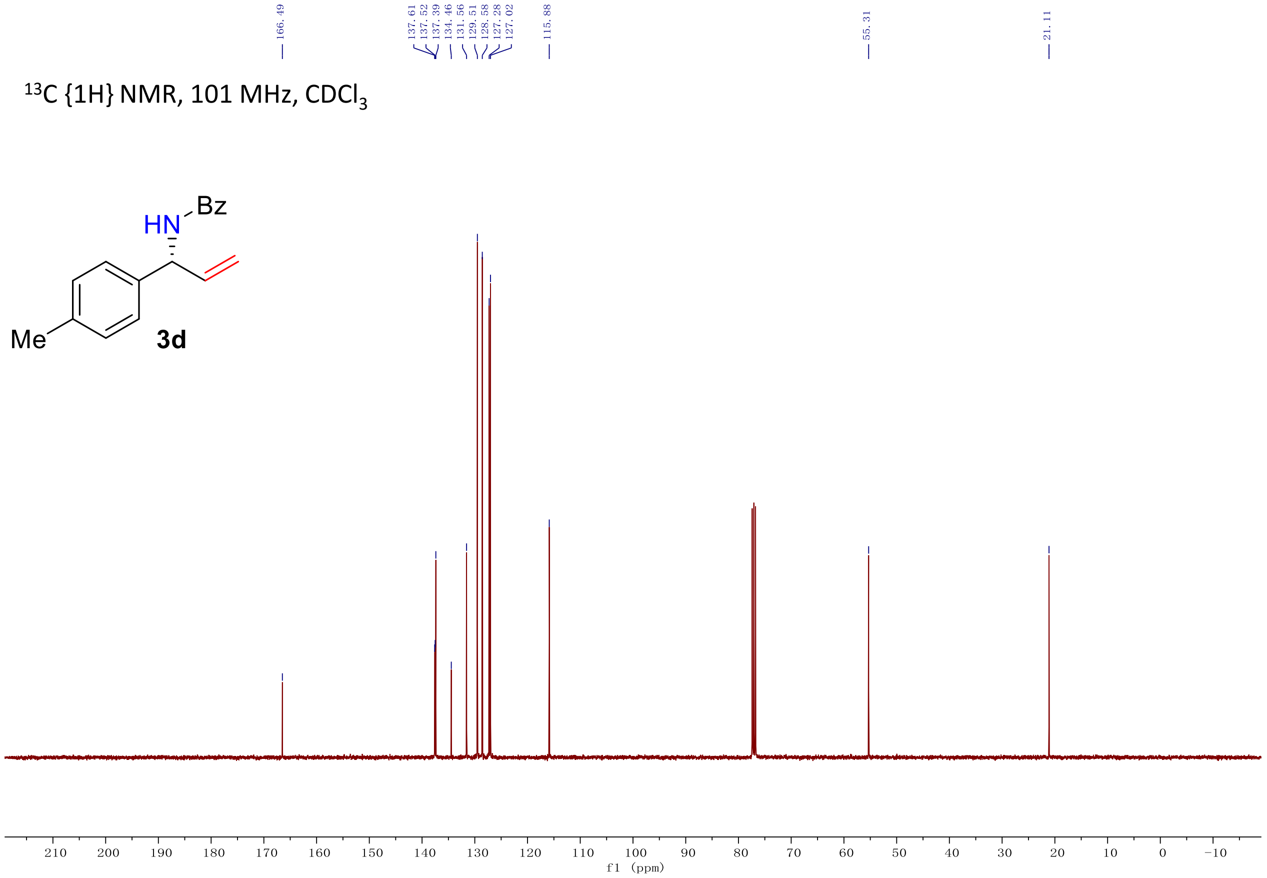

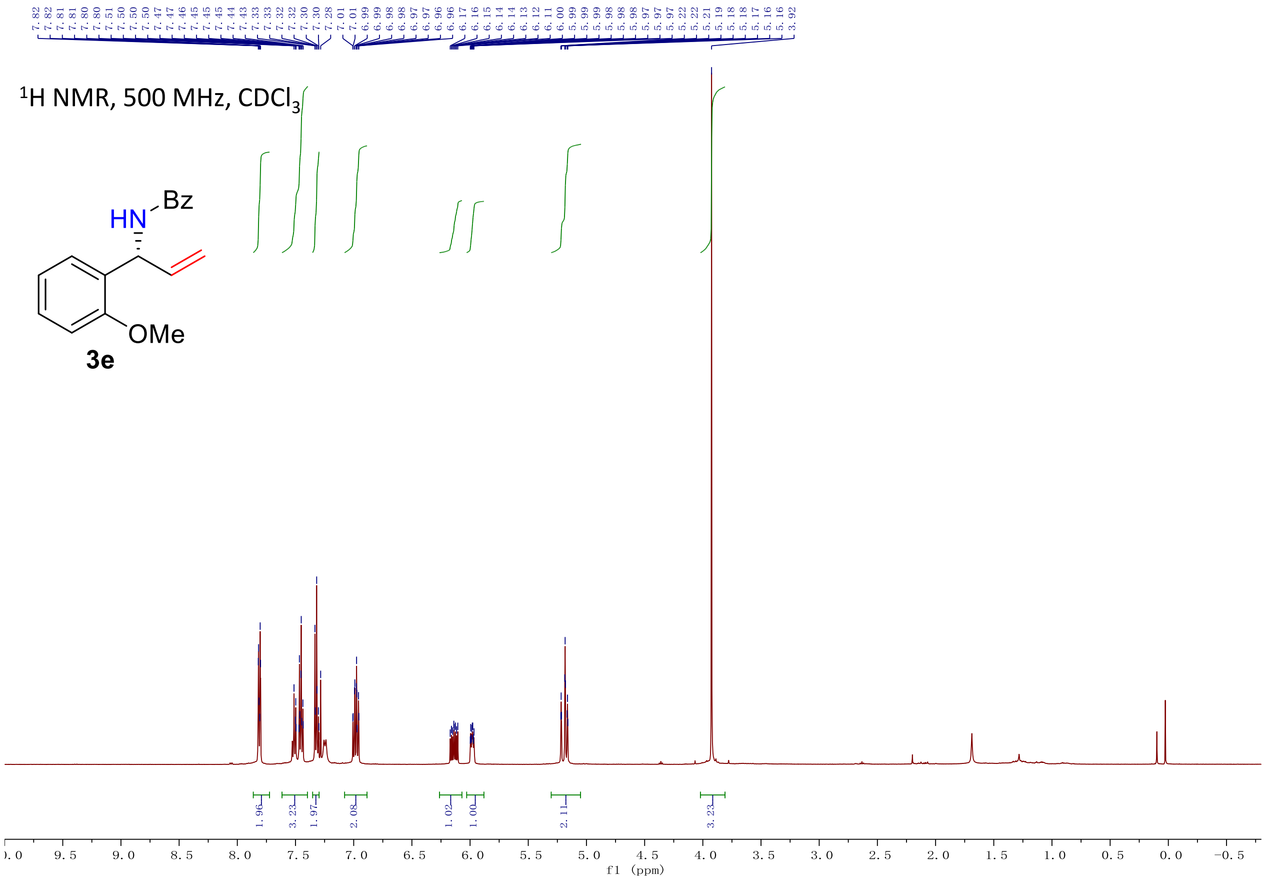

1H NMR, 500 MHz, CDCl3
HN
Bz
OMe
3e
f1 (ppm)


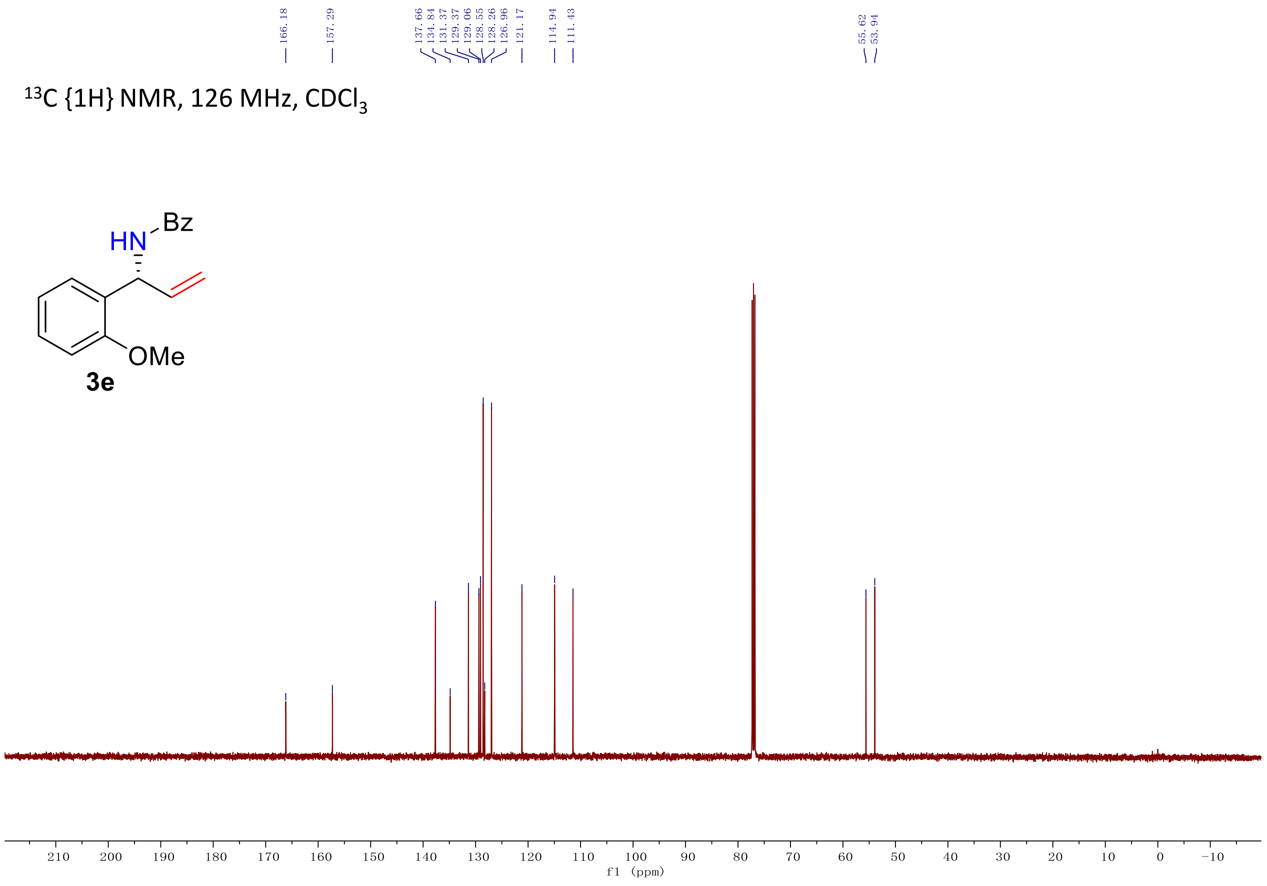

13C {1H} NMR, 126 MHz, CDCl3
HN
Bz
OMe
3e
166.18
157.29
137.66
134.84
131.37
129.37
129.06
128.55
128.26
126.96
121.17
114.94
111.43
55.62
53.94
f1 (ppm)

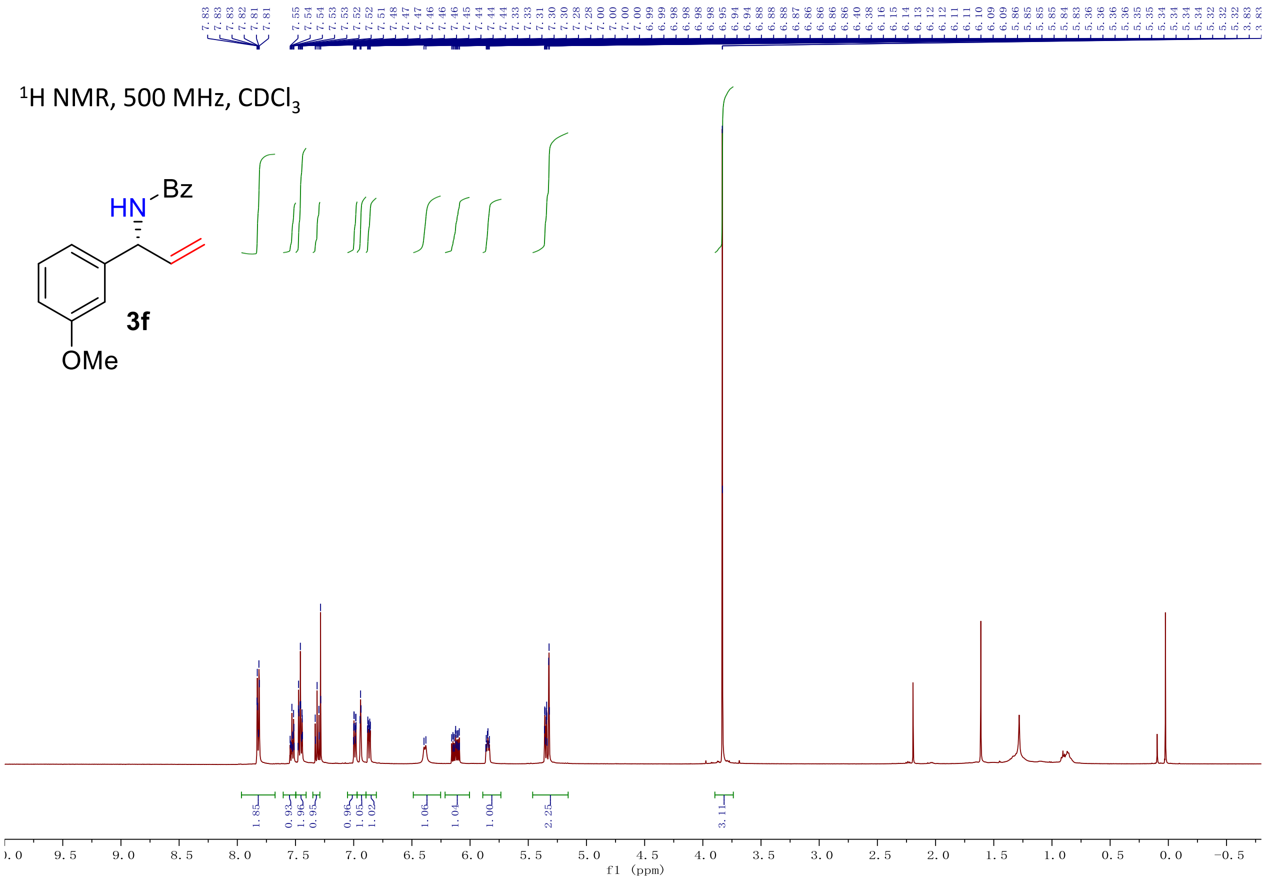

1H NMR, 500 MHz, CDCl3
HN
Bz
3f
OMe
1.85
0.93
1.96
0.95
0.96
1.05
1.02
1.06
1.04
1.00
2.25
3.11
f1 (ppm)


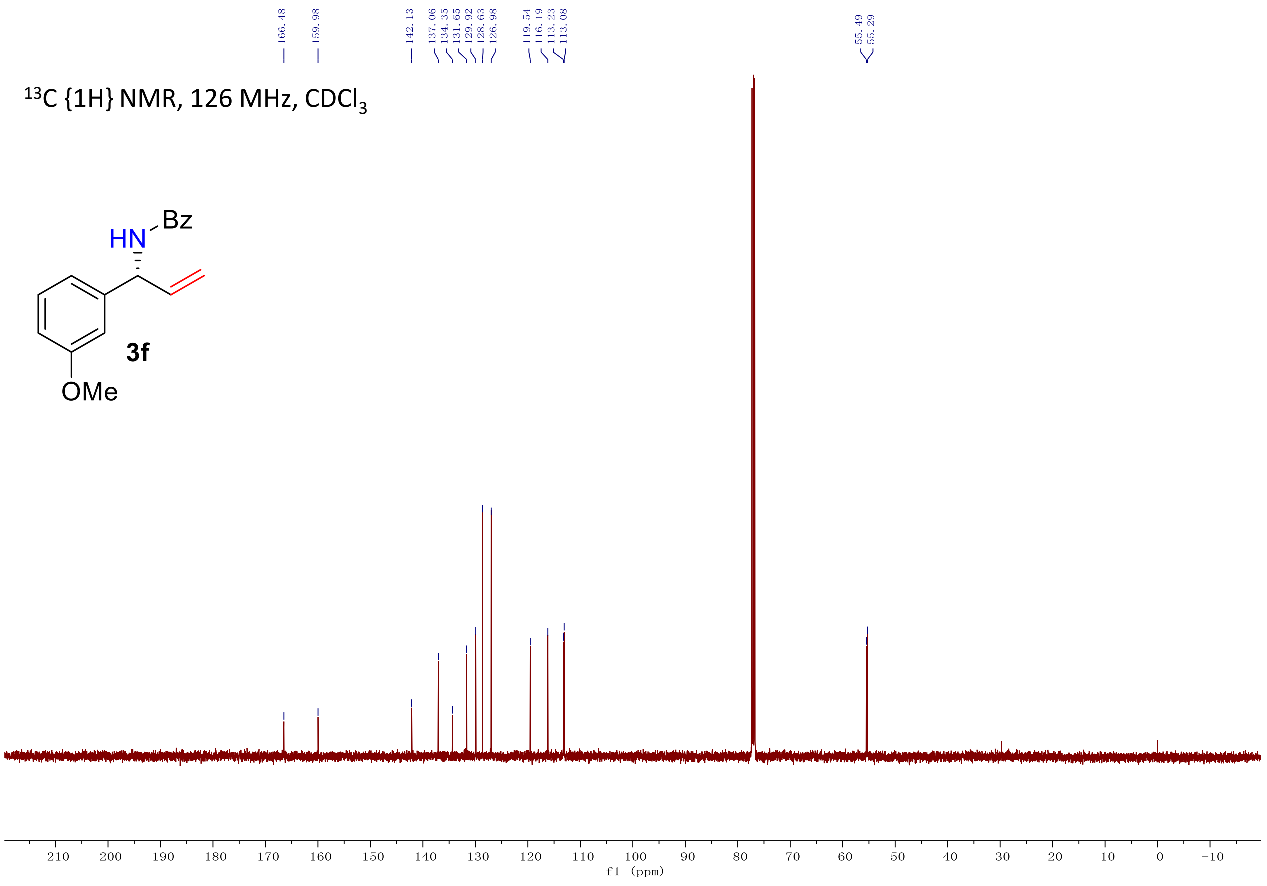

166.48
159.98
142.13
137.06
134.35
131.65
129.92
128.63
126.98
119.54
116.19
113.23
113.08
55.49
55.29
13C {1H} NMR, 126 MHz, CDCl3
HN
Bz
3f
OMe
f1 (ppm)

$^{1}H$ NMR, 500 MHz, $CDCl_3$

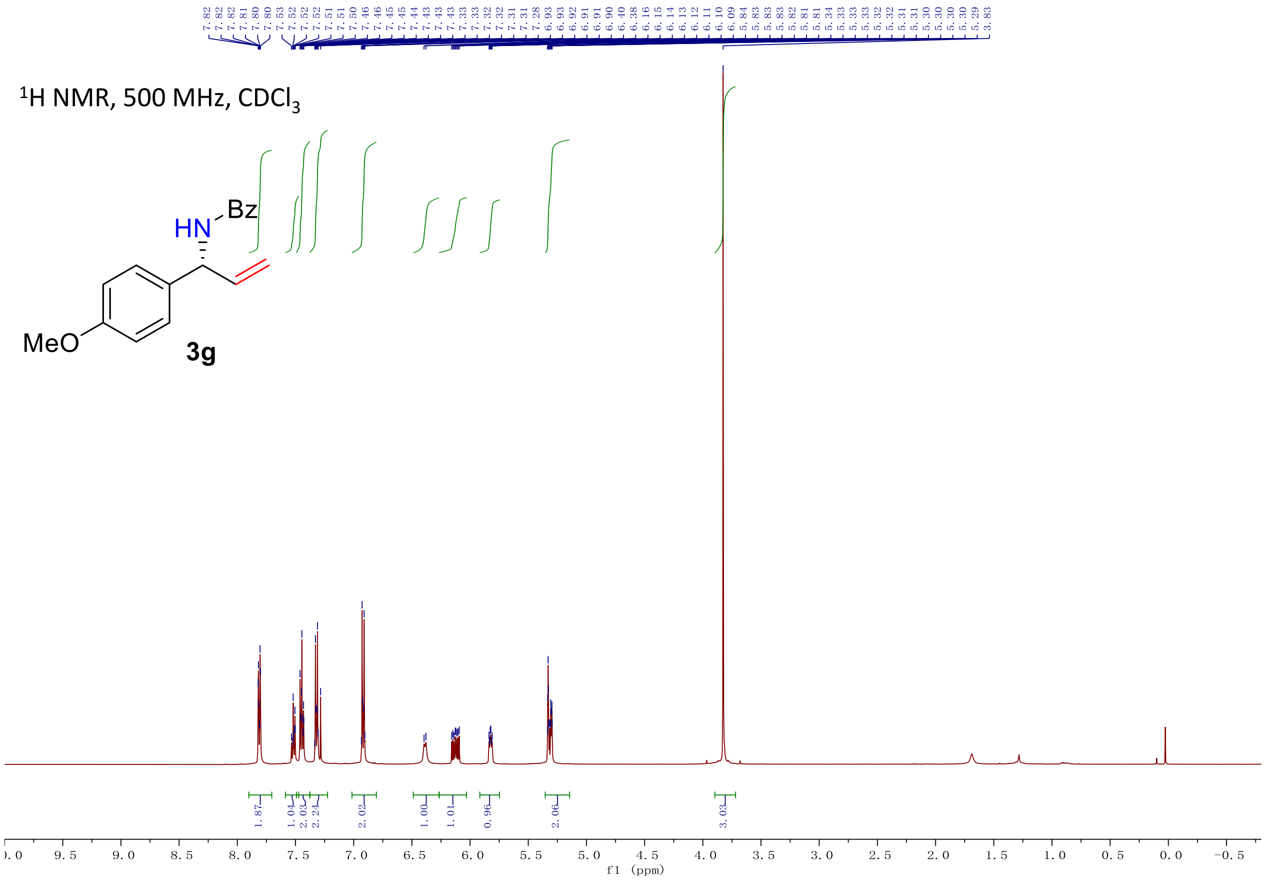


3g

$^{13}C$ {1H} NMR, 126 MHz, $CDCl_3$

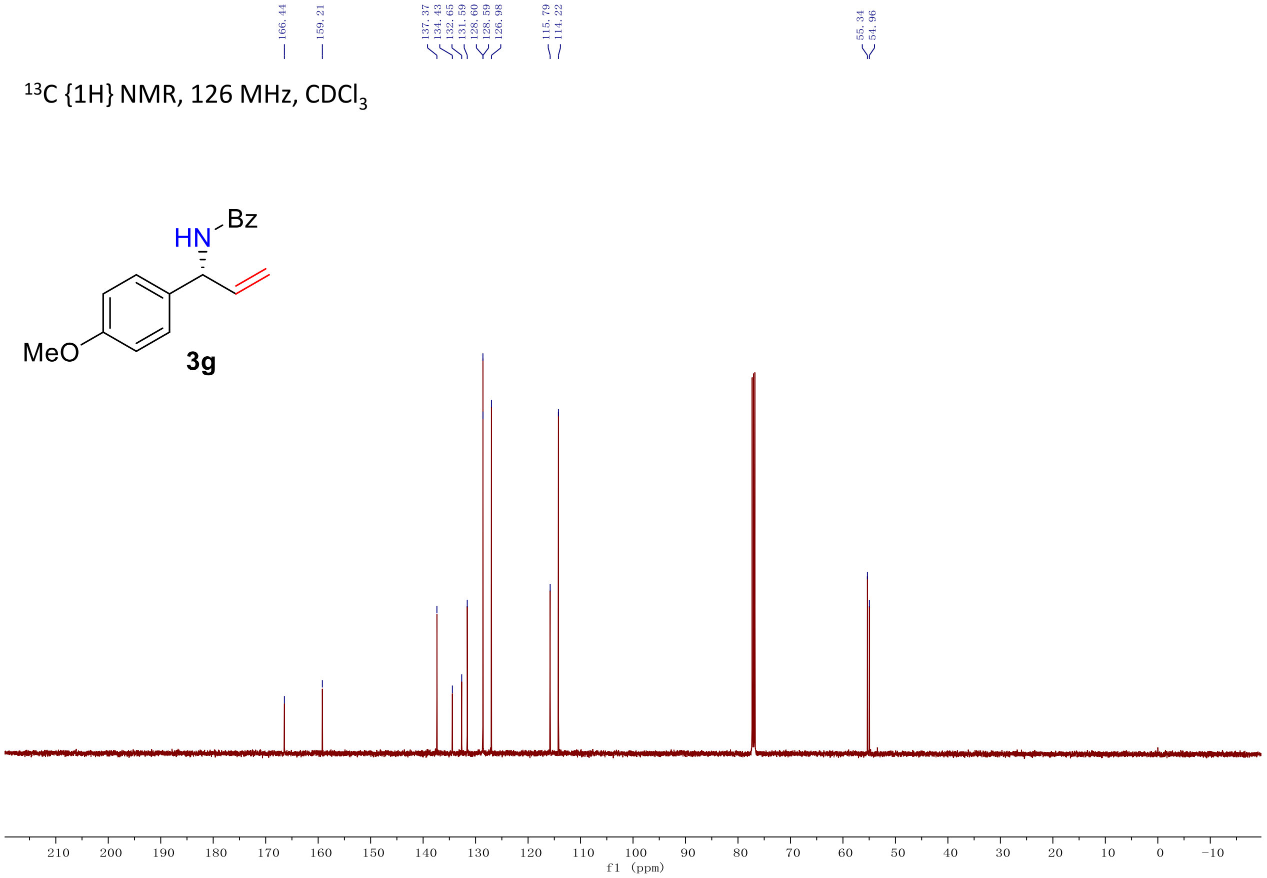


3g

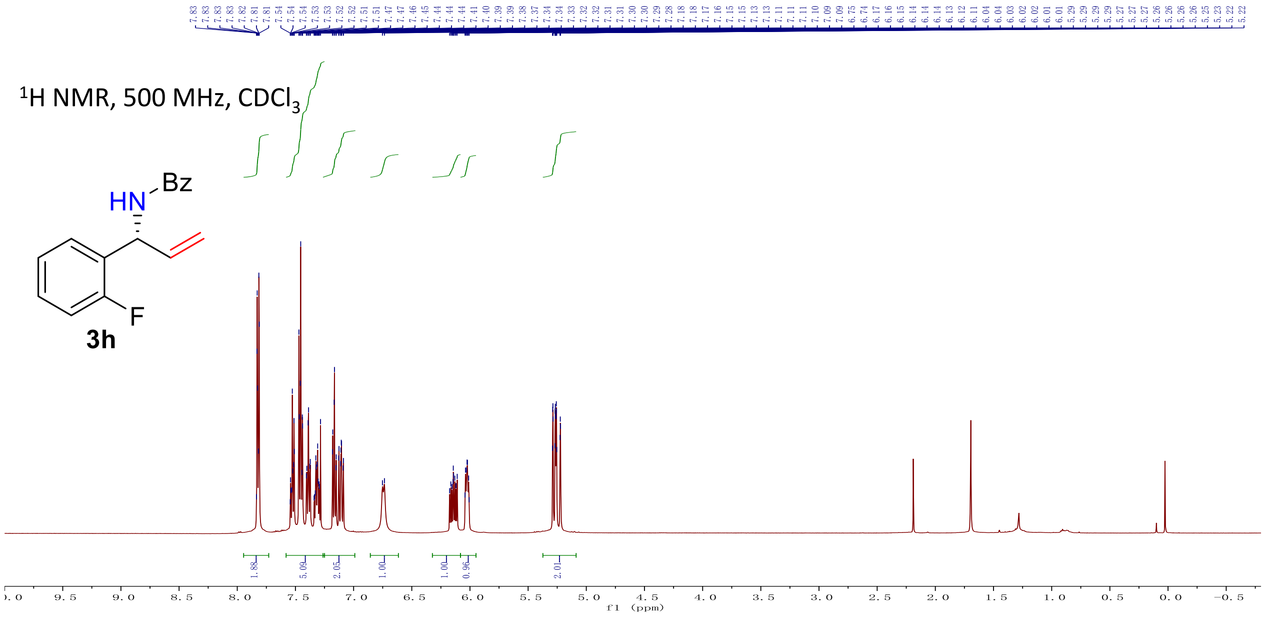
1H NMR, 500 MHz, CDCl3
HN
Bz
F
3h

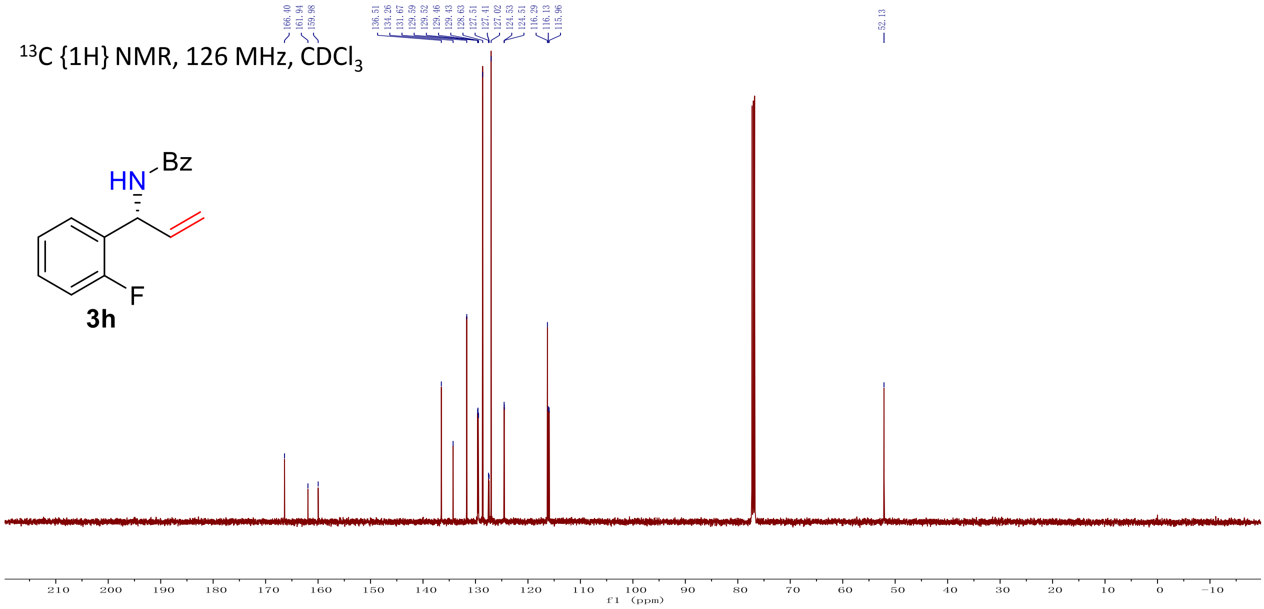
13C {1H} NMR, 126 MHz, CDCl3
166.40
161.94
159.98
136.51
134.26
131.67
129.59
129.52
129.46
129.43
128.63
127.51
127.41
127.02
124.53
124.51
116.29
116.13
115.96
52.13
HN
Bz
F
3h

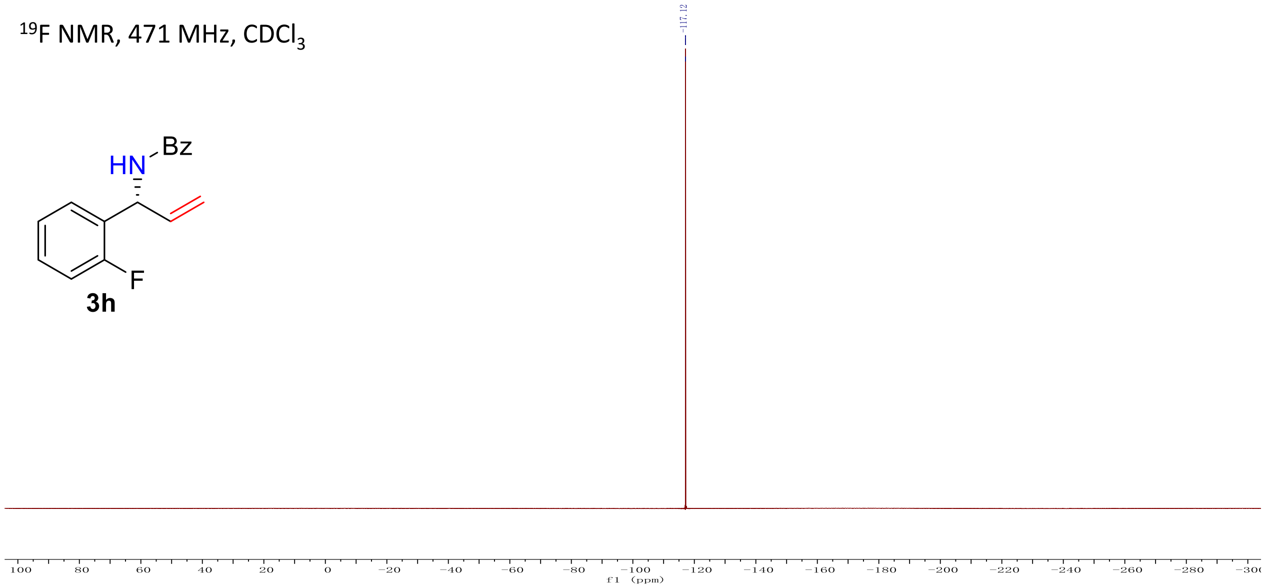
19F NMR, 471 MHz, CDCl3
-117.12
HN
Bz
F
3h

$^{1}$H NMR, 500 MHz, $CDCl_3$

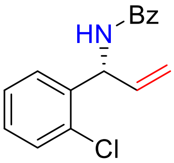


**3i**

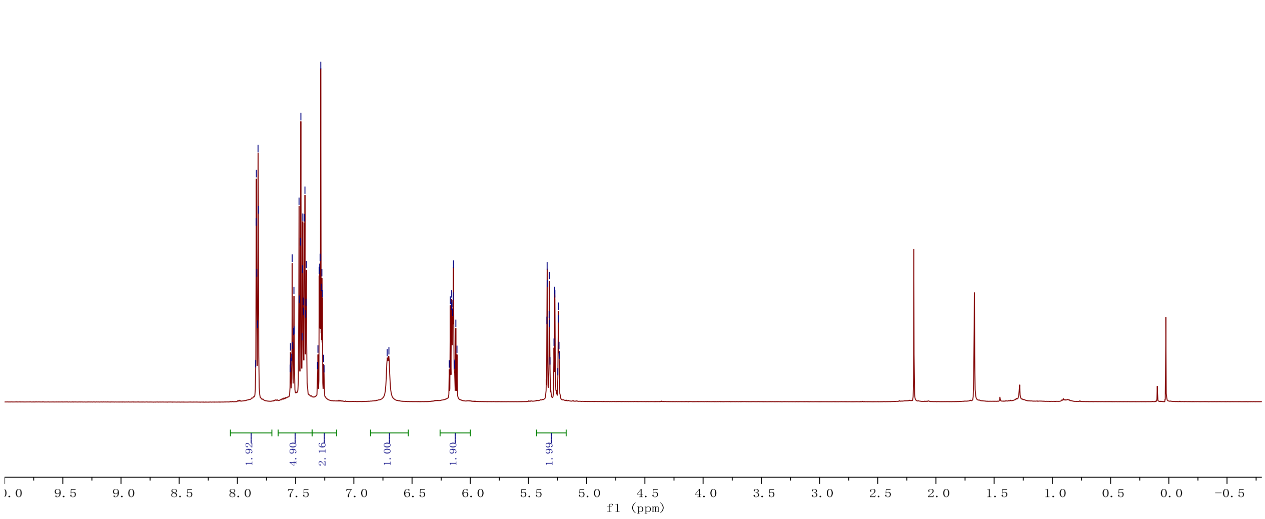


$^{13}$C {1H} NMR, 126 MHz, $CDCl_3$

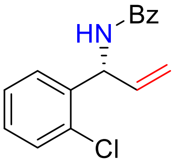


**3i**

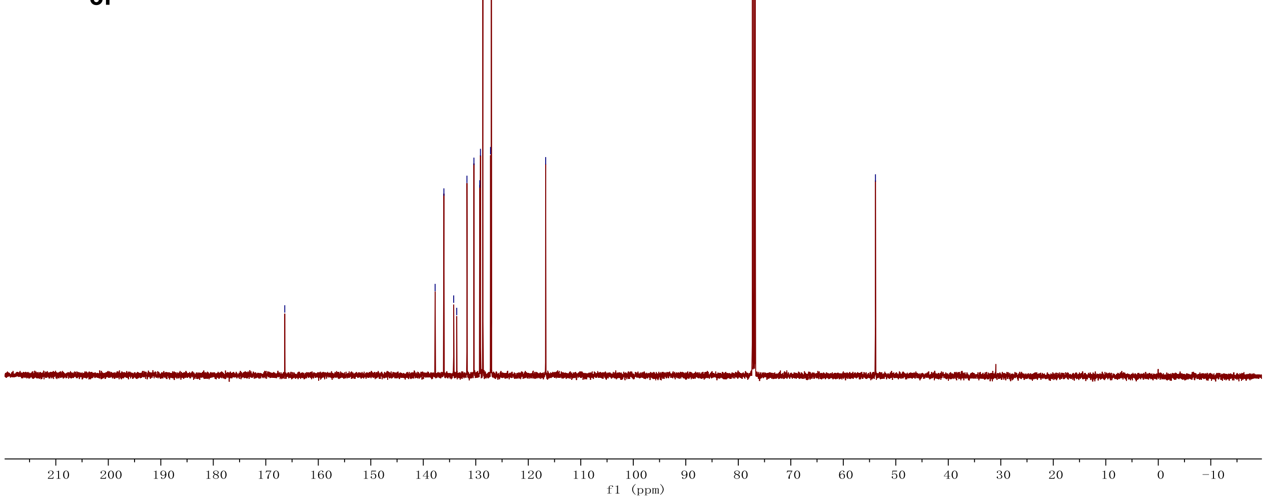

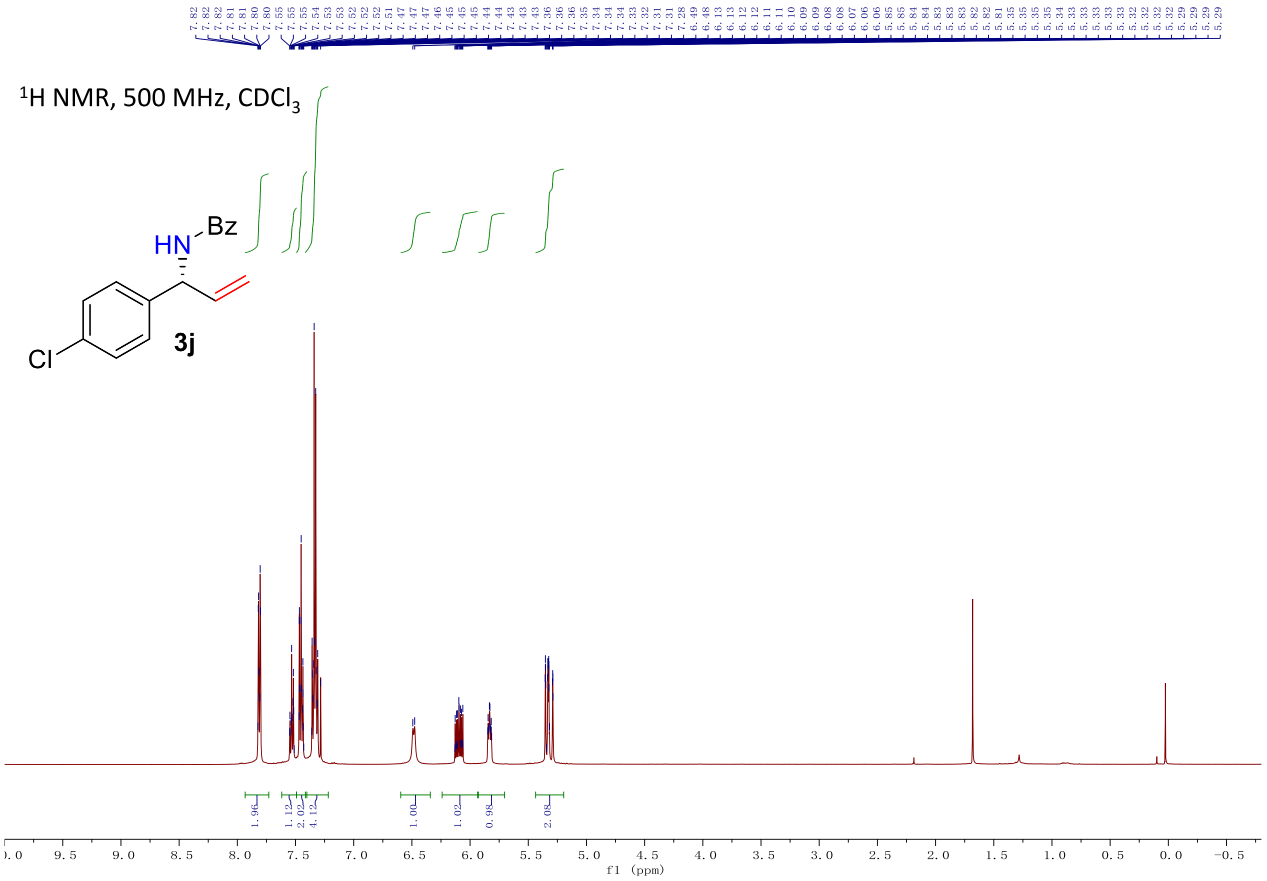
¹H NMR, 500 MHz, CDCl3
HN
Bz
Cl
3j
1.96
1.12
2.02
4.12
1.00
1.02
0.98
2.08
f1 (ppm)

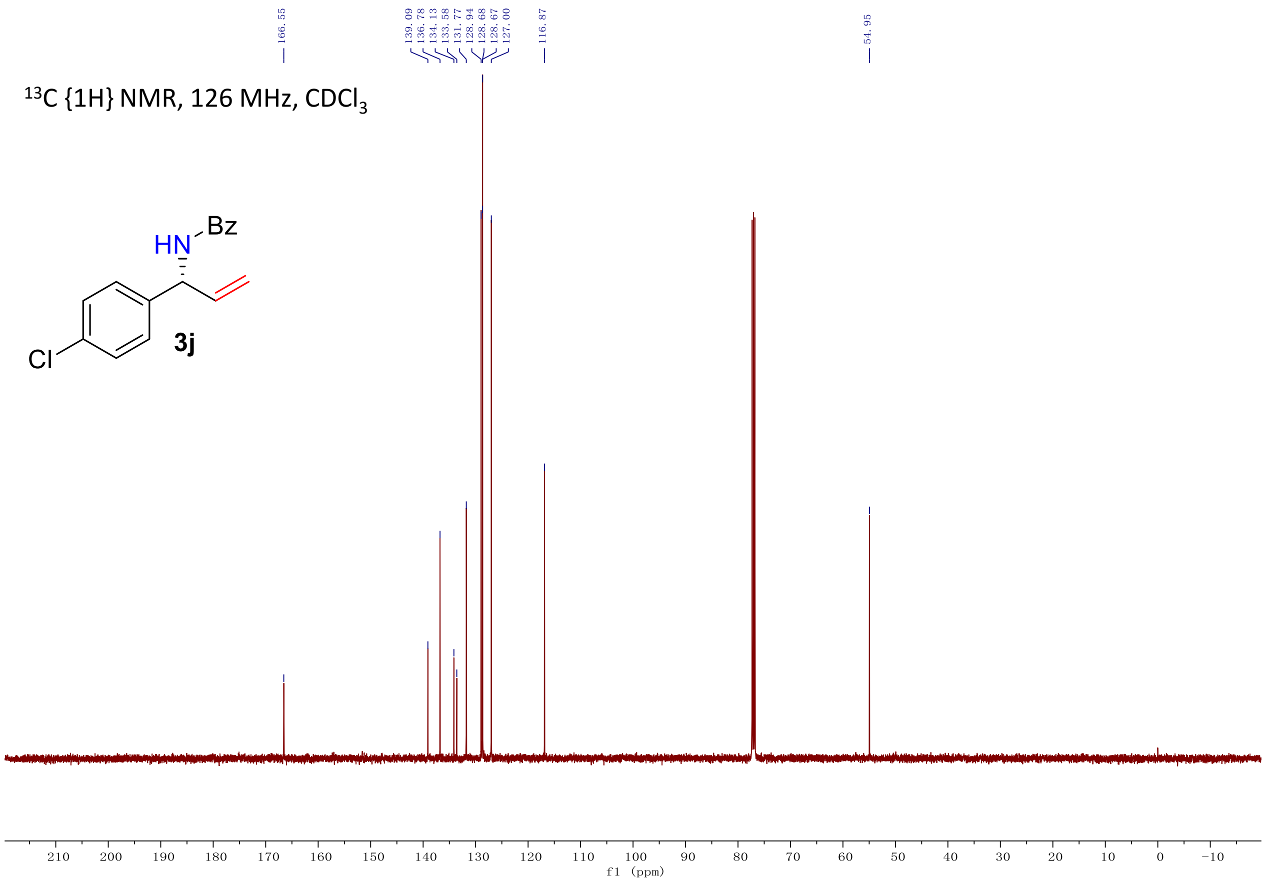
¹³C {1H} NMR, 126 MHz, CDCl3
166.55
139.09
136.78
134.13
133.58
131.77
128.94
128.68
128.67
127.00
116.87
54.95
HN
Bz
Cl
3j
f1 (ppm)

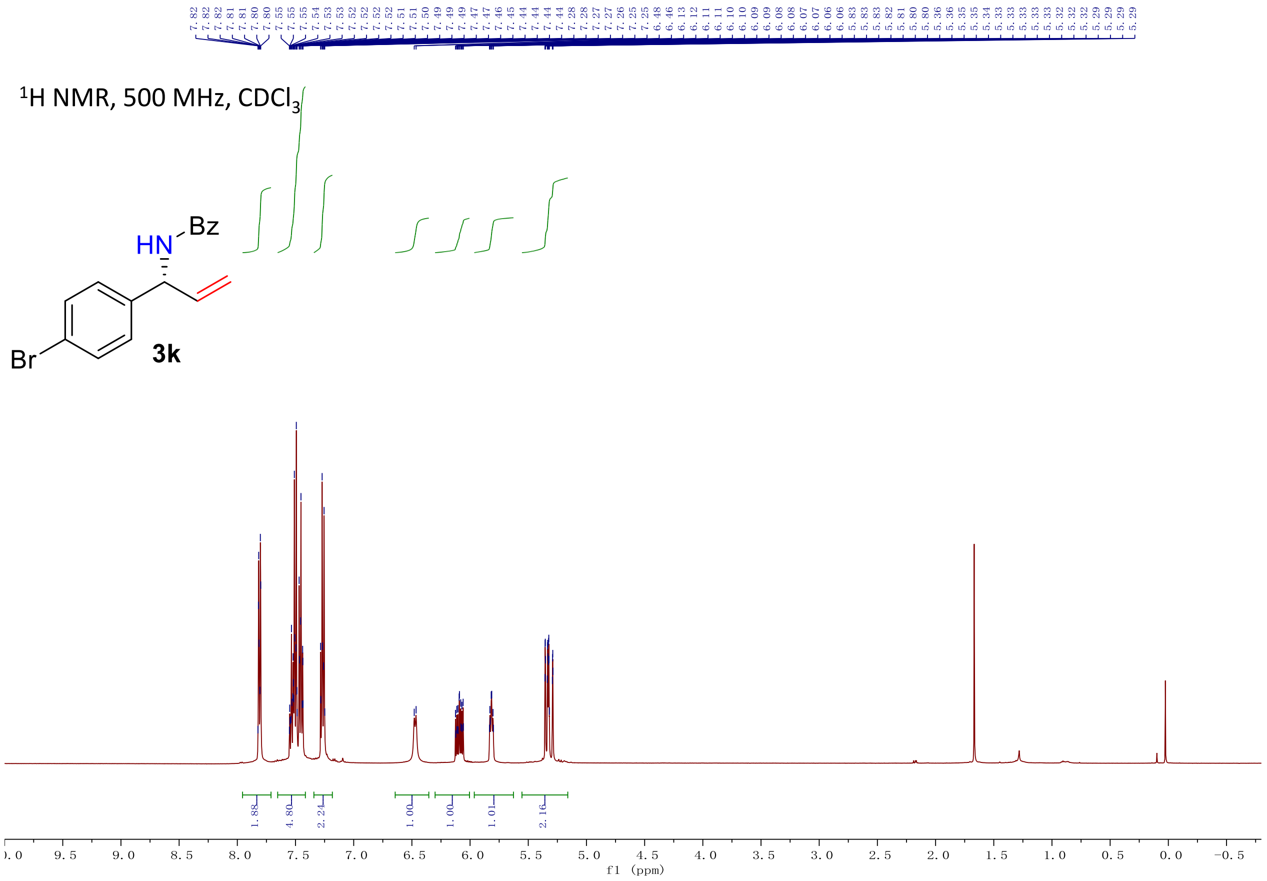

1H NMR, 500 MHz, CDCl3
HN
Bz
Br
3k
1.88
4.80
2.24
1.00
1.00
1.01
2.16
f1 (ppm)


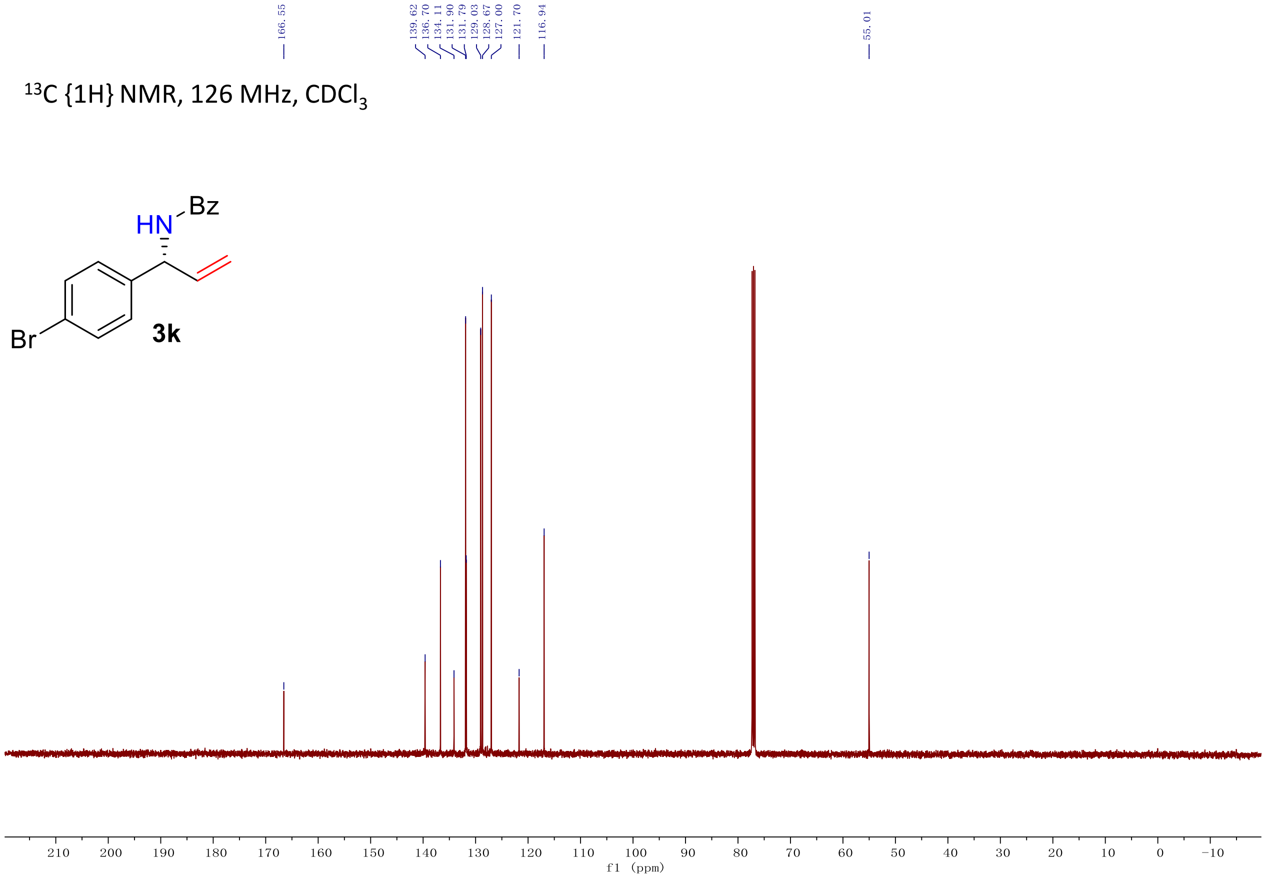

166.55
139.62
136.70
134.11
131.90
131.79
129.03
128.67
127.00
121.70
116.94
55.01
13C {1H} NMR, 126 MHz, CDCl3
HN
Bz
Br
3k
f1 (ppm)

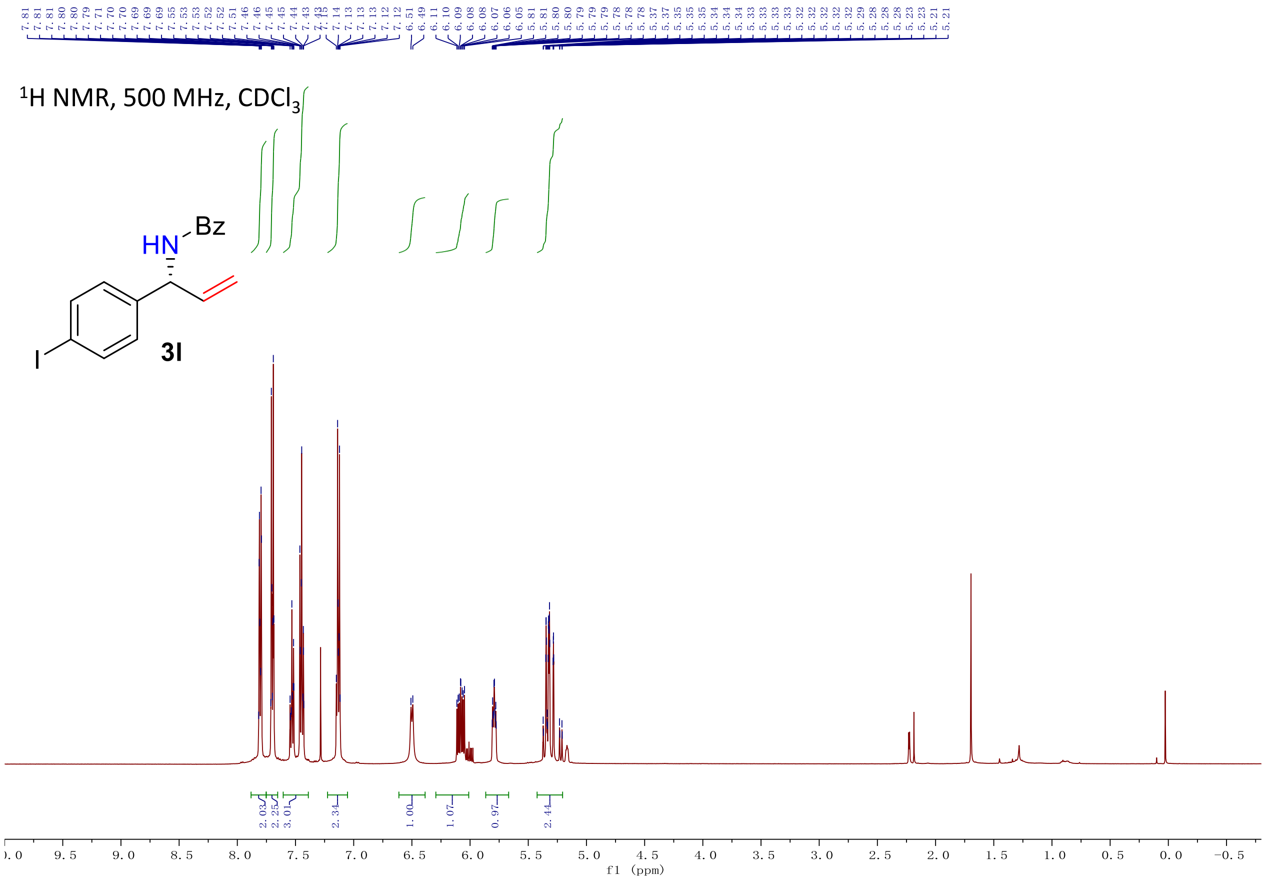

1H NMR, 500 MHz, CDCl3
HN
Bz
I
3l
f1 (ppm)


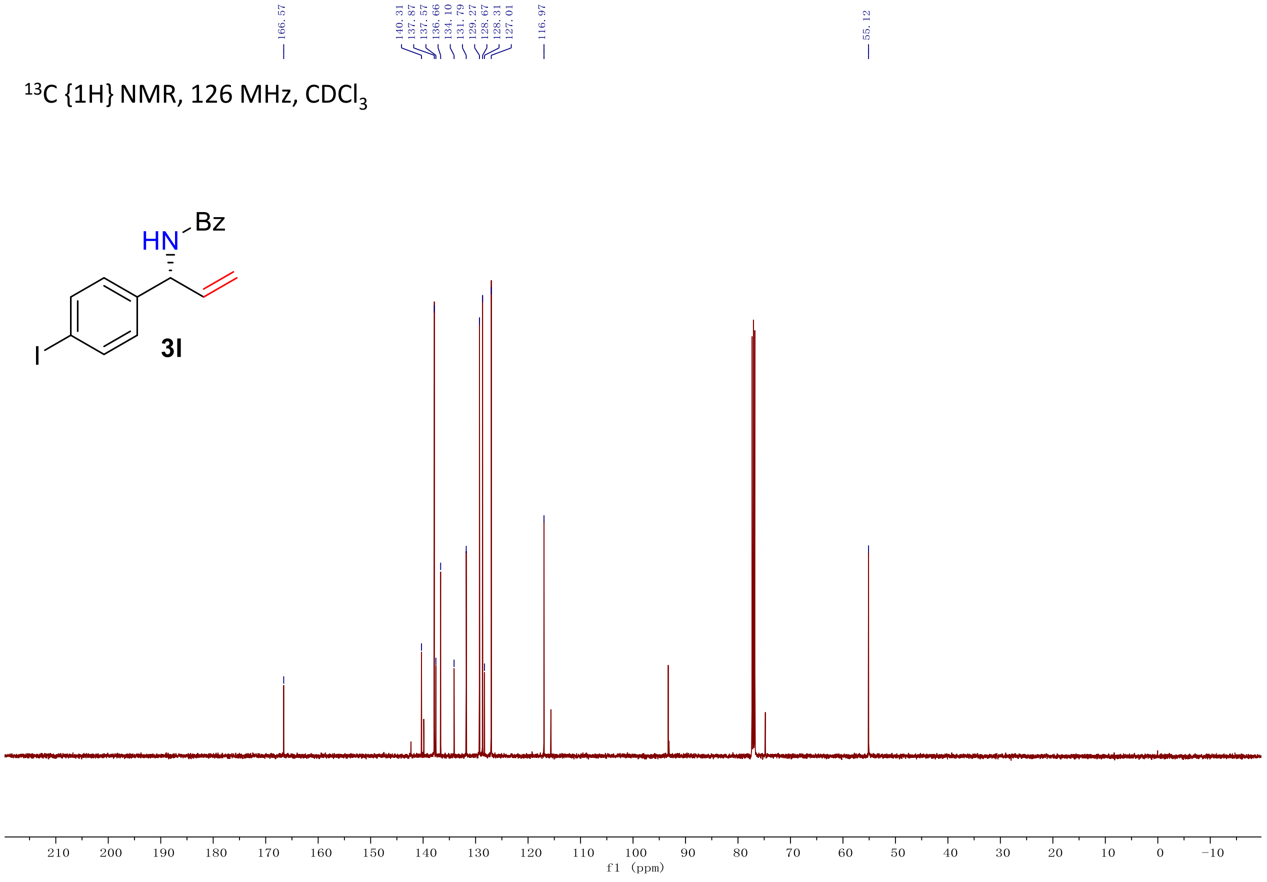

13C {1H} NMR, 126 MHz, CDCl3
HN
Bz
I
3l
166.57
140.31
137.87
137.57
136.66
134.10
131.79
129.27
128.67
128.31
127.01
116.97
55.12
f1 (ppm)

$^{1}H$ NMR, 500 MHz, $CDCl_3$

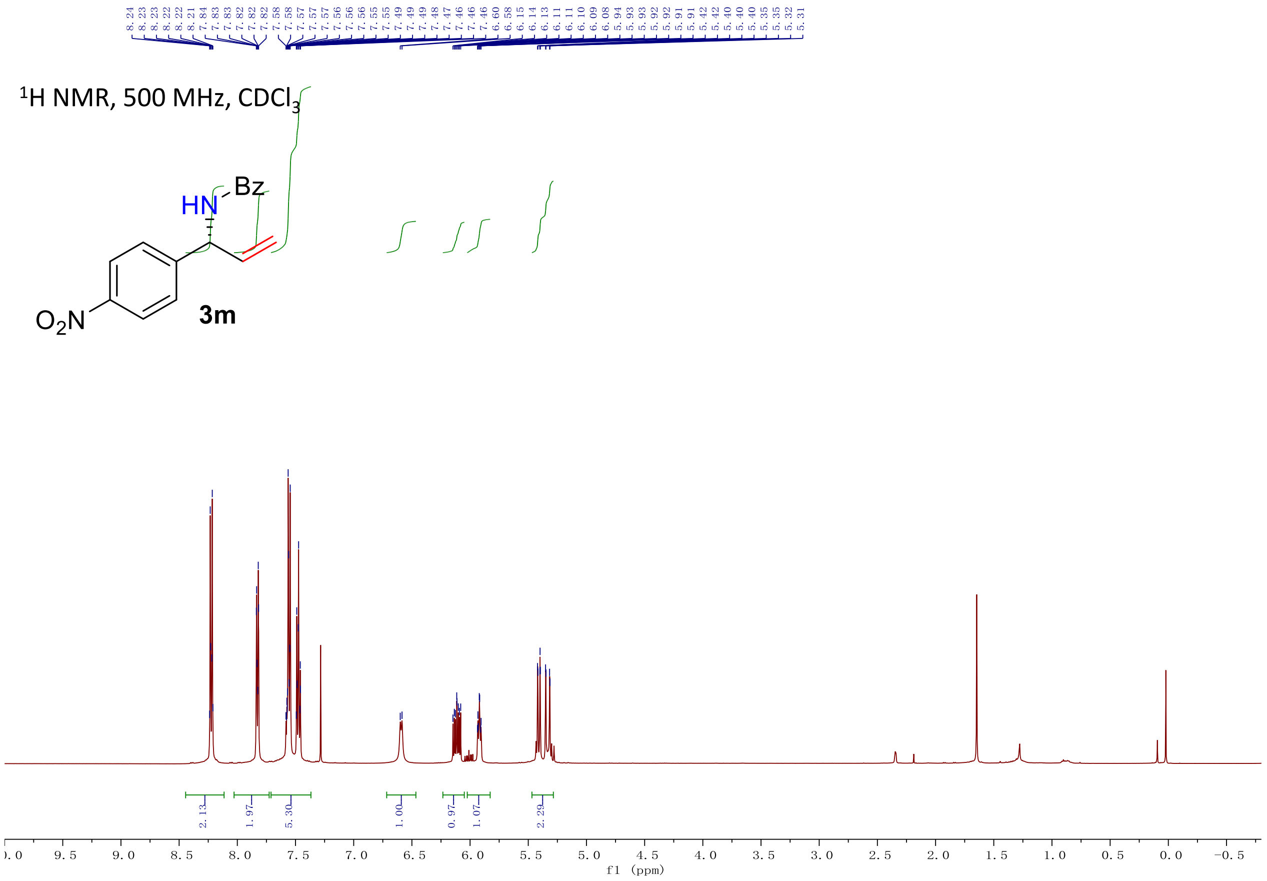


$^{13}C$ {1H} NMR, 126 MHz, $CDCl_3$

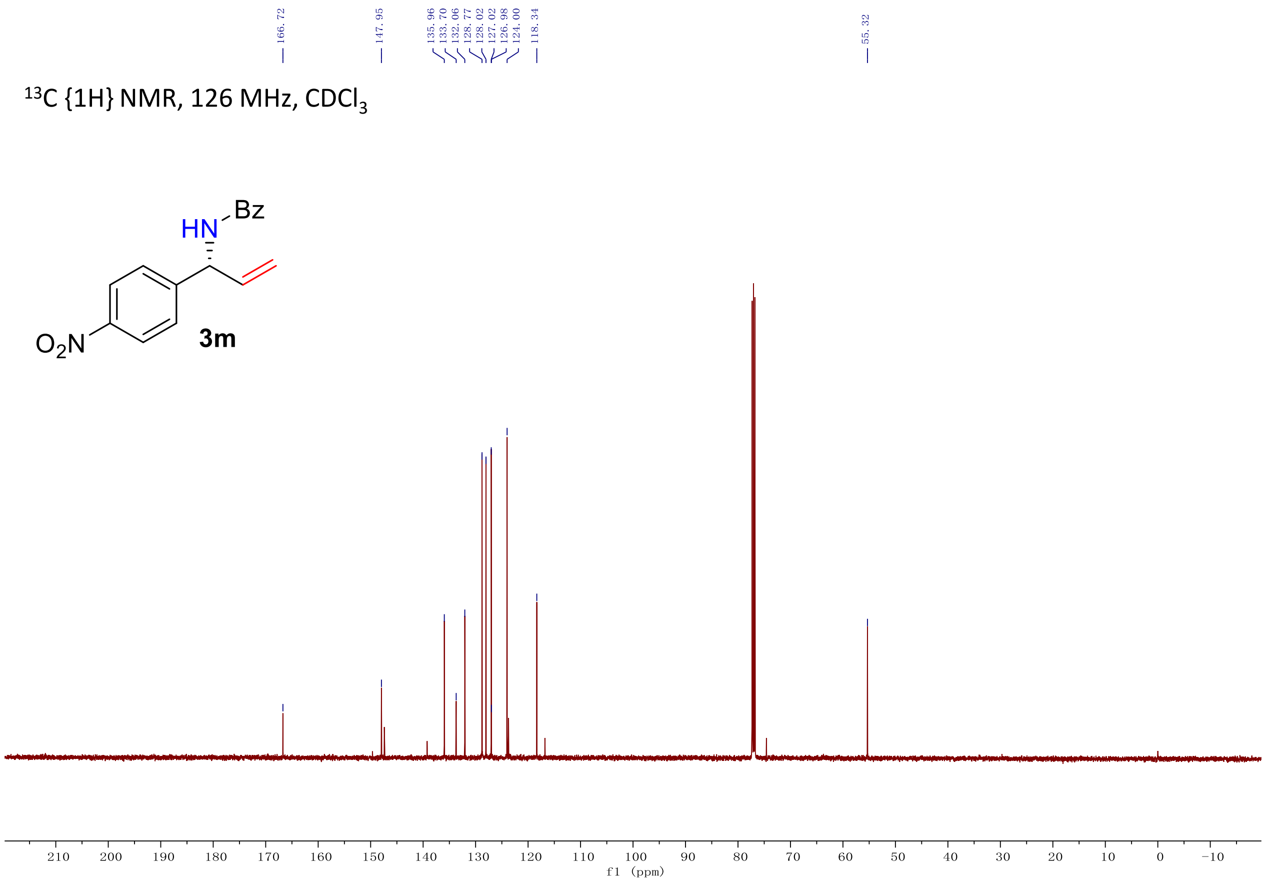

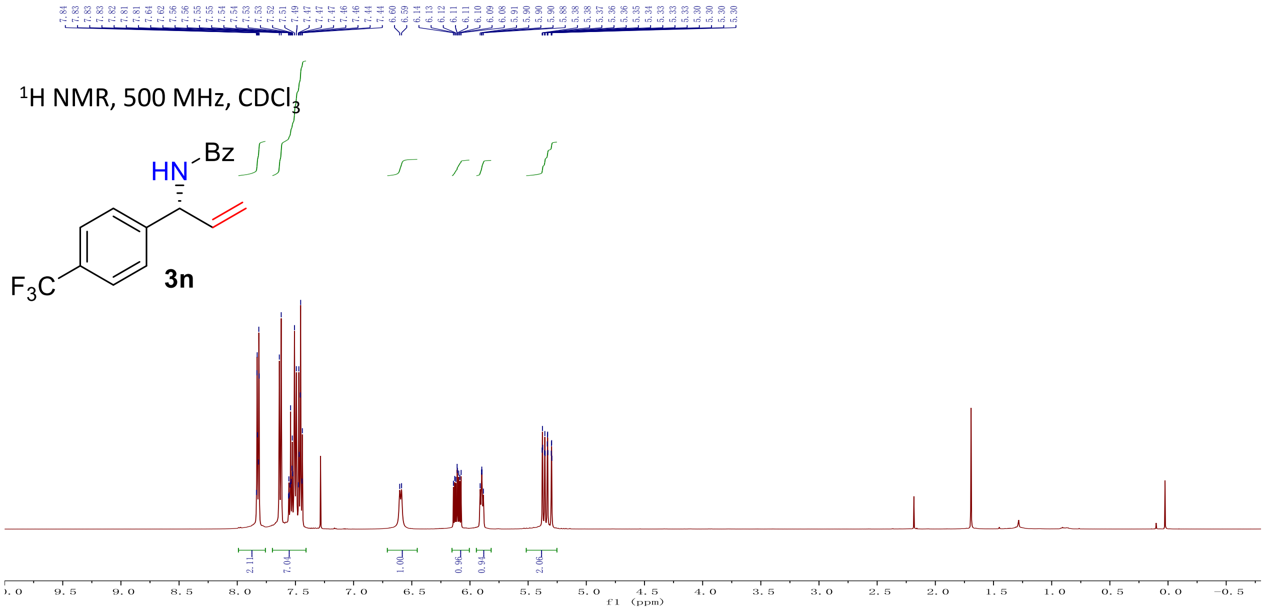
7.84 7.83 7.83 7.83 7.82 7.81 7.81 7.64 7.62 7.56 7.56 7.55 7.55 7.54 7.54 7.53 7.53 7.52 7.51 7.49 7.47 7.47 7.47 7.46 7.46 7.44 7.44 6.60 6.59 6.14 6.13 6.12 6.11 6.11 6.10 6.09 6.08 5.91 5.90 5.90 5.90 5.88 5.38 5.38 5.38 5.37 5.36 5.36 5.35 5.34 5.33 5.33 5.33 5.30 5.30 5.30 5.30
1H NMR, 500 MHz, CDCl3
HN
Bz
F3C
3n
2.11
7.04
1.00
0.96
0.94
2.06
f1 (ppm)

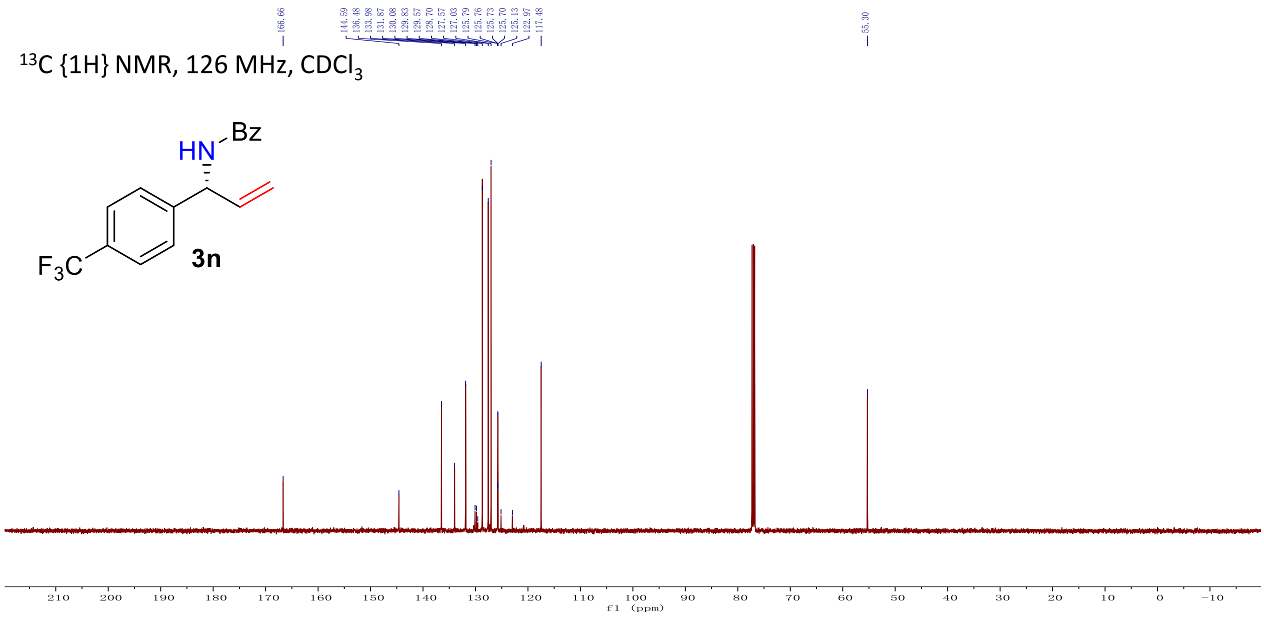
166.66
144.59 136.48 133.98 131.87 130.08 129.83 129.57 128.70 127.57 127.03 125.79 125.76 125.73 125.70 125.13 122.97 117.48
55.30
13C {1H} NMR, 126 MHz, CDCl3
HN
Bz
F3C
3n
f1 (ppm)

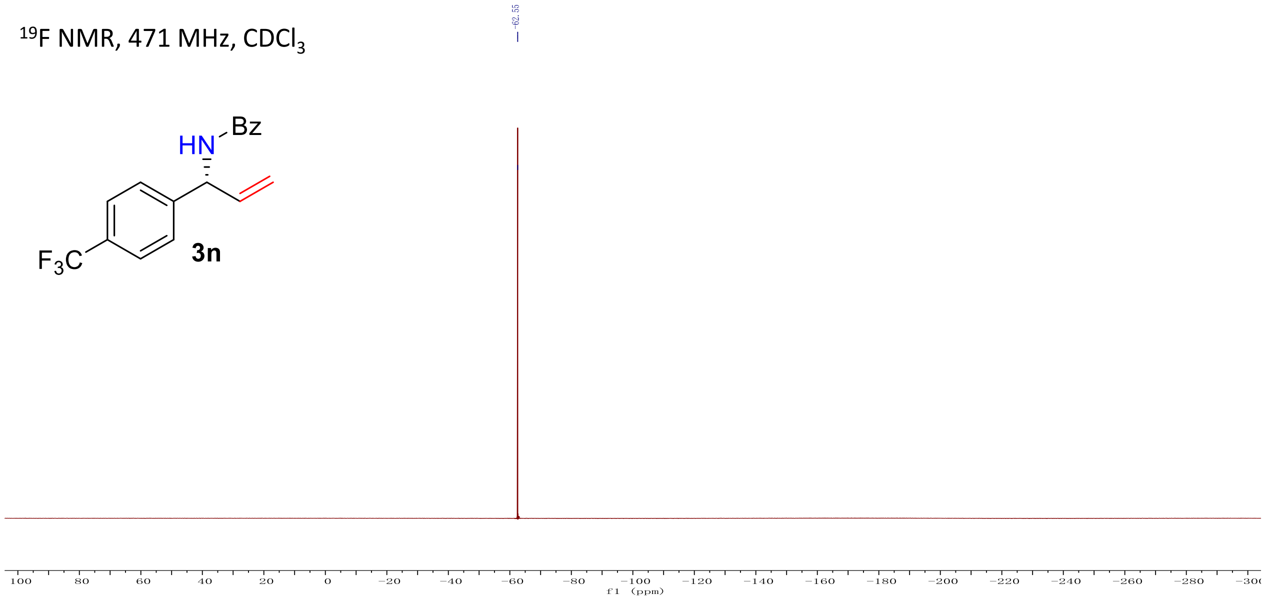
-62.55
19F NMR, 471 MHz, CDCl3
HN
Bz
F3C
3n
f1 (ppm)

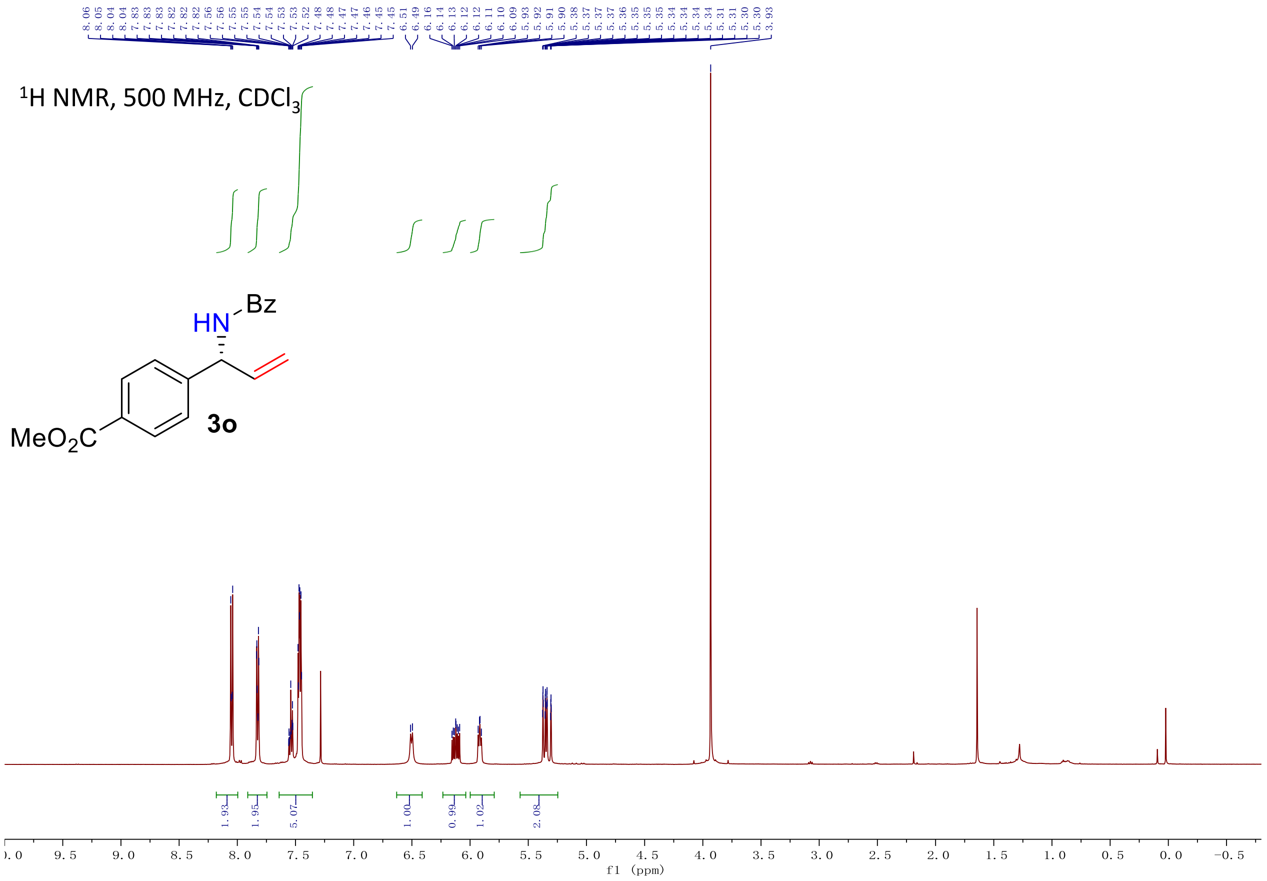
1H NMR, 500 MHz, CDCl3
HN
Bz
MeO2C
3o

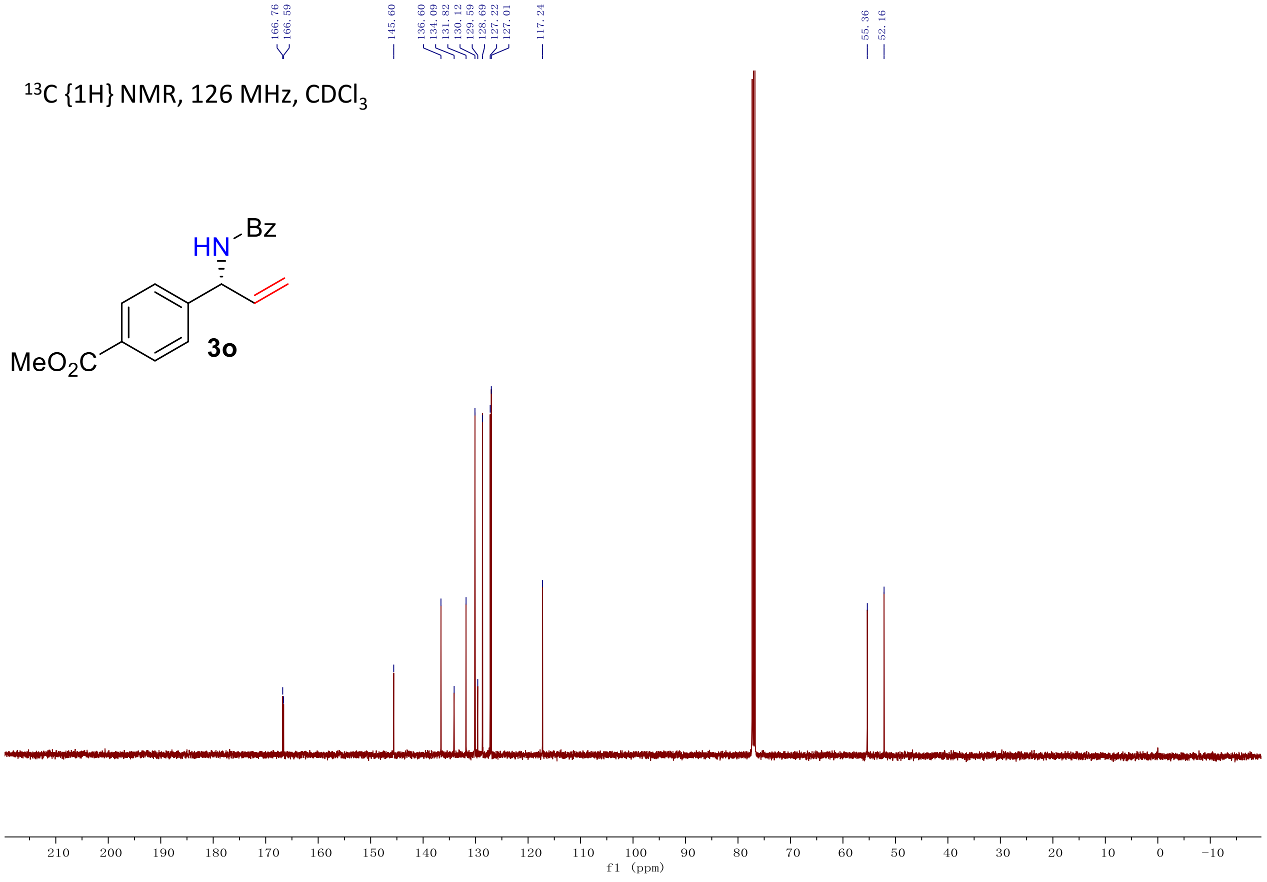
13C {1H} NMR, 126 MHz, CDCl3
HN
Bz
MeO2C
3o

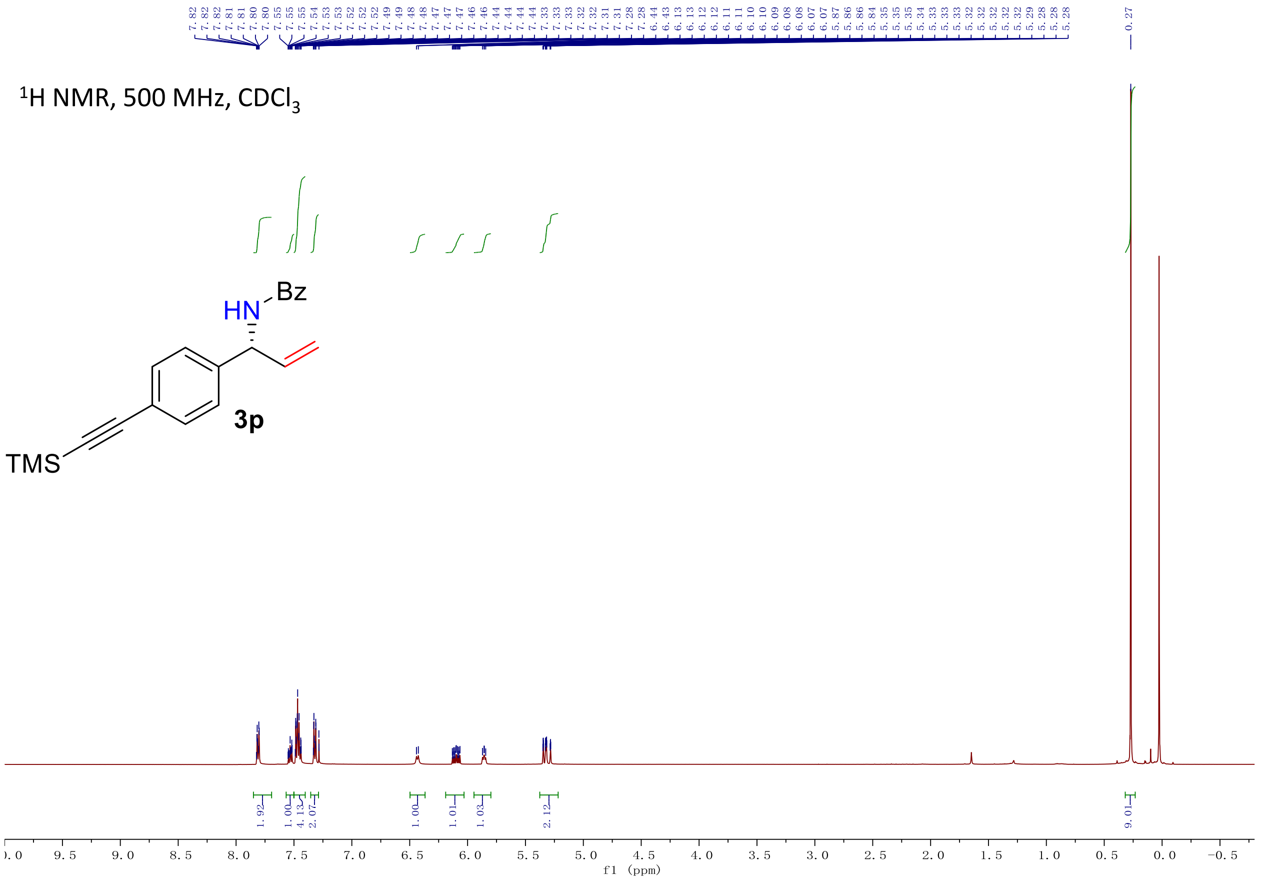

1H NMR, 500 MHz, CDCl3
HN
Bz
TMS
3p
1.92
1.00
4.13
2.07
1.00
1.01
1.03
2.12
9.01
0.27
f1 (ppm)


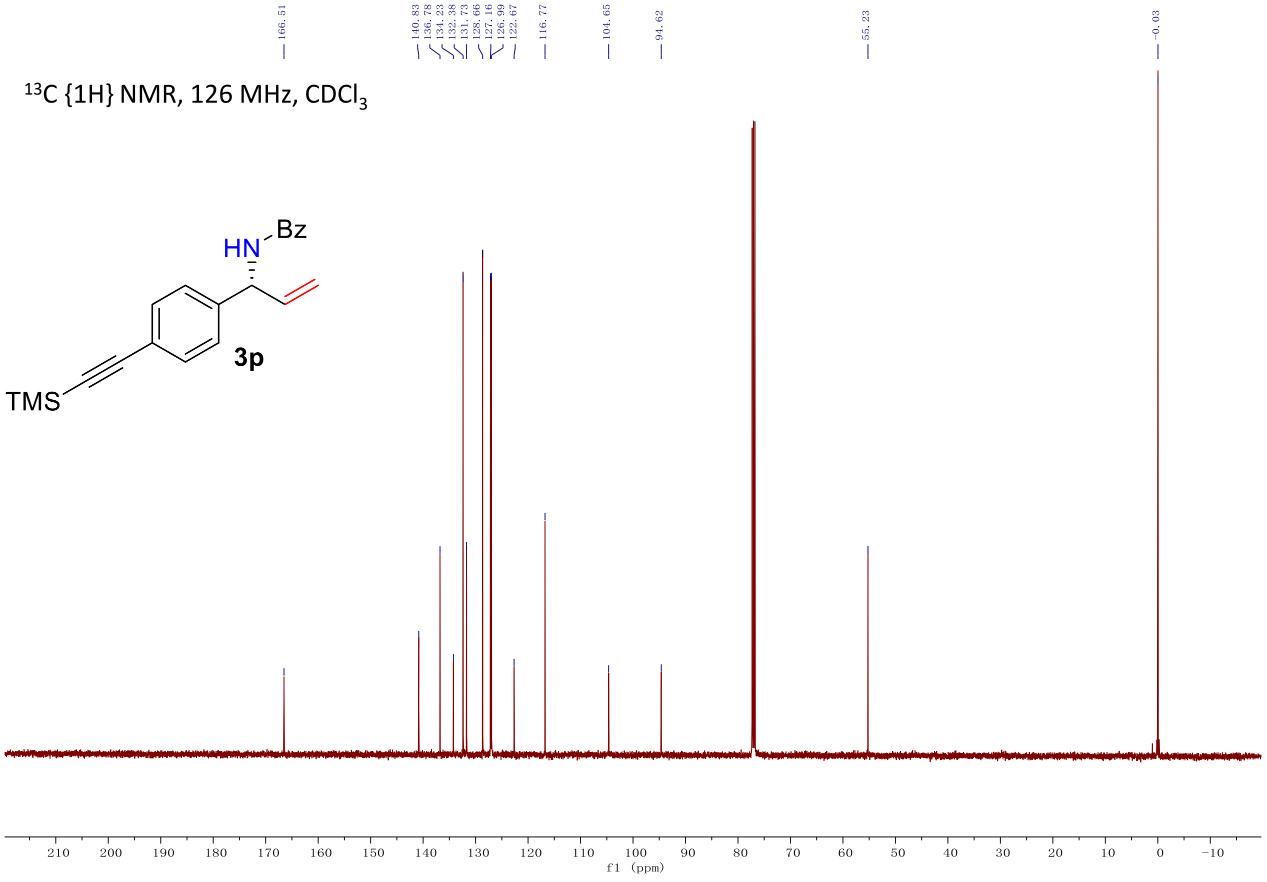

13C {1H} NMR, 126 MHz, CDCl3
HN
Bz
TMS
3p
166.51
140.83
136.78
134.23
132.38
131.73
128.66
127.16
126.99
122.67
116.77
104.65
94.62
55.23
-0.03
f1 (ppm)

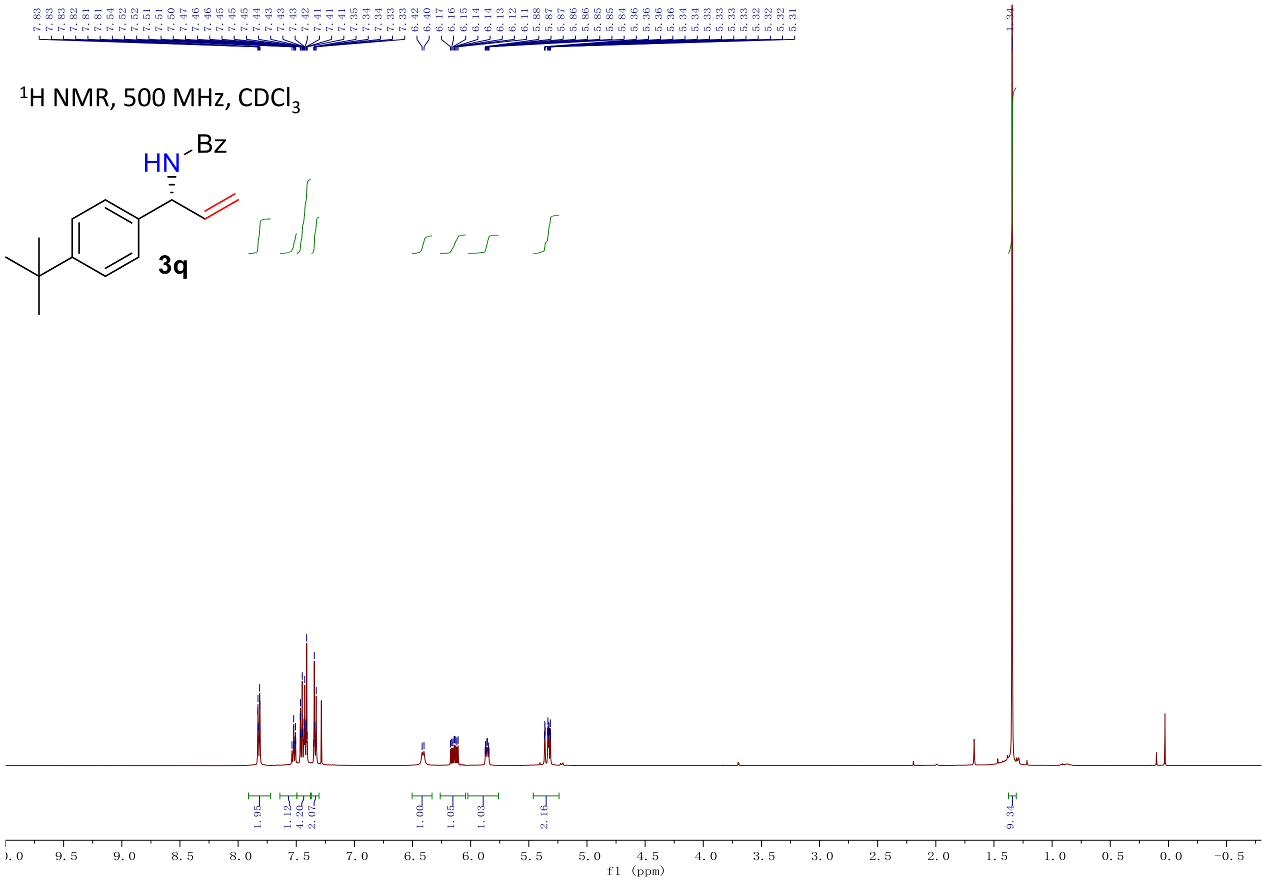

$^1H$ NMR, 500 MHz, $CDCl_3$

**3q**

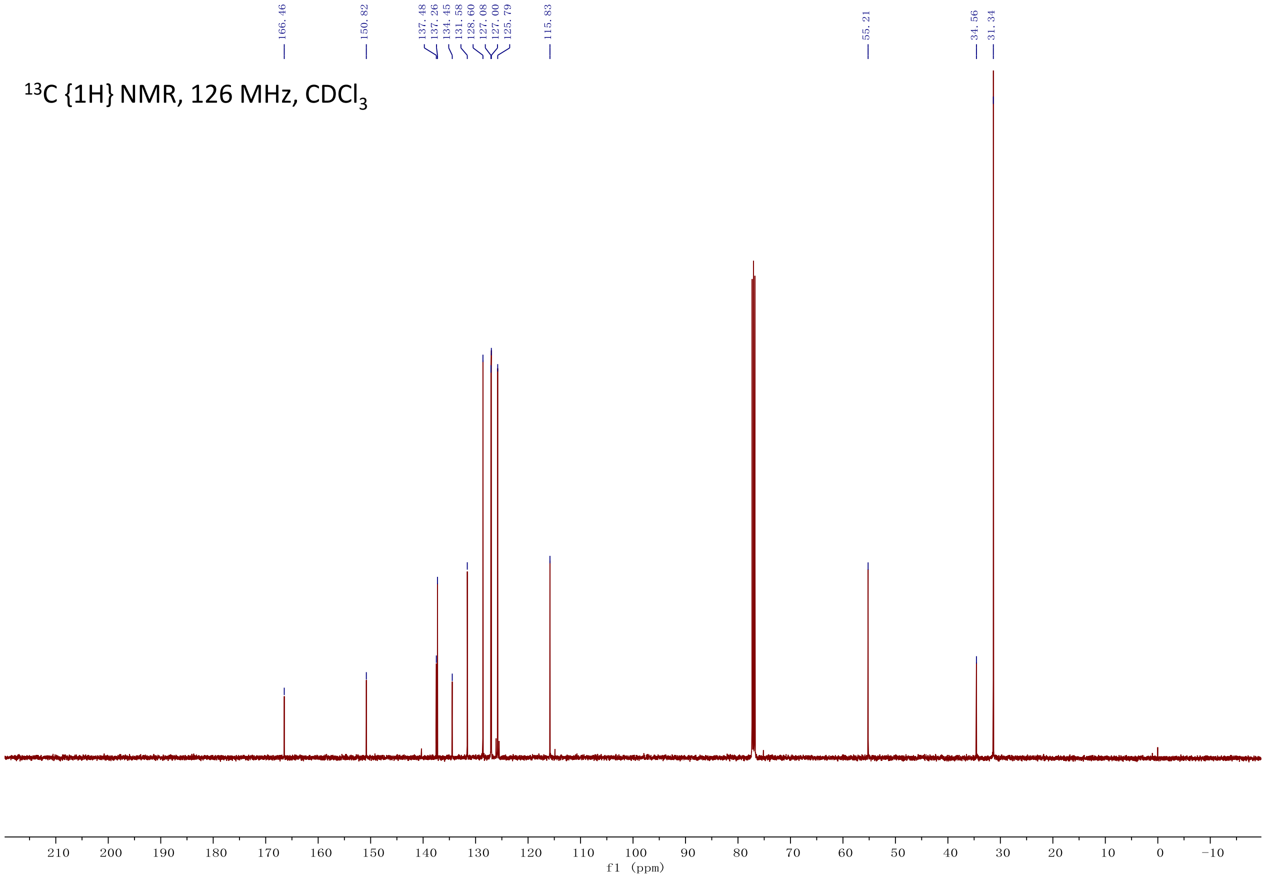

$^{13}C$ {1H} NMR, 126 MHz, $CDCl_3$

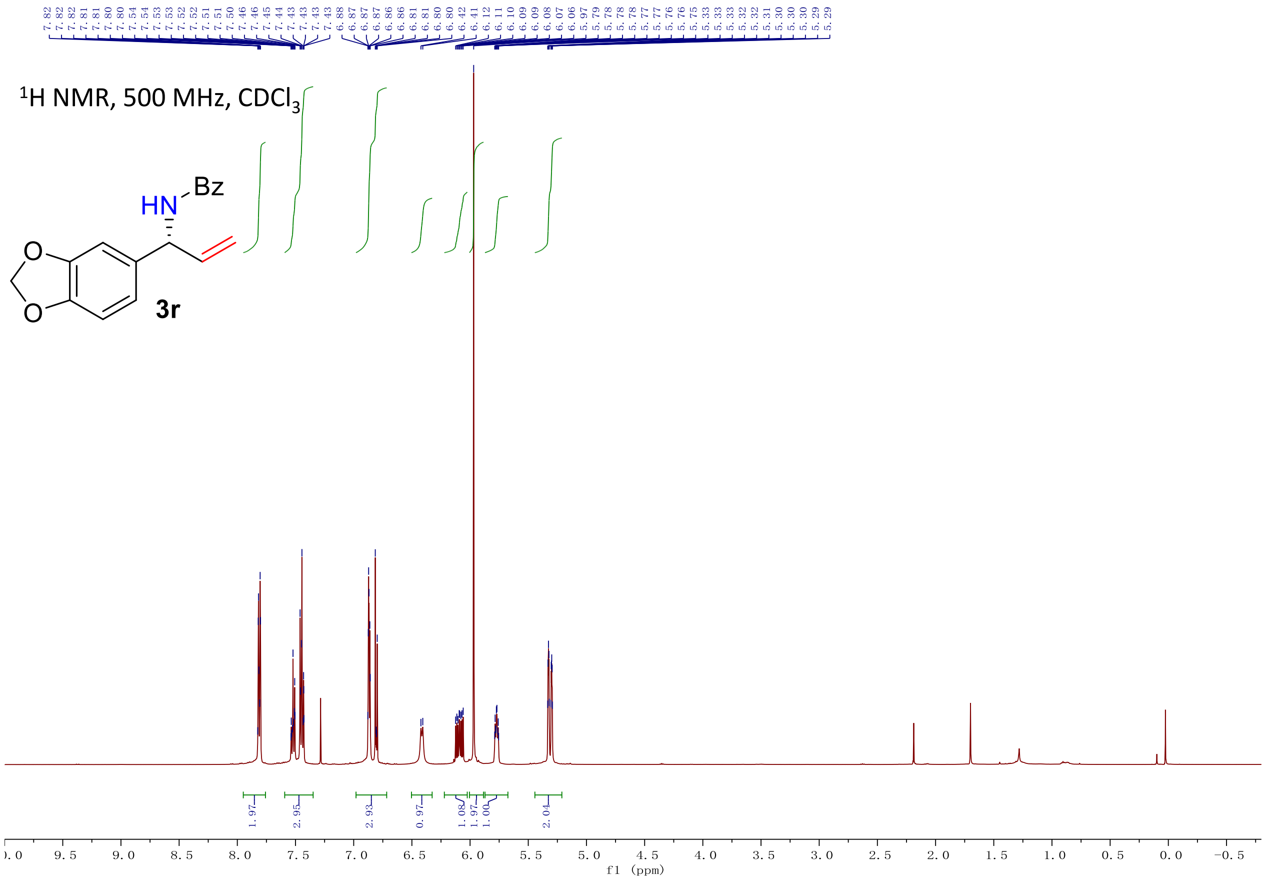
$^1$H NMR, 500 MHz, $CDCl_3$
HN
Bz
O
O
3r
f1 (ppm)

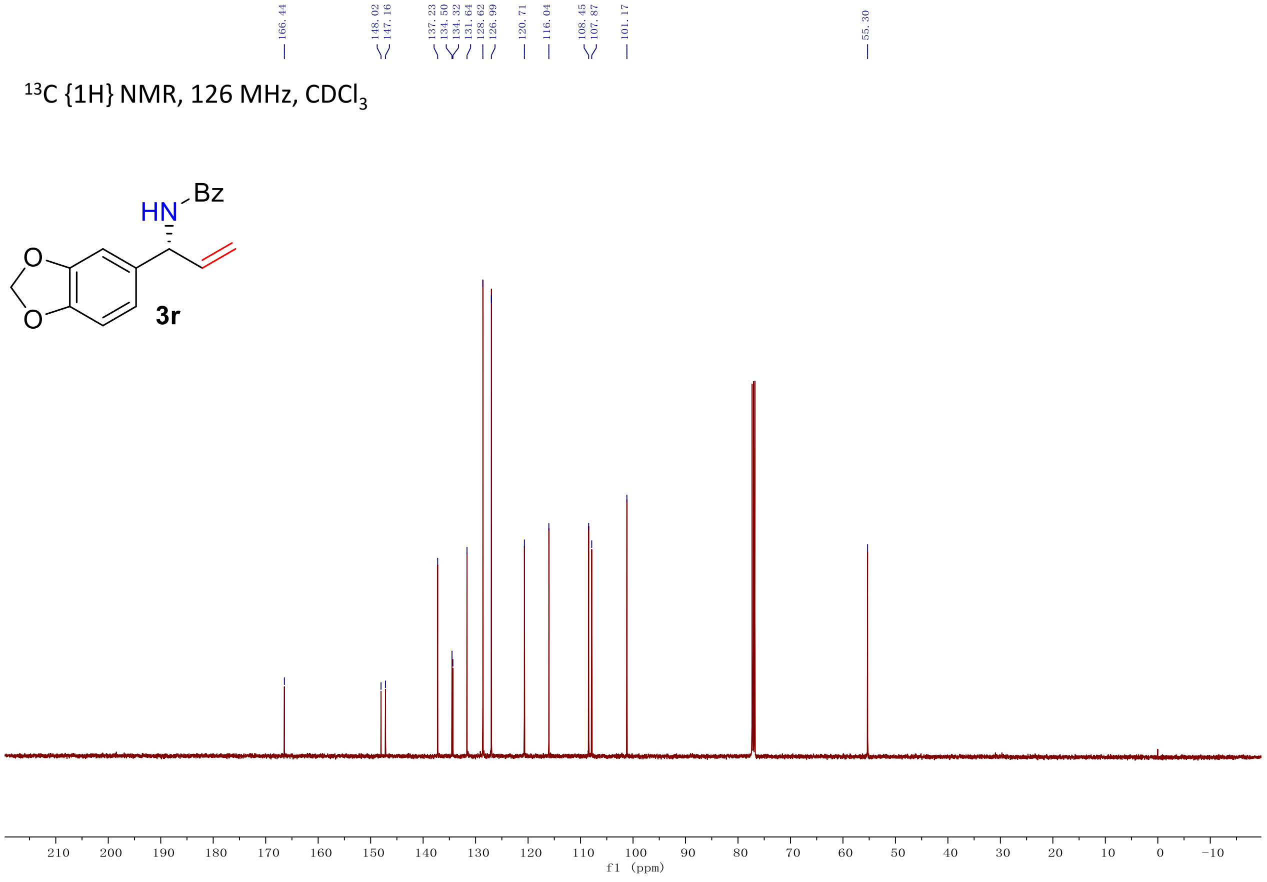
$^{13}$C {1H} NMR, 126 MHz, $CDCl_3$
HN
Bz
O
O
3r
166.44
148.02
147.16
137.23
134.50
134.32
131.64
128.62
126.99
120.71
116.04
108.45
107.87
101.17
55.30
f1 (ppm)

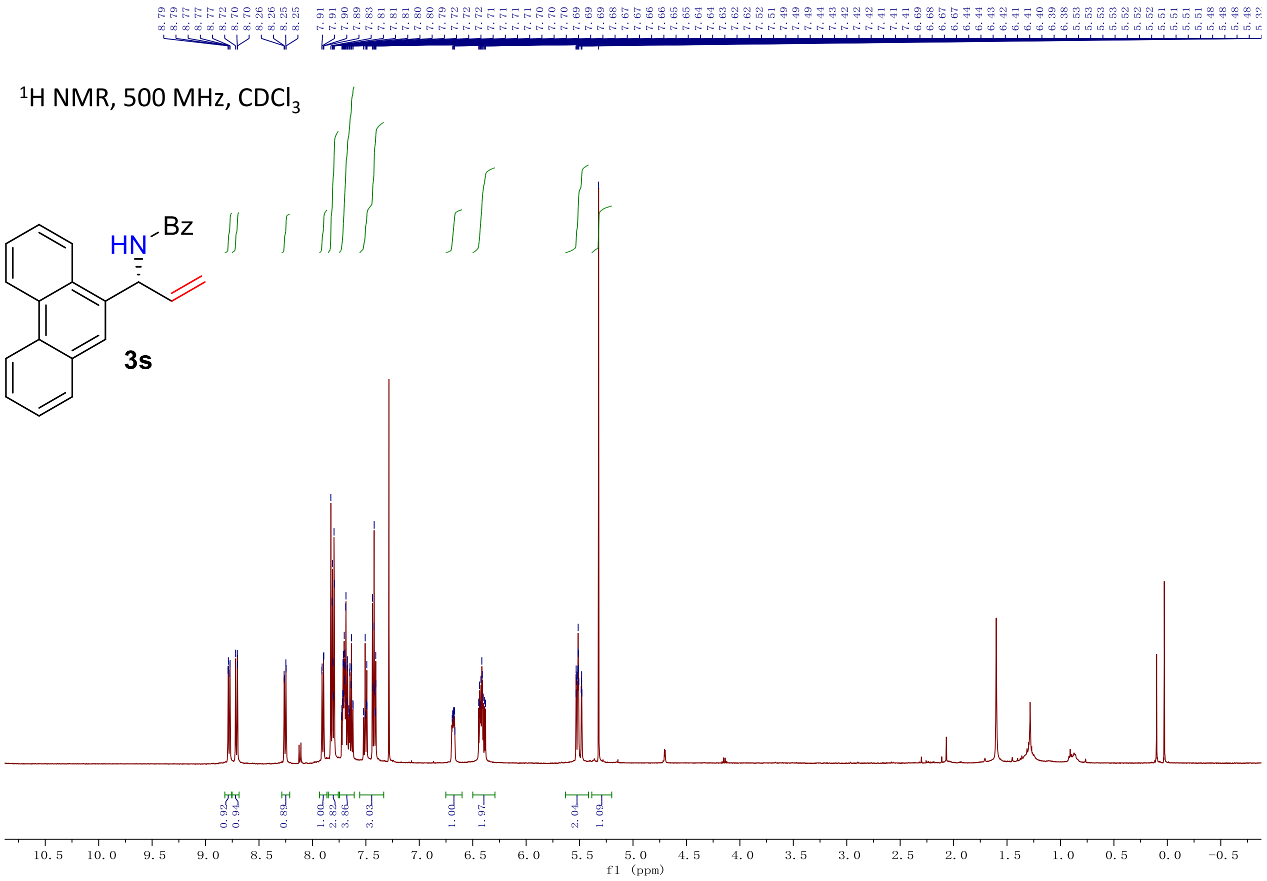

1H NMR, 500 MHz, CDCl3
HN
Bz
3s
0.92
0.91
0.89
1.00
2.82
3.86
3.03
1.00
1.97
2.04
1.09
f1 (ppm)


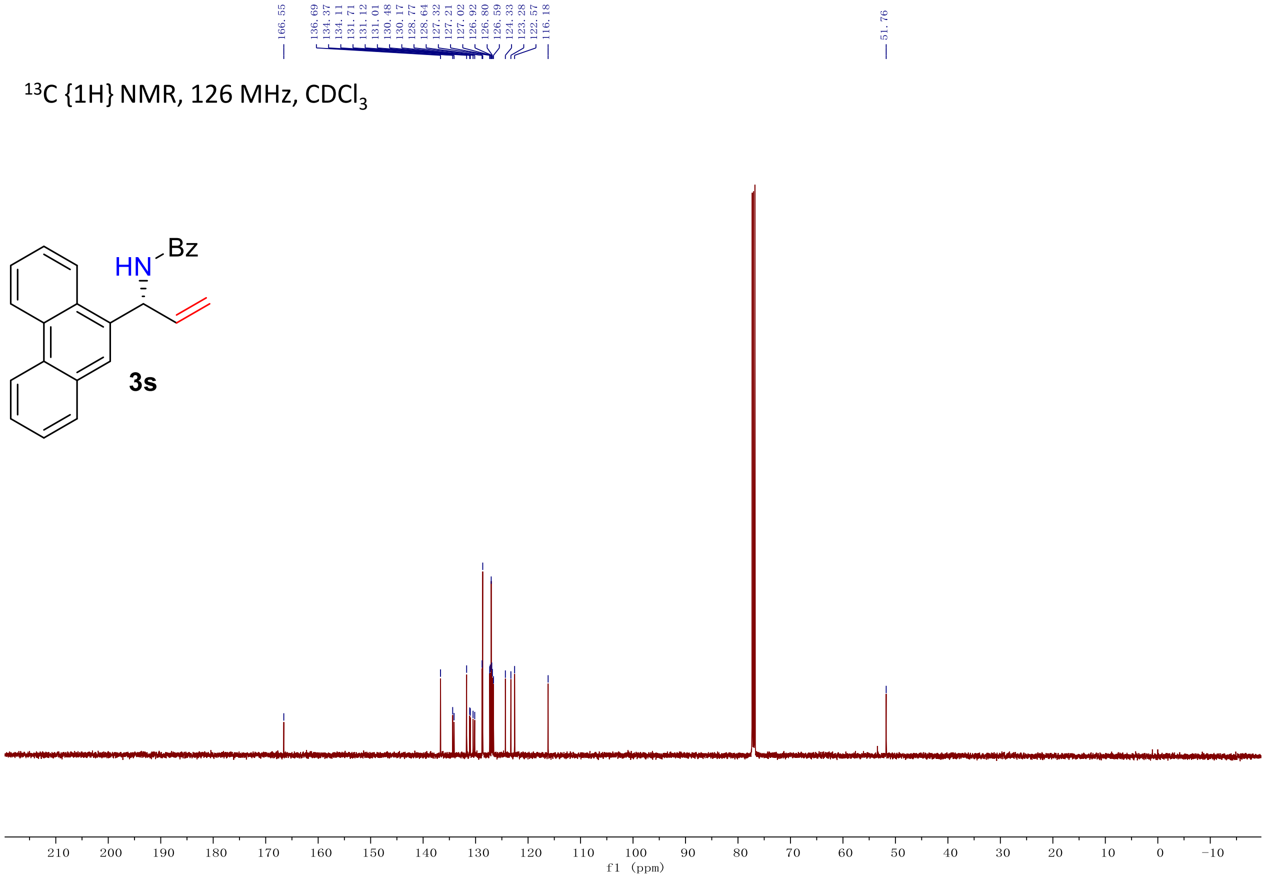

166.55
136.69
134.37
134.11
131.71
131.12
131.01
130.48
130.17
128.77
128.64
127.32
127.21
127.02
126.92
126.80
126.59
124.33
123.28
122.57
116.18
51.76
13C {1H} NMR, 126 MHz, CDCl3
HN
Bz
3s
f1 (ppm)

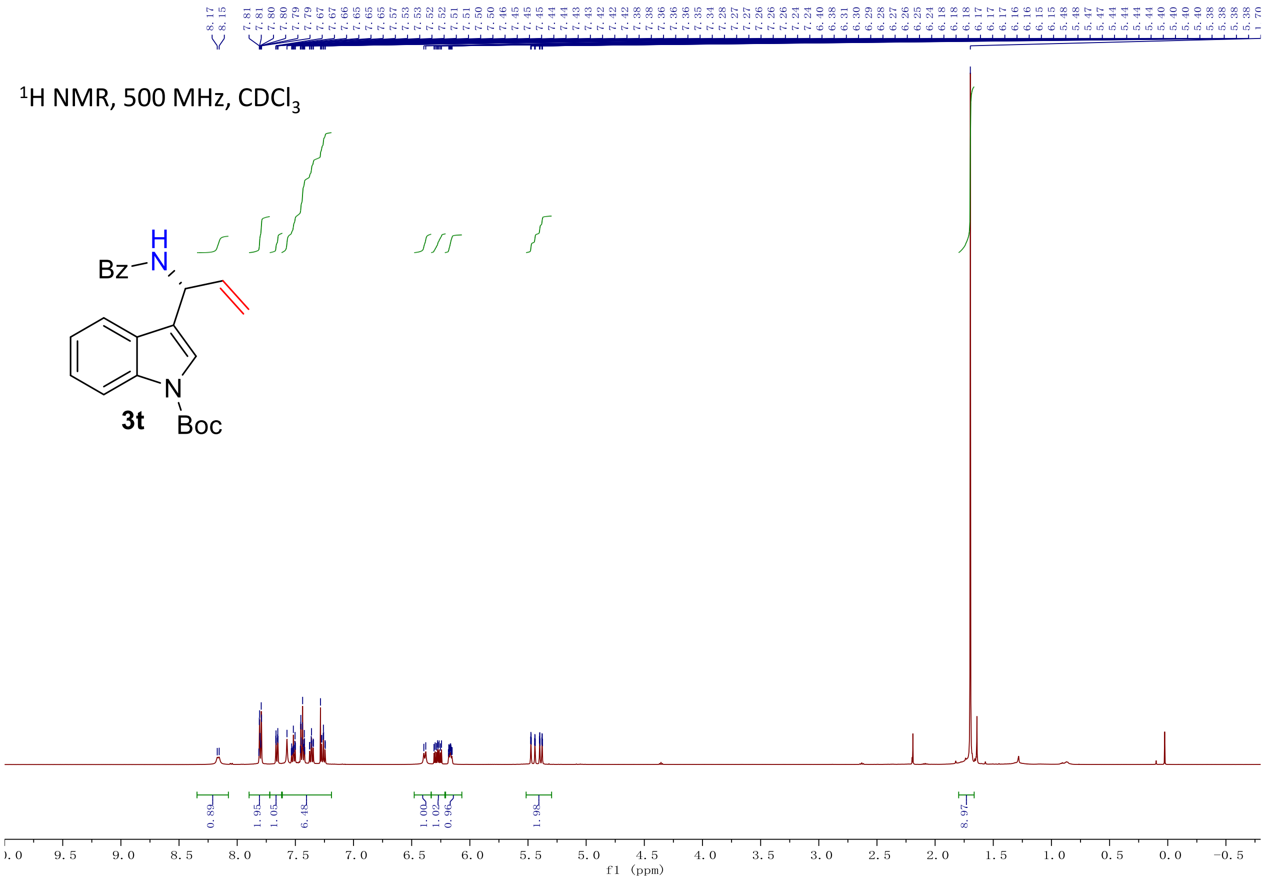

1H NMR, 500 MHz, CDCl3
Bz
H
N
3t
N
Boc
f1 (ppm)


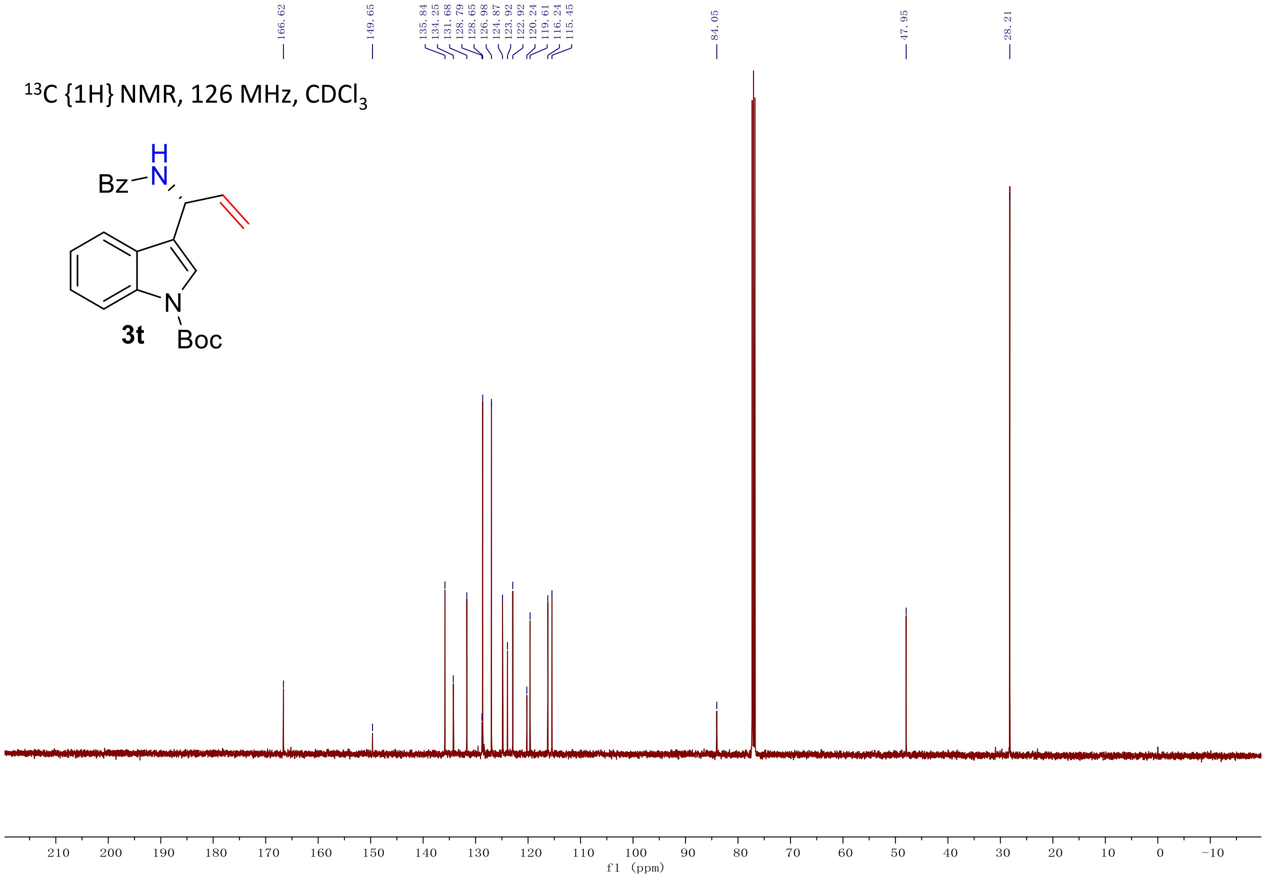

13C {1H} NMR, 126 MHz, CDCl3
166.62
149.65
135.84
134.25
131.68
128.79
128.65
126.98
124.87
123.92
122.92
120.24
119.61
116.24
115.45
84.05
47.95
28.21
Bz
H
N
3t
N
Boc
f1 (ppm)

$^1$H NMR, 500 MHz, $CDCl_3$

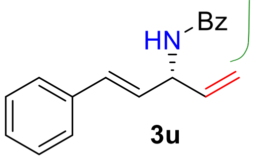

3u

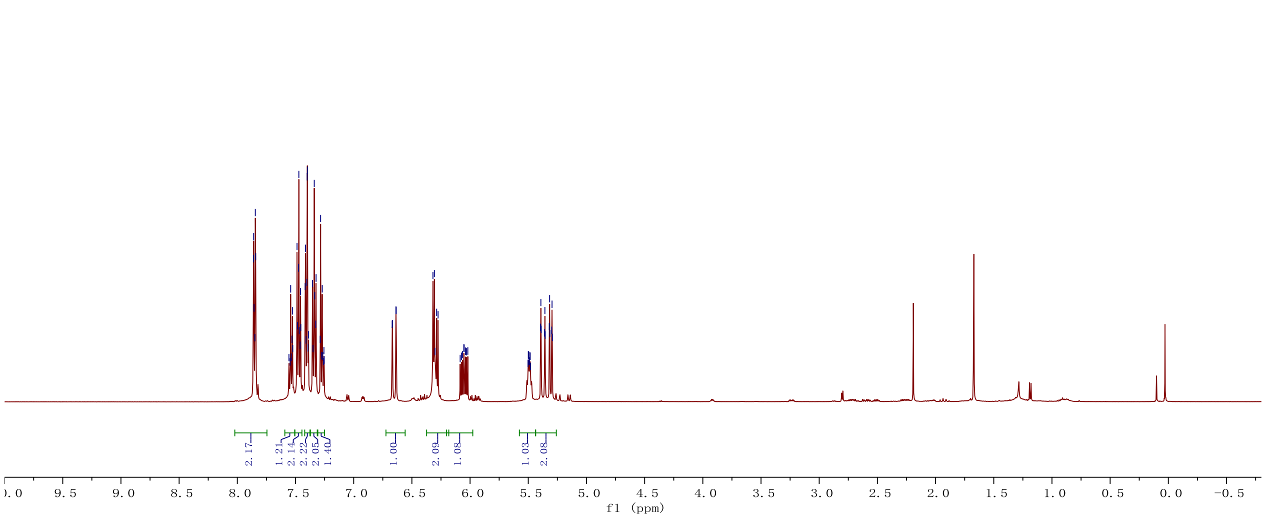

$^{13}$C {1H} NMR, 126 MHz, $CDCl_3$

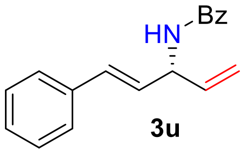

3u

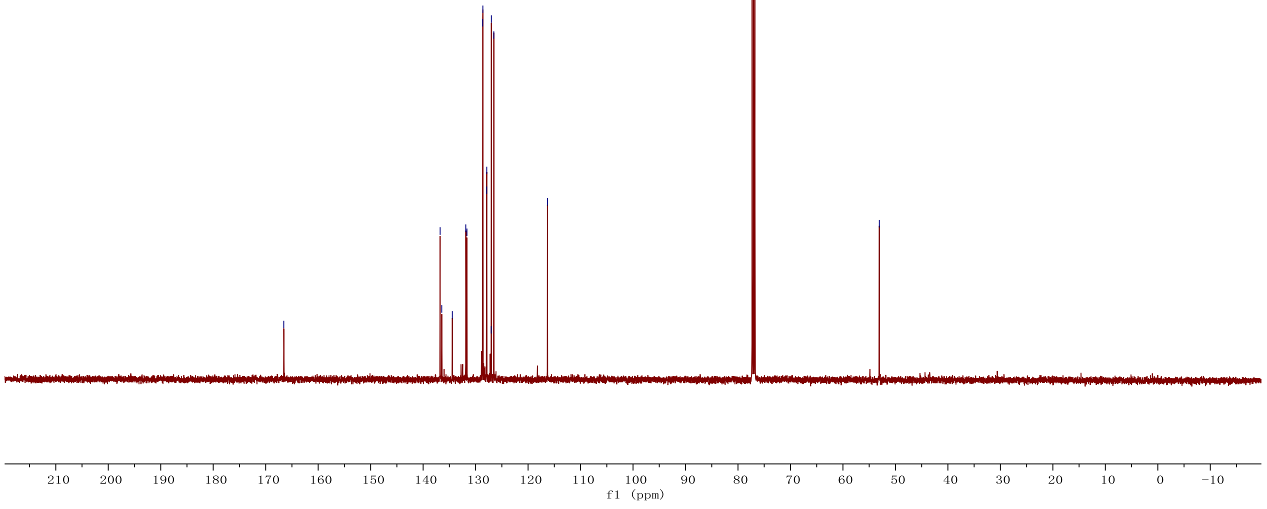

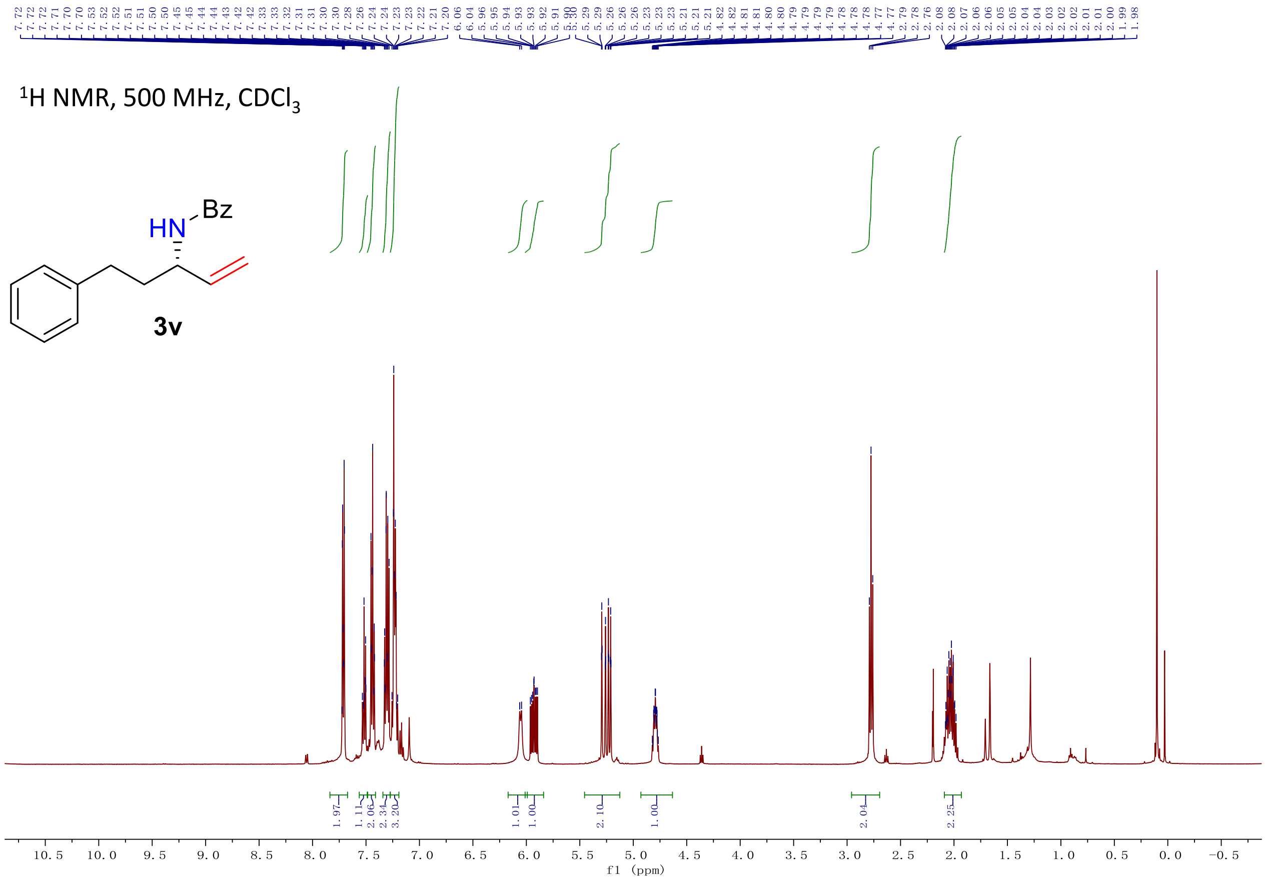
1H NMR, 500 MHz, CDCl3
HN
Bz
3v
f1 (ppm)

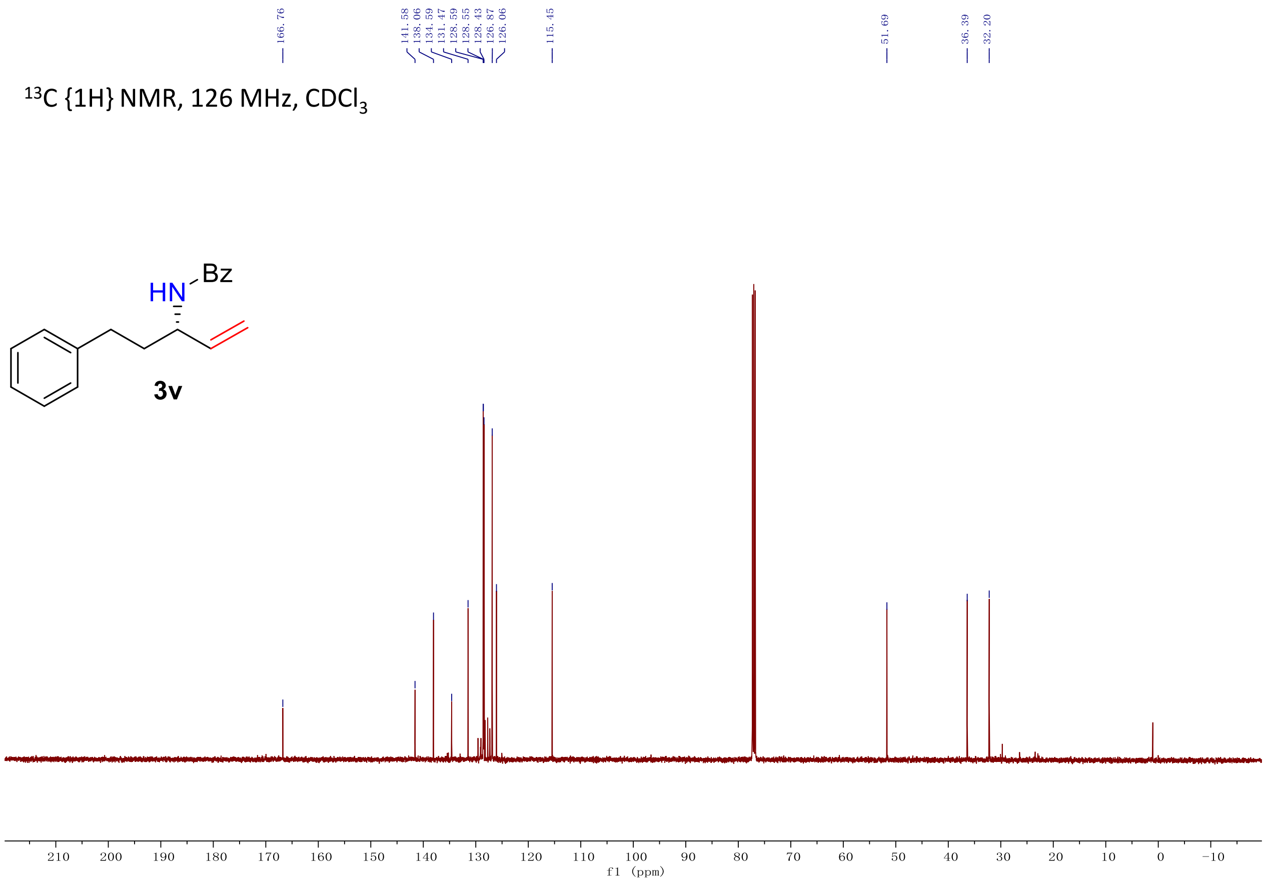
166.76
141.58
138.06
134.59
131.47
128.59
128.55
128.43
126.87
126.06
115.45
51.69
36.39
32.20
13C {1H} NMR, 126 MHz, CDCl3
HN
Bz
3v
f1 (ppm)

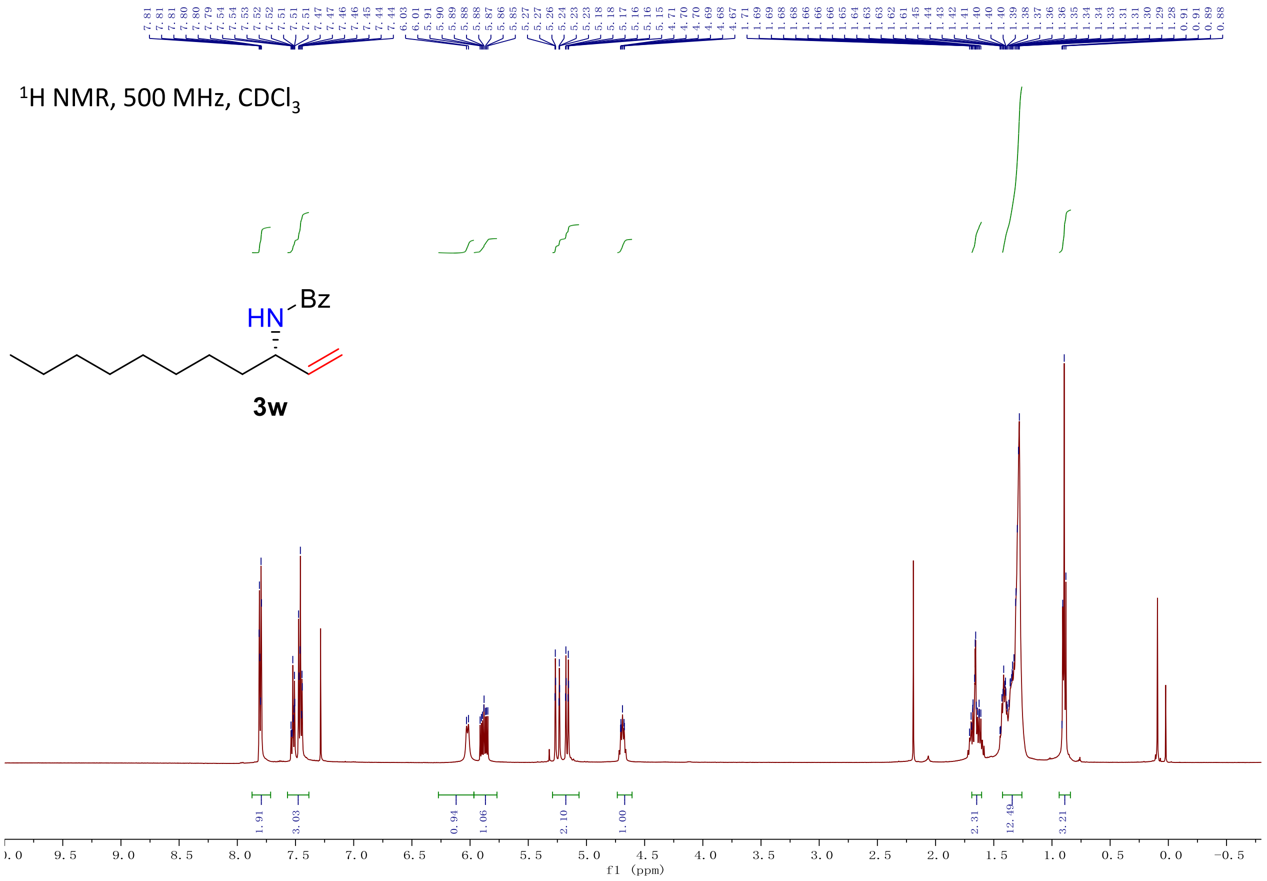

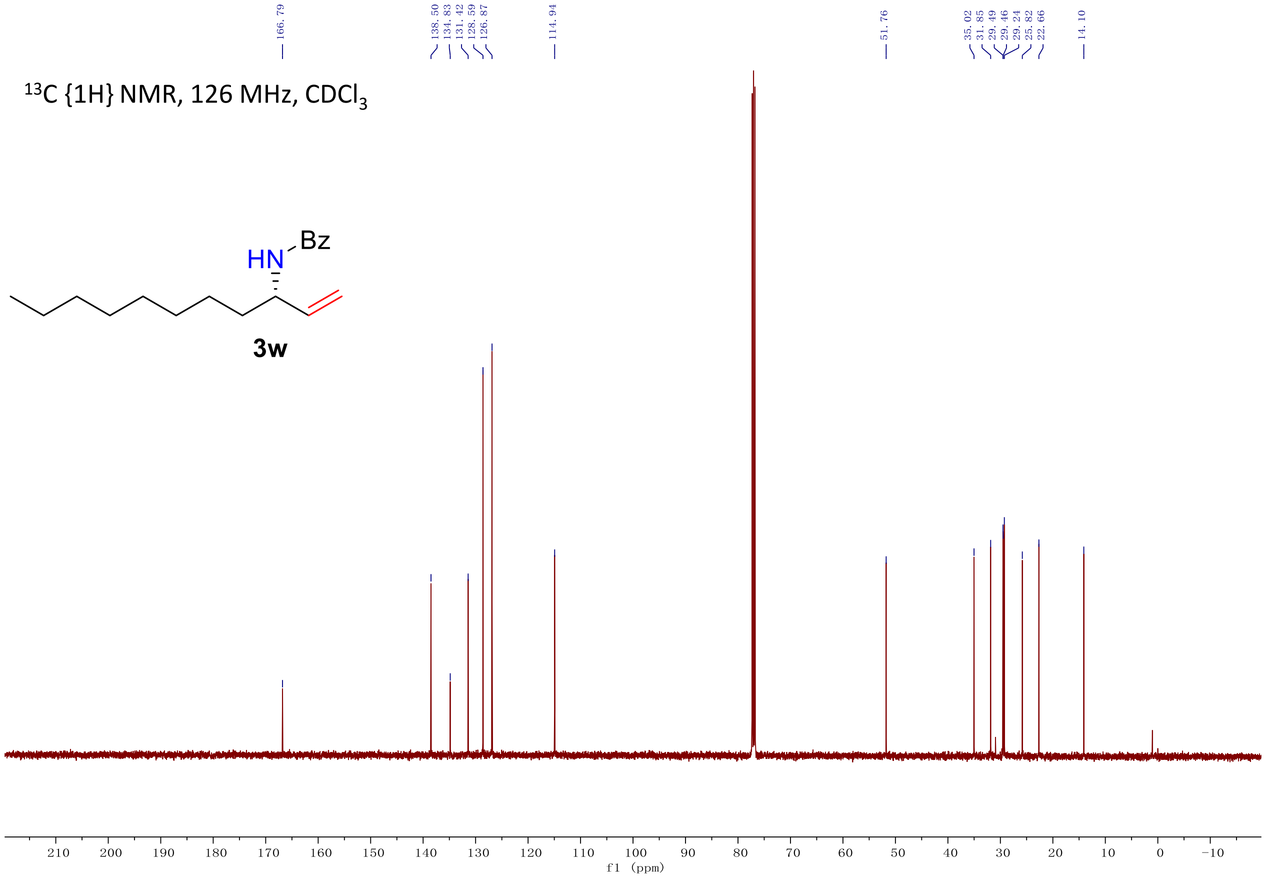

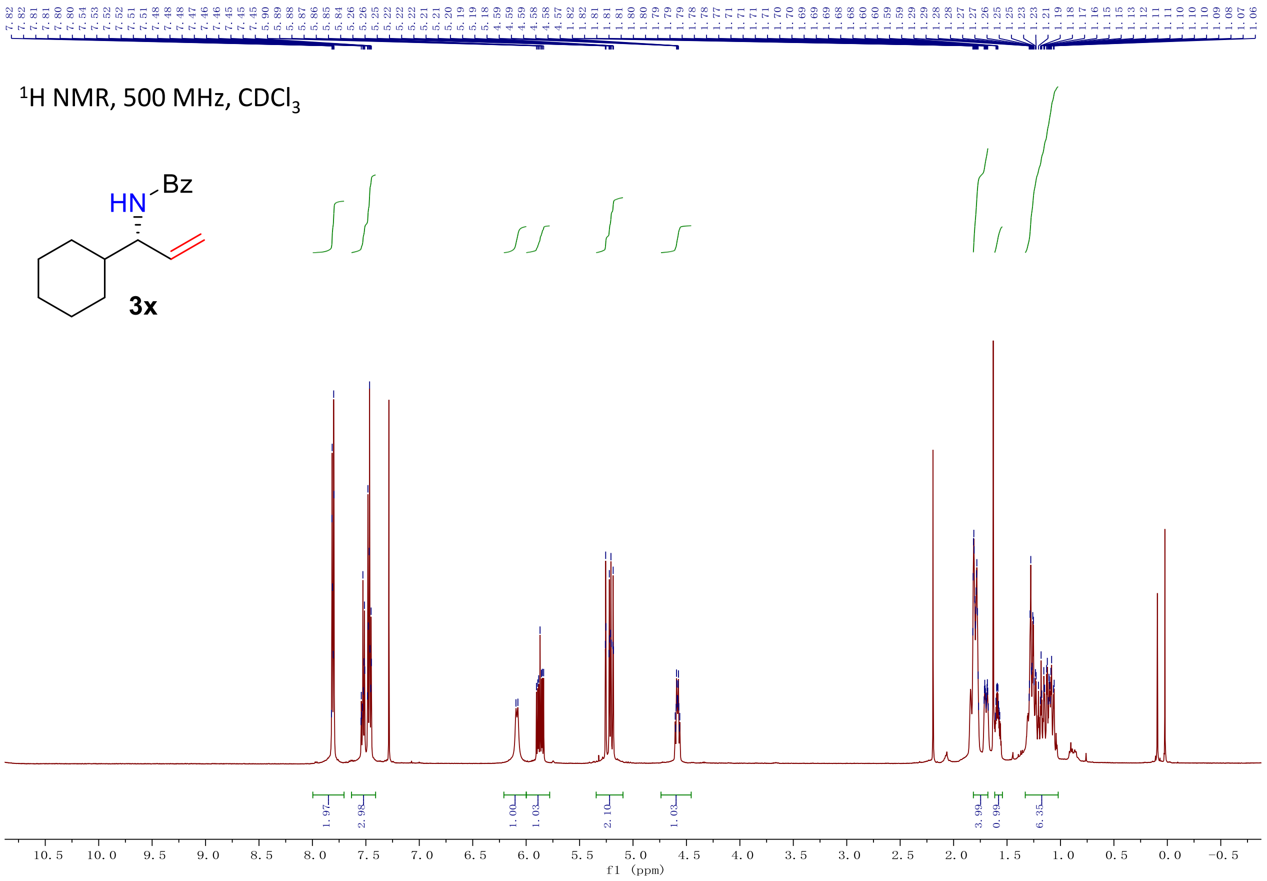

$^{1}$H NMR, 500 MHz, CDCl$_3$
HN
Bz
3x
7.82 7.82 7.81 7.80 7.80 7.54 7.53 7.52 7.52 7.51 7.51 7.48 7.48 7.47 7.46 7.45 7.45 7.45 5.90 5.89 5.88 5.87 5.86 5.85 5.84 5.26 5.26 5.25 5.22 5.22 5.21 5.21 5.20 5.20 5.19 5.19 5.18 4.59 4.59 4.59 4.58 4.57 1.82 1.82 1.81 1.81 1.81 1.80 1.79 1.79 1.79 1.78 1.77 1.71 1.71 1.71 1.70 1.69 1.69 1.69 1.68 1.68 1.60 1.59 1.59 1.29 1.29 1.28 1.28 1.27 1.27 1.26 1.25 1.25 1.23 1.23 1.21 1.19 1.18 1.17 1.16 1.15 1.13 1.12 1.11 1.11 1.10 1.10 1.09 1.08 1.07 1.06
1.97 2.98 1.00 1.03 2.10 1.03 3.99 0.99 6.35
10.5 10.0 9.5 9.0 8.5 8.0 7.5 7.0 6.5 6.0 5.5 5.0 4.5 4.0 3.5 3.0 2.5 2.0 1.5 1.0 0.5 0.0 -0.5
f1 (ppm)


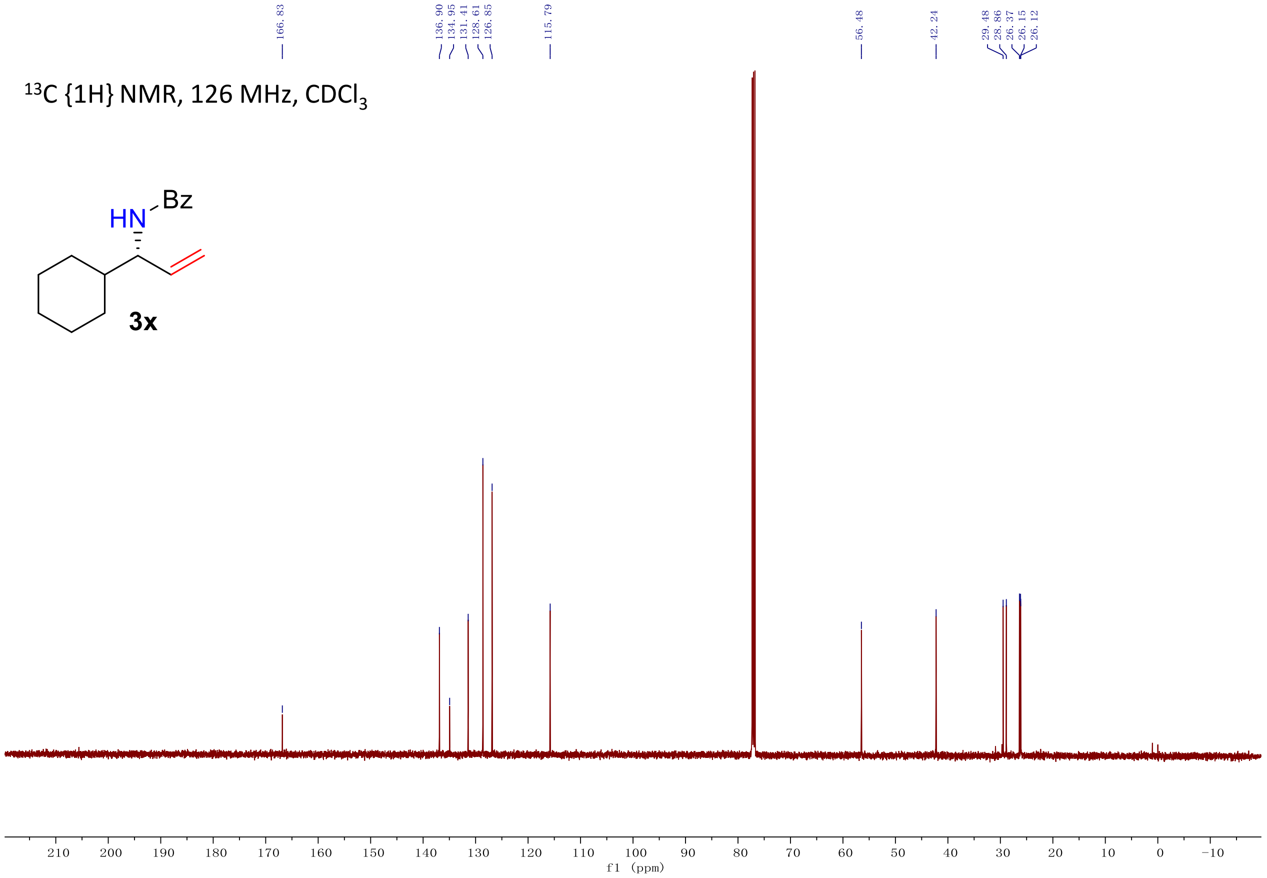

$^{13}$C {1H} NMR, 126 MHz, CDCl$_3$
HN
Bz
3x
166.83
136.90 134.95 131.41 128.61 126.85
115.79
56.48
42.24
29.48 28.86 26.37 26.15 26.12
210 200 190 180 170 160 150 140 130 120 110 100 90 80 70 60 50 40 30 20 10 0 -10
f1 (ppm)

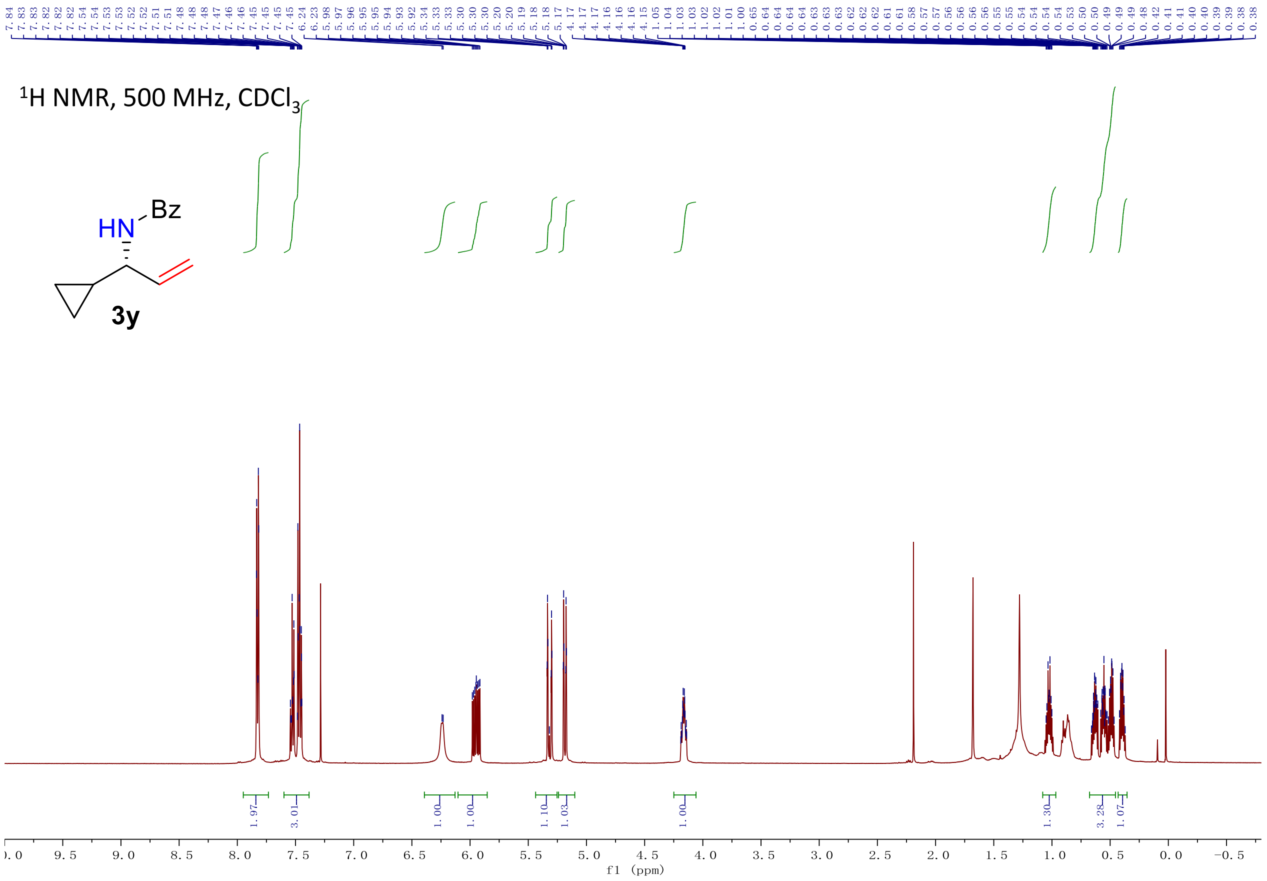

1H NMR, 500 MHz, CDCl3
HN
Bz
3y
f1 (ppm)


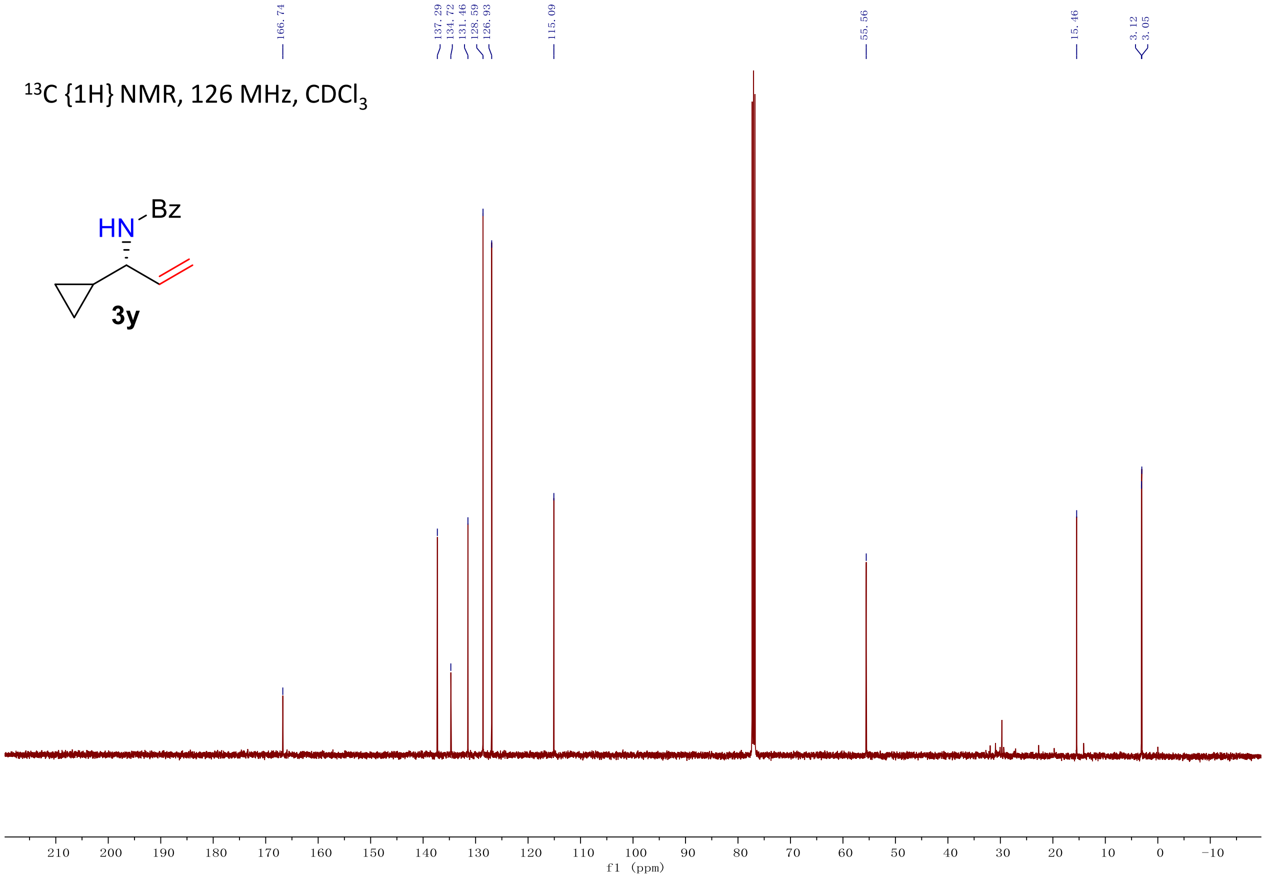

13C {1H} NMR, 126 MHz, CDCl3
HN
Bz
3y
f1 (ppm)

$^{1}H$ NMR, 500 MHz, $CDCl_3$

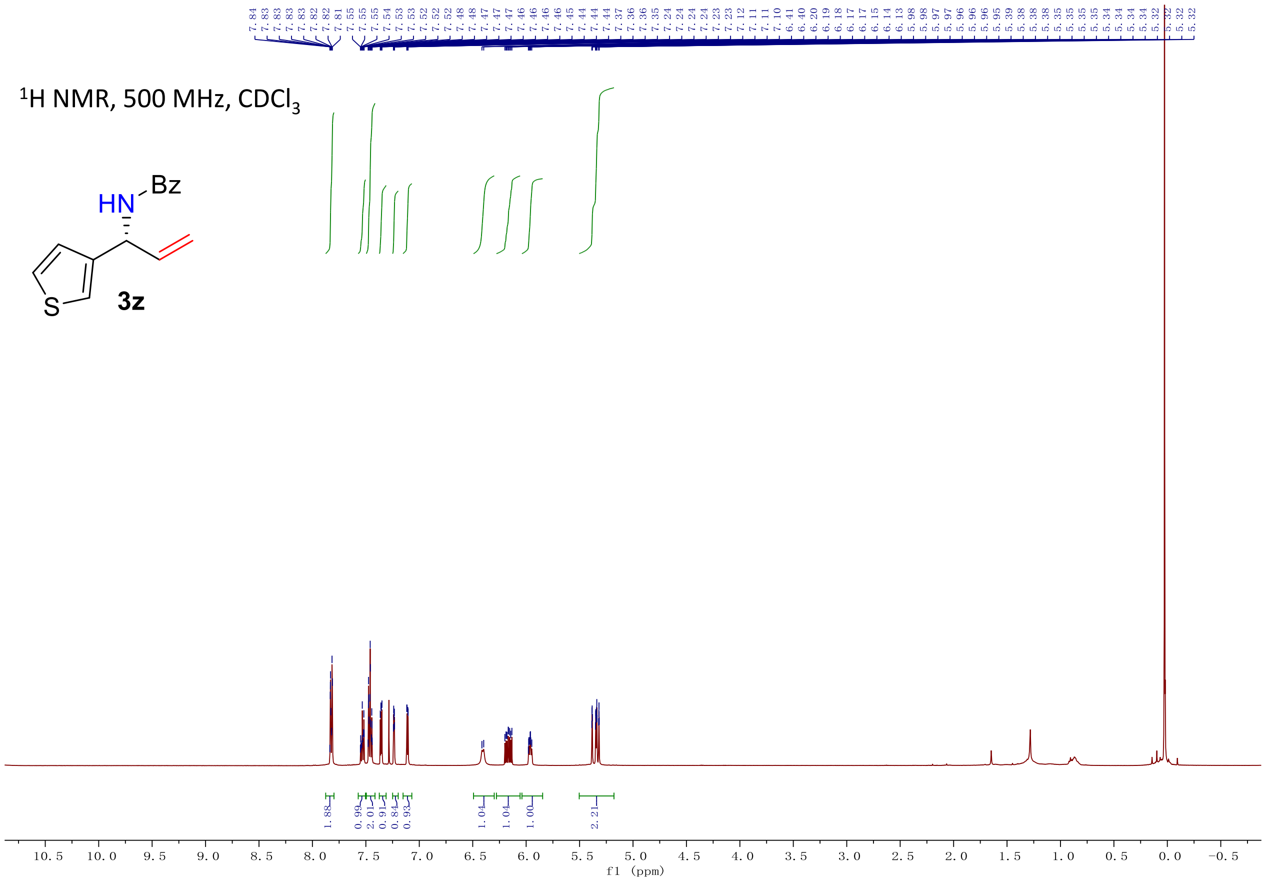


$^{13}C$ {1H} NMR, 126 MHz, $CDCl_3$

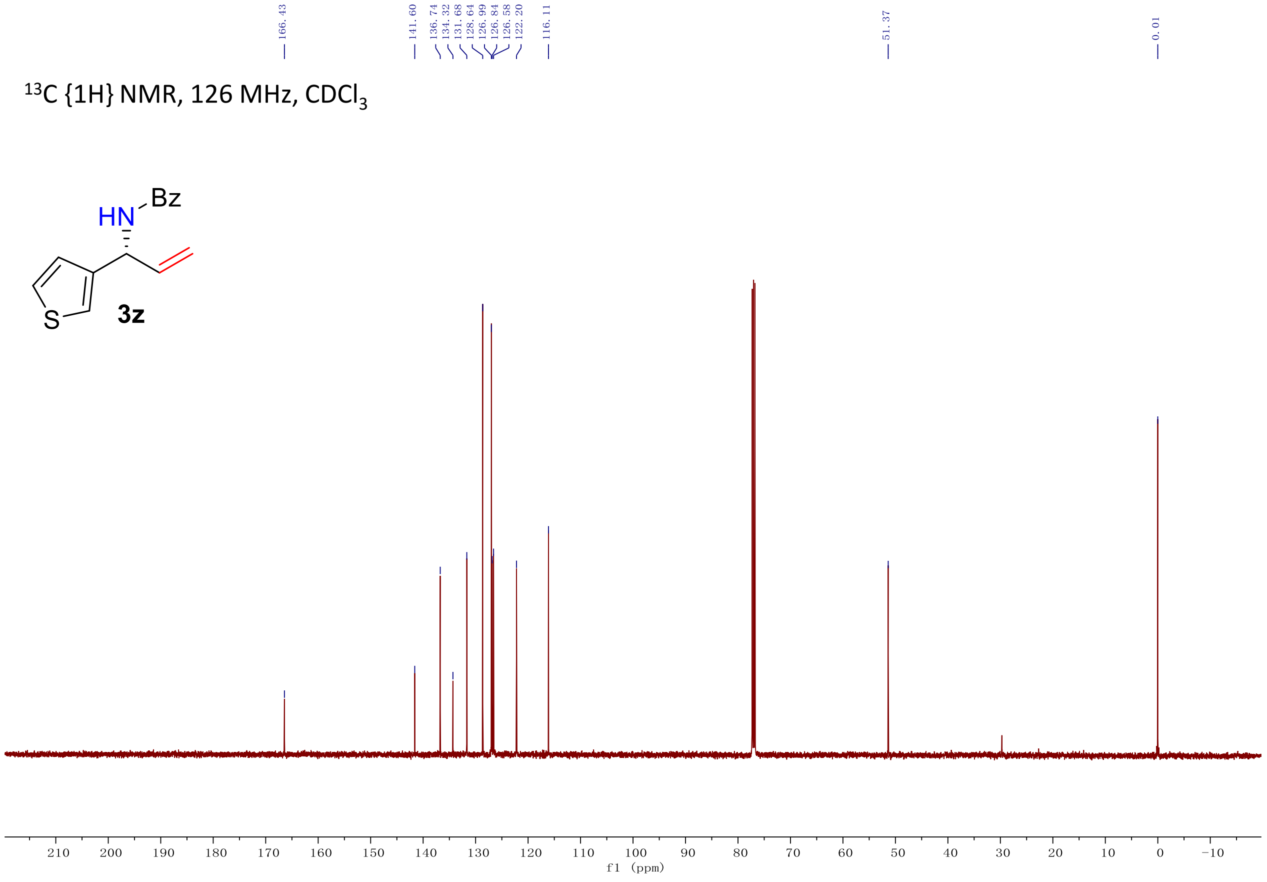

$^{1}$H NMR, 500 MHz, $CDCl_3$

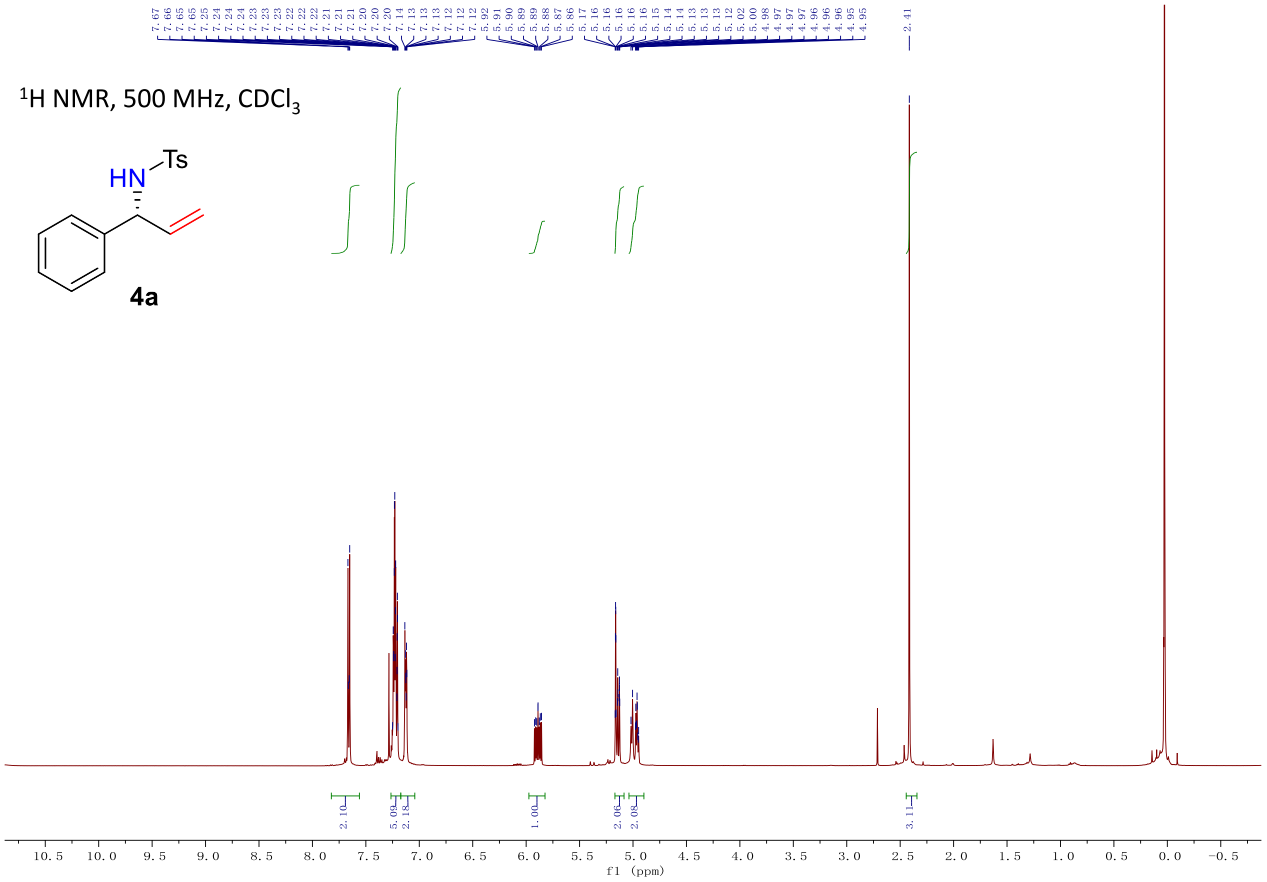

**4a**

$^{13}$C {1H} NMR, 126 MHz, $CDCl_3$

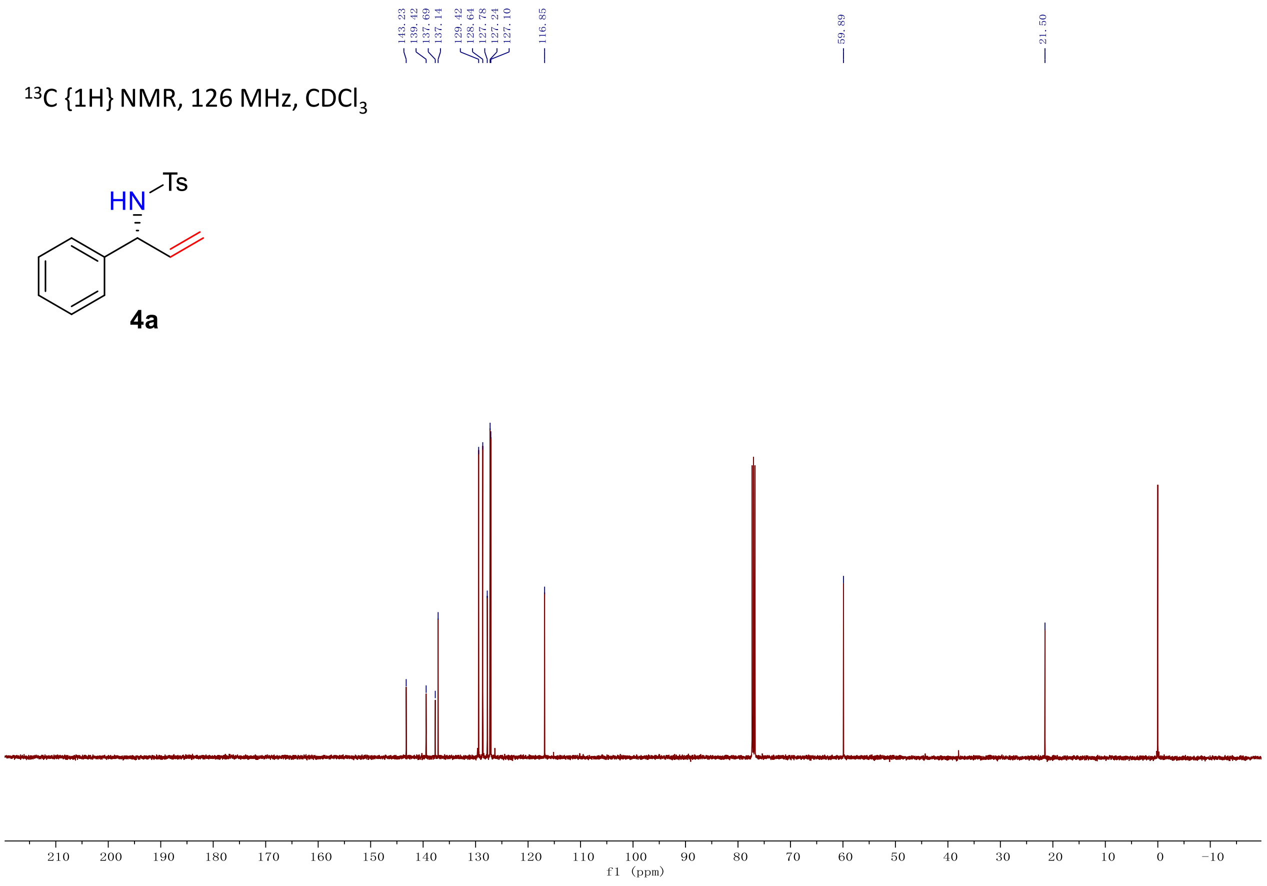

**4a**

## 13. HPLC Chromatograms

### HPLC Chromatogram of compound 3a *(racemic product)*

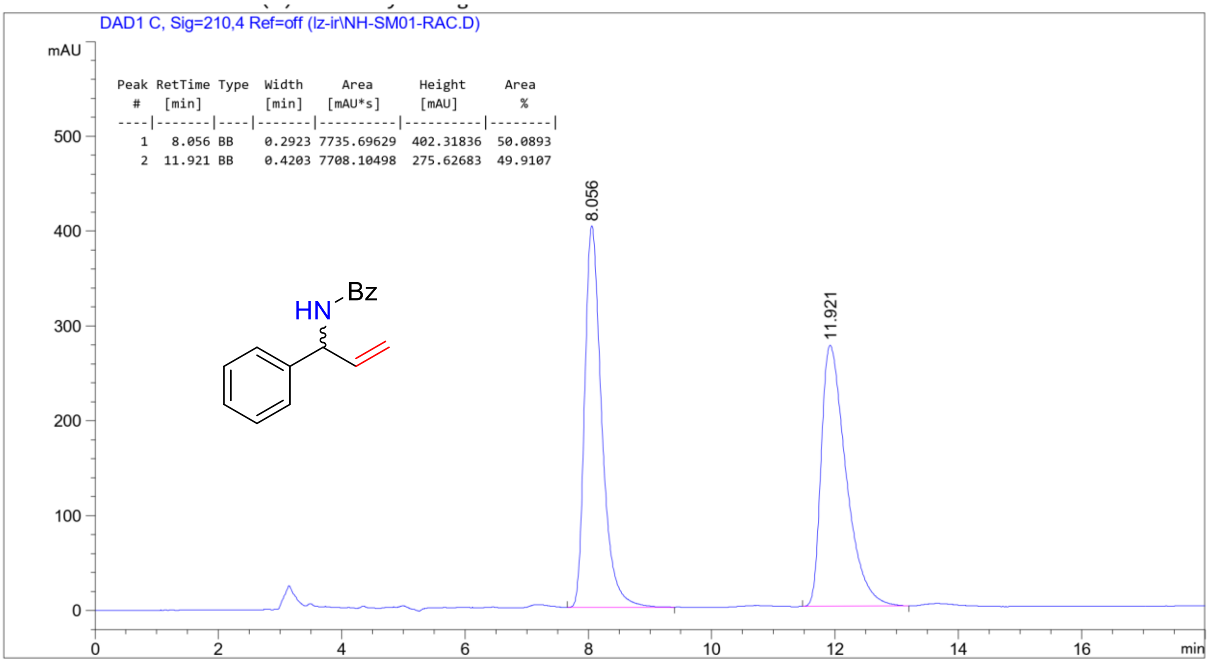


### HPLC Chromatogram of compound 3a *(chiral product)*

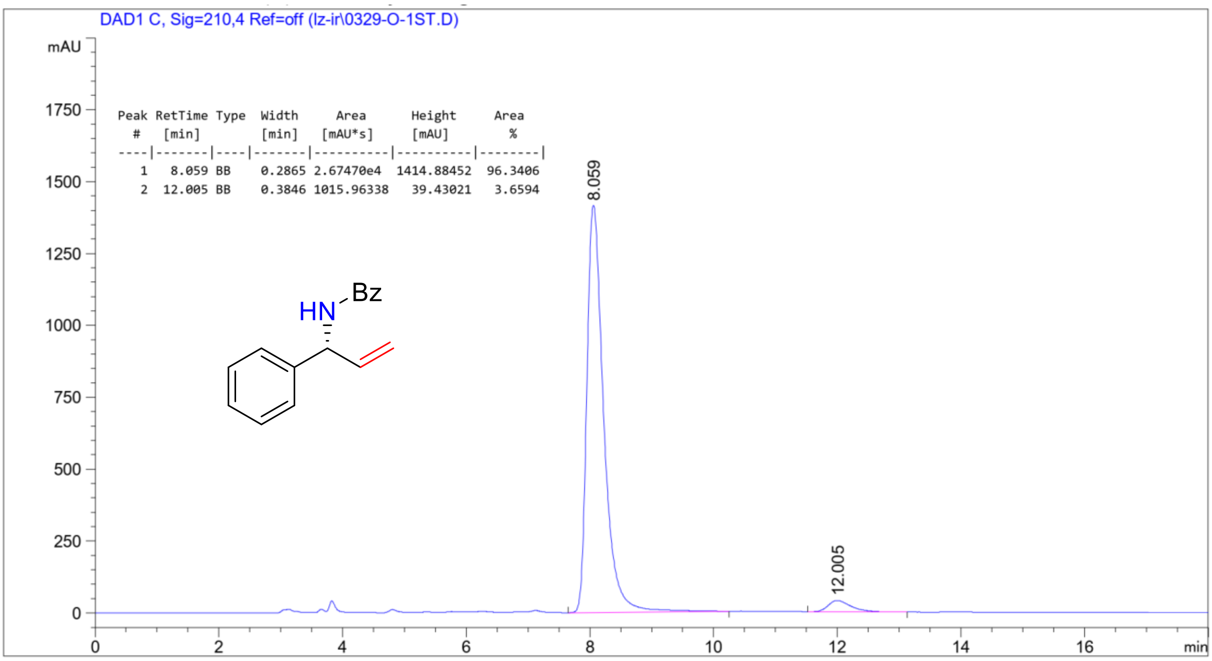

## HPLC Chromatogram of compound 3b *(racemic product)*

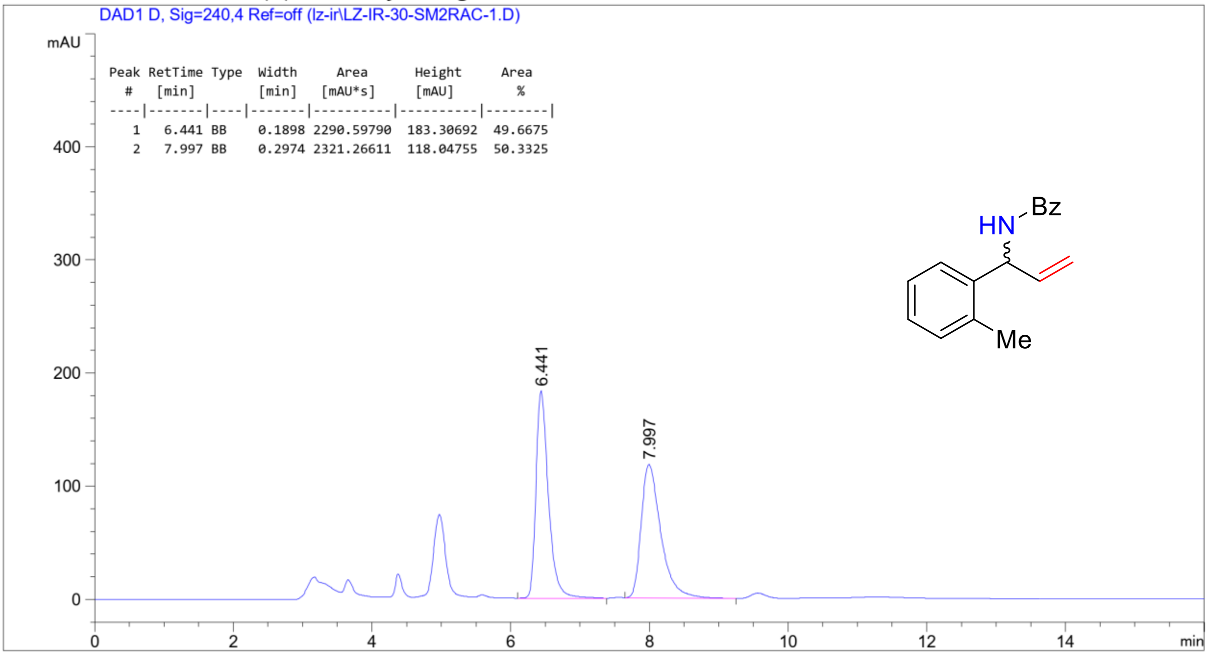


| Peak # | RetTime [min] | Type | Width [min] | Area [mAU*s] | Height [mAU] | Area % |
|---|---|---|---|---|---|---|
| 1 | 6.441 | BB | 0.1898 | 2290.59790 | 183.30692 | 49.6675 |
| 2 | 7.997 | BB | 0.2974 | 2321.26611 | 118.04755 | 50.3325 |

## HPLC Chromatogram of compound 3b *(chiral product)*

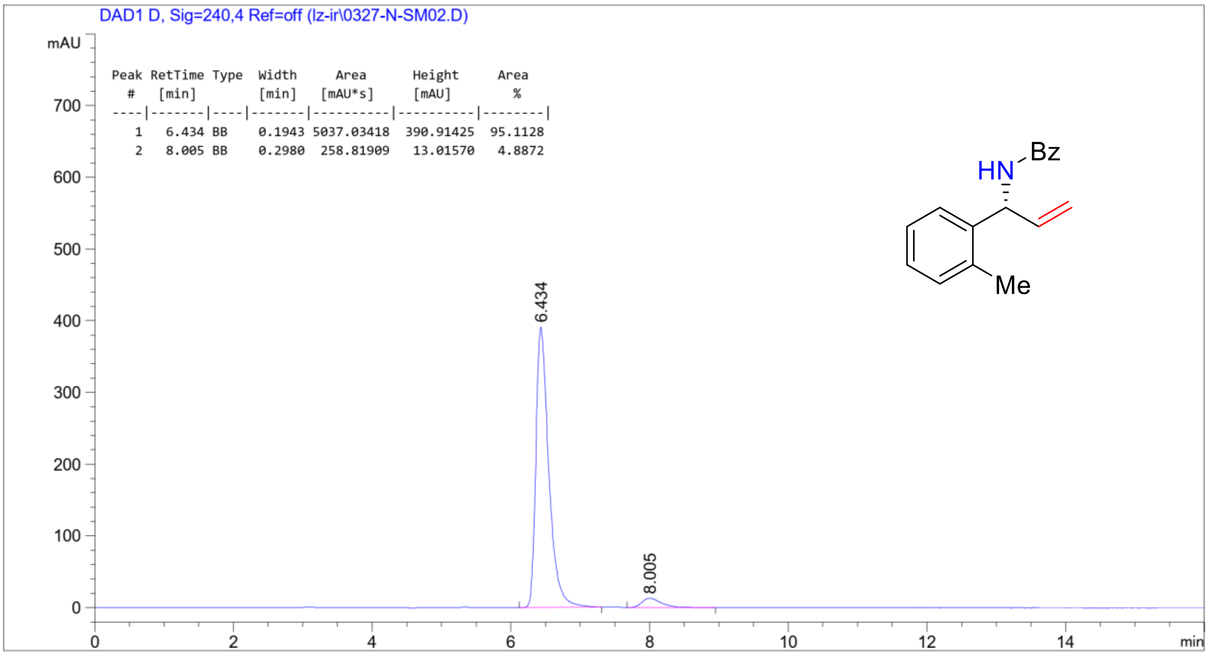


| Peak # | RetTime [min] | Type | Width [min] | Area [mAU*s] | Height [mAU] | Area % |
|---|---|---|---|---|---|---|
| 1 | 6.434 | BB | 0.1943 | 5037.03418 | 390.91425 | 95.1128 |
| 2 | 8.005 | BB | 0.2980 | 258.81909 | 13.01570 | 4.8872 |

## HPLC Chromatogram of compound 3c *(racemic product)*

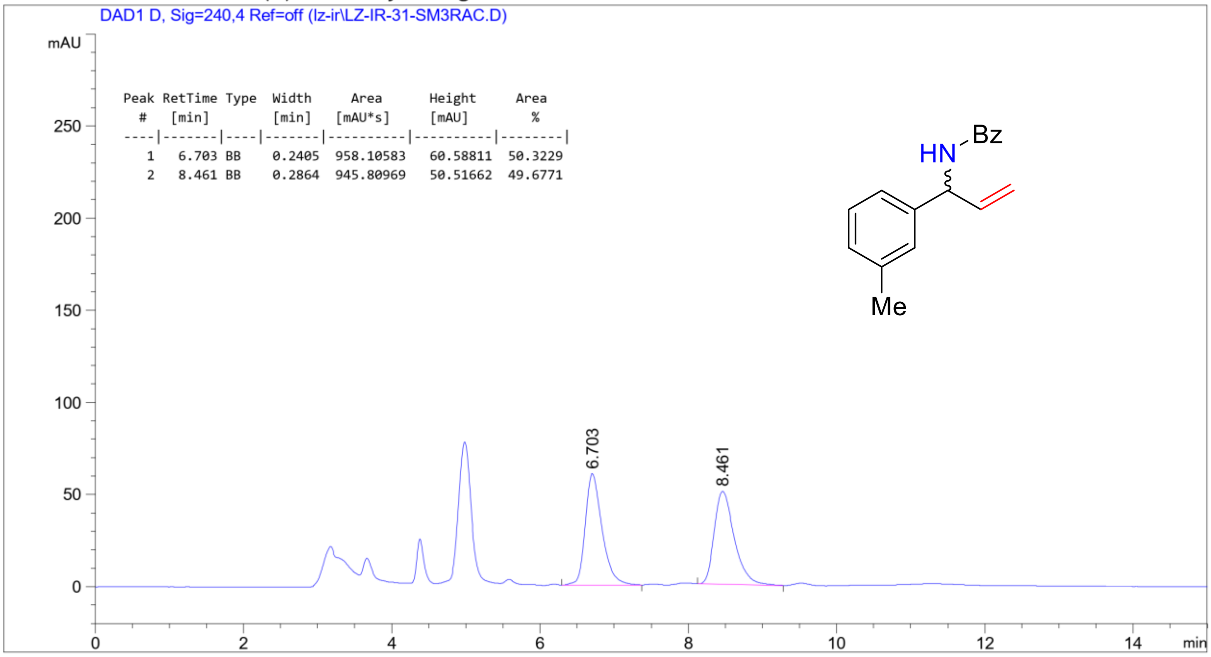


| Peak # | RetTime [min] | Type | Width [min] | Area [mAU*s] | Height [mAU] | Area % |
|---|---|---|---|---|---|---|
| 1 | 6.703 | BB | 0.2405 | 958.10583 | 60.58811 | 50.3229 |
| 2 | 8.461 | BB | 0.2864 | 945.80969 | 50.51662 | 49.6771 |

## HPLC Chromatogram of compound 3c *(chiral product)*

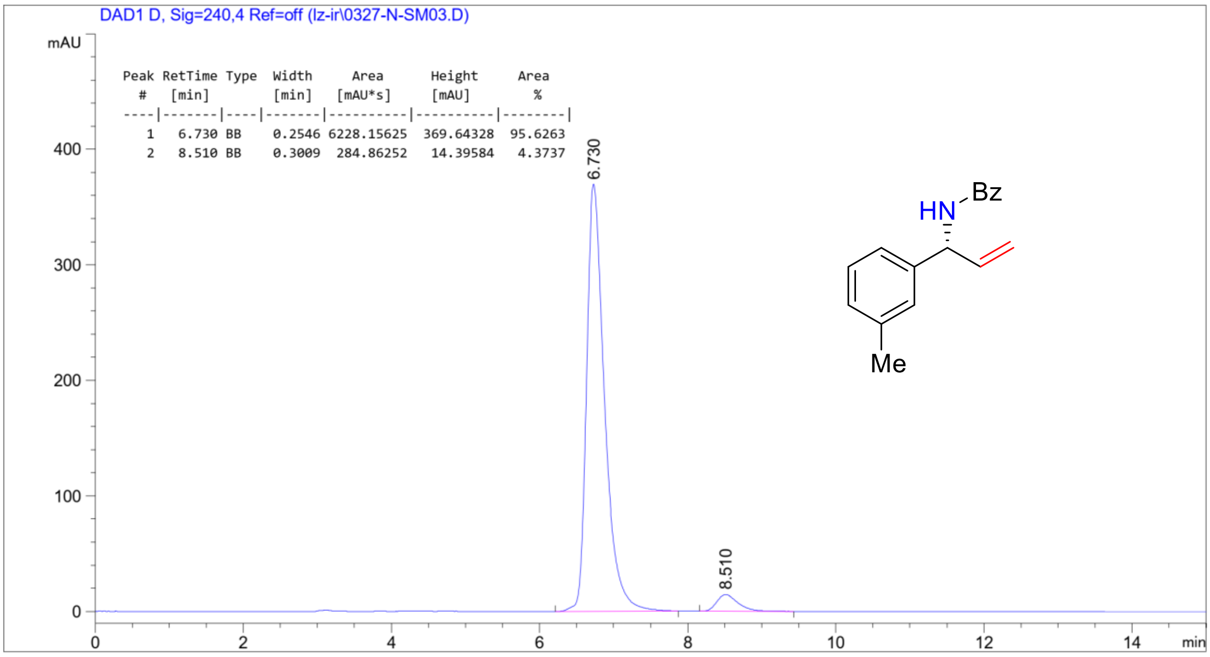


| Peak # | RetTime [min] | Type | Width [min] | Area [mAU*s] | Height [mAU] | Area % |
|---|---|---|---|---|---|---|
| 1 | 6.730 | BB | 0.2546 | 6228.15625 | 369.64328 | 95.6263 |
| 2 | 8.510 | BB | 0.3009 | 284.86252 | 14.39584 | 4.3737 |

## HPLC Chromatogram of compound 3d *(racemic product)*

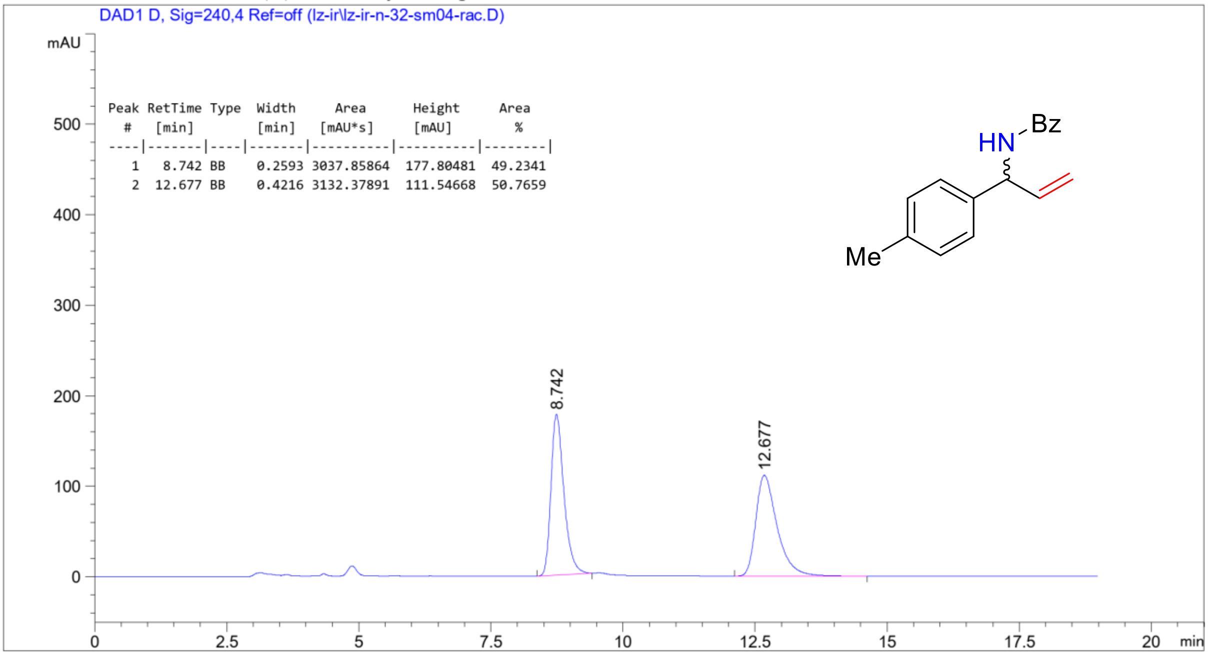


| Peak # | RetTime [min] | Type | Width [min] | Area [mAU*s] | Height [mAU] | Area % |
|---|---|---|---|---|---|---|
| 1 | 8.742 | BB | 0.2593 | 3037.85864 | 177.80481 | 49.2341 |
| 2 | 12.677 | BB | 0.4216 | 3132.37891 | 111.54668 | 50.7659 |

## HPLC Chromatogram of compound 3d *(chiral product)*

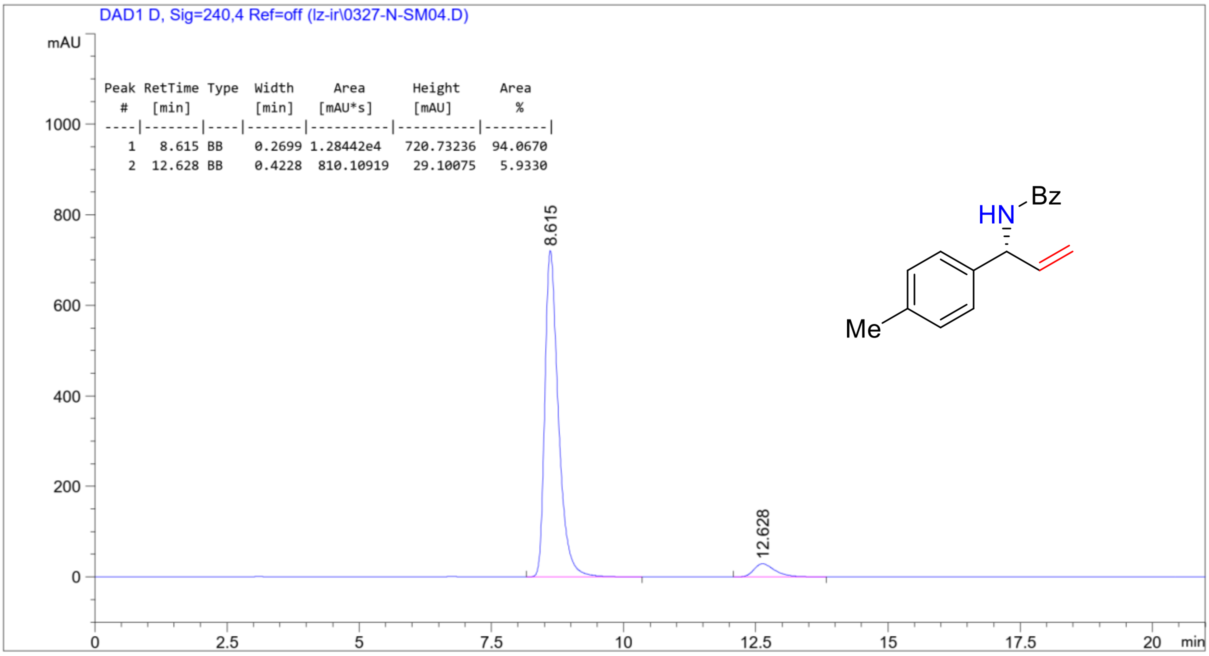


| Peak # | RetTime [min] | Type | Width [min] | Area [mAU*s] | Height [mAU] | Area % |
|---|---|---|---|---|---|---|
| 1 | 8.615 | BB | 0.2699 | 1.28442e4 | 720.73236 | 94.0670 |
| 2 | 12.628 | BB | 0.4228 | 810.10919 | 29.10075 | 5.9330 |

## HPLC Chromatogram of compound 3e *(racemic product)*

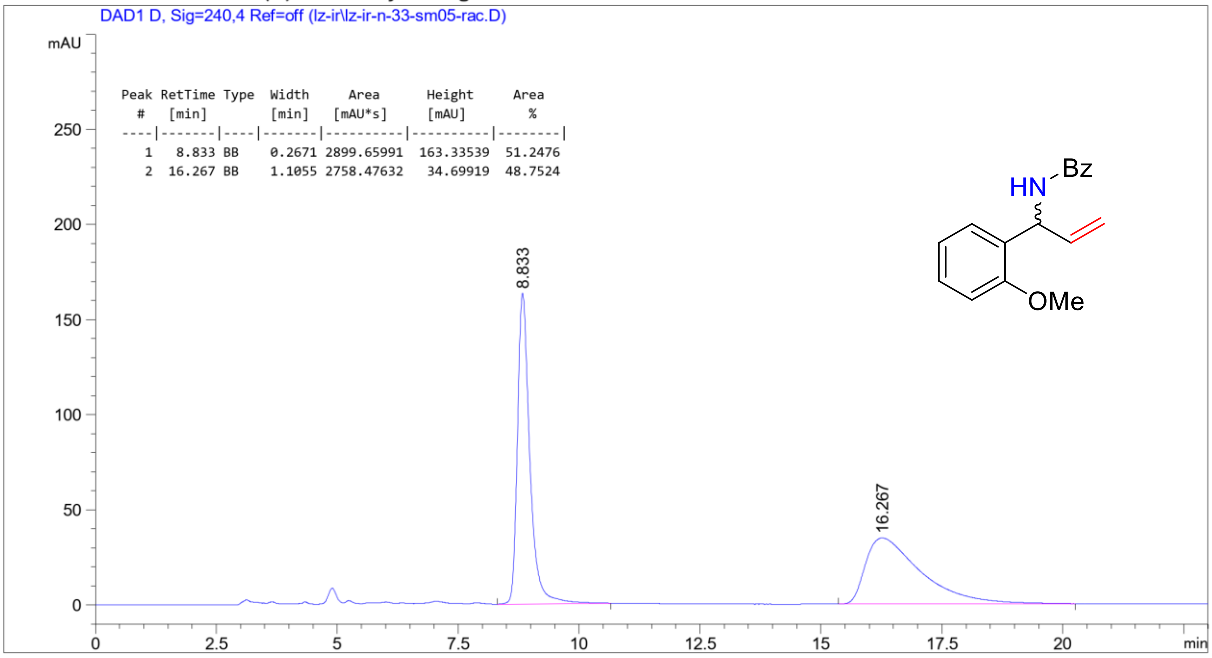


| Peak # | RetTime [min] | Type | Width [min] | Area [mAU*s] | Height [mAU] | Area % |
|---|---|---|---|---|---|---|
| 1 | 8.833 | BB | 0.2671 | 2899.65991 | 163.33539 | 51.2476 |
| 2 | 16.267 | BB | 1.1055 | 2758.47632 | 34.69919 | 48.7524 |

## HPLC Chromatogram of compound 3e *(chiral product)*

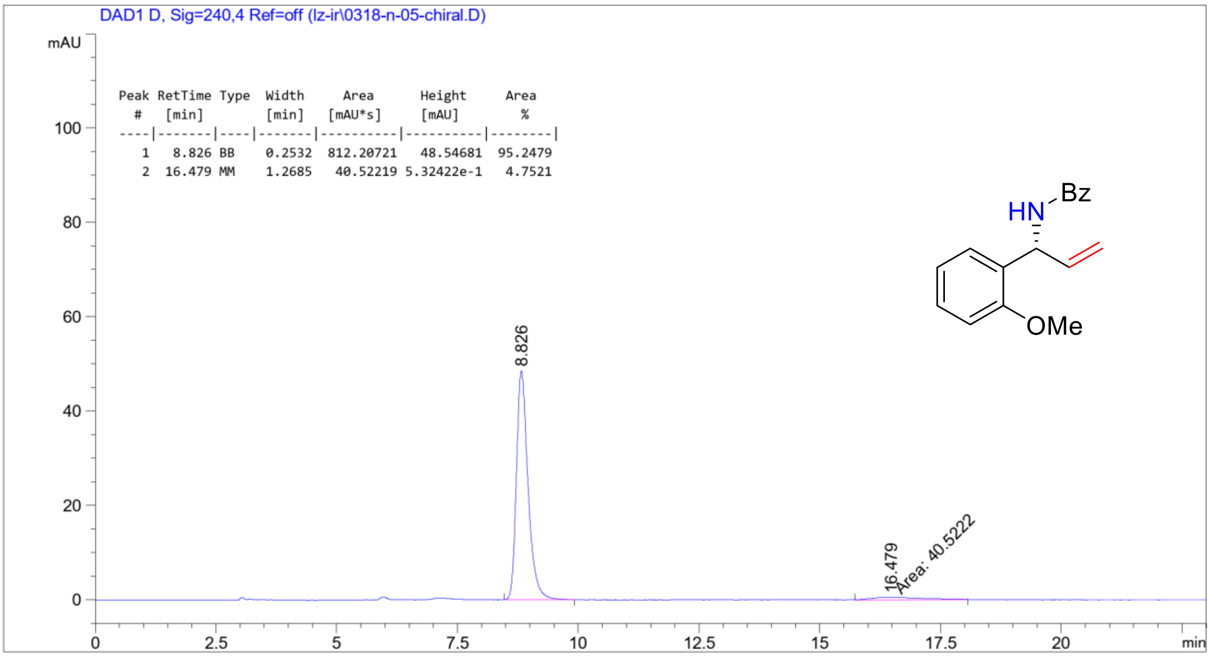


| Peak # | RetTime [min] | Type | Width [min] | Area [mAU*s] | Height [mAU] | Area % |
|---|---|---|---|---|---|---|
| 1 | 8.826 | BB | 0.2532 | 812.20721 | 48.54681 | 95.2479 |
| 2 | 16.479 | MM | 1.2685 | 40.52219 | 5.32422e-1 | 4.7521 |

## HPLC Chromatogram of compound 3f *(racemic product)*

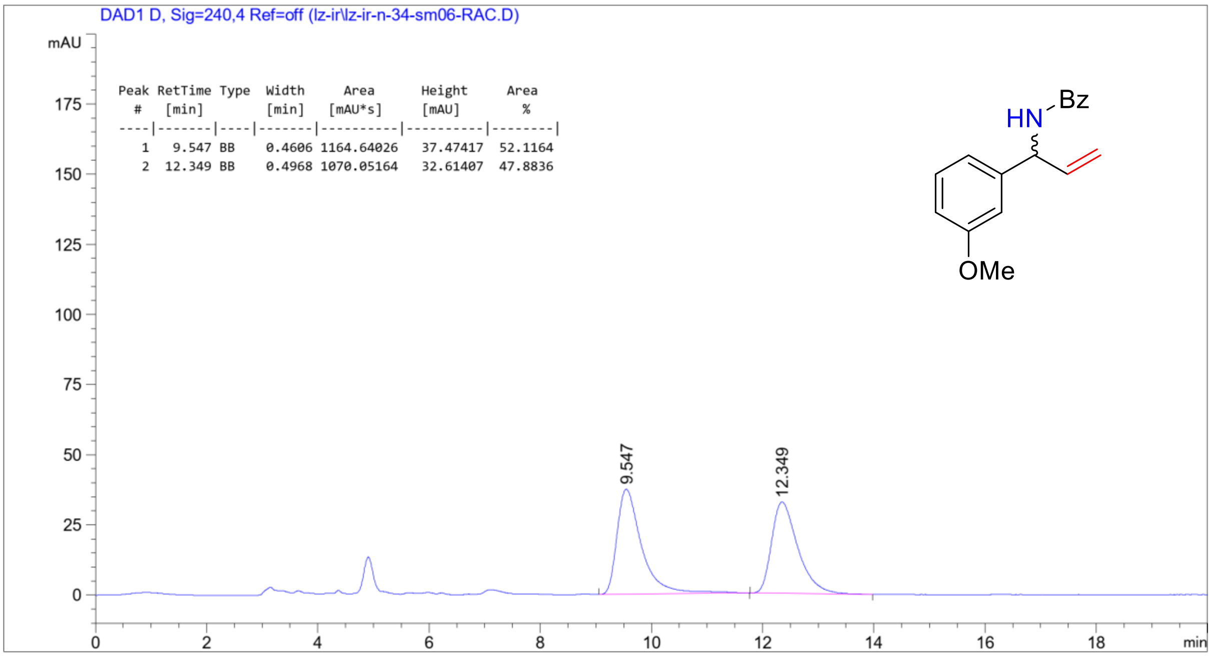


| Peak # | RetTime [min] | Type | Width [min] | Area [mAU*s] | Height [mAU] | Area % |
|---|---|---|---|---|---|---|
| 1 | 9.547 | BB | 0.4606 | 1164.64026 | 37.47417 | 52.1164 |
| 2 | 12.349 | BB | 0.4968 | 1070.05164 | 32.61407 | 47.8836 |

## HPLC Chromatogram of compound 3f *(chiral product)*

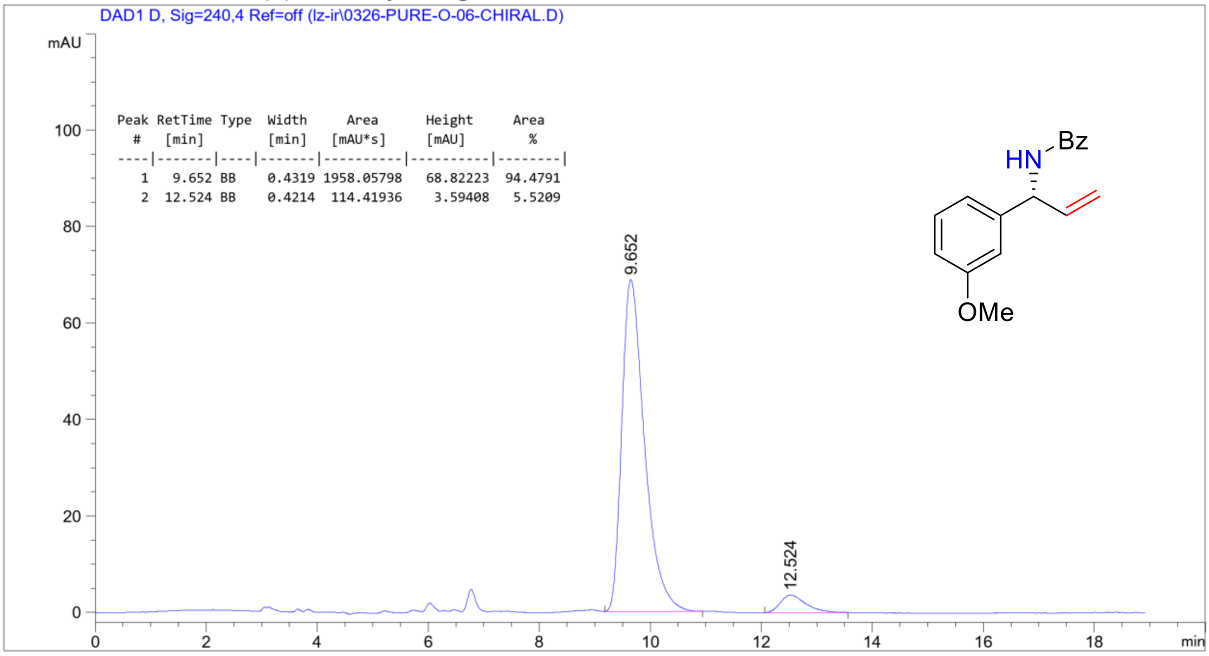


| Peak # | RetTime [min] | Type | Width [min] | Area [mAU*s] | Height [mAU] | Area % |
|---|---|---|---|---|---|---|
| 1 | 9.652 | BB | 0.4319 | 1958.05798 | 68.82223 | 94.4791 |
| 2 | 12.524 | BB | 0.4214 | 114.41936 | 3.59408 | 5.5209 |

## HPLC Chromatogram of compound 3g *(racemic product)*

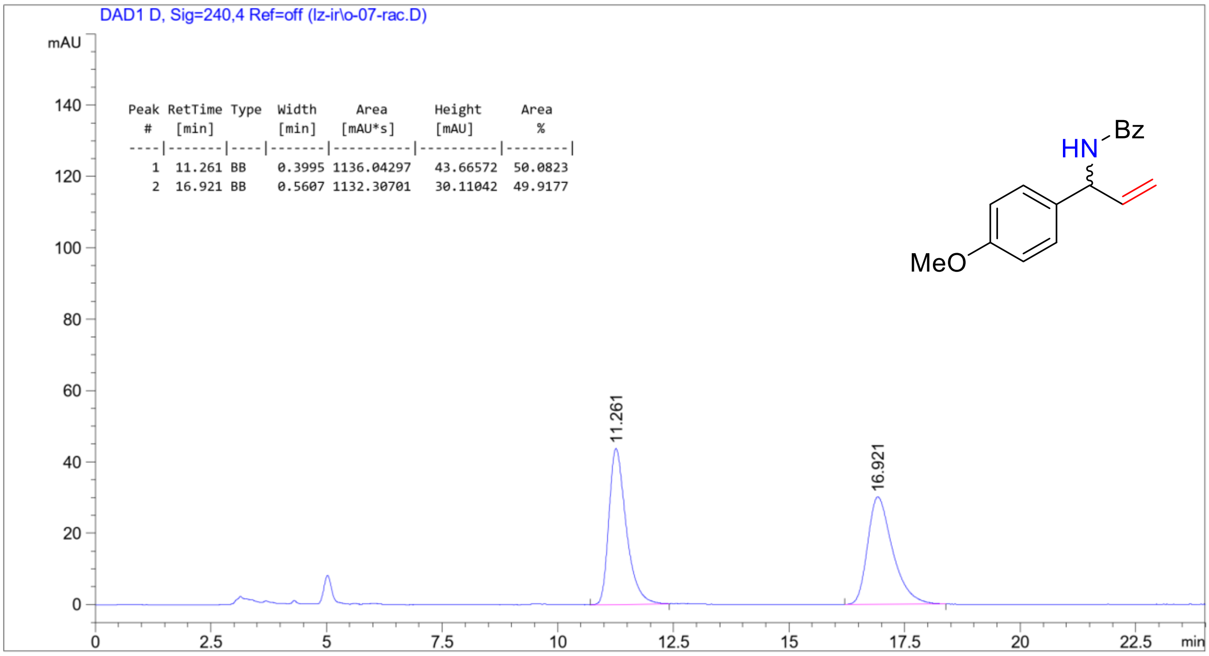


## HPLC Chromatogram of compound 3g *(chiral product)*

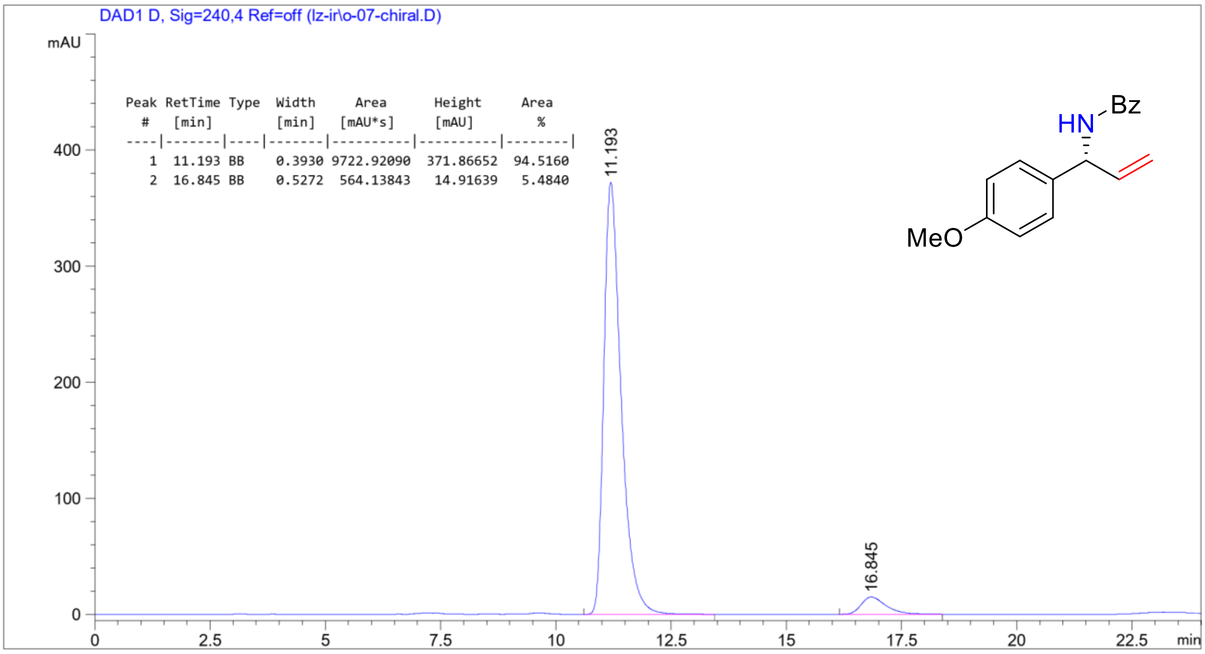

## HPLC Chromatogram of compound 3h *(racemic product)*

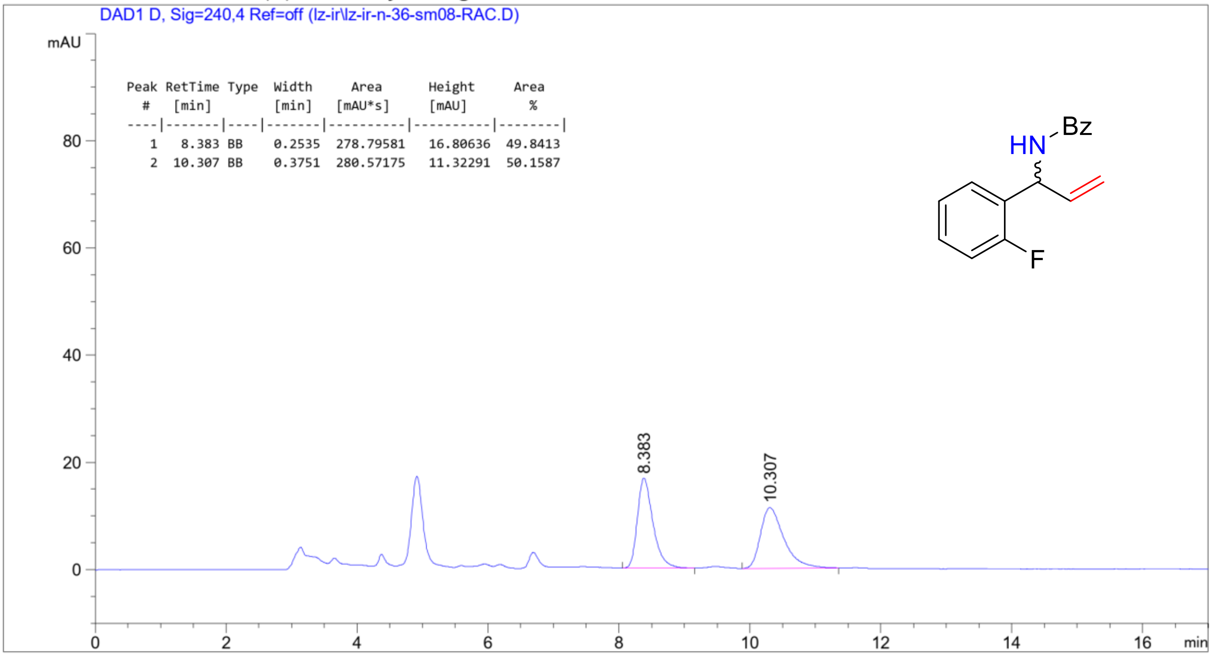


| Peak # | RetTime [min] | Type | Width [min] | Area [mAU*s] | Height [mAU] | Area % |
|---|---|---|---|---|---|---|
| 1 | 8.383 | BB | 0.2535 | 278.79581 | 16.80636 | 49.8413 |
| 2 | 10.307 | BB | 0.3751 | 280.57175 | 11.32291 | 50.1587 |

## HPLC Chromatogram of compound 3h *(chiral product)*

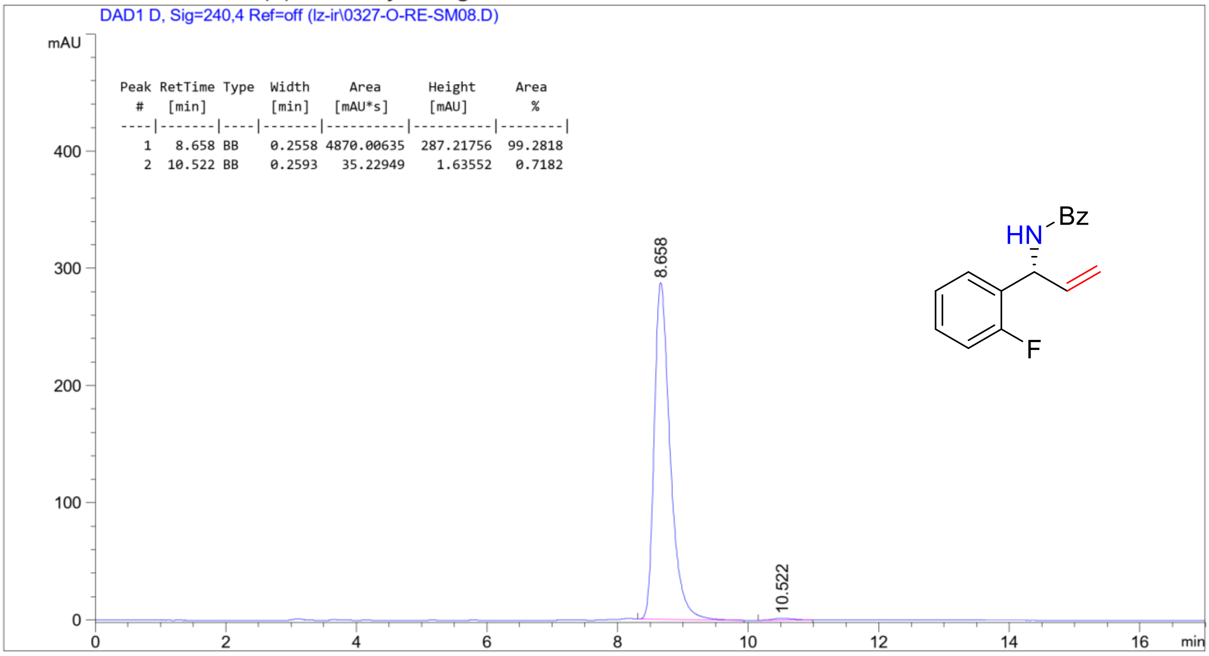


| Peak # | RetTime [min] | Type | Width [min] | Area [mAU*s] | Height [mAU] | Area % |
|---|---|---|---|---|---|---|
| 1 | 8.658 | BB | 0.2558 | 4870.00635 | 287.21756 | 99.2818 |
| 2 | 10.522 | BB | 0.2593 | 35.22949 | 1.63552 | 0.7182 |

## HPLC Chromatogram of compound 3i *(racemic product)*

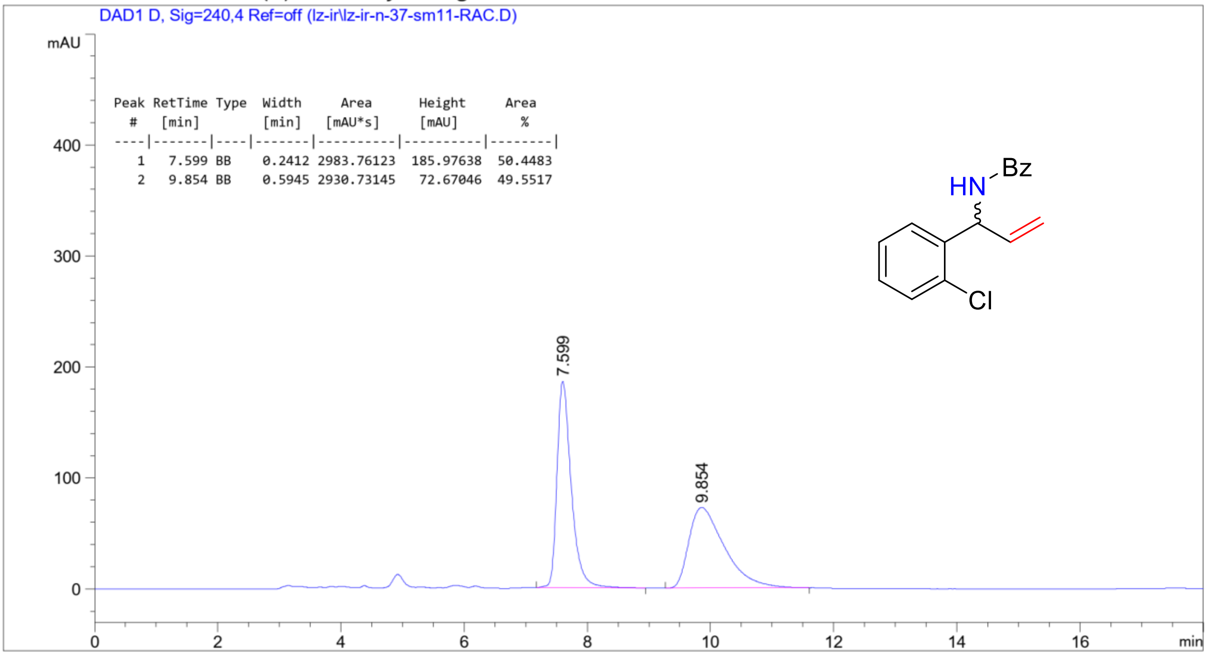


| Peak # | RetTime [min] | Type | Width [min] | Area [mAU*s] | Height [mAU] | Area % |
|---|---|---|---|---|---|---|
| 1 | 7.599 | BB | 0.2412 | 2983.76123 | 185.97638 | 50.4483 |
| 2 | 9.854 | BB | 0.5945 | 2930.73145 | 72.67046 | 49.5517 |

## HPLC Chromatogram of compound 3i *(chiral product)*

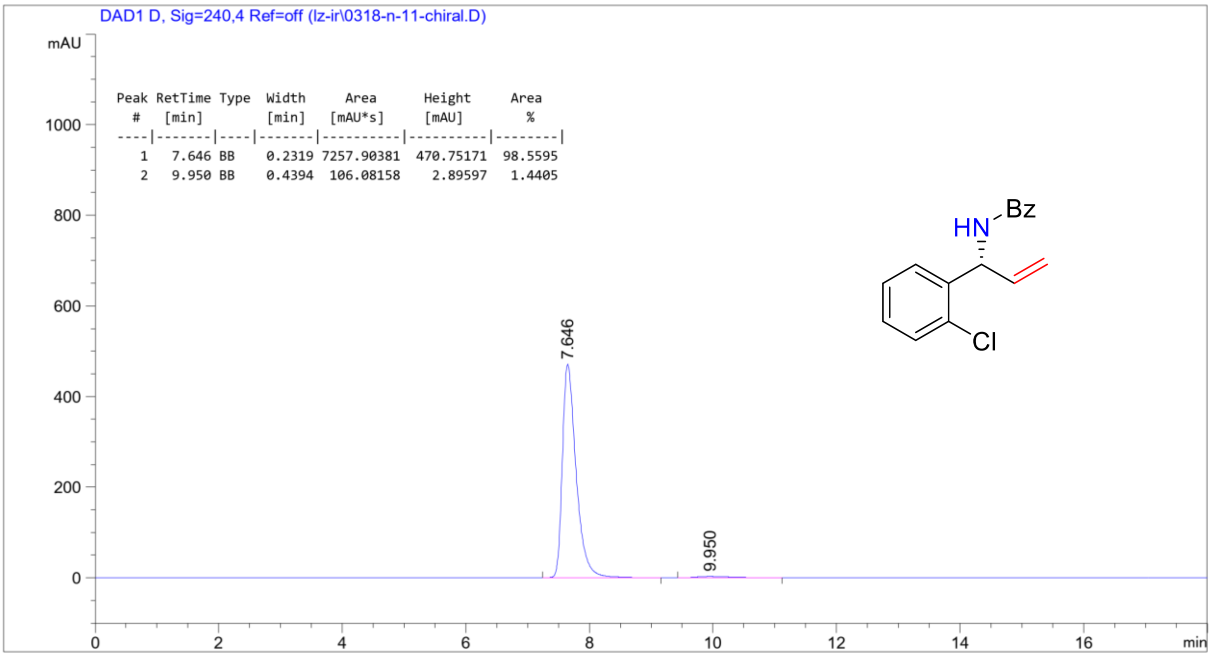


| Peak # | RetTime [min] | Type | Width [min] | Area [mAU*s] | Height [mAU] | Area % |
|---|---|---|---|---|---|---|
| 1 | 7.646 | BB | 0.2319 | 7257.90381 | 470.75171 | 98.5595 |
| 2 | 9.950 | BB | 0.4394 | 106.08158 | 2.89597 | 1.4405 |

## HPLC Chromatogram of compound 3j *(racemic product)*

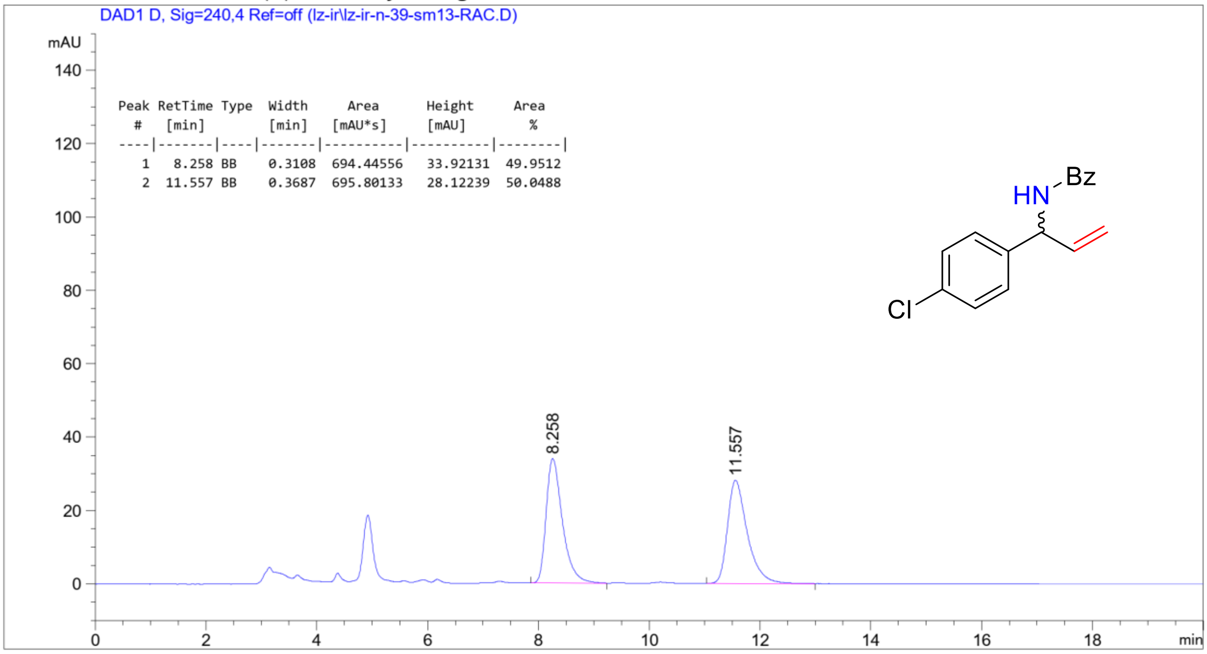


| Peak # | RetTime [min] | Type | Width [min] | Area [mAU*s] | Height [mAU] | Area % |
|---|---|---|---|---|---|---|
| 1 | 8.258 | BB | 0.3108 | 694.44556 | 33.92131 | 49.9512 |
| 2 | 11.557 | BB | 0.3687 | 695.80133 | 28.12239 | 50.0488 |

## HPLC Chromatogram of compound 3j *(chiral product)*

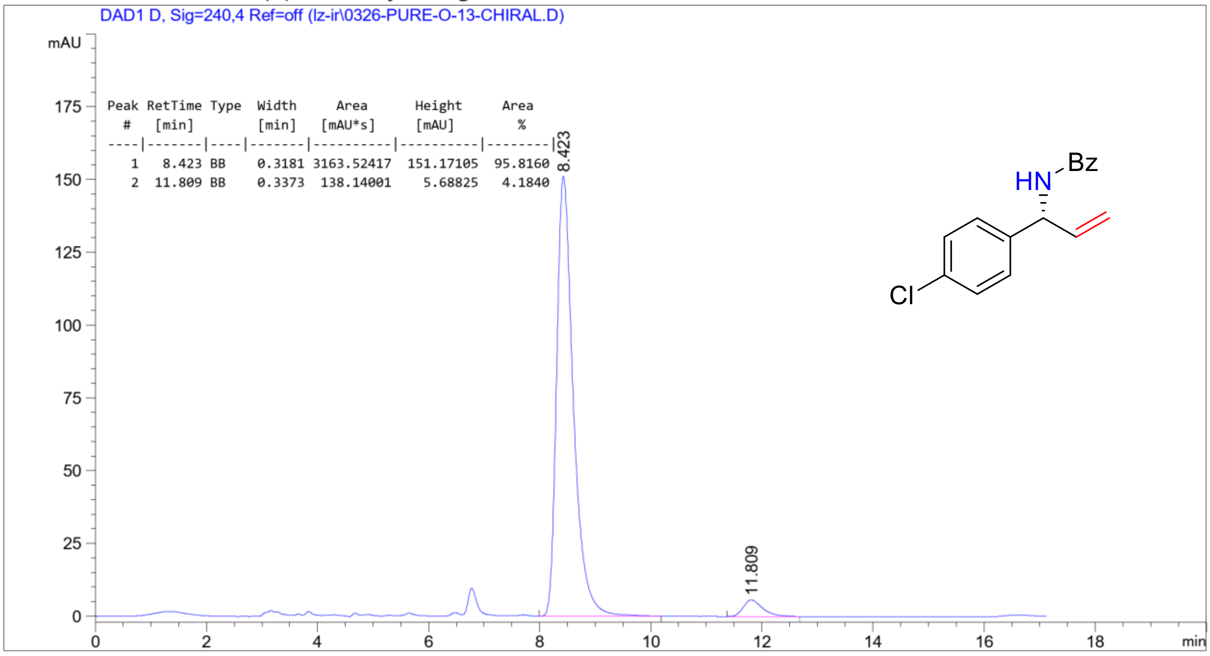


| Peak # | RetTime [min] | Type | Width [min] | Area [mAU*s] | Height [mAU] | Area % |
|---|---|---|---|---|---|---|
| 1 | 8.423 | BB | 0.3181 | 3163.52417 | 151.17105 | 95.8160 |
| 2 | 11.809 | BB | 0.3373 | 138.14001 | 5.68825 | 4.1840 |

## HPLC Chromatogram of compound 3k *(racemic product)*

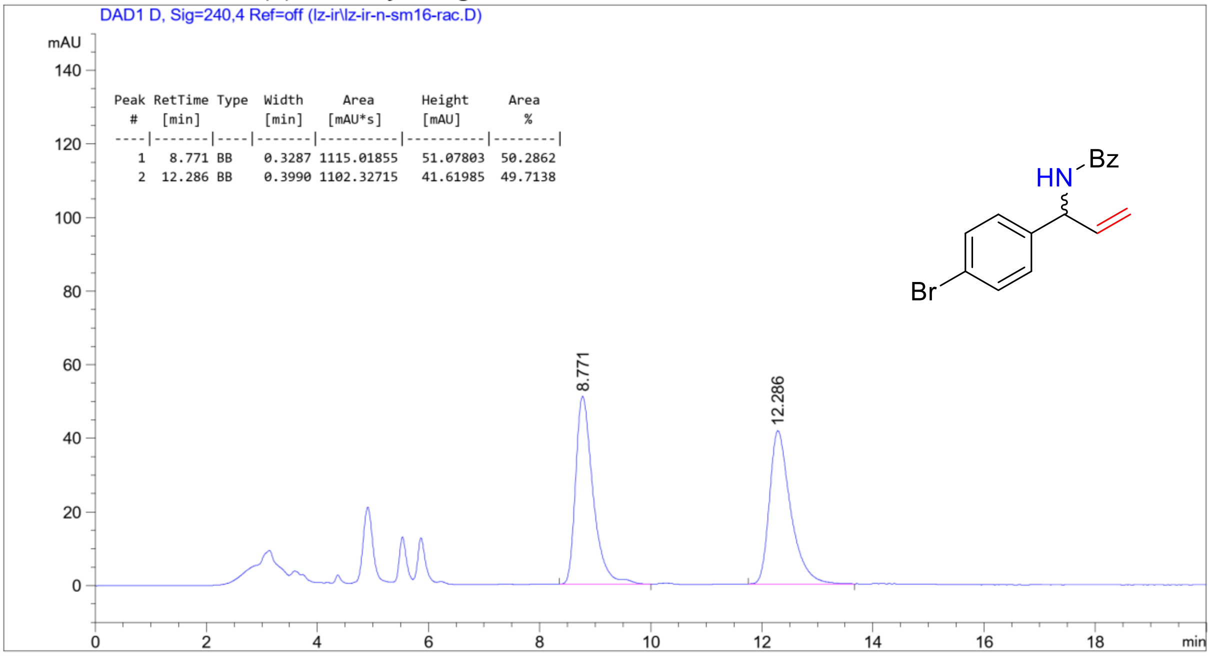


| Peak # | RetTime [min] | Type | Width [min] | Area [mAU*s] | Height [mAU] | Area % |
|---|---|---|---|---|---|---|
| 1 | 8.771 | BB | 0.3287 | 1115.01855 | 51.07803 | 50.2862 |
| 2 | 12.286 | BB | 0.3990 | 1102.32715 | 41.61985 | 49.7138 |

## HPLC Chromatogram of compound 3k *(chiral product)*

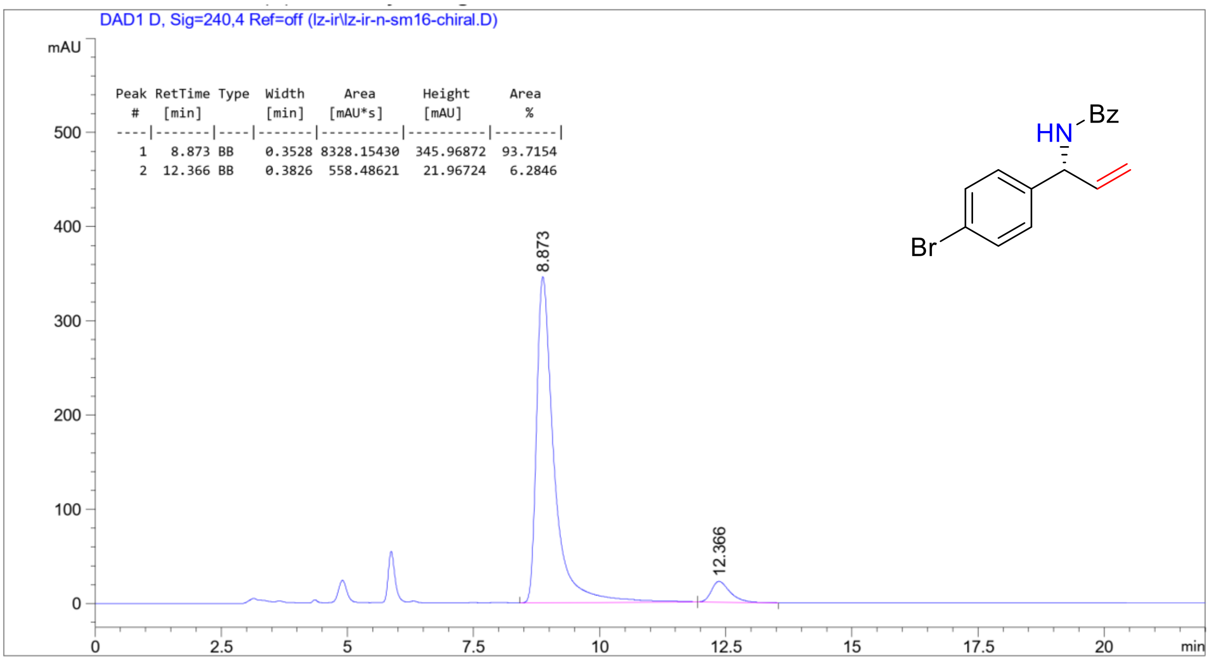


| Peak # | RetTime [min] | Type | Width [min] | Area [mAU*s] | Height [mAU] | Area % |
|---|---|---|---|---|---|---|
| 1 | 8.873 | BB | 0.3528 | 8328.15430 | 345.96872 | 93.7154 |
| 2 | 12.366 | BB | 0.3826 | 558.48621 | 21.96724 | 6.2846 |

## HPLC Chromatogram of compound 3l *(racemic product)*

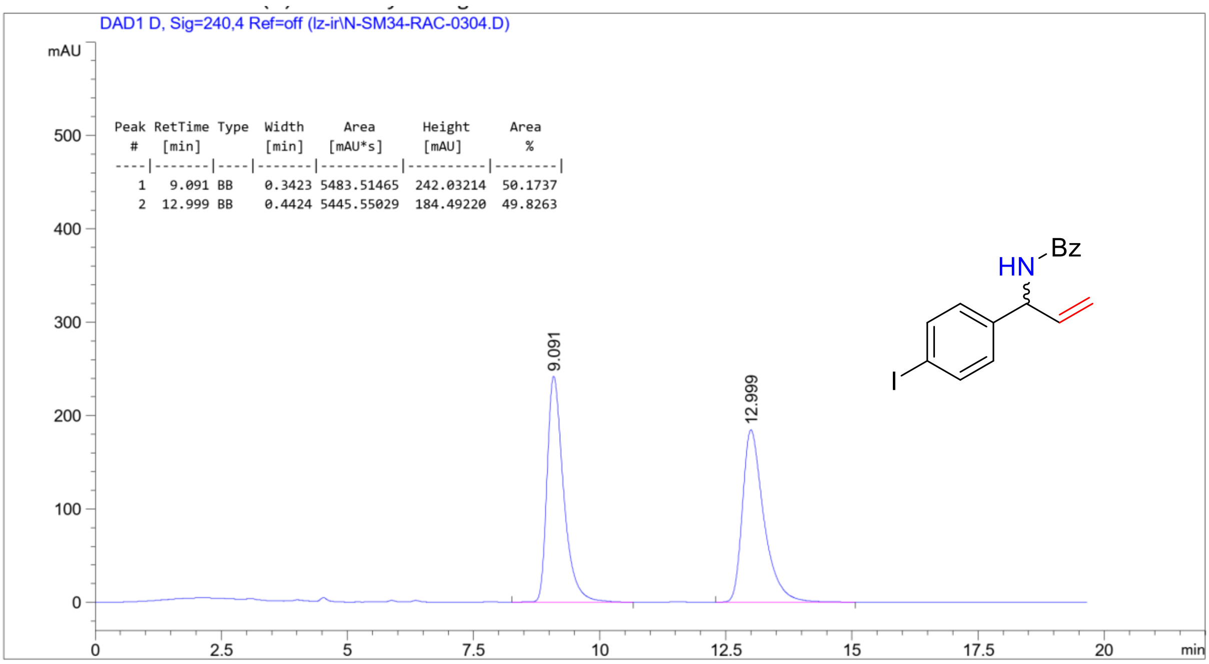


| Peak # | RetTime [min] | Type | Width [min] | Area [mAU*s] | Height [mAU] | Area % |
|---|---|---|---|---|---|---|
| 1 | 9.091 | BB | 0.3423 | 5483.51465 | 242.03214 | 50.1737 |
| 2 | 12.999 | BB | 0.4424 | 5445.55029 | 184.49220 | 49.8263 |

## HPLC Chromatogram of compound 3l *(chiral product)*

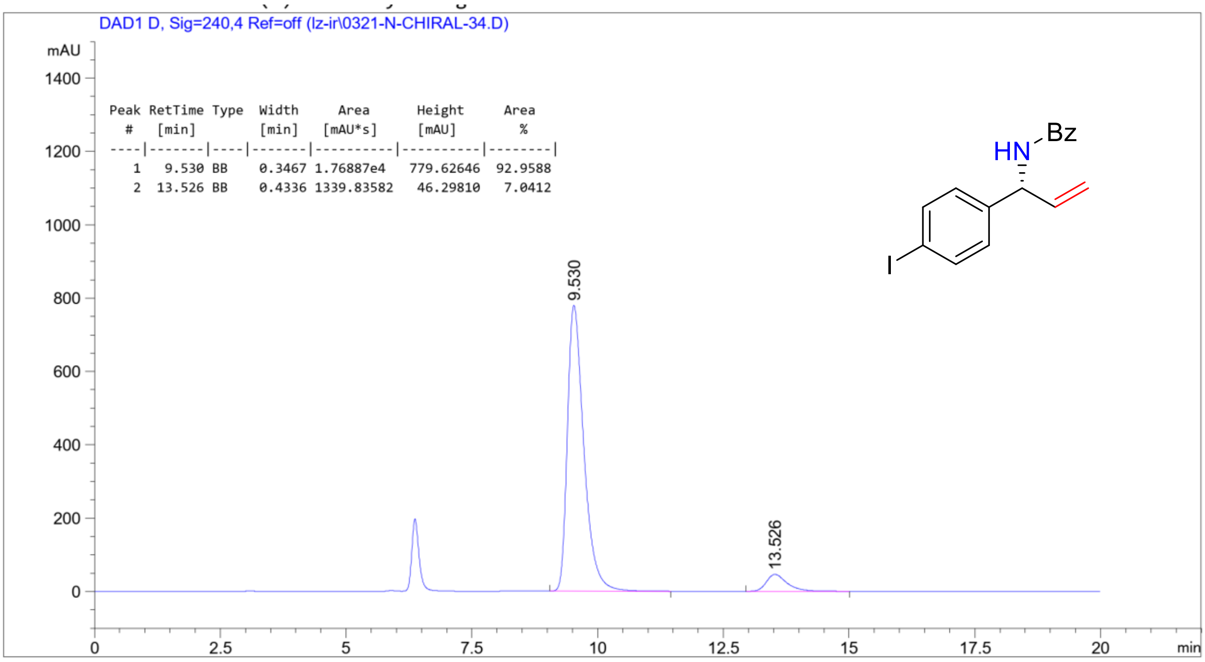


| Peak # | RetTime [min] | Type | Width [min] | Area [mAU*s] | Height [mAU] | Area % |
|---|---|---|---|---|---|---|
| 1 | 9.530 | BB | 0.3467 | 1.76887e4 | 779.62646 | 92.9588 |
| 2 | 13.526 | BB | 0.4336 | 1339.83582 | 46.29810 | 7.0412 |

## HPLC Chromatogram of compound 3m *(racemic product)*

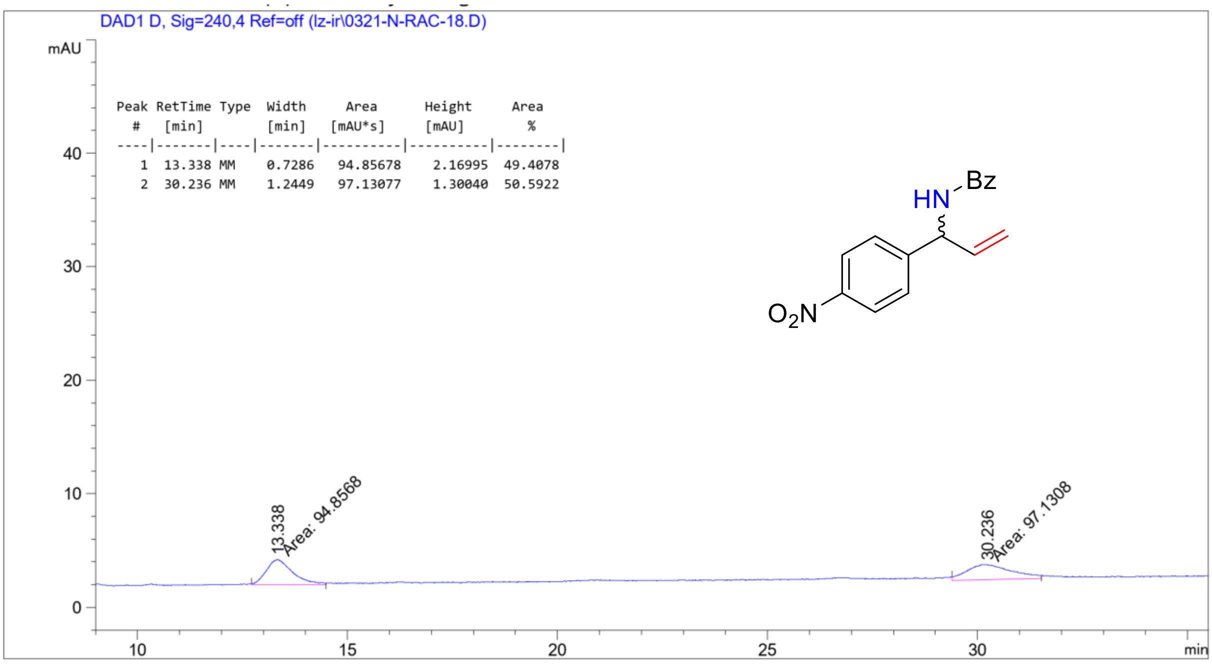


| Peak # | RetTime [min] | Type | Width [min] | Area [mAU*s] | Height [mAU] | Area % |
|---|---|---|---|---|---|---|
| 1 | 13.338 | MM | 0.7286 | 94.85678 | 2.16995 | 49.4078 |
| 2 | 30.236 | MM | 1.2449 | 97.13077 | 1.30040 | 50.5922 |

## HPLC Chromatogram of compound 3m *(chiral product)*

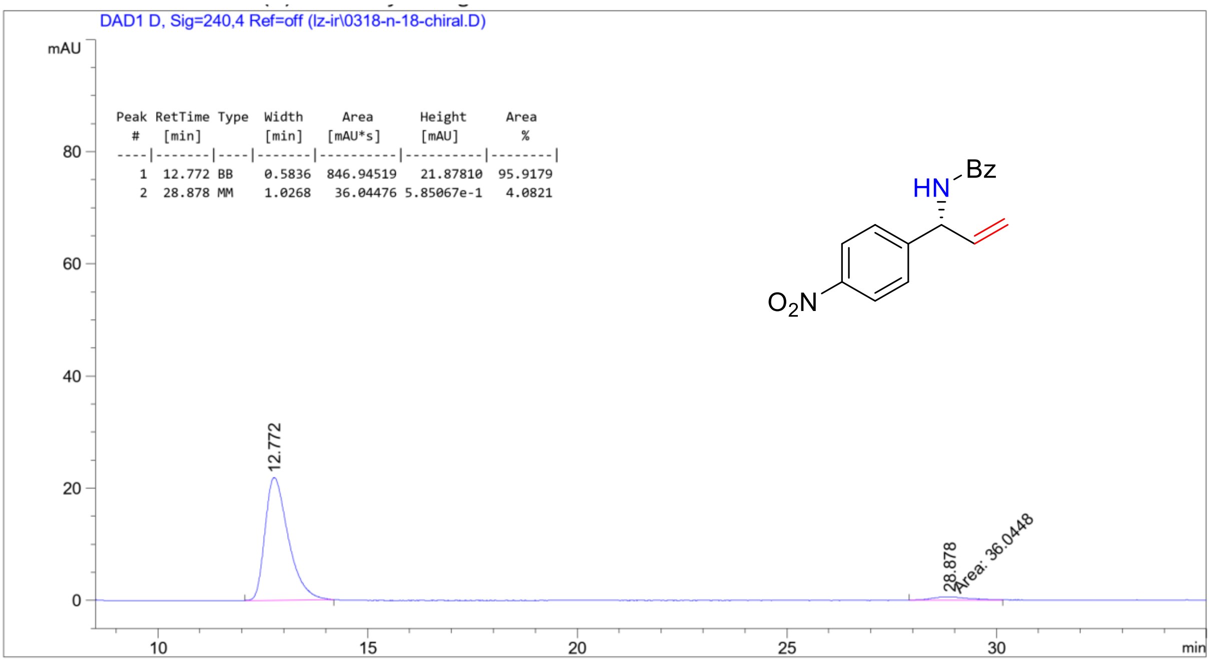


| Peak # | RetTime [min] | Type | Width [min] | Area [mAU*s] | Height [mAU] | Area % |
|---|---|---|---|---|---|---|
| 1 | 12.772 | BB | 0.5836 | 846.94519 | 21.87810 | 95.9179 |
| 2 | 28.878 | MM | 1.0268 | 36.04476 | 5.85067e-1 | 4.0821 |

## HPLC Chromatogram of compound 3n *(racemic product)*

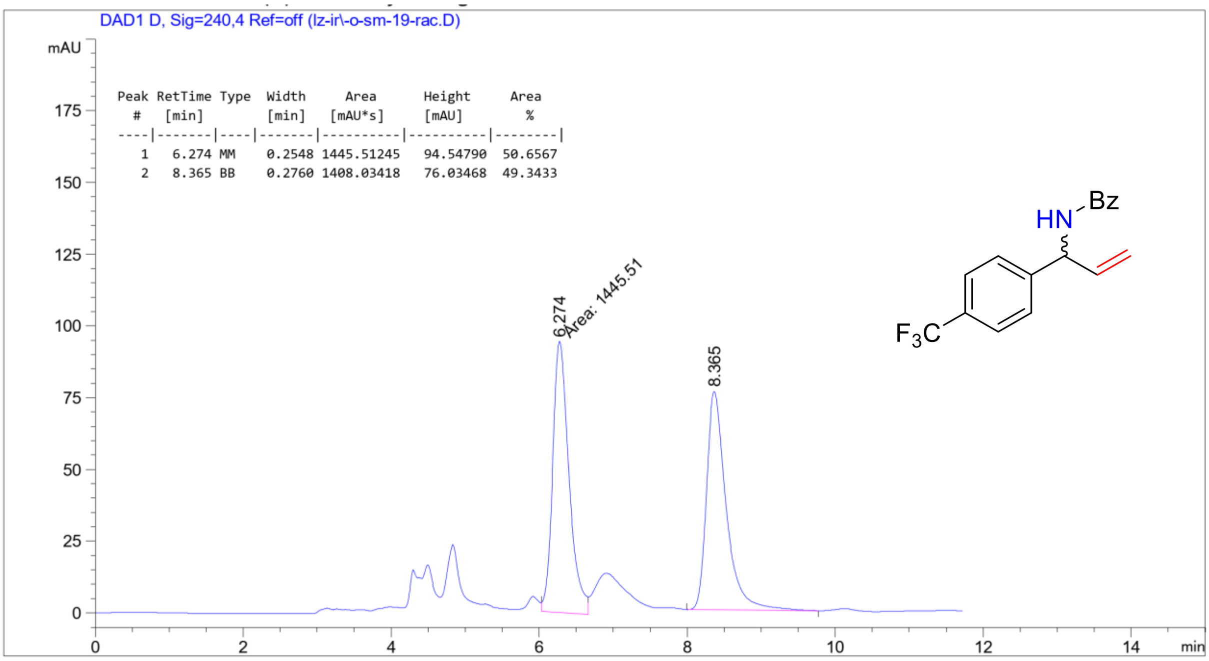


| Peak # | RetTime [min] | Type | Width [min] | Area [mAU*s] | Height [mAU] | Area % |
|---|---|---|---|---|---|---|
| 1 | 6.274 | MM | 0.2548 | 1445.51245 | 94.54790 | 50.6567 |
| 2 | 8.365 | BB | 0.2760 | 1408.03418 | 76.03468 | 49.3433 |

## HPLC Chromatogram of compound 3n *(chiral product)*

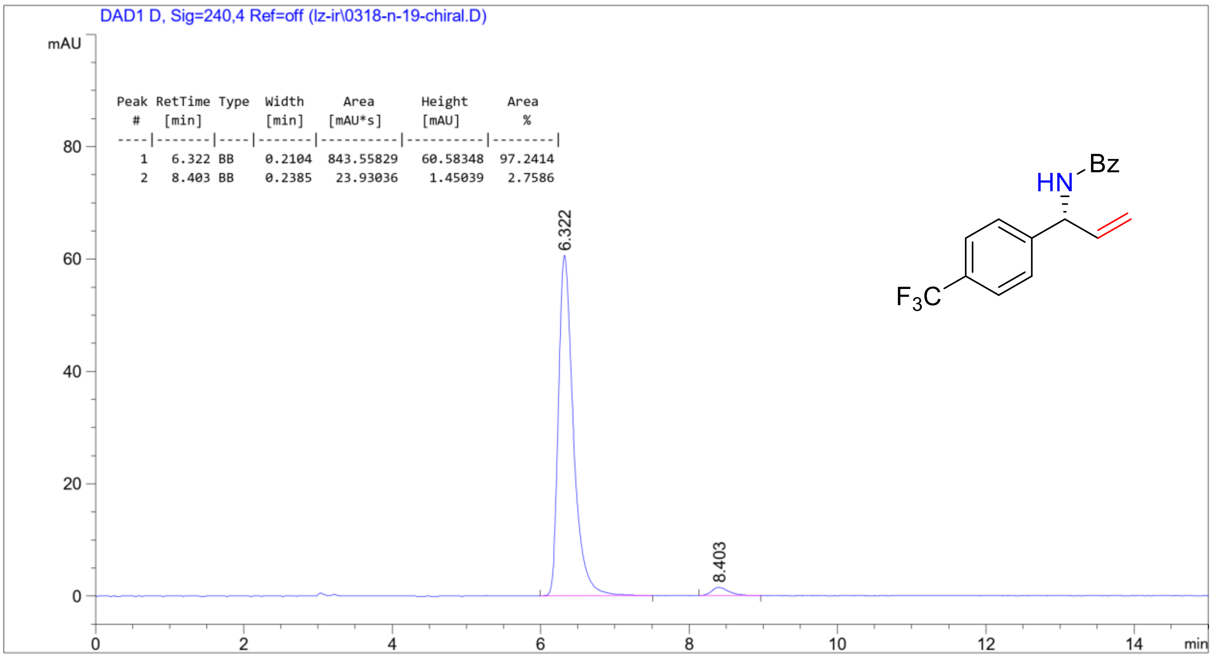


| Peak # | RetTime [min] | Type | Width [min] | Area [mAU*s] | Height [mAU] | Area % |
|---|---|---|---|---|---|---|
| 1 | 6.322 | BB | 0.2104 | 843.55829 | 60.58348 | 97.2414 |
| 2 | 8.403 | BB | 0.2385 | 23.93036 | 1.45039 | 2.7586 |

## HPLC Chromatogram of compound 3o *(racemic product)*

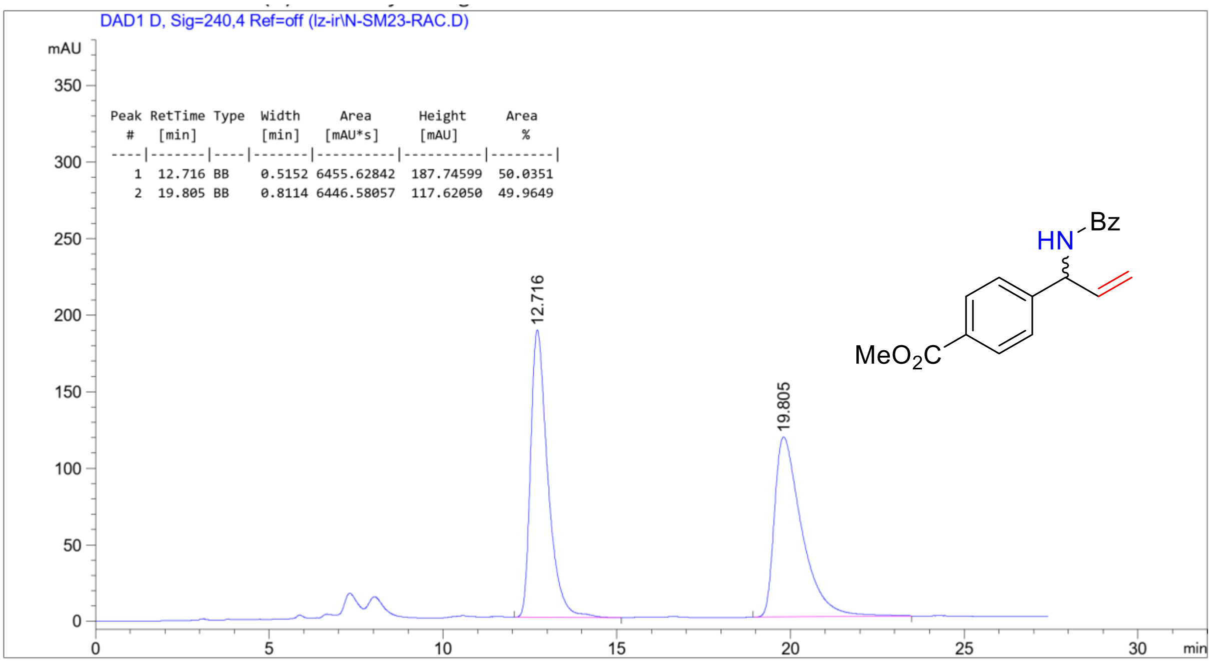


| Peak # | RetTime [min] | Type | Width [min] | Area [mAU*s] | Height [mAU] | Area % |
|---|---|---|---|---|---|---|
| 1 | 12.716 | BB | 0.5152 | 6455.62842 | 187.74599 | 50.0351 |
| 2 | 19.805 | BB | 0.8114 | 6446.58057 | 117.62050 | 49.9649 |

## HPLC Chromatogram of compound 3o *(chiral product)*

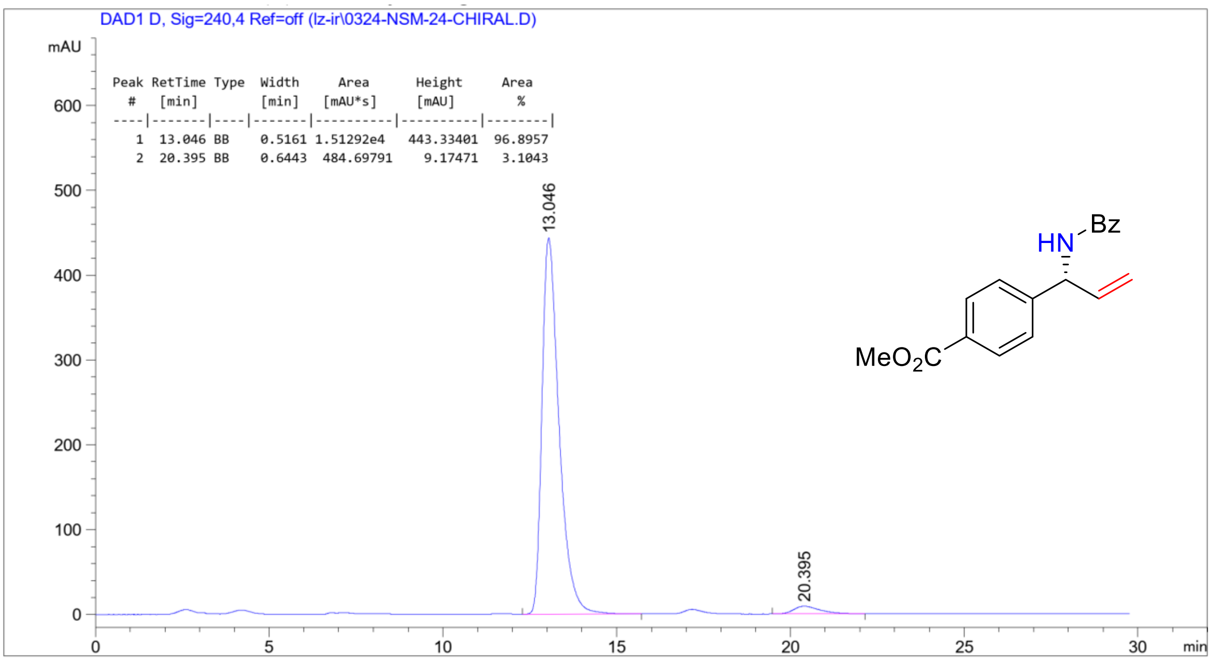


| Peak # | RetTime [min] | Type | Width [min] | Area [mAU*s] | Height [mAU] | Area % |
|---|---|---|---|---|---|---|
| 1 | 13.046 | BB | 0.5161 | 1.51292e4 | 443.33401 | 96.8957 |
| 2 | 20.395 | BB | 0.6443 | 484.69791 | 9.17471 | 3.1043 |

## HPLC Chromatogram of compound 3p *(racemic product)*

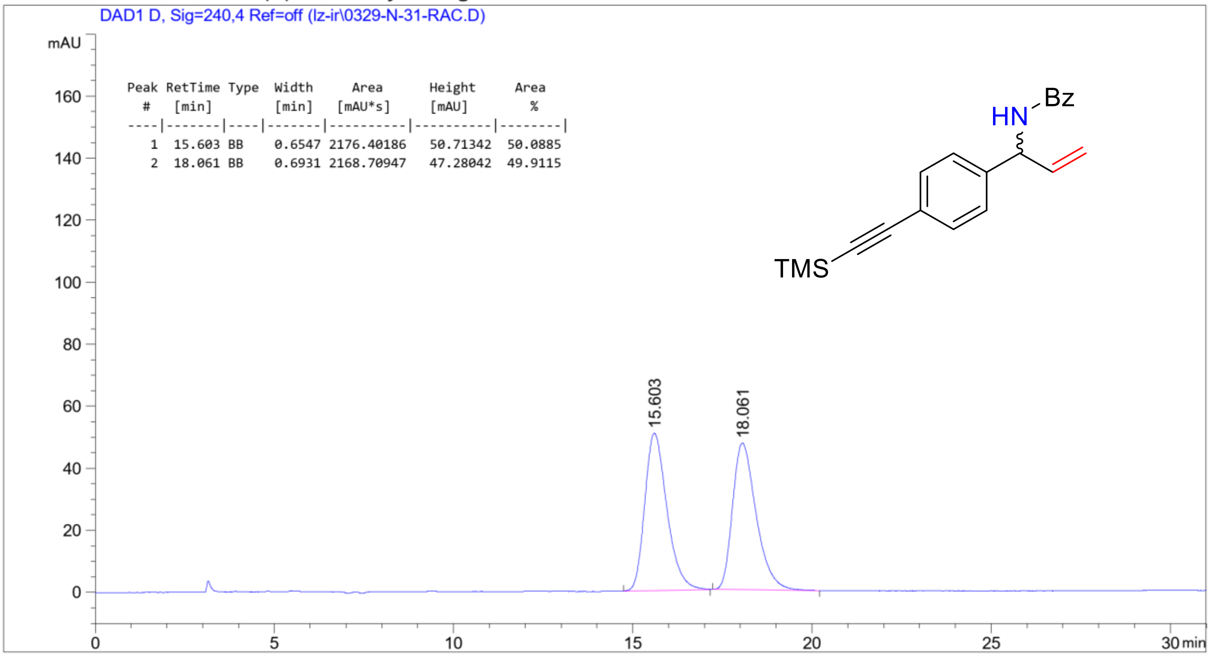


| Peak # | RetTime [min] | Type | Width [min] | Area [mAU*s] | Height [mAU] | Area % |
|---|---|---|---|---|---|---|
| 1 | 15.603 | BB | 0.6547 | 2176.40186 | 50.71342 | 50.0885 |
| 2 | 18.061 | BB | 0.6931 | 2168.70947 | 47.28042 | 49.9115 |

## HPLC Chromatogram of compound 3p *(chiral product)*

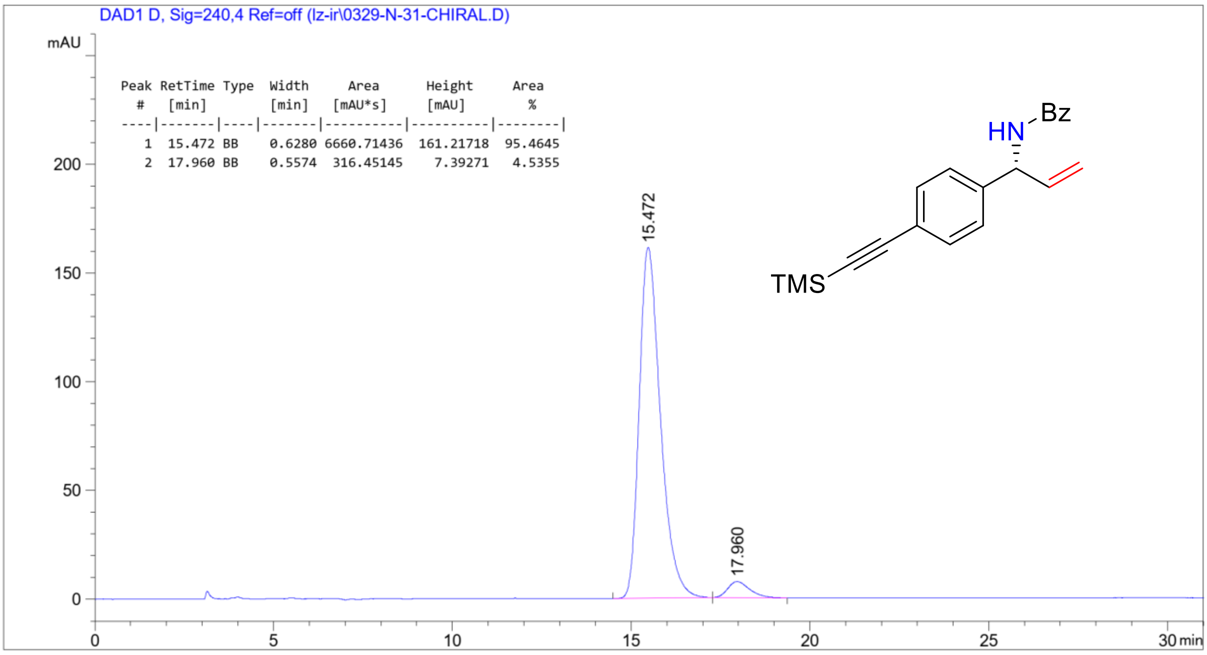


| Peak # | RetTime [min] | Type | Width [min] | Area [mAU*s] | Height [mAU] | Area % |
|---|---|---|---|---|---|---|
| 1 | 15.472 | BB | 0.6280 | 6660.71436 | 161.21718 | 95.4645 |
| 2 | 17.960 | BB | 0.5574 | 316.45145 | 7.39271 | 4.5355 |

## HPLC Chromatogram of compound 3q *(racemic product)*

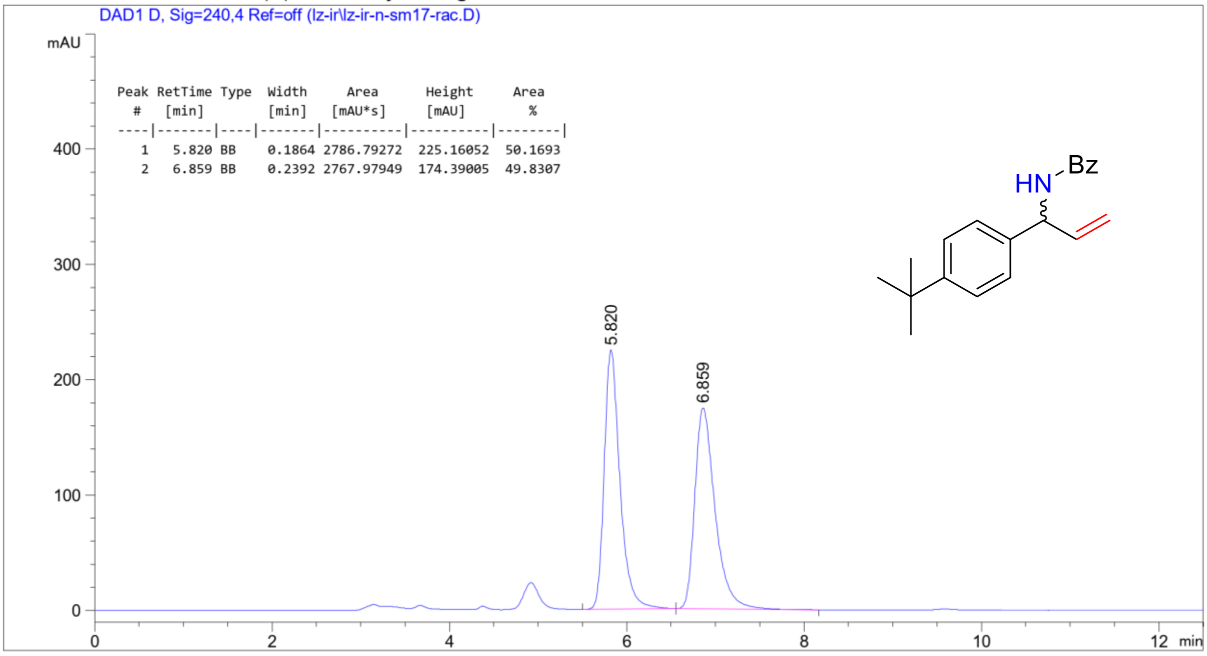


| Peak # | RetTime [min] | Type | Width [min] | Area [mAU*s] | Height [mAU] | Area % |
|---|---|---|---|---|---|---|
| 1 | 5.820 | BB | 0.1864 | 2786.79272 | 225.16052 | 50.1693 |
| 2 | 6.859 | BB | 0.2392 | 2767.97949 | 174.39005 | 49.8307 |

## HPLC Chromatogram of compound 3q *(chiral product)*

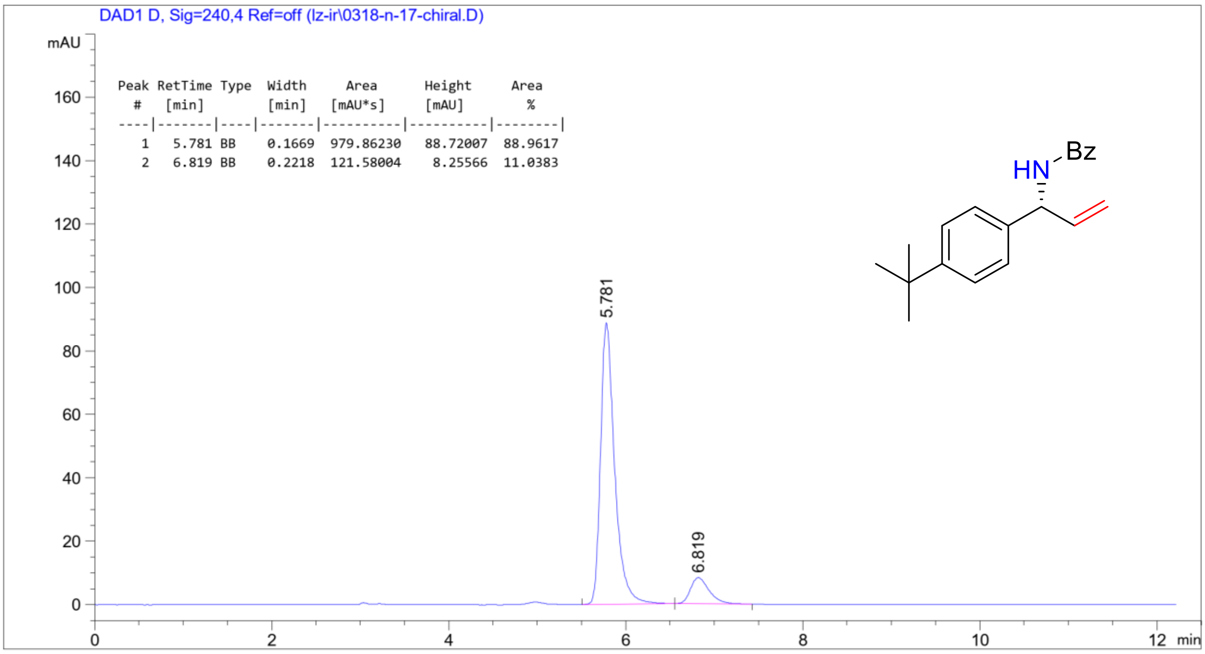


| Peak # | RetTime [min] | Type | Width [min] | Area [mAU*s] | Height [mAU] | Area % |
|---|---|---|---|---|---|---|
| 1 | 5.781 | BB | 0.1669 | 979.86230 | 88.72007 | 88.9617 |
| 2 | 6.819 | BB | 0.2218 | 121.58004 | 8.25566 | 11.0383 |

## HPLC Chromatogram of compound 3r *(racemic product)*

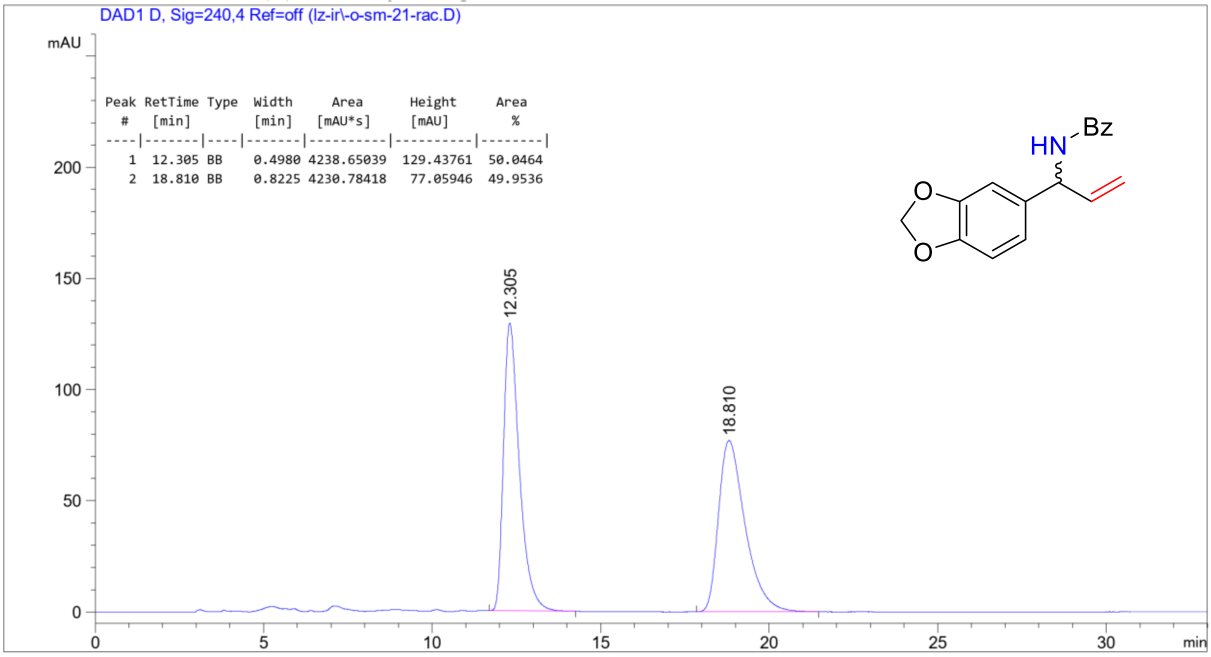


| Peak # | RetTime [min] | Type | Width [min] | Area [mAU*s] | Height [mAU] | Area % |
|---|---|---|---|---|---|---|
| 1 | 12.305 | BB | 0.4980 | 4238.65039 | 129.43761 | 50.0464 |
| 2 | 18.810 | BB | 0.8225 | 4230.78418 | 77.05946 | 49.9536 |

## HPLC Chromatogram of compound 3r *(chiral product)*

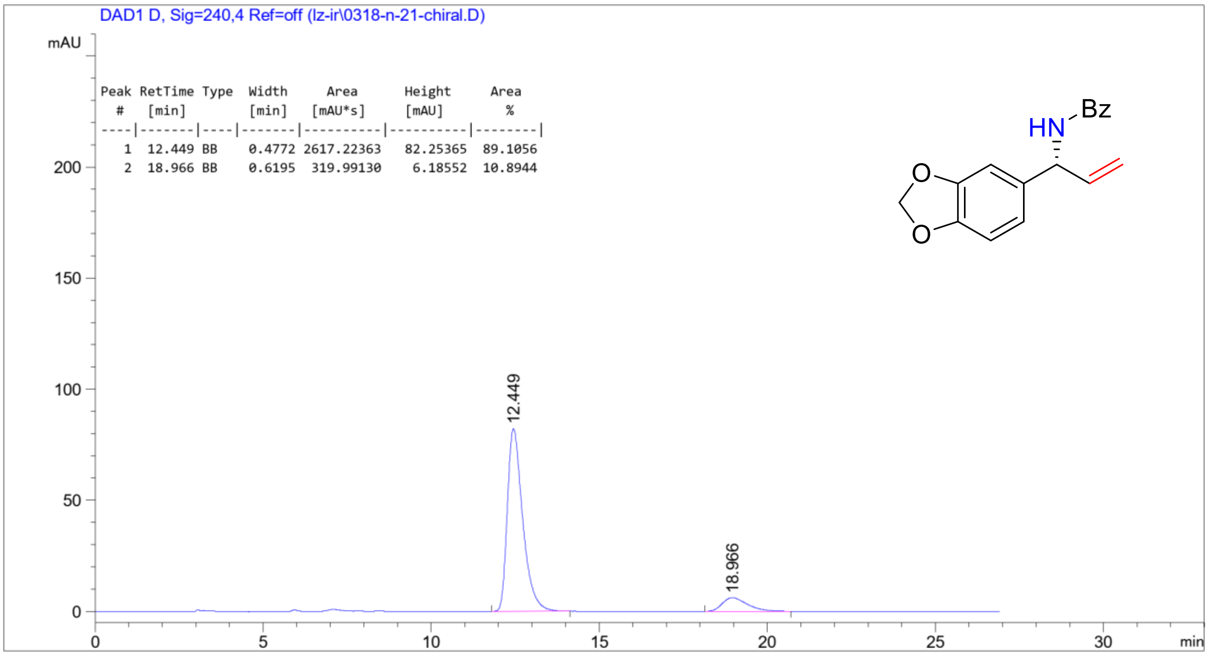


| Peak # | RetTime [min] | Type | Width [min] | Area [mAU*s] | Height [mAU] | Area % |
|---|---|---|---|---|---|---|
| 1 | 12.449 | BB | 0.4772 | 2617.22363 | 82.25365 | 89.1056 |
| 2 | 18.966 | BB | 0.6195 | 319.99130 | 6.18552 | 10.8944 |

## HPLC Chromatogram of compound 3s *(racemic product)*

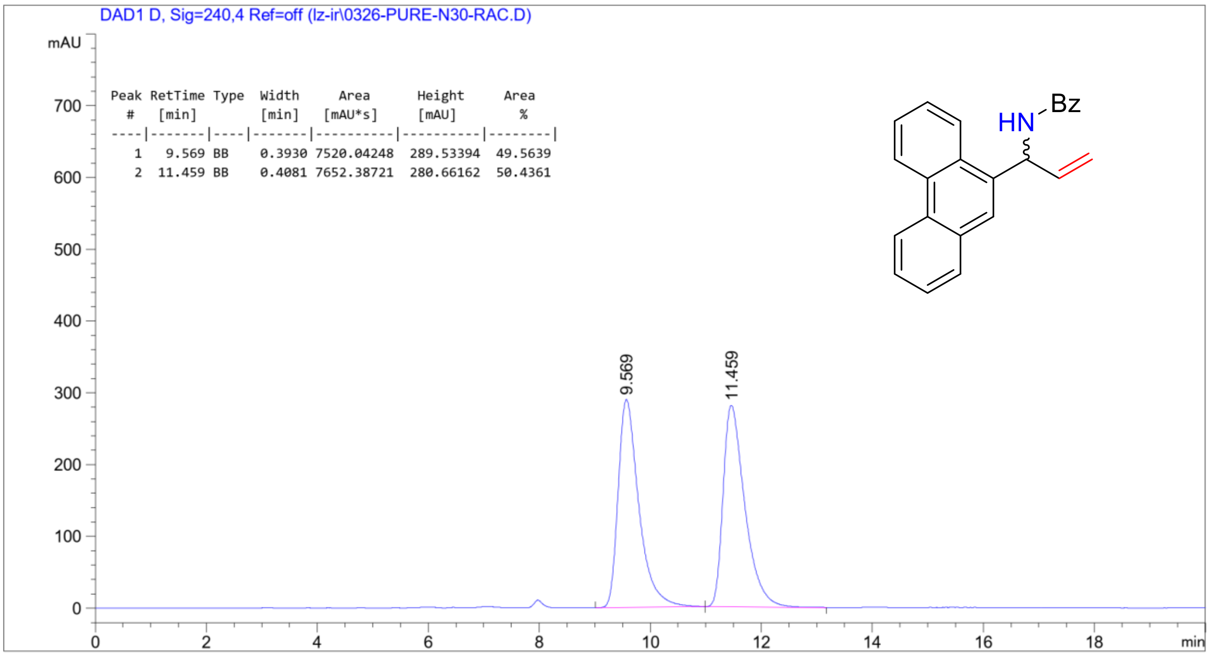


| Peak # | RetTime [min] | Type | Width [min] | Area [mAU*s] | Height [mAU] | Area % |
|---|---|---|---|---|---|---|
| 1 | 9.569 | BB | 0.3930 | 7520.04248 | 289.53394 | 49.5639 |
| 2 | 11.459 | BB | 0.4081 | 7652.38721 | 280.66162 | 50.4361 |

## HPLC Chromatogram of compound 3s *(chiral product)*

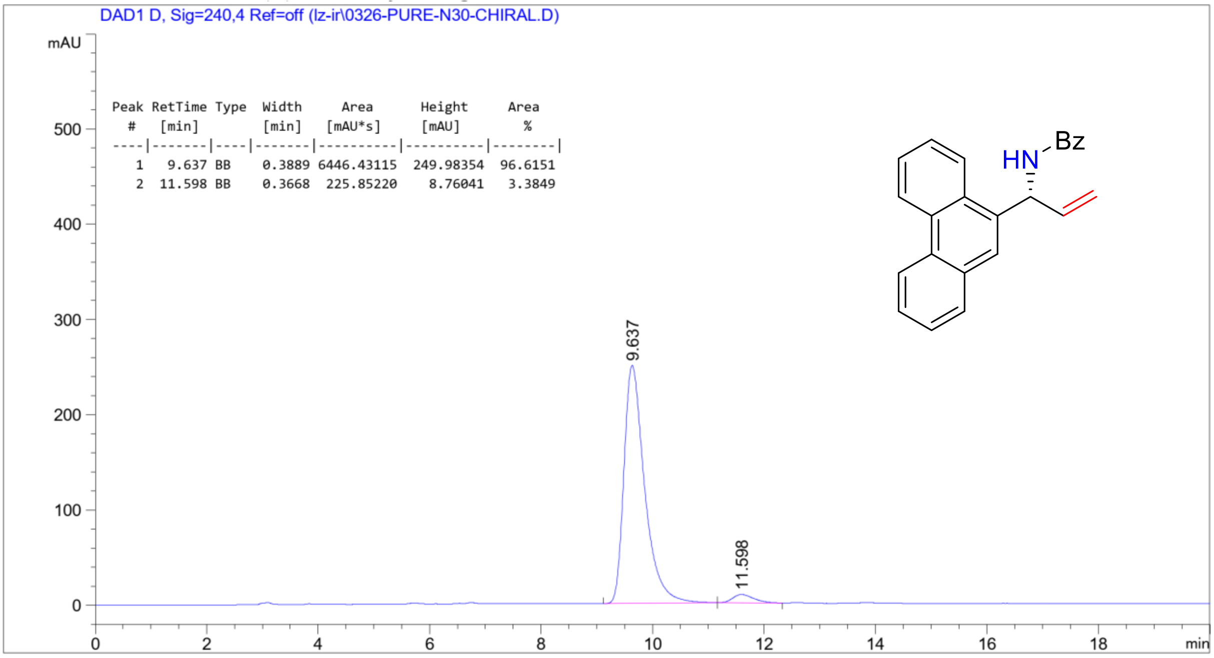


| Peak # | RetTime [min] | Type | Width [min] | Area [mAU*s] | Height [mAU] | Area % |
|---|---|---|---|---|---|---|
| 1 | 9.637 | BB | 0.3889 | 6446.43115 | 249.98354 | 96.6151 |
| 2 | 11.598 | BB | 0.3668 | 225.85220 | 8.76041 | 3.3849 |

## **HPLC Chromatogram of compound 3t** *(racemic product)*

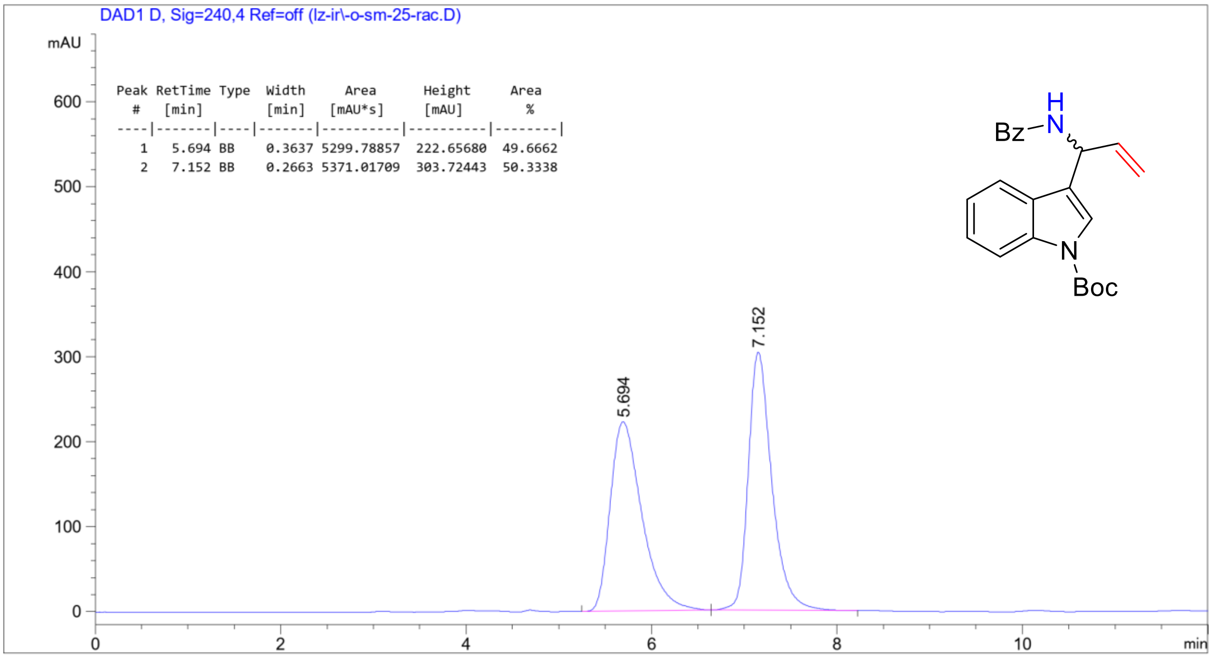


| Peak # | RetTime [min] | Type | Width [min] | Area [mAU*s] | Height [mAU] | Area % |
|---|---|---|---|---|---|---|
| 1 | 5.694 | BB | 0.3637 | 5299.78857 | 222.65680 | 49.6662 |
| 2 | 7.152 | BB | 0.2663 | 5371.01709 | 303.72443 | 50.3338 |

## **HPLC Chromatogram of compound 3t** *(chiral product)*

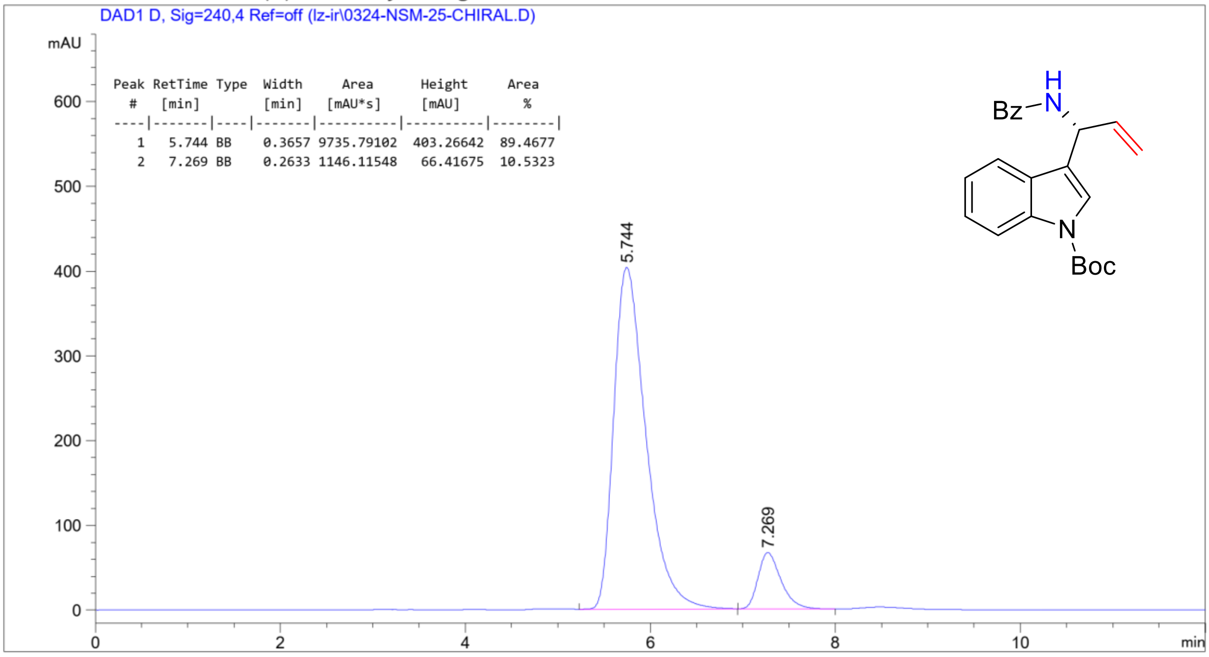


| Peak # | RetTime [min] | Type | Width [min] | Area [mAU*s] | Height [mAU] | Area % |
|---|---|---|---|---|---|---|
| 1 | 5.744 | BB | 0.3657 | 9735.79102 | 403.26642 | 89.4677 |
| 2 | 7.269 | BB | 0.2633 | 1146.11548 | 66.41675 | 10.5323 |

## HPLC Chromatogram of compound 3u *(racemic product)*

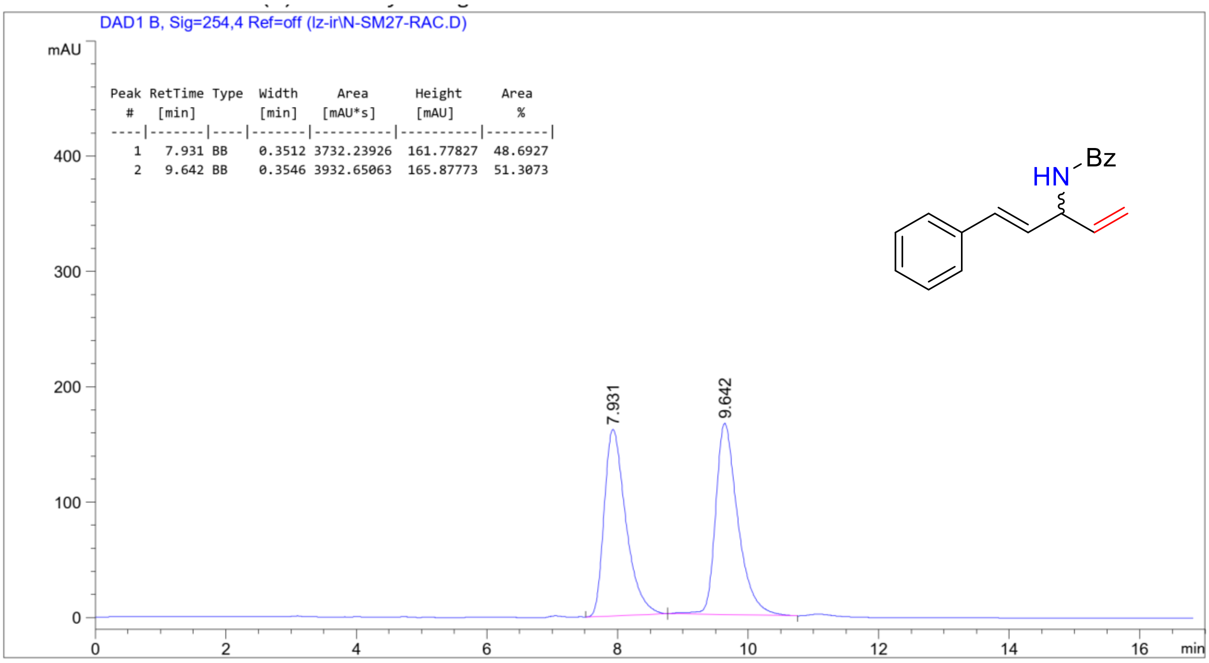


| Peak # | RetTime [min] | Type | Width [min] | Area [mAU*s] | Height [mAU] | Area % |
|---|---|---|---|---|---|---|
| 1 | 7.931 | BB | 0.3512 | 3732.23926 | 161.77827 | 48.6927 |
| 2 | 9.642 | BB | 0.3546 | 3932.65063 | 165.87773 | 51.3073 |

## HPLC Chromatogram of compound 3u *(chiral product)*

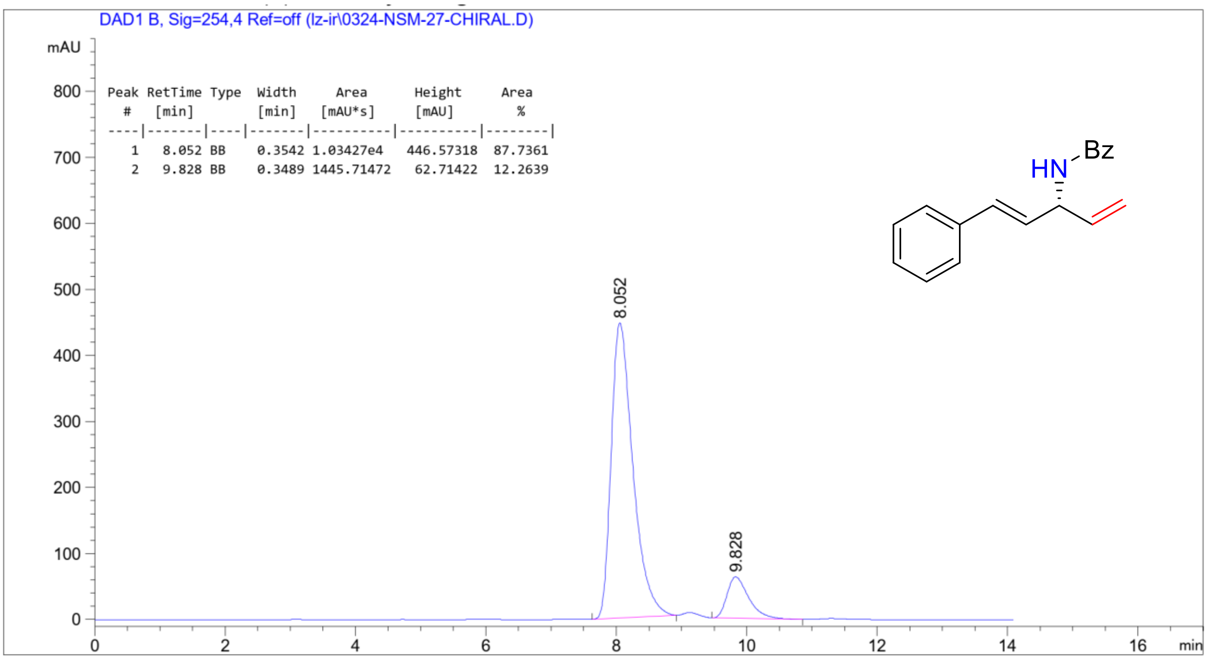


| Peak # | RetTime [min] | Type | Width [min] | Area [mAU*s] | Height [mAU] | Area % |
|---|---|---|---|---|---|---|
| 1 | 8.052 | BB | 0.3542 | 1.03427e4 | 446.57318 | 87.7361 |
| 2 | 9.828 | BB | 0.3489 | 1445.71472 | 62.71422 | 12.2639 |

## HPLC Chromatogram of compound 3v *(racemic product)*

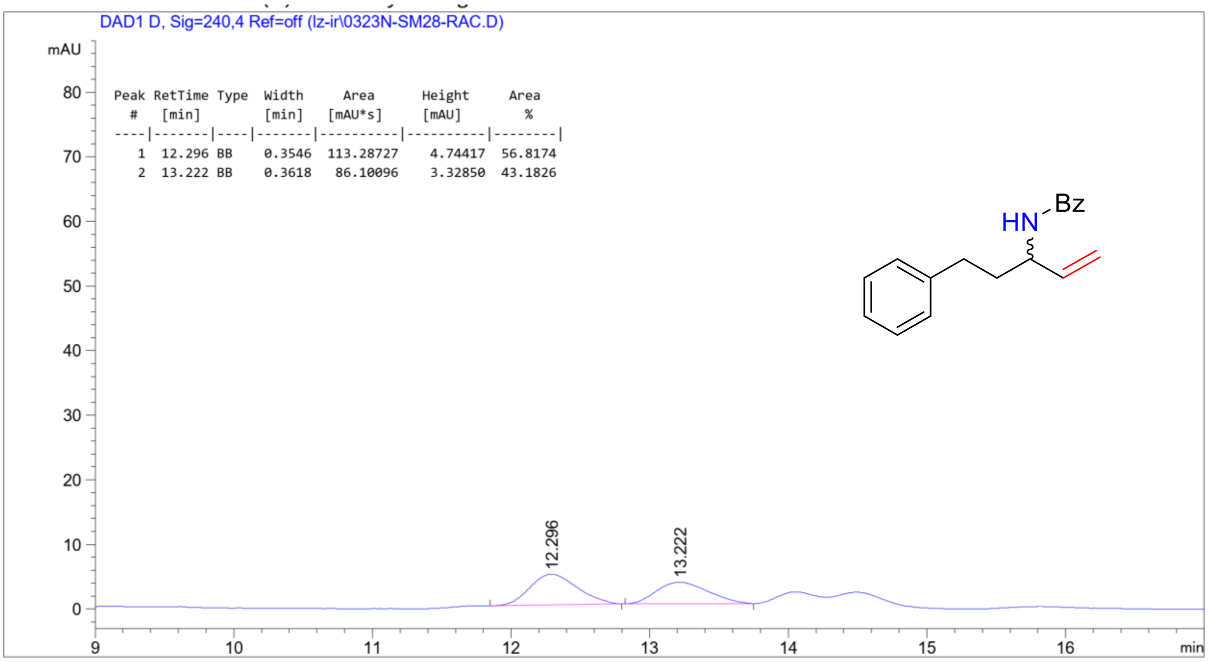


| Peak # | RetTime [min] | Type | Width [min] | Area [mAU*s] | Height [mAU] | Area % |
|---|---|---|---|---|---|---|
| 1 | 12.296 | BB | 0.3546 | 113.28727 | 4.74417 | 56.8174 |
| 2 | 13.222 | BB | 0.3618 | 86.10096 | 3.32850 | 43.1826 |

## HPLC Chromatogram of compound 3v *(chiral product)*

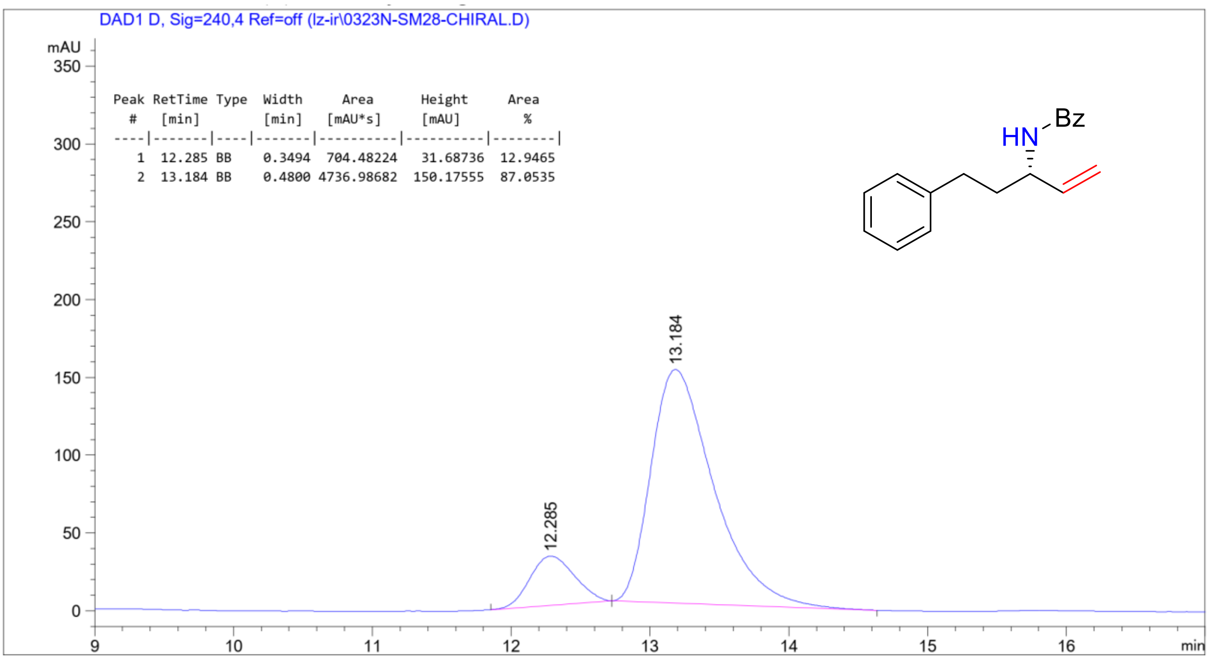


| Peak # | RetTime [min] | Type | Width [min] | Area [mAU*s] | Height [mAU] | Area % |
|---|---|---|---|---|---|---|
| 1 | 12.285 | BB | 0.3494 | 704.48224 | 31.68736 | 12.9465 |
| 2 | 13.184 | BB | 0.4800 | 4736.98682 | 150.17555 | 87.0535 |

## **HPLC Chromatogram of compound 3w** *(racemic product)*

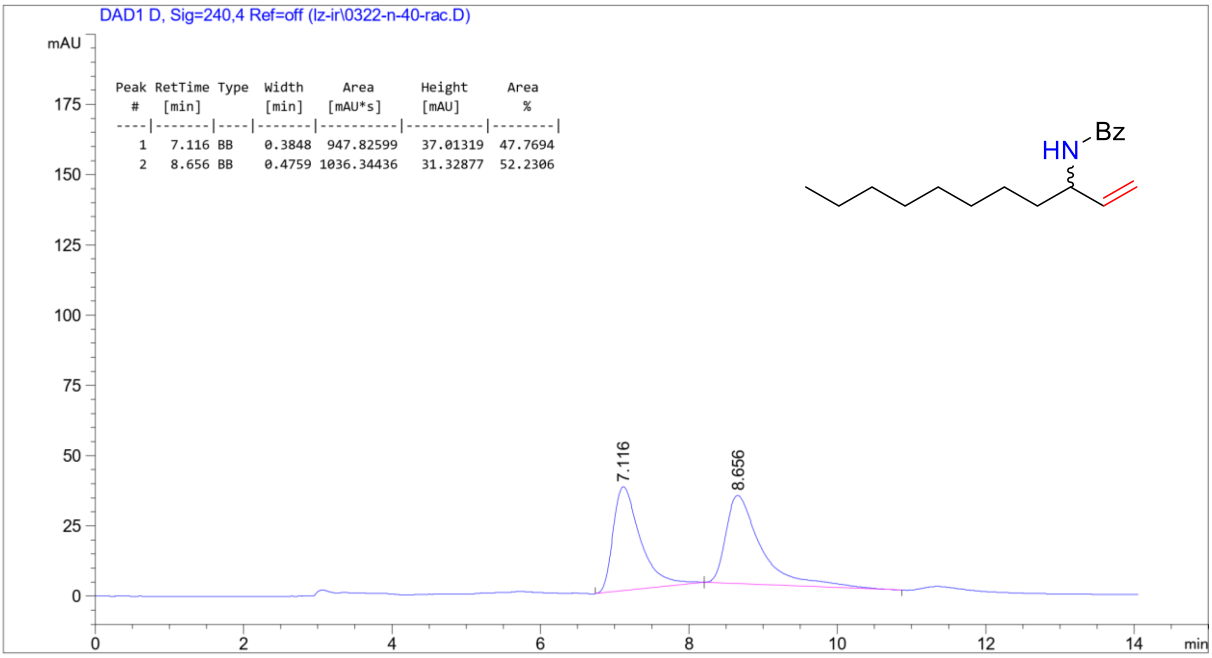


| Peak # | RetTime [min] | Type | Width [min] | Area [mAU*s] | Height [mAU] | Area % |
|---|---|---|---|---|---|---|
| 1 | 7.116 | BB | 0.3848 | 947.82599 | 37.01319 | 47.7694 |
| 2 | 8.656 | BB | 0.4759 | 1036.34436 | 31.32877 | 52.2306 |

## **HPLC Chromatogram of compound 3w** *(chiral product)*

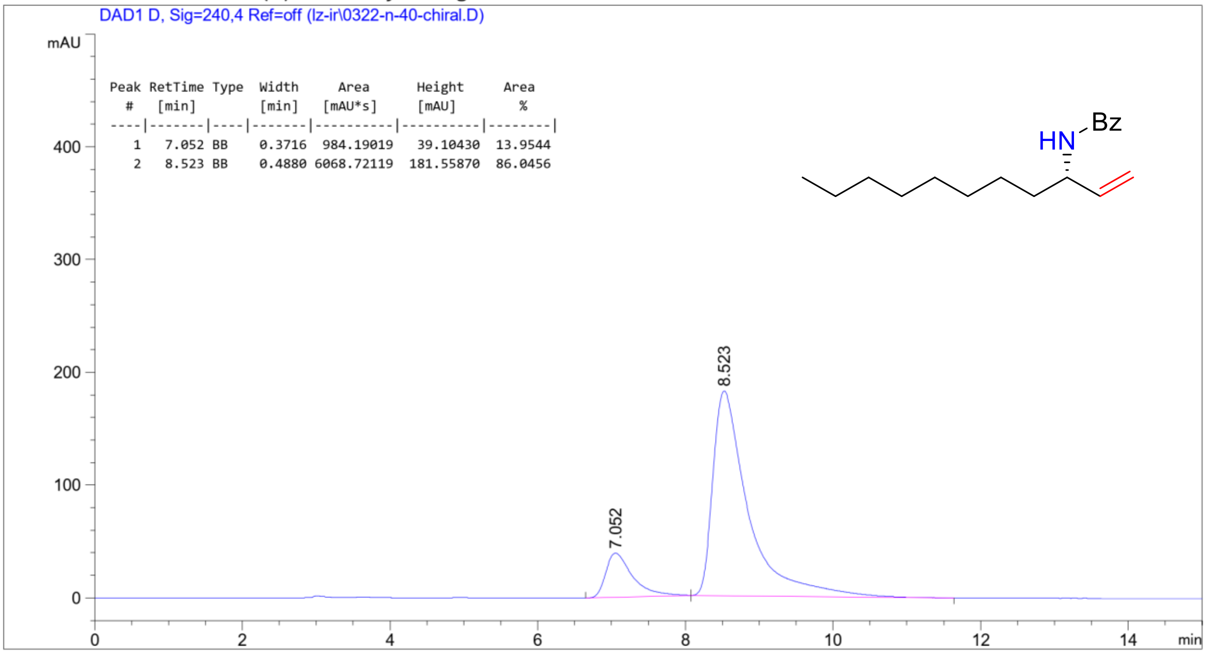


| Peak # | RetTime [min] | Type | Width [min] | Area [mAU*s] | Height [mAU] | Area % |
|---|---|---|---|---|---|---|
| 1 | 7.052 | BB | 0.3716 | 984.19019 | 39.10430 | 13.9544 |
| 2 | 8.523 | BB | 0.4880 | 6068.72119 | 181.55870 | 86.0456 |

## HPLC Chromatogram of compound 3x *(racemic product)*

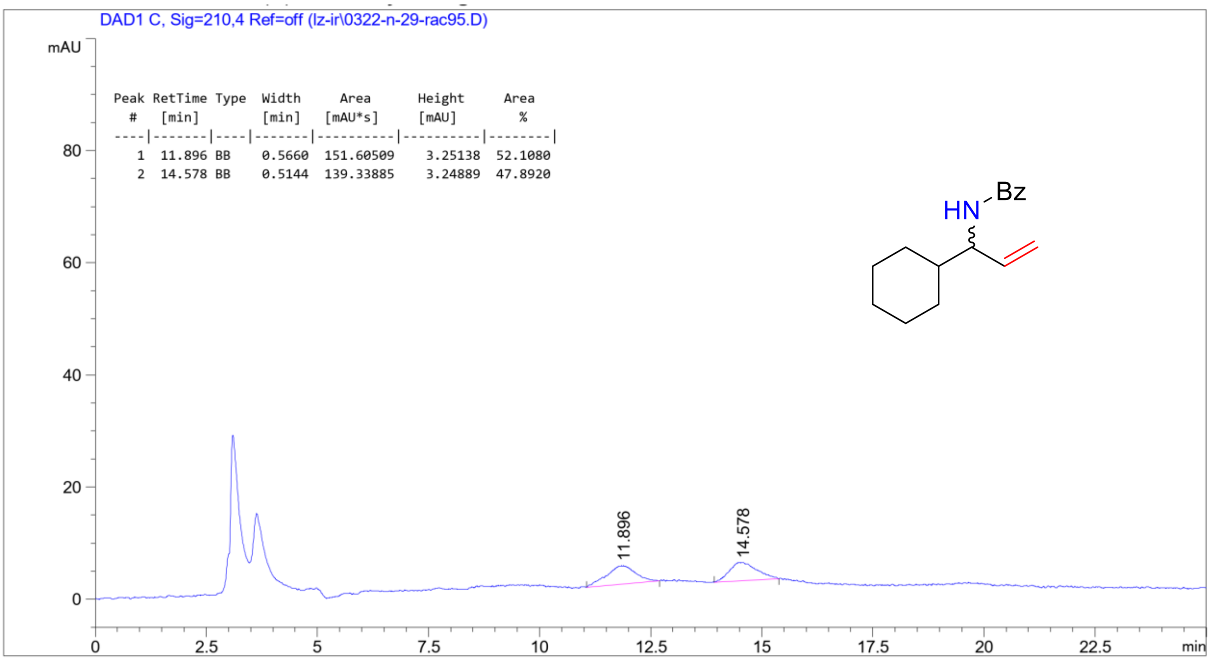


| Peak # | RetTime [min] | Type | Width [min] | Area [mAU*s] | Height [mAU] | Area % |
|---|---|---|---|---|---|---|
| 1 | 11.896 | BB | 0.5660 | 151.60509 | 3.25138 | 52.1080 |
| 2 | 14.578 | BB | 0.5144 | 139.33885 | 3.24889 | 47.8920 |

## HPLC Chromatogram of compound 3x *(chiral product)*

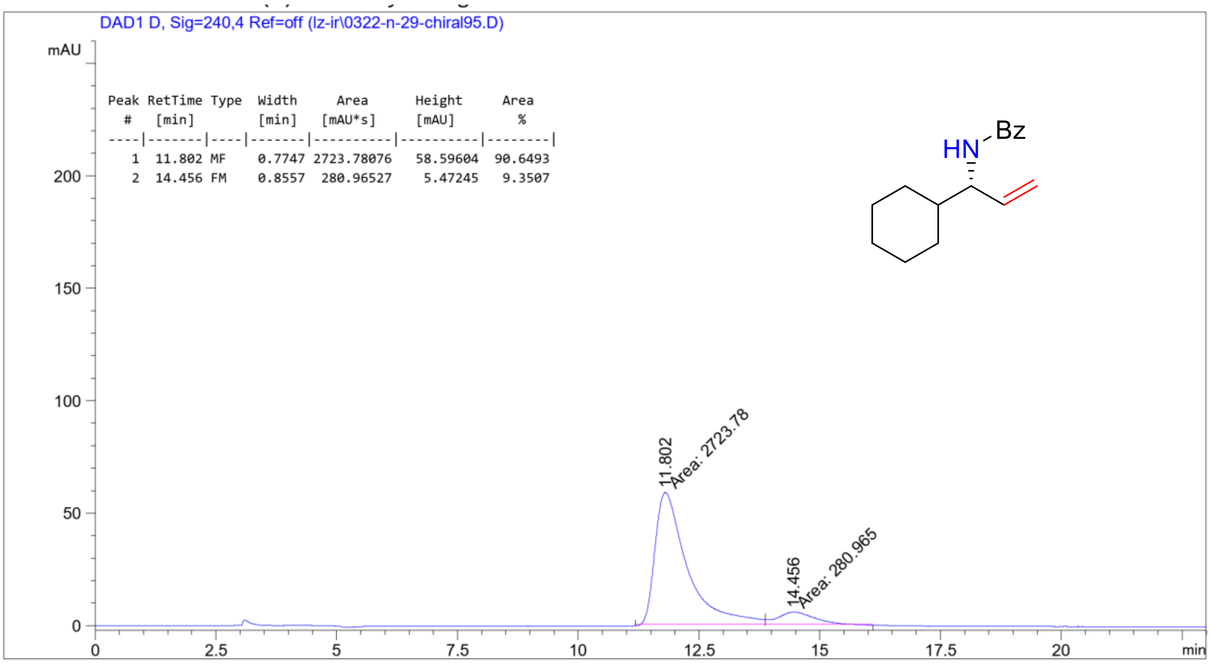


| Peak # | RetTime [min] | Type | Width [min] | Area [mAU*s] | Height [mAU] | Area % |
|---|---|---|---|---|---|---|
| 1 | 11.802 | MF | 0.7747 | 2723.78076 | 58.59604 | 90.6493 |
| 2 | 14.456 | FM | 0.8557 | 280.96527 | 5.47245 | 9.3507 |

## HPLC Chromatogram of compound 3y *(racemic product)*

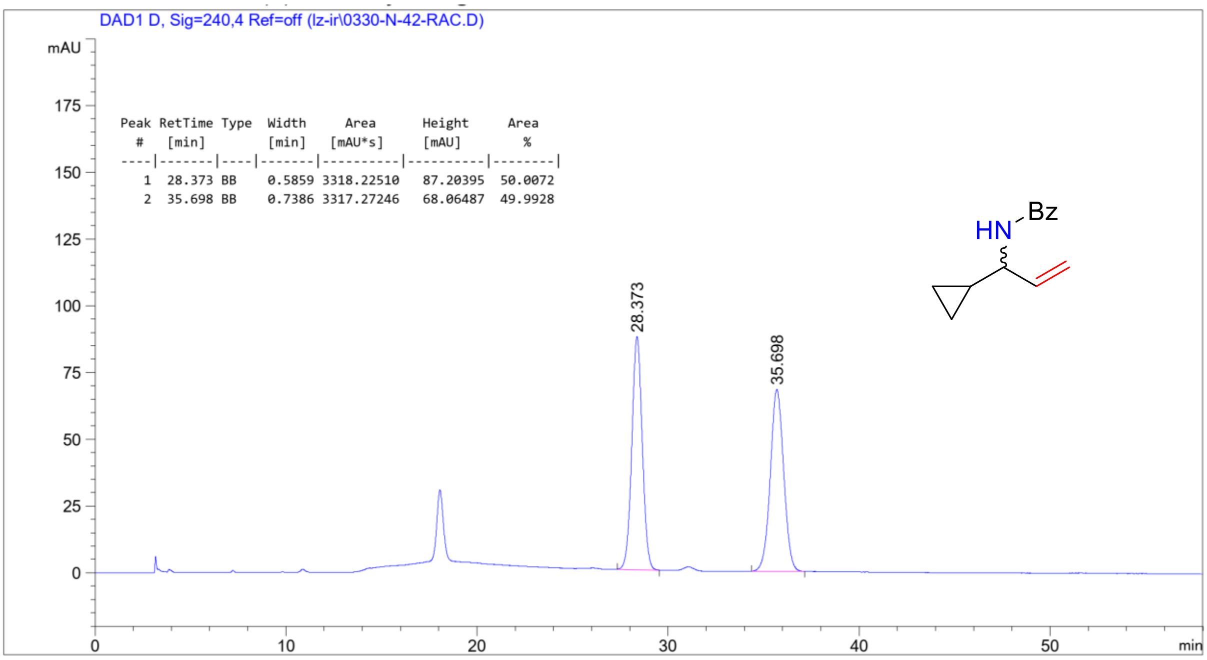


## HPLC Chromatogram of compound 3y *(chiral product)*

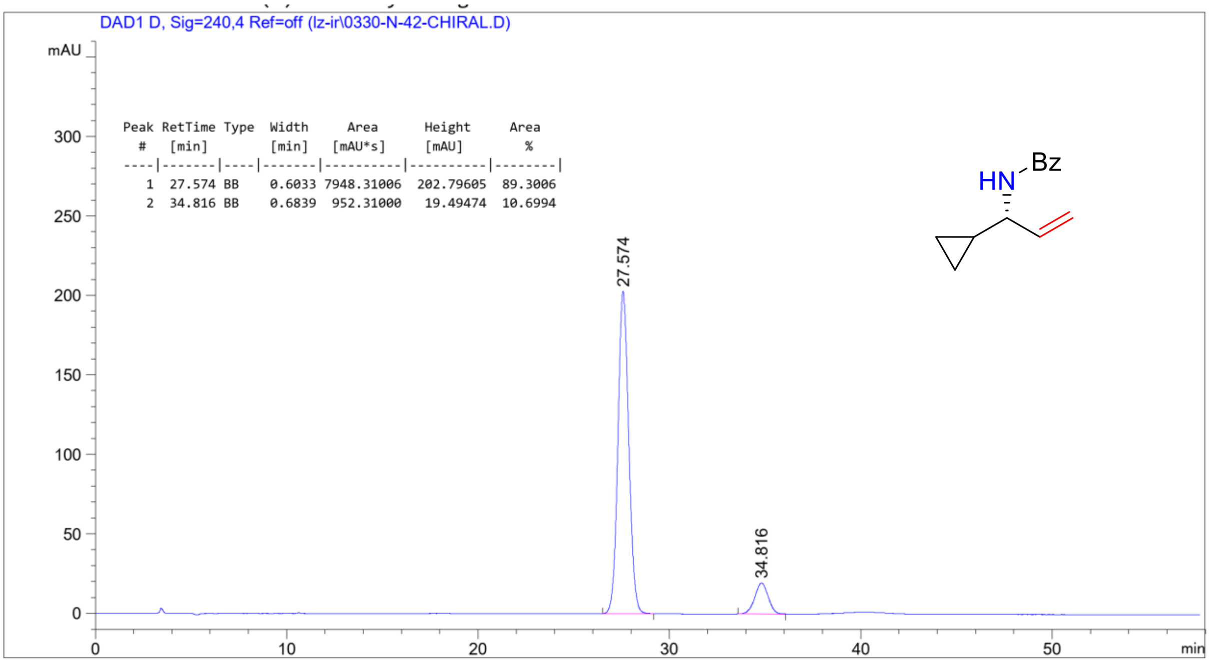

## HPLC Chromatogram of compound 3z *(racemic product)*

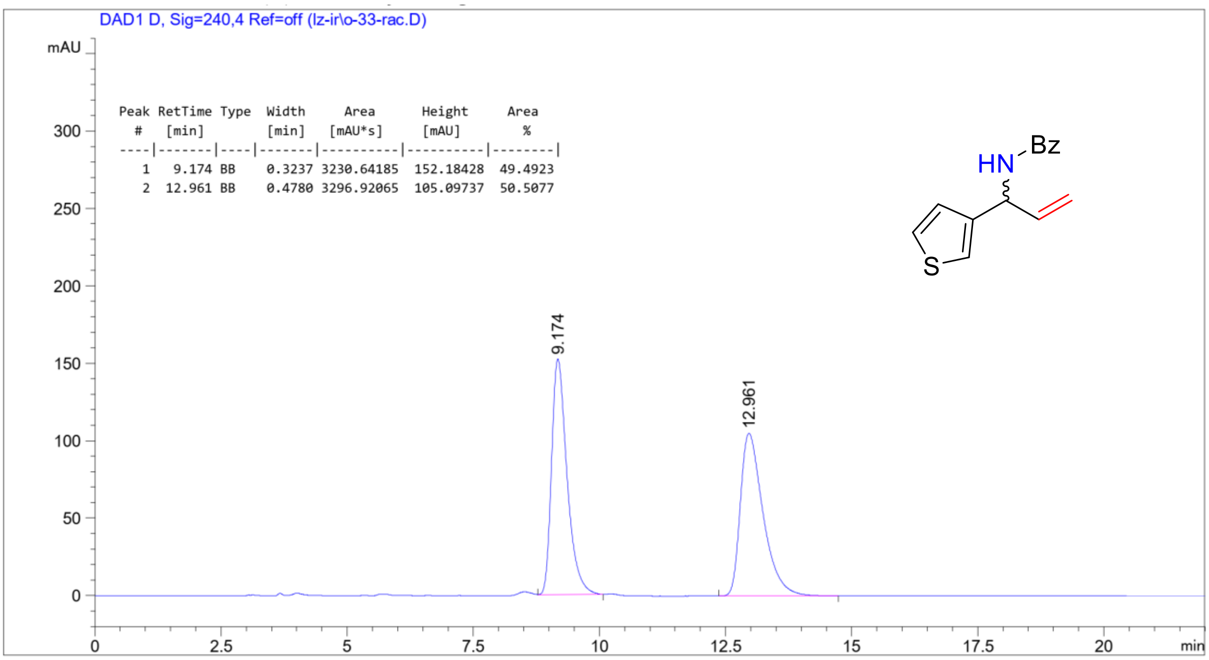


## HPLC Chromatogram of compound 3z *(chiral product)*

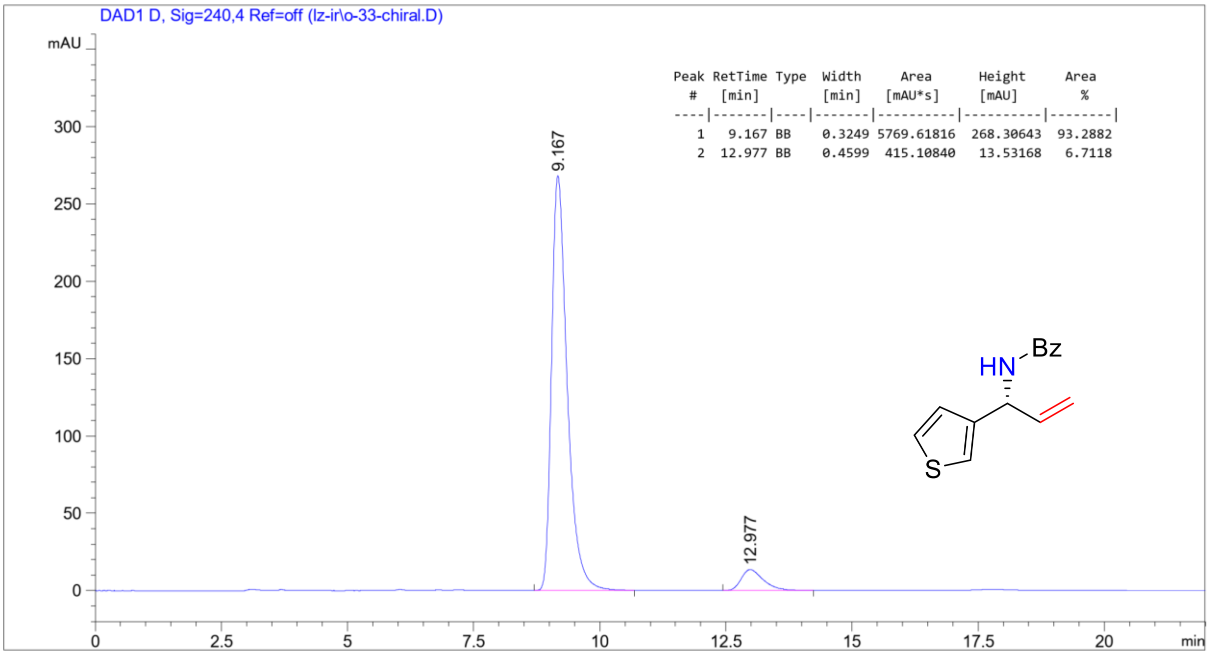

## HPLC Chromatogram of compound 1a *(racemic product)*

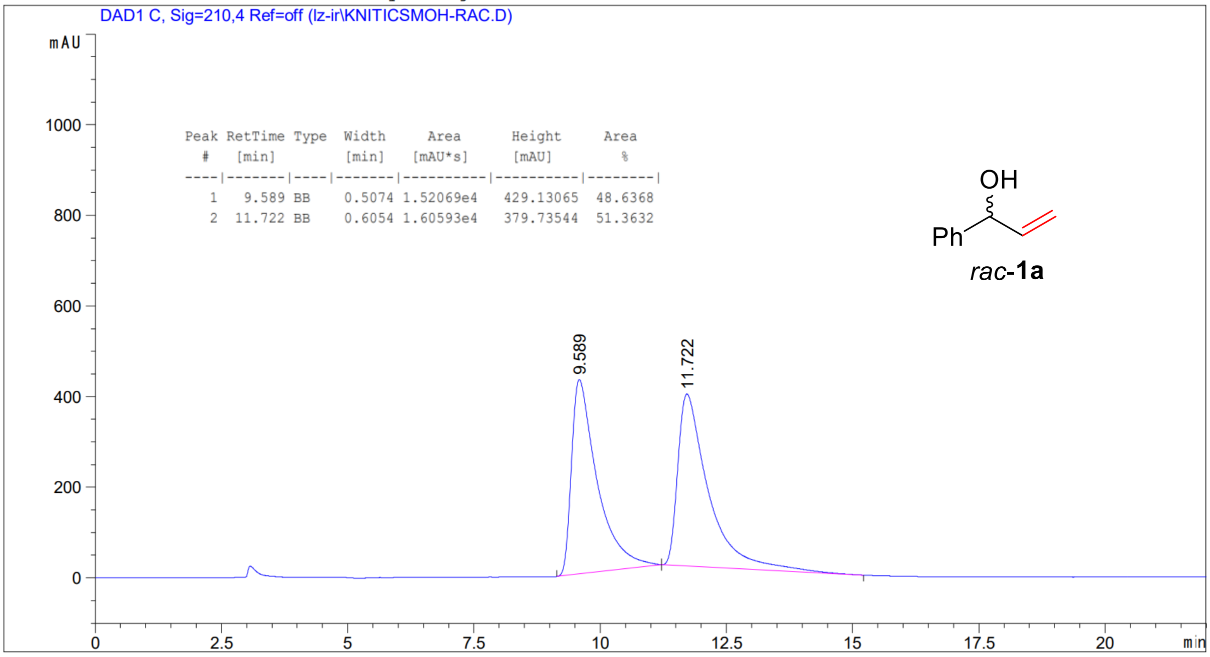


## HPLC Chromatogram of compound (-)-1a*(chiral product)*

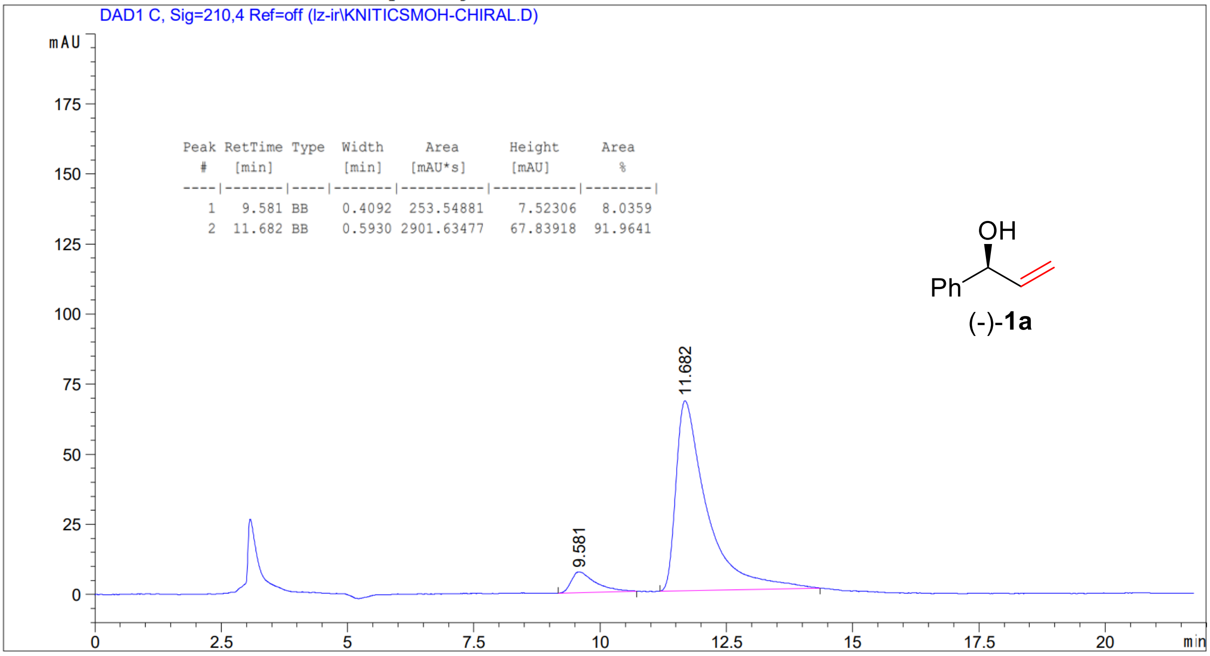

Supplement: Supplementary file 1 — jo2c02589_si_001.pdf [file jo2c02589_si_001.pdf]
